# Supplementary material for: Pestalotioid Species Associated with Medicinal Plants in Southwest China and Thailand
Source: Microbiol Spectr. 2023 Jan 10;11(1):e03987-22. doi: 10.1128/spectrum.03987-22 (PMC9927317; doi:10.1128/spectrum.03987-22)
Supplement: Supplemental file 1 — Supplemental material. Download spectrum.03987-22-s0001.pdf, PDF file, 0.5 MB [file spectrum.03987-22-s0001.pdf]

Neopestalotiopsis alignment

>AH11

CATTATAGAGTTTTCTAAACTCCCAACCCATGTGAACTTACCTTTTGTTGCCTCGGCAGAAGTTAT
AGGTCTTCTTATAGCTGCTGCCGGTGGACCATTAAACTCTTGTTATTTTATGTAATCTGAGCGTCT
TATTTTAATAAGTCAAAACTTTCAACAACGGATCTCTTGGTTCTGGCATCGATGAAGAACGCAGC
GAAATGCGATAAGTAATGTGAATTGCAGAATTCAGTGAATCATCGAATCTTTGAACGCACATTG
CGCCCATTAGTATTCTAGTGGGCATGCCTGTTCGAGCGTCATTTCAACCCTTAAGCCTAGCTTAG
TGTTGGGAATCTACTTCTCTTAGGAGTTGTAGTTCCTGAAATACAACGGCGGATTTGTAGTATCC
TCTGAGCGTAGTAA-
TTTTTTTCTCGCTTTTGTTAGGTGCTATAACTCCCAGCCGCTAAACCCCCAATTTTTTGTGGTTGAC
CTCGGATCAGGT???????????????TATTGATTCCCATCAT-------CATTCCCCTTCTC-
TTCAGCGTCATGATTTTCAACCTACGCGTTGAAAATTA-TTTTCGCTCCTTCCACAC--TTTTT--
TCGCTGGTTACCCCGCCGCGAGGCACCCGCACGACCCCGCGGTGCAAACGAAAAATTTCTTAT
CACAGCCCCACCTTTCACAAGCAACCATGCATTGCTCATGAGACCCACTT--
TGAACGATTGCTAATGCCTTCATACAGGAAGCCGCCGAGCTCGGTAAGGGTTCCTTCAAGTACG
CCTGGGTTCTTGACAAGCTCAAGGCCGAGCGTGAGCGTGGTATCACCATCGATATCGCTCTCTG
GAAGTTCGAGACCAACGAGTACAATGTCACCGTCATTGGTTAGTACCCCTCCACCTATGCCATG
TGCTGCTCCATAAGACACTTGACTAACCTTGCTTCATAGACGCTCCCGGTCACCGTGATTTCATC
AAGAACATGCTGCCTTCTGGTATGTAATCTGTCTGCCTCGACACGGCCTCAATACGACGTTTTTC
GTGCCTGCACGACGGCCCCGATCAG-
TGAATTAGGTCAAGATACAGGGAACATGACGCTAATAGGTCATTTATAGGCAAACCATCTCTGG
CGAGCACGGTCTCGACAGCAATGGAGTGTATGTACTATTTTCAATTCCTCCTGCTTCCTGTTGAG
CTTGTAGGCTGAC-
TCGATGGCCATTTAGCTACAACGGTACCTCCGAGCTCCAGCTCGAGCGTATGAGCGTCTACTTC
AACGAGGCTTCGGGCAACAAGTACGTTCCTCGTGCCGTCCTCGTCGATCTCGAGCCCGGTACCA
TGGATGCCGTCCGCGCCGGTCCCTTCGGCCAGCTCTTCCGCC???????????????????????????????
?????????????

>AH9

CATTATAGAGTTTTCTAAACTCCCAACCCATGTGAACTTACCTTTTGTTGCCTCGGCAGAAGTTAT
AGGTCTTCTTATAGCTGCTGCCGGTGGACCATTAAACTCTTGTTATTTTATGTAATCTGAGCGTCT
TATTTTAATAAGTCAAAACTTTCAACAACGGATCTCTTGGTTCTGGCATCGATGAAGAACGCAGC
GAAATGCGATAAGTAATGTGAATTGCAGAATTCAGTGAATCATCGAATCTTTGAACGCACATTG
CGCCCATTAGTATTCTAGTGGGCATGCCTGTTCGAGCGTCATTTCAACCCTTAAGCCTAGCTTAG
TGTTGGGAATCTACTTCTCTTAGGAGTTGTAGTTCCTGAAATACAACGGCGGATTTGTAGTATCC
TCTGAGCGTAGTAA-
TTTTTTTCTCGCTTTTGTTAGGTGCTATAACTCCCAGCCGCTAAACCCCCAATTTTTTGTGGTTGAC
CTCGGATCAGGTAGAAGGTTAGTCATCTACTGATTCCCTTCAT-------
CATTCTCCTTCACACTTCATGCCATGATTTTCAACCTACATGTTGAAAATTA-
TTTTCGCTCCTTCCACAC--TTTTT--
TCGCTGGTTACCCCGCCGCGAGGCACCCGCACGACCCCGCGGTGCAAACGAAAAATTTCTTAT
CACAGCCCCACCTTGCATAAGCAACCATGCATTGCTCATGGGATCCACTT--
TGAATAATCGCTAATGCCTTCATACAGGAAGCCGCCGAGCTCGGTAAGGGTTCCTTCAAGTACG
CCTGGGTTCTTGACAAGCTCAAGGCCGAGCGTGAGCGTGGTATCACCATCGATATCGCTCTCTG
GAAGTTCGAGACCAACGAGTACAATGTCACCGTCATTGGTTAGTACCCCTCCACCTATGCCATG

TGCTGCTCCATAAGACACTTGACTAACCTTGCTTTATAGACGCTCCCGGTCACCGT?????????????
???TGCTGCCTTCTGGTATGTAATCTGTCTGCCTCGACACGGCCTTAATACGACGTTTTTCGTGCC
TGCACGACGGCCCCGATCAG-
TGAATTAGGTCAAGATACAGGGAACATGATGCTAATAGGTCATTTATAGGCAAACCATCTCTGG
CGAGCACGGTCTCGACAGCAATGGAGTGTATGTACTATTTTCAATTCCTCCTGCTTCCTGTTGAG
CTTGTAGGCTGAC-
TCGATGGCCATTTAGCTACAACGGTACCTCCGAGCTCCAGCTCGAGCGTATGAGCGTCTACTTC
AACGAGGCTTCCGGCAACAAGTACGTTCCTCGTGCCGTCCTCGTCGATCTCGAGCCCGGTACCA
TGGATGCCGTCCGCGCCGGTCCCTTCGGCCAGCTCTTCCGCCCTGACAACTTC????????????????
?????????????????
>AH9_1
CATTATAGAGTTTTCTAAACTCCCAACCCATGTGAACTTACCTTTTGTTGCCTCGGCAGAAGTTAT
AGGTCTTCTTATAGCTGCTGCCGGTGGACCATTAAACTCTTGTTATTTTATGTAATCTGAGCGTCT
TATTTTAATAAGTCAAAACTTTCAACAACGGATCTCTTGGTTCTGGCATCGATGAAGAACGCAGC
GAAATGCGATAAGTAATGTGAATTGCAGAATTCAGTGAATCATCGAATCTTTGAACGCACATTG
CGCCCATTAGTATTCTAGTGGGCATGCCTGTTCGAGCGTCATTTCAACCCTTAAGCCTAGCTTAG
TGTTGGGAATCTACTTCTCTTAGGAGTTGTAGTTCCTGAAATACAACGGCGGATTTGTAGTATCC
TCTGAGCGTAGTAA-
TTTTTTTCTCGCTTTTGTTAGGTGCTATAACTCCCAGCCGCTAAACCCCCAATTTTTTGTGGTTGAC
CTCGGATCAGGTAGAAGGTTAGTCATCTACTGATTCCCTTCAT-------
CATTCTCCTTCACACTTCATGCCATGATTTTCAACCTACATGTTGAAAATTA-
TTTTCGCTCCTTCCACAC--TTTTT--
TCGCTGGTTACCCCGCCGCGAGGCACCCGCACGACCCCGCGGTGCAAACGAAAAATTTCTTAT
CACAGCCCCACCTTGCATAAGCAACCATGCATTGCTCATGGGATCCACTT--
TGAATAATCGCTAATGCCTTCATACAGGAAGCCGCCGAGCTCGGTAAGGGTTCCTTCAAGTACG
CCTGGGTTCTTGACAAGCTCAAGGCCGAGCGTGAGCGTGGTATCACCATCGATATCGCTCTCTG
GAAGTTCGAGACCAACGAGTACAATGTCACCGTCATTGGTTAGTACCCCTCCACCTATGCCATG
TGCTGCTCCATAAGACACTTGACTAACCTTGCTTTATAGACGCTCCCGGTCACCGT?????????????
???TGCTGCCTTCTGGTATGTAATCTGTCTGCCTCGACACGGCCTTAATACGACGTTTTTCGTGCC
TGCACGACGGCCCCGATCAG-
TGAATTAGGTCAAGATACAGGGAACATGATGCTAATAGGTCATTTATAGGCAAACCATCTCTGG
CGAGCACGGTCTCGACAGCAATGGAGTGTATGTACTATTTTCAATTCCTCCTGCTTCCTGTTGAG
CTTGTAGGCTGAC-
TCGATGGCCATTTAGCTACAACGGTACCTCCGAGCTCCAGCTCGAGCGTATGAGCGTCTACTTC
AACGAGGCTTCCGGCAACAAGTACGTTCCTCGTGCCGTCCTCGTCGATCTCGAGCCCGGTACCA
TGGATGCCGTCCGCGCCGGTCCCTTCGGCCAGCTCTTCCGCCCTGACAACTTC????????????????
?????????????????
>BB1
?????????????????????CCCAACCCATGTGAACTTACCTTTTGTTGCCTCGGCAGAAGTTATAGGTC
TTCTTATAGCTGCTGCCGGTGGACCATTAAACTCTTGTTATTTTATGTAATCTGAGCGTCTTATTTT
AATAAGTCAAAACTTTCAACAACGGATCTCTTGGTTCTGGCATCGATGAAGAACGCAGCGAAAT
GCGATAAGTAATGTGAATTGCAGAATTCAGTGAATCATCGAATCTTTGAACGCACATTGCGCCC
ATTAGTATTCTAGTGGGCATGCCTGTTCGAGCGTCATTTCAACCCTTAAGCCTAGCTTAGTGTTG
GGAATCTACTTCTCTTAGGAGTTGTAGTTCCTGAAATACAACGGCGGATTTGTAGTATCCTCTGA

GCGTAGTAA-
TTTTTTTCTCGCTTTTGTTAGGTGCTATAACTCCCAGCCGCTAAACCCCCAATTTTTTGTGGTTGAC
CTCGGATCAGGTAGAAGGTTAGTCATCTGTTGATTCCTATCAT--------CATTCCCTTCAC-
ATCAGCGTCATGATTTTCAACCTACGTGTTGAAAATTA-TTTTCGCTCCTTCCACAC--TTTTT--
TCGCTGGTTACCCCGCCGCGAGGCACCCGCACGACCCCGCGGTGCAAACGAAAAATTTCTTAT
CACAGCCCCACCTTGCACAAGCAACCATGCATTGCTCATGAGACCCACTT--
TGAACAATTGCTAATGCCTTCATACAGGAAGCCGCCGAGCTTGGTAAGGGTTCCTTCAAGTACG
CCTGGGTTCTTGACAAGCTCAAGGCCGAGCGTGAGCGTGGTATCACCATCGATATCGCTCTCTG
GAAGTTCGAGACCAACGAGTACAATGTCACCGTCATTGGTTAGTACCCCTCCACCAATGCCATA
TGCTGCTCCATAAGACACTTGACTAACCTTACTTCATAGACGCTCCCGGTCACCGTGATTTCATC
AAGAACATGCTGCCTTCTGGTATGTAATCTGTCTGCCTCGACACGGCCTCAATACGACGTTTTTC
GTGCCTGCACGACGGCCCCGATCAG-
TGAATTAGGTCAAGATACAGGGAACATGACGCTAATAGGTCATTTATAGGCAAACCATCTCTGG
CGAGCACGGTCTCGACAGCAATGGAGTGTATGTACTATTTTCAATTCCTCCTGCTTCCTGTTGAG
CTTGTAGGCTGAC-
TCGATGGCCATTTAGCTACAACGGTACCTCCGAGCTCCAGCTCGAGCGTATGAGCGTCTACTTC
AACGAGGCTTCCGGCAACAAGTACGTTCCTCGTGCCGTCCTCGTCGATCTCGAGCCCGGTACCA
TGGATGCCGTCCGCGCCGGTCCCTTCGGCCAGCTCTTCCGCCCTGACAACTTCGTCTTCGG?????
????????????????????
>CL1_2
????????AGTTTTCTAAACTCCCAACCCATGTGAACTTACCTTTTGTTGCCTCGGCAGAAGTTATAG
GTCTTCTTATAGCTGCTGCCGGTGGACCATTAAACTCTTGTTATTTTATGTAATCTGAGCGTCTTA
TTTTAATAAGTCAAAACTTTCAACAACGGATCTCTTGGTTCTGGCATCGATGAAGAACGCAGCGA
AATGCGATAAGTAATGTGAATTGCAGAATTCAGTGAATCATCGAATCTTTGAACGCACATTGCG
CCCATTAGTATTCTAGTGGGCATGCCTGTTCGAGCGTCATTTCAACCCTTAAGCCTAGCTTAGTG
TTGGGAATCTACTTCTCTTAGGAGTTGTAGTTCCTGAAATACAACGGCGGATTTGTAGTATCCTC
TGAGCGTAGTAA-
TTTTTTTCTCGCTTTTGTTAGGTGCTATAACTCCCAGCCGCTAAACCCCCAATTTTTTGTGGTTGAC
CTCGGATCAGGTAGAAGGTTAGTCATCTGTTGATTCCTATCAT--------CATTCCCTTCAC-
ATCAGCGTCATGATTTTCAACCTACGTGTTGAAAATTA-TTTTCGCTCCTTCCACAC--TTTTT--
TCGCTGGTTACCCCGCCGCGAGGCACCCGCACGACCCCGCGGTGCAAACGAAAAATTTCTTAT
CACAGCCCCACCTTGCACAAGCAACCATGCATTGCTCATGAGACCCACTT--
TGAACAATTGCTAATGCCTTCATACAGGAAGCTGCCGAGCTCGGTAAGGGTTCCTTCAAGTACG
CCTGGGTTCTTGACAAGCTCAAGGCCGAGCGTGAGCGTGGTATCACCATCGATATCGCTCTCTG
GAAGTTCGAGACCAACGAGTACAATGTCACCGTCATTGGTTAGTACCCCTCCACCAATGCCATA
TGCTGCTCCATAAGACACTTGACTAACCTTACTTCATAGACGCTCCCGGTCACCGTGATTTCATC
AAGAACATGCTGCCTTCTGGTATGTAATCTGTCTGCCTCGACACGGCCTCAATACGACGTTTTTC
GTGCCTGCACGACGGCCCCGATCAG-
TGAATTAGGTCAAGATACAGGGAACATGACGCTAATAGGTCATTTATAGGCAAACCATCTCTGG
CGAGCACGGTCTCGACAGCAATGGAGTGTATGTACTATTTTCAATTCCTCCTGCTTCCTGTTGAG
CTTGTAGGCTGAC-
TCGATGGCCATTTAGCTACAACGGTACCTCCGAGCTCCAGCTCGAGCGTATGAGCGTCTACTTC
AACGAGGCTTCCGGCAACAAGTACGTTCCTCGTGCCGTCCTCGTCGATCTCGAGCCCGGTACCA
TGGATGCCGTCCGCGCCGGTCCCTTCGGCCAGCTCTTCCGCC???????????????????????????????

????????????
>CL5_1
??TTATAGAGTTTTCTAAACTCCCAACCCATGTGAACTTACCTTTTGTTGCCTCGGCAGAAGTTAT
AGGTCTTCTTATAGCTGCTGCCGGTGGACCATTAAACTCTTGTTATTTTATGTAATCTGAGCGTCT
TATTTTAATAAGTCAAAACTTTCAACAACGGATCTCTTGGTTCTGGCATCGATGAAGAACGCAGC
GAAATGCGATAAGTAATGTGAATTGCAGAATTCAGTGAATCATCGAATCTTTGAACGCACATTG
CGCCCATTAGTATTCTAGTGGGCATGCCTGTTCGAGCGTCATTTCAACCCTTAAGCCTAGCTTAG
TGTTGGGAATCTACTTCTCTTAGGAGTTGTAGTTCCTGAAATACAACGGCGGATTTGTAGTATCC
TCTGAGCGTAGTAA-
TTTTTTTCTCGCTTTTGTTAGGTGCTATAACTCCCAGCCGCTAAACCCCCAATTTTTTGTGGTTGAC
CTCGGATCAGGTAGAAGGTTAGTCATCTATTGATTCCCATCAT-------CATTTCCCTTCAC-
TTCAGTGTTATAATTTTCAACCTACGTGTTGAAAATTA-TTTTCGCTCCTTCCACAC--TTTTT--
TCGCTGGTTACCCCGCCGCGAGGCACCCGCACGACCCCGCGGTGCAAACGAAAAATTTCTTAT
CACAGCCCCACCTTGCACAAGCAATCATGCATTGCTCATGAGACCCACTT--
TGAACAATTGCTAATGCCTTCATACAGGAAGCCGCCGAGCTCGGTAAGGGTTCCTTCAAGTACG
CCTGGGTTCTTGACAAGCTCAAGGCCGAGCGTGAGCGTGGTATCACCATCGATATCGCTCTCTG
GAAGTTCGAGACCAACGAGTACAATGTCACCGTCATTGGTTAGTACCCCTCCACCTATGCCATG
TGCTGCTCCATAAGACACTTGACTAACCTTGCTTCATAGACGCTCCCGGTCACCGTGATTTCATC
AAGA???TGCTGCCTTCTGGTATGTTACCTGTCTGCCTCGACACGGCCTTACTACGACGTTTTTCG
TGCCTGCACGACGGCCCCGAACAG-
TGAAATAGGTCAAGATAGAGGGAACATGATACTAATAGGTCATTTATAGGCAAACCATCTCTGG
CGAGCACGGTCTCGACAGCAATGGAGTGTATGTACTAACTTCAATTCCTCCTGCTTCCTGTTGAG
TTTGTAGGCTGAC-
TCGATGGCCATTTAGCTACAACGGTACCTCCGAGCTCCAGCTCGAGCGTATGAGCGTCTACTTC
AACGAGGCTTCCGGCAACAAGTACGTTCCTCGTGCCGTCCTCGTCGATCTCGAGCCCGGTACCA
TGGATGCCGTCCGCGCCGGTCCCTTCGGCCAGCT???
??????????
>CL5_1_1
????????AGTTTTCTAAACTCCCAACCCATGTGAACTTACCTTTTGTTGCCTCGGCAGAAGTTATAG
GTCTTCTTATAGCTGCTGCCGGTGGACCATTAAACTCTTGTTATTTTATGTAATCTGAGCGTCTTA
TTTTAATAAGTCAAAACTTTCAACAACGGATCTCTTGGTTCTGGCATCGATGAAGAACGCAGCGA
AATGCGATAAGTAATGTGAATTGCAGAATTCAGTGAATCATCGAATCTTTGAACGCACATTGCG
CCCATTAGTATTCTAGTGGGCATGCCTGTTCGAGCGTCATTTCAACCCTTAAGCCTAGCTTAGTG
TTGGGAATCTACTTCTCTTAGGAGTTGTAGTTCCTGAAATACAACGGCGGATTTGTAGTATCCTC
TGAGCGTAGTAA-
TTTTTTTCTCGCTTTTGTTAGGTGCTATAACTCCCAGCCGCTAAACCCCCAATTTTTTGTGGTTGAC
CTCGGATCAGGTAGAAGGTTAGTCATCTATTGATTCCCATCAT-------CATTTCCCTTCAC-
TTCAGTGTTATAATTTTCAACCTACGTGTTGAAAATTA-TTTTCGCTCCTTCCACAC--TTTTT--
TCGCTGGTTACCCCGCCGCGAGGCACCCGCACGACCCCGCGGTGCAAACGAAAAATTTCTTAT
CACAGCCCCACCTTGCACAAGCAATCATGCATTGCTCATGAGACCCACTT--
TGAACAATTGCTAATGCCTTCATACAGGAAGCCGCCGAGCTCGGTAAGGGTTCCTTCAAGTACG
CCTGGGTTCTTGACAAGCTCAAGGCCGAGCGTGAGCGTGGTATCACCATCGATATCGCTCTCTG
GAAGTTCGAGACCAACGAGTACAATGTCACCGTCATTGGTTAGTACCCCTCCACCTATGCCATG
TGCTGCTCCATAAGACACTTGACTAACCTTGCTTCATAGACGCTCCCGGTCACCGTGATTTCATC

AAGAA??TGCTGCCTTCTGGTATGTTACCTGTCTGCCTCGACACGGCCTTACTACGACGTTTTTCG
TGCCTGCACGACGGCCCCGAACAG-
TGAAATAGGTCAAGATAGAGGGAACATGATACTAATAGGTCATTTATAGGCAAACCATCTCTGG
CGAGCACGGTCTCGACAGCAATGGAGTGTATGTACTAACTTCAATTCCTCCTGCTTCCTGTTGAG
TTTGTAGGCTGAC-
TCGATGGCCATTTAGCTACAACGGTACCTCCGAGCTCCAGCTCGAGCGTATGAGCGTCTACTTC
AACGAGGCTTCCGGCAACAAGTACGTTCCTCGTGCCGTCCTCGTCGATCTCGAGCCCGGTACCA
TGGATGCCGTCCGCGCCGGTCCCTTCGGCCAGCT??
??????????
>CR20
????ATAGAGTTTTCTAAACTCCCAACCCATGTGAACTTACCTTTTGTTGCCTCGGCAGAAGTTATA
GGTCTTCTTATAGCTGCTGCCGGTGGACCATTAAACTCTTGTTATTTTATGTAATCTGAGCGTCTT
ATTTTAATAAGTCAAAACTTTCAACAACGGATCTCTTGGTTCTGGCATCGATGAAGAACGCAGCG
AAATGCGATAAGTAATGTGAATTGCAGAATTCAGTGAATCATCGAATCTTTGAACGCACATTGC
GCCCATTAGTATTCTAGTGGGCATGCCTGTTCGAGCGTCATTTCAACCCTTAAGCCTAGCTTAGT
GTTGGGAATCTACTTCTTTA--
TAGTTGTAGTTCCTGAAATACAACGGCGGATTTGTAGTATCCTCTGAGCGTAGTAA-
TTTTTTTCTCGCTTTTGTTAGGTGCTATAACTCCCAGCCGCTAAACCCCCAATTTTTTGTGGTTGAC
CTCGGATCAGGTAGAAGGTTAGTCATTTATTGATTCCCATCAT-------CATCCCCCTTCAC-
TTCAGCATCATAATTTTCAACCTACATGTTGAAAATTA-TTTTCGCTCCTTCCACAC--TTTTT--
TCGCTGGTTACCCCGCCGCGAGGCACCCGCACGACCCCGCGGTGCAAACGAAAAATTTCTTAT
CACAGCCCCACCTTGCACAAGCAACCATGCATTGCTCATGAGACCCACTTTGTGAACAATTGCT
AATGCCTTCATACAGGAAGCCGCCGAGCTCGGTAAGGGTTCCTTCAAGTACGCCTGGGTTCTTG
ACAAGCTCAAGGCCGAGCGTGAGCGTGGTATCACCATCGATATCGCTCTCTGGAAGTTCGAGA
CCAACGAGTACAATGTCACCGTCATTGGTTAGTACCACTCCACCTATGCCATGTGCTGCTCCATA
AGACACTTGACTAACCCTGCTTCACAGACGCTCCCGGTCACCGTGATTTCATCAAGAACATGCT
GCCTTCTGGTATGTAATCTGTCTGCCTCGACACGGCCTTAATACGACGTTTTTCGTGCCTGCACG
ACGGCCCCGATCAG-
TGAATTAGGTCAAGATACAGGGAACATGATGCTAATAGGTCATTTATAGGCAAACCATCTCTGG
CGAGCACGGTCTCGACAGCAATGGAGTGTATGTACTATTTTCAATTCCTCCTGCTTCCTGTTGAG
CTTGTAGGCTGAC-
TCGATGGCCATTTAGCTACAACGGTACCTCCGAGCTCCAGCTCGAGCGTATGAGCGTCTACTTC
AACGAGGCTTCCGGCAACAAGTACGTTCCTCGTGCCGTCCTCGTCGATCTCGAGCCCGGTACCA
TGGATGCCGTCCGCGCCGGTCCCTTCGGCCAGCT??
??????????
>E2
CATTATAGAGTTTTCTAAACTCCCAACCCATGTGAACTTACCTTTTGTTGCCTCGGCAGAAGTTAT
AGGTCTTCTTATAGCTGCTGCCGGTGGACCATTAAACTCTTGTTATTTTATGTAATCTGAGCGTCT
TATTTTAATAAGTCAAAACTTTCAACAACGGATCTCTTGGTTCTGGCATCGATGAAGAACGCAGC
GAAATGCGATAAGTAATGTGAATTGCAGAATTCAGTGAATCATCGAATCTTTGAACGCACATTG
CGCCCATTAGTATTCTAGTGGGCATGCCTGTTCGAGCGTCATTTCAACCCTTAAGCCTAGCTTAG
TGTTGGGAATCTACTTCTCCTAGGAGTTGTAGTTCCTGAAATACAACGGCGGATTTGTAGTATCC
TCTGAGCGTAGTAA-
TTTTTTTCTCGCTTTTGTTAGGTGCTATAACTCCCAGCCGCTAAACCCCCAATTTTTTGTGGTTGAC

CTCGGATCAGGT???????????????????GATTCCCATCAT-------CATCCCCCTTCAC-
TTCAGCATCATAATTTTCAACCTACATGTTGAAAATTA-TTTTCGCTCCTTCCACAC--TTTTT--
TCGCTGGTTACCCCGCCGCGAGGCACCCGCACGACCCCGCGGTGCAAACGAAAAATTTCTTAT
CACAGCCCCACCTTGCACAAGCAACCATGCATTGCTCATGAGACCCACTTTGTGAACAATTGCT
AATGCCTTCATACAGGAAGCCGCCGAGCTCGGTAAGGGTTCCTTCAAGTACGCCTGGGTTCTTG
ACAAGCTCAAGGCCGAGCGTGAGCGTGGTATCACCATCGATATCGCTCTCTGGAAGTTCGAGA
CCAACGAGTACAATGTCACCGTCATTGGTTAGTACCACTCCACCTATGCCATGTGCTGCTCCATA
AGACACTTGACTAACCCTGCTTCACAGACGCTCCCGGTCACCGTGATTTCATCAAGAACATGCT
GCCTTCTGGTATGTAATCTGTCTGCCTCGACACGGCCTTGATACGACGTTTTTCGTGCCTGCACG
ACGGCCCCGATCAG-
TGAATTAGGTCAAGATACAGGGAACATGATGCTAATAGGTCATTTATAGGCAAACCATCTCTGG
CGAGCACGGTCTCGACAGCAATGGAGTGTATGTACTATTTTCAATTCCTCCTGCTTCCTGTTGAG
CTTGTAGGCTGAC-
TCGATGGCCATTTAGCTACAACGGTACCTCCGAGCTCCAGCTCGAGCGTATGAGCGTCTACTTC
AACGAGGCTTCCGGCAACAAGTACGTTCCTCGTGCCGTCCTCGTCGATCTCGAGCCCGGTACCA
TGGATGCCGTCCGCGCCGGTCCCTTCGGCCAGCTC??
???????????
>JK15_2
?????TAGAGTTTTCTAAACTCCCAACCCATGTGAACTTACCTTTTGTTGCCTCGGCAGAAGTTATA
GGTCTTCTTATAGCTGCTGCCGGTGGACCATTAAACTCTTGTTATTTTATGTAATCTGAGCGTCTT
ATTTTAATAAGTCAAAACTTTCAACAACGGATCTCTTGGTTCTGGCATCGATGAAGAACGCAGCG
AAATGCGATAAGTAATGTGAATTGCAGAATTCAGTGAATCATCGAATCTTTGAACGCACATTGC
GCCCATTAGTATTCTAGTGGGCATGCCTGTTCGAGCGTCATTTCAACCCTTAAGCCTAGCTTAGT
GTTGGGAATCTACTTCTCTTAGGAGTTGTAGTTCCTGAAATACAACGGCGGATTTGTAGTATCCT
CTGAGCGTAGTAA-
TTTTTTTCTCGCTTTTGTTAGGTGCTATAACTCCCAGCCGCTAAACCCCCAATTTTTTGTGGTTGAC
CTCGGATCAGG?AGAAGGTTAGTCATCTGTTGATTCCTATCAT--------CATTCCCTTCAC-
ATCAGCGTCATGATTTTCAACCTACGTGTTGAAAATTA-TTTTCGCTCCTTCCACAC--TTTTT--
TCGCTGGTTACCCCGCCGCGAGGCACCCGCACGACCCCGCGGTGCAAACGAAAAATTTCTTAT
CACAGCCCCACCTTGCACAAGCAACCATGCATTGCTCATGAGACCCACTT--
TGAACAATTGCTAATGCCTTCATACAGGAAGCTGCCGAGCTCGGTAAGGGTTCCTTCAAGTACG
CCTGGGTTCTTGACAAGCTCAAGGCCGAGCGTGAGCGTGGTATCACCATCGATATCGCTCTCTG
GAAGTTCGAGACCAACGAGTACAATGTCACCGTCATTGGTTAGTACCCCTCCACCAATGCCATA
TGCTGCTCCATAAGACACTTGACTAACCTTACTTCATAGACGCTCCCGGTCACCGTGATTTCATC
AAGAACATGCTGCCTTCTGGTATGTAATCTGTCTGCCTCGACACGGCCTCAATACGACGTTTTTC
GTGCCTGCACGACGGCCCCGATCAG-
TGAATTAGGTCAAGATACAGGGAACATGACGCTAATAGGTCATTTATAGGCAAACCATCTCTGG
CGAGCACGGTCTCGACAGCAATGGAGTGTATGTACTATTTTCAATTCCTCCTGCTTCCTGTTGAG
CTTGTAGGCTGAC-
TCGATGGCCATTTAGCTACAACGGTACCTCCGAGCTCCAGCTCGAGCGTATGAGCGTCTACTTC
AACGAGGCTTCCGGCAACAAGTACGTTCCTCGTGCCGTCCTCGTCGATCTCGAGCCCGGTACCA
TGGATGCCGTCCGCGCCGGTCCCTTCGGCCAGCTC??
???????????
>L8

??TTATAGAGTTTTCTAAACTCCCAACCCATGTGAACTTACCTTTTGTTGCCTCGGCAGAAGTTAT
AGGATTTCTTATAGCCGCTGCCGGTGGACCATCAAACTCTTGTTATTTTATGTAATCTGAGCGTCT
TATTTTAATAAGTCAAAACTTTCAACAACGGATCTCTTGGTTCTGGCATCGATGAAGAACGCAGC
GAAATGCGATAAGTAATGTGAATTGCAGAATTCAGTGAATCATCGAATCTTTGAACGCACATTG
CGCCCATTAGTATTCTAGTGGGCATGCCTGTTCGAGCGTCATTTCAACCCTTAAGCCCAGCTTAG
TGTTGGGAATCTACTTCTTCA--
CAGTCGTAGTTCCTGAAATACAACGGCGGATTGATAGCATCCTCTGAGCGTAGTAA-
TTTTTTTCTCGCTTTTGTCAGGTGCTGTGACTCCCAGCCGCTAAACCCCCAATTTTTTGTGGTTGA
CCTCGGATCAGGT????????????????????????????CATCAC--------------------------------
AAATTTCAACCTACGTGTTGAAAATTA-
TTTTCGCTCCTTCCACACTTTTTTTCCCCGCTGGCTACCCCGCCGCGAGGCACCCGCACGACCCC
GCGGTGCAAACGAAAAATTTCTTATCATAGCCCCACCTTGCATAAGCAACCATGCATTACTCAT
GAGATCCACTT--
TGAATAATTGCTAATGCCTTCATACAGGAAGCCGCCGAGCTCGGTAAGGGTTCCTTCAAGTACG
CCTGGGTTCTTGACAAGCTCAAGGCCGAGCGTGAGCGTGGTATCACCATCGATATCGCTCTCTG
GAAGTTCGAGACCAACGAGTACAATGTCACCGTCATTGGTTAGTACCTCTCCACCTATGCCATG
TGCTGCACCATAAGGCACTTCACTAACCT------------
GCTCAC????????????????????????????TGCTGCCTTCTGGTATGTAACCTGTCTGCCTCGACACGGCC
TTGATACGACGTTTTTCGTGCCTGCACGACGGCCTCGAACAG-
TGAACTAGGTCAAGACAGAGGGAACATAATGCTAATAGGTCATTTATAGGCAAACCATCTCTGG
CGAGCACGGTCTCGACAGCAATGGAGTGTATGTACCATTTTCAATT-------
CTTCCTGTTGAGCTTGTAGGCTGAC-
TCGATGGCCATTTAGCTACAACGGTACCTCCGAGCTCCAGCTCGAGCGTATGAGCGTCTACTTC
AACGAGGCTTCCGGCAACAAGTACGTTCCTCGTGCCGTCCTCGTCGATCTCGAGCCCGGTACCA
TGGATGCCGTCCGCGCCGGTCCCTTCGGCCAGCTCTTCCGCCCTGACAACTTCGTCTTCGGTCA
GTCCGGTGCTGGCAACAACTGG
>L8_1
??????????????CTAAACTCCCAACCCATGTGAACTTACCTTTTGTTGCCTCGGCAGAAGTTATAGG
ATTTCTTATAGCCGCTGCCGGTGGACCATCAAACTCTTGTTATTTTATGTAATCTGAGCGTCTTAT
TTTAATAAGTCAAAACTTTCAACAACGGATCTCTTGGTTCTGGCATCGATGAAGAACGCAGCGA
AATGCGATAAGTAATGTGAATTGCAGAATTCAGTGAATCATCGAATCTTTGAACGCACATTGCG
CCCATTAGTATTCTAGTGGGCATGCCTGTTCGAGCGTCATTTCAACCCTTAAGCCCAGCTTAGTG
TTGGGAATCTACTTCTTCA--
CAGTCGTAGTTCCTGAAATACAACGGCGGATTGATAGCATCCTCTGAGCGTAGTAA-
TTTTTTTCTCGCTTTTGTCAGGTGCTGTGACTCCCAGCCGCTAAACCCCCAATTTTTTGTGGTTGA
CCTCGGATCAGGT??
????????????????????????????CC-----------------
CCGCTGGCTACCCCGCCGCGAGGCACCCGCACGACCCCGCGGTGCAAACGAAAAATTTCTTAT
CATAGCCCCACCTTGCATAAGCAACCATGCATTACTCATGAGATCCACTT--
TGAATAATTGCTAATGCCTTCATACAGGAAGCCGCCGAGCTCGGTAAGGGTTCCTTCAAGTACG
CCTGGGTTCTTGACAAGCTCAAGGCCGAGCGTGAGCGTGGTATCACCATCGATATCGCTCTCTG
GAAGTTCGAGACCAACGAGTACAATGTCACCGTCATTGGTTAGTACCTCTCCACCTATGCCATG
TGCTGCACCATAAGGCAC??TGCTGCC
TTCTGGTATGTAACCTGTCTGCCTCGACACGGCCTTGATACGACGTTTTTCGTGCCTGCACGACG

GCCTCGAACAG-
TGAACTAGGTCAAGACAGAGGGAACATAATGCTAATAGGTCATTTATAGGCAAACCATCTCTGG
CGAGCACGGTCTCGACAGCAATGGAGTGTATGTACCATTTTCAATT-------
CTTCCTGTTGAGCTTGTAGGCTGAC-
TCGATGGCCATTTAGCTACAACGGTACCTCCGAGCTCCAGCTCGAGCGTATGAGCGTCTACTTC
AACGAGGCTTCCGGCAACAAGTACGTTCCTCGTGCCGTCCTCGTCGATCTCGAGCCCGGTACCA
TGGATGCCGTCCGCGCCGGTCCCTTCGGCCAGCTCTTCCGCCCTGACAACTTCGTCTTCGGTCA
GTCCGGTGCTGGCAACAACTGG
>HN89_1
CATTATAGAGTTTTCTAAACTCCCAACCCATGTGAACTTACCTTTTGTTGCCTCGGCAGAAGTTAT
AGGATTTCTTATAGCCGCTGCCGGTGGACCATCAAACTCTTGTTATTTTATGTAATCTGAGCGTCT
TATTTTAATAAGTCAAAACTTTCAACAACGGATCTCTTGGTTCTGGCATCGATGAAGAACGCAGC
GAAATGCGATAAGTAATGTGAATTGCAGAATTCAGTGAATCATCGAATCTTTGAACGCACATTG
CGCCCATTAGTATTCTAGTGGGCATGCCTGTTCGAGCGTCATTTCAACCCTTAAGCCTAGCTTAG
TGTTGGGAATCTACTTCTTCT--
CAGTCGTAGTTCCTGAAATACAACGGCGGATTTATAGCATCCTCTGAGCGTAGTAA-
TTTTTTTCTCGCTTTTGTCAGGTGCTGTGACTCCCAGCCGCTAAACCCCCAATTTTTTGTGG-
TGACCTC-GATCAG-T?????????????????????????CATCAT-------CATTACCCTCC-
CCTTCAGCATCACAAATTTCAACCTACGTGTTGAAAAATA-TTTTCGCTCCTTCCCCTT--TTTTT-
TCCCGTGGCTACCCCGCCGCGAGGC-
CCCGCACGACCCCGCGGTGCAAACGAAAAATTTCTTATCATAGCCCCACCTTGCATAAGCAACC
ATGCATTACTCATGAGATCCACTT--
TGAATAATTGCTAATGCCTTCATACAGGAAGCCGCCGAGCTCGGTAAGGGTTCCTTCAAGTACG
CCTGGGTTCTTGACAAGCTCAAGGCCGAGCGTGAGCGTGGTATCACCATCGATATCGCTCTC-
GGAAGTTCGAGACCAACGAGTACAATGTCACCGTC-
TTGGTTAGTACCTCTCCACCTATGCCATGTGCTGCACCACAAGGCACTTCACTAACCTTGCT-
CACAGATGCTACC-------------
TCCCCAA?????TGCTGCCTTCTGGTATGTAACCTGTCTGCCTCGACACGGCCTTAATACGACGTTT
TTCGTGCCTGCACGACGGCCTCGAACAG-
TGAACTAGGTCAAGACAGAGGGAACATAATACTAATAGGTCATTTATAGGCAAACCATCTCTGG
CGAGCACGGTCTCGACAGCAATGGAGTGTATGTACCATTTTCAATT-------
CTTCCTGTTGAGCTTGTAGGCTGAC-
TCGATGGCCATTTAGCTACAACGGTACCTCCGAGCTCCAGCTCGAGCGTATGAGCGTCTACTTC
AACGAGGCTTCCGGCAACAAGTACGTTCCTCGTGCCGTCCTCGTCGATCTCGAGCCCGGTACCA
TGGATGCCGTCCGCGCCGGTCCCTTCGGCCAGCTCTTCCGCCCTGACAACTTCGTC-
TCGGTCAGTCC-GTGC--GCAACAA-TGG
>LD1
????????AGTTTTCTAAACTCCCAACCCATGTGAACTTACCTTTTGTTGCCTCGGCAGAAGTTATAG
GTCTTCTTATAGCTGCTGCCGGTGGACCATTAAACTCTTGTTATTTTATGTAATCTGAGCGTCTTA
TTTTAATAAGTCAAAACTTTCAACAACGGATCTCTTGGTTCTGGCATCGATGAAGAACGCAGCGA
AATGCGATAAGTAATGTGAATTGCAGAATTCAGTGAATCATCGAATCTTTGAACGCACATTGCG
CCCATTAGTATTCTAGTGGGCATGCCTGTTCGAGCGTCATTTCAACCCTTAAGCCTAGCTTAGTG
TTGGGAATCTACTTCTCTTAGGAGTTGTAGTTCCTGAAATACAACGGCGGATTTGTAGTATCCTC
TGAGCGTAGTAA-

TTTTTTTCTCGCTTTTGTTAGGTGCTATAACTCCCAGCCGCTAAACCCCCAATTTTTTGTGGTTGAC
CTCGGATCAGGT???????????????????GATTCCCATCAT-------CATCCCCCTTCAC-
TTCAGCATCATAATTTTCAACCTACATGTTGAAAATTA-TTTTCGCTCCTTCCACAC--TTTTT--
TCGCTGGTTACCCCGCCGCGAGGCACCCGCACGACCCCGCGGTGCAAACGAAAAATTTCTTAT
CACAGCCCCACCTTGCACAAGCAACCATGCATTGCTCATGAGACCCACTTTGTGAACAATTGCT
AATGCCTTCATACAGGAAGCCGCCGAGCTCGGTAAGGGTTCCTTCAAGTACGCCTGGGTTCTTG
ACAAGCTCAAGGCCGAGCGTGAGCGTGGTATCACCATCGATATCGCTCTCTGGAAGTTCGAGA
CCAACGAGTACAATGTCACCGTCATTGGTTAGTACCACTCCACCTATGCCATGTGCTGCTCCATA
AGACACTTGACTAACCCTGCTTCACAGA????????????????????????????????TGCTGCCTTCTGGT
ATGTTACCTGTCTGCCTCGACACGGCCTTACTACGACGTTTTTCGTGCCTGCACGACGGCCCCG
AACAG-
TGAAATAGGTCAAGATAGAGGGAACATGATACTAATAGGTCATTTATAGGCAAACCATCTCTGG
CGAGCACGGTCTCGACAGCAATGGAGTGTATGTACTAACTTCAATTCCTCCTGCTTCCTGTTGAG
TTTGTAGGCTGAC-
TCGATGGCCATTTAGCTACAACGGTACCTCCGAGCTCCAGCTCGAGCGTATGAGCGTCTACTTC
AACGAGGCTTCCGGCAACAAGTACGTTCCTCGTGCCGTCCTCGTCGATCTCGAGCCCGGTACCA
TGGATGCCGTCCGCGCCGGTCCCTTCGGCCAGCTCTTCCGCCCTGACAACTTCGTCTTCGGTCA
GTCCGGTGCTGGCAACAACTGG
>ML3
??????????????????????CCAACCCATGTGAACTTACCTTTTGTTGCCTCGGCAGAAGTTATAGGTC
TTCTTATAGCTGCTGCCGGTGGACCATTAAACTCTTGTTATTTTATGTAATCTGAGCGTCTTATTTT
AATAAGTCAAAACTTTCAACAACGGATCTCTTGGTTCTGGCATCGATGAAGAACGCAGCGAAAT
GCGATAAGTAATGTGAATTGCAGAATTCAGTGAATCATCGAATCTTTGAACGCACATTGCGCCC
ATTAGTATTCTAGTGGGCATGCCTGTTCGAGCGTCATTTCAACCCTTAAGCCTAGCTTAGTGTTG
GGAATCTACTTCTCTTAGGAGTTGTAGTTCCTGAAATACAACGGCGGATTTGTAGTATCCTCTGA
GCGTAGTAA-
TTTTTTTCTCGCTTTTGTTAGGTGCTATAACTCCCAGCCGCTAAACCCCCAATTTTTTGTGGTTGAC
CTCGGATCAGGT??????????????????????????ATCAT-------CATCCCCCTTGAC-
TTCAGCATCATAATTTTCAACCTACGTGTTGAAAATTA-TTTTCGCTCCTTCCACAC--TTTTT--
TCGCTGGTTACCCCGCCGCGAGGCACCCGCACGACCCCGCGGTGCAAACGAAAAATTTCTTAT
CACAGCCCCACCTTGCACAAGCAACCATGCATTGCTCATGAGACCCACTT--
TGAACAATTGCTAATGCCTTCATACAGGAAGCCGCCGAGCTCGGTAAGGGTTCCTTCAAGTACG
CCTGGGTTCTTGACAAGCTCAAGGCCGAGCGTGAGCGTGGTATCACCATCGATATCGCTCTCTG
GAAGTTCGAGACCAACGAGTACAATGTCACCGTCATTGGTTAGTACCCCTCCGCCTATGCCATG
TGCTGCTCCATAAGACACTTGACTAACCTTGCTTCATAGACGCTCCCGGTCA??????????????????
??TGCTGCCTTCTGGTATGTAATCTGTCTGCCTCGACACGGCCTTAATACGACGTTTTTCGTGCCT
GCACGACGGCCCCGATCAG-
TGAATTAGGTCAAGATACAGGGAACATGATGCTAATAGGTCATTTATAGGCAAACCATCTCTGG
CGAGCACGGTCTCGACAGCAATGGAGTGTATGTACTATTTTCAATTCCTCCTGCTTCCTGTTGAG
CTTGTAGGCTGAC-
TCGATGGCCATTTAGCTACAACGGTACCTCCGAGCTCCAGCTCGAGCGTATGAGCGTCTACTTC
AACGAGGCTTCCGGCAACAAGTACGTTCCTCGTGCCGTCCTCGTCGATCTCGAGCCCGGTACC?
???
>N_alpapicalis_MFLUCC_17_2544

CATTATAGAGTTTTCTAAACTCCCAACCCATGTGAACTTACC-
TTTGTTGCCTCGGCAGAAGTTATAGGTCTTCTTATAGCTGCTGCCGGTGGACCATTAAACTCTTG
TTATTTTATGTAATCTGAGCGTCTTATTTTAATAAGTCAAAACTTTCAACAACGGATCTCTTGGTTC
TGGCATCGATGAAGAACGCAGCGAAATGCGATAAGTAATGTGAATTGCAGAATTCAGTGAATC
ATCGAATCTTTGAACGCACATTGCGCCCATTAGTATTCTAGTGGGCATGCCTGTTCGAGCGTCAT
TTCAACCCTTAAGCCTAGCTTAGTGTTGGGAATCTACTTCTTTA--
TAGTTGTAGTTCCTGAAATACAACGGCGGATTTGTAGTATCCTCTGAGCGTAGTAA-
TTTTTTTCTCGCTTTTGTTAGGTGCTATAACTCCCAGCCGCTAAACCCCCAATTTTTTGTGGTTGAC
CTCGGATCAGGT??????????????????????????CATCAT----------CCCCCTTCAC-TTCAGCAT--
-AATTTTCAACCTACGTGTTGAAAATTA-TTTTCGCTCCTTCCACAC--TTTTT--
TCGCTGGTTACCCCGCCGCGAGGCACCCGCACGACCCCGCGGTGCAAACGAAAAATTTCTTAT
CACAGCCCCACCTTGCACAAGCAACCATGCATTGCTCATGAGACCCACTT--
TGAACAATTGCTAATGCCTTCATACAGGAAGCCGCCGAGCTCGGTAAGGGTTCCTTCAAGTACG
CCTGGGTTCTTGACAAGCTCAAGGCCGAGCGTGAGCGTGGTATCACCATCGATATCGCTCTCTG
GAAGTTCGAGACCAACGAGTACAATGTCACCGTCATTGGTTAGTACTCCTCCACCTATGCCATG
TGCTGCACCATGAGACACTTGACTAACCTTGCTTCATAGACGCTCCCGGTCACCGTGATTTCATC
AAGAACATGCTGCTTTCTGGTATGTAATCTGTCTGCCTCGACACGGCCTTAATACGACGTTTTTC
GTGCCTGCACGACGGCCCCGATCAG-
TGAATTAGGTCAAGATACAGGGAACATGATGCTAATAGGTCATTTATAGGCAAACCATCTCTGG
CGAGCACGGTCTCGACAGCAATGGAGTGTATGTACTATTTTCAATTCCTCCTGCTTCCTGTTGAG
CTTGTAGGCTGAC-
TCGATGGCCATTTAGCTACAACGGTACCTCCGAGCTCCAGCTCGAGCGTATGAGCGTCTACTTC
AACGAGGCTTCCGGCAACAAGTACGTTCCTCGTGCCGTCCTCGTCGATCTCGAGCCCGGTACCA
TGGATGCCGTCCGCGCCGGTCCCTTCGGCCAGCTCTTCCGCCCTGACAACTTCGTCTTCGGTCA
GTCCGGTGCT?????????????
>N_aotearoa_CBS_367_54
CATTATAGAGTTTTCTAAACTCCCAACCCATGTGAACTTACCTTTTGTTGCCTCGGCAGGAGTTAT
AGGTCTTCTTATAGCTGCTGCCGGTGGACCATTAAACTCTTGTTATTTTATGTAATCTGAGCGTCT
TATTTTAATAAGTCAAAACTTTCAACAACGGATCTCTTGGTTCTGGCATCGATGAAGAACGCAGC
GAAATGCGATAAGTAATGTGAATTGCAGAATTCAGTGAATCATCGAATCTTTGAACGCACATTG
CGCCCATTAGTATTCTAGTGGGCATGCCTGTTCGAGCGTCATTTCAACCCTTAAGCCTAGCTTAG
TGTTGGGAATCTACTTCTTTA--
TAGTTGTAGTTCCTGAAATACAACGGCGGATTTGTAGTATCCTCTGAGCGTAGTAA-
TTTTTTTCTCGCTTTTGTTAGGTGCTATAACTCCCAGCCGCTAAACCCCCAATTTTTTGTGGTTGAC
CTCGGATCAGGTAGAAGGTTAGTCATTTATTGATTCCCATCAT-------CATCCCCCTTCAC-
TTCAGCATCATAATTTTCAACCTACGTGTTGAAAATTA-TTTTCGCTCCTTCCACAC--TTTTT--
TCGCTGGTTACCCCGCCGCGAGGCACCCGCACGACCCCGCGGTGCAAACGAAAAATTTCTTAT
CACAGCCCCACCTTGCACAAGCAACCATGCATTGCTCATGAGACCCACTT--
TGAACAATTGCTAATGCCTTCATACAGGAAGCCGCCGAGCTCGGTAAGGGTTCCTTCAAGTACG
CCTGGGTTCTTGACAAGCTCAAGGCCGAGCGTGAGCGTGGTATCACCATCGATATCGCTCTCTG
GAAGTTCGAGACCAACGAGTACAATGTCACCGTCATTGGTTAGTACCCCTCCACCTATGCCATG
TGCTGCTCCATAAGACACTTGACTAACCTTGCTTCATAGACGCTCCCGGTCACCGTGATTTCATC
AAGAACATGCTGCCTTCTGGTATGTAATCTGTCTGCCTCGACACGGCCTTAATACGACGTTTTTC
GTGCCTGCACGACGGCCCCGATCAG-

TGAATTAGGTCAAGATACAGGGAACATGATGCTAATAGGTCATTTATAGGCAAACCATCTCTGG
CGAGCACGGTCTCGACAGCAATGGAGTGTATGTACTATTTTCAATTCCTCCTGCTTCCTGTTGAG
CTTGTAGGCTGAC-
TCGATGGCCATTTAGCTACAACGGTACCTCCGAGCTCCAGCTCGAGCGTATGAGCGTCTACTTC
AACGAGGCTTCCGGCAACAAGTACGTTCCTCGTGCCGTCCTCGTCGATCTCGAGCCCGGTACCA
TGGATGCCGTCCGCGCCGGTCCCTTCGGCCAGCTCTTCCGCCCTGACAACTTCGTCTTCGGTCA
GTCCGGTGCTGGCAACAACTGG
>N_asiatica_MFLUCC_12_0286
CATTATAGAGTTTTCTAAACTCCCAACCCATGTGAACTTACCTTTTGTTGCCTCGGCAGAAGTTAT
AGGTCTTCTTATAGCTGCTGCCGGTGGACCATTAAACTCTTGTTATTTTATGTAATCTGAGCGTCT
TATTTTAATAAGTCAAAACTTTCAACAACGGATCTCTTGGTTCTGGCATCGATGAAGAACGCAGC
GAAATGCGATAAGTAATGTGAATTGCAGAATTCAGTGAATCATCGAATCTTTGAACGCACATTG
CGCCCATTAGTATTCTAGTGGGCATGCCTGTTCGAGCGTCATTTCAACCCTTAAGCCTAGCTTAG
TGTTGGGAATCTACTTCTTTA--
TAGTTGTAGTTCCTGAAATACAACGGCGGATTTGTAGTATCCTCTGAGCGTAGTAA-
TTTTTTTCTCGCTTTTGTTAGGTGCTATAACTCCCAGCCGCTAAACCCCCAATTTTTTGTGGTTGAC
CTCGGATCAGGTAGAAGGTTAGTCATCTACTGATTCCCTTCAT-------
CATTCTCCTTCACACTTCATGCCATGATTTTCAACCTACGTGTTGAAAATTA-
TTTTCGCTCCTTCCACAC--TTTTT--
TCGCTGGTTACCCCGCCGCGAGGCACCCGCACGACCCCGCGGTGCAAACGAAAAATTTCTTAT
CACAGCCCCACCTTGCATAAGCAACCATGCATTGCTCATGGGATCCACTT--
TGAATAATCGCTAATGCCTTCATACAGGAAGCCGCCGAGCTCGGTAAGGGTTCCTTCAAGTACG
CCTGGGTTCTTGACAAGCTCAAGGCCGAGCGTGAGCGTGGTATCACCATCGATATCGCTCTCTG
GAAGTTCGAGACCAACGAGTACAATGTCACCGTCATTGGTTAGTACCCCTCCACCTATGCCATG
TGCTGCTCCATAAGACACTTGACTAACCTTGCTTTATAGACGCTCCCGGTCACCGTGATTTCATC
AAGAACATGCTGCTTTCTGGTATGTTACCTGTCTGCCTCGACACGGCCTTAATACGACGTTTTTC
GTGCCTGCACGACGGCCCCGAACAG-
TGAAATAGGTCAAGATAGAGGGAACATAATACTAATAGGTCATTTATAGGCAAACCATCTCTGG
CGAGCACGGTCTCGACAGCAATGGAGTGTATGTACTAACTTCAATTCCTCCTGCTTCCTGTTGAG
CTTGTAGGCTGAC-
TCGATGGCCATTTAGCTACAACGGTACCTCCGAGCTCCAGCTCGAGCGTATGAGCGTCTACTTC
AACGAGGCTTCCGGCAACAAGTACGTTCCTCGTGCCGTCCTCGTCGATCTCGAGCCCGGTACCA
TGGATGCCGTCCGCGCCGGTCCCTTCGGCCAGCTCTTCCGCCCTGACAACTTCGTCTTCGGTCA
GTCCGGTGCTGGCAACAACTGG
>N_australis_CBS_114159
CATTATAGAGTTTTCTAAACTCCCAACCCATGTGAACTTACCTTTTGTTGCCTCGGCAGAAGTTAT
AGGTCTTCTTATAGCTGCTGCCGGTGGACCATTAAACTCTTGTTATTTTATGTAATCTGAGCGTCT
TATTTTAATAAGTCAAAACTTTCAACAACGGATCTCTTGGTTCTGGCATCGATGAAGAACGCAGC
GAAATGCGATAAGTAATGTGAATTGCAGAATTCAGTGAATCATCGAATCTTTGAACGCACATTG
CGCCCATTAGTATTCTAGTGGGCATGCCTGTTCGAGCGTCATTTCAACCCTTAAGCCTAGCTTAG
TGTTGGGAATCTACTTCTTTTATTAGTTGTAGTTCCTGAAATACAACGGCGGATTTGTAGTATCCT
CTGAGCGTAGTAA-
TTTTTTTCTCGCTTTTGTTAGGTGCTACAACTCCCAGCCGCTAAACCCCCAATTTTTTGTGGTTGA
CCTCGGATCAGGT?GAAGGTTAGTCATCTACTGATTCCCGTCAT-------CATTCTCCTTCAC-

TTCAGCGTCATGATTTTCAACCTACGTGTTGAAAATTA-TTTTCGCTCCTTCCACAC--TTTTT--
TCGCTGGTTACCCCGCCGCGAGGCACCAGCACGACCCCGCGGTGCAAACGAAAAATTTCTTAT
CACAGCCCCACCTTGCATAAGCAACCATGCATTGCTCATGAGATCCACTT--
TGAACAATTGCTAATGCCTTCATACAGGAAGCCGCCGAGCTCGGTAAGGGTTCCTTCAAGTACG
CCTGGGTTCTTGACAAGCTCAAGGCCGAGCGTGAGCGTGGTATCACCATCGATATCGCTCTCTG
GAAGTTCGAGACCAACGAGTACAATGTCACCGTCATTGGTTAGTACCCCTCCACCTATGCCATG
TGCTGCTCCATAAGACACTTGACTAACCTTGCTTCATAGACGCTCCCGGTCACCGTGATTTCATC
AAGAACATGCTGCCTTCTGGTATGTAACCTGTCTGCCTCGACACGGCCTTAATACGACGTTTTTC
GTGCCTGCACGACGGCCCCGAACAG-
TGAATTAGGTCAAGATAGAGGGAACATGATGCTAATAGGTCATTGATAGGCAAACCATCTCTGG
CGAGCACGGTCTCGACAGCAATGGAGTGTATGTACTATTTTTAATTCCTCCTGCTTCCTGTTAAG
CTTGTAGGCTGAC-
TCGATGGCCATTTAGCTACAACGGTACCTCCGAGCTCCAGCTCGAGCGTATGAGCGTCTACTTC
AACGAGGCTTCCGGCAACAAGTACGTTCCTCGTGCCGTCCTCGTCGATCTCGAGCCCGGTACCA
TGGATGCCGTCCGCGCCGGTCCCTTCGGCCAGCTCTTCCGCCCTGACAACTTCGTCTTCGGTCA
GTCCGGTGCTGGCAACAACT??
>N_brachiata_MFLUCC_17_1555
CATTATAGAGTTTTCTAAACTCCCAACCCATGTGAACTTACCTTTTGTTGCCTCGGCAGAAGTTAT
AGGTCTTCTTATAGCTGCTGCCGGTGGACCATTAAACTCTTGTTATTTTATGTAATCTGAGCGTCT
TATTTTAATAAGTCAAAACTTTCAACAACGGATCTCTTGGTTCTGGCATCGATGAAGAACGCAGC
GAAATGCGATAAGTAATGTGAATTGCAGAATTCAGTGAATCATCGAATCTTTGAACGCACATTG
CGCCCATTAGTATTCTAGTGGGCATGCCTGTTCGAGCGTCATTTCAACCCTTAAGCCTAGCTTAG
TGTTGGGAATCTACTTCTTTA--
TAGTTGTAGTTCCTGAAATACAACGGCGGATTTGTAGTATCCTCTGAGCGTAGTAA-
TTTTTTTCTCGCTTTTGTTAGGTGCTATAACTCCCAGCCGCTAAACCCCCAATTTTTTGTGGTTGAC
CTCGGATCAGGT??????TTAGTCATTTATTGATTCCCATCAT-------CATCCCCCTTCAC-
TTCAGCATCATAAATTTCAACCTACGTGTTGAAAATTA-TTTTCGCTCCTTCCACAC--TTTTT--
TCGCTGGTTACCCCGCCGCGAGGCACCCGCACGACCCCGCGGTGCAAACGAAAAATTTCTTAT
CACAGCCCCACCTTGCACAAGCAACCATGCATTGCTCATGAGACCCACTT--
TGAACAATTGCTAATGCCTTCATACAGGAAGCCGCCGAGCTCGGTAAGGGTTCCTTCAAGTACG
CCTGGGTTCTTGACAAGCTCAAGGCCGAGCGTGAGCGTGGTATCACCATCGATATCGCTCTCTG
GAAGTTCGAGACCAACGAGTACAATGTCACCGTCATTGGTTAGTACCCCTCCACCTATGCCATG
TGCTGCTCCATAAGACACTTGACTAACCTTGCTTCATAGACGCTCCCGGTCACCGTGATTTCATC
AAG????TGCTGCCTTCTGGTATGTAATCTGTCTGCCTCGACACGGCCTTAATACGACGTTTTTCGT
GCCTGCACGACGGCCCCGATCAG-
TGAATTAGGTCAAGATACAGGGAACATGATGCTAATAGGTCATTTATAGGCAAACCATCTCTGG
CGAGCACGGTCTCGACAGCAATGGAGTGTATGTACTATTTTCAATTCCTCCTGCTTCCTGTTGAG
CTTGTAGGCTGAC-
TCGATGGCCATTTAGCTACAACGGTACCTCCGAGCTCCAGCTCGAGCGTATGAGCGTCTACTTC
AACGAGGCTTCCGGCAACAAGTACGTTCCTCGTGCCGTCCTCGTCGATCTCGAGCCCGGTACCA
TGGATGCCGTCCGCGCCGGTCCCTTCGGCCAGCTCTTCCGCCCTGACAACTTCGTCTTCGGTCA
GTCCGGTGCTGGCAACAACTGG
>N_brasiliensis_COAD_2166
CATTATAGAGTTTTCTAAACTCCCAACCCATGTGAACTTACCTTTTGTTGCCTCGGCAGAAGTTAT

AGGTCTTCTTATAGCTGCTGCCGGTGGACCATTAAACTCTTGTTATTTTATGTAATCTGAGCGTCT
TATTTTAATAAGTCAAAACTTTCAACAACGGATCTCTTGGTTCTGGCATCGATGAAGAACGCAGC
GAAATGCGATAAGTAATGTGAATTGCAGAATTCAGTGAATCATCGAATCTTTGAACGCACATTG
CGCCCATTAGTATTCTAGTGGGCATGCCTGTTCGAGCGTCATTTCAACCCTTAAGCCTAGCTTAG
TGTTGGGAATCTACTTCTCTTAGGAGTTGTAGTTCCTGAAATACAACGGCGGATTTGTAGTATCC
TCTGAGCGTAGTAA-
TTTTTTTCTCGCTTTTGTTAGGTGCTATAACTCCCAGCCGC???????????????????????????????????
???AGAAGGTTAGCCATCTACTGATTTCCGTCAT-------TATTCTCCTTCAC-
TTCAGCGTGATGATTTTCAACCTACGTGTTGAAAATTA-TTTTCGCTCCTTCCACAC--TTTTT--
TCGCTGGTTACCCCGCCGCGAGGCACCCGCACGACCCCGCGGTGCAAGCGAAAAATTTCTTAT
CACAGCCCCACCTTGCATAAGCAACCATGCATTGCTCATGAGATCCACTT--
TGAACAATTGCTAATGCTTTCATACAGGAAGCCGCCGAGCTCGGTAAGGGTTCCTTCAAGTACG
CCTGGGTTCTTGACAAGCTCAAGGCCGAGCGTGAGCGTGGTATCACCATCGATATCGCTCTCTG
GAAGTTCGAGACCAACGAGTACAATGTCACCGTCATTGGTTAGTACCCCTCCACCTATGTCATG
TGCTGCTCCATAAGACACTTGACTAACCTTGCTTCATAGACGCTCCCGGTCACCGTGATTTCATC
AAGAACATGCTGCCTTCTGGTATGTTACCTGTCTGCCTCGACACGGCCTTACTACGACGTTTTTC
GTGCCTGCACGACGGCCCCGAACAG-
TGAAATAGGTCAAGATAGAGGGAACATGATACTAATAGGTCATTTATAGGCAAACCATCTCTGG
CGAGCACGGTCTCGACAGCAATGGAGTGTATGTACTAACTTCAATTCCTCCTGCTTCCTGTTGAG
TTTGTAGGCTGAC-
TCGATGGCCATTTAGCTACAACGGTACCTCCGAGCTCCAGCTCGAGCGTATGAGCGTCTACTTC
AACGAGGCTTCCGGCAACAAGTACGTTCCTCGTGCCGTCCTCGTCGATCTCGAGCCCGGTACCA
TGGATGCCGTCCGCGCCGGTCCCTTCGGCCAGCTCTTCCGCCCTGACAACTTCGTCTTCGGTCA
GTCCGGTGCTGGCA????????
>N_camelliae_oleiferaeCSUFTCC81
??????????????????????CCCACCCATGTG-
ACTTACCTTTTGTTGCCTCGGCAGAAGTTATAGGTCTTCTTATAGCTGCTGCCGGTGGACCATTA
AACTCTTGTTATTTTATGTAATCTGAGCGTCTTATTTTAATAAGTCAAAACTTTCAACAACGGATC
TCTTGGTTCTGGCATCGATGAAGAACGCAGCGAAATGCGATAAGTAATGTGAATTGCAGAATTC
AGTGAATCATCGAATCTTTGAACGCACATTGCGCCCATTAGTATTCTAGTGGGCATGCCTGTTCG
AGCGTCATTTCAACCCTTAAGCCTAGCTTAGTGTTGGGAATCTACTTCTTTTATTAGTTGTAGTTC
CTGAAATACAACGGCGGATTTGTAGTATCCTCTGAGCGTAGTAA-
TTTTTTTCTCGCTTTTGTTAGGTGCTATAACTCCCAGCCGCTAAACCCCCAATTTTTTGTGGTTGAC
CTC??????????????????????????????TTCCCATTAT-------CATTTCCCTTCAC-
TGCAGCGTCATCATTTTCAATCTACGTGTTGAAAATTA-TTTTCGCTCCTTCCACAC--TTTTT--
TCGCTGGTTACCCCGCCGCGAGGCACCCGCACGACCCCGCGGTGCAAACGAAAAATTTCTTAT
CACAGCCCCACCTTGCACAAGCAACAATGCATTGCTCATGAGACCCACTT--
TGAACAATTGCTAATGCCTTCATATAGGAAGCCGCCGAGCTCGGTAAGGGTTCCTTCAAGTACG
CCTGGGTTCTTGACAAGCTCAAGGCCGAGCGTGAGCGTGGTATCACCATCGATATCGCTCTCTG
GAAGTTCGAGACCAACGAGTACAATGTCACCGTCATTGGTTAGTACCCCTCCACCTATGCC---
TGCCGCTCCATAAGGCACTTGACTAACTTTGCTTCATAGACGCTCCCGGTCACCGTGATTTCA???
??????TGCTGCCTTCTGGTATGTAACCTGTCTGCCTCGACACGGCCTTGATACGACGTTTTTCGTG
CCTGCACGACGGCCCCGAACAG-
TGAATTAGGTCAAGATAGAGGGAACATGATGCTAATAGGTCATTGATAGGCAAACCATCTCTGG

CGAGCACGGTCTCGACAGCAATGGAGTGTATGTACTATTTTCAATTCCTCCTGCTTCCTGTTGAG
CTTGTAGGCTGAC-
TCGATGGCCATTTAGCTACAACGGTACCTCCGAGCTCCAGCTCGAGCGTATGAGCGTCTACTTC
AACGAGGCTTCCGGTAACAAGTACGTTCCTCGTGCCGTCCTCGTCGATCTCGAGCCCGGTACCA
TGGATGCCGTCCGCGCCGGTCCCTTCGGCCAGCTCTTCCGCCCTGACAA?????????????????????
????????????????
>N_camelliae_oleiferaeCSUFTCC82
??????????????????????CCCACCCATGTG-
ACTTACCTTTTGTTGCCTCGGCAGAAGTTATAGGTCTTCTTATAGCTGCTGCCGGTGGACCATTA
AACTCTTGTTATTTTATGTAATCTGAGCGTCTTATTTTAATAAGTCAAAACTTTCAACAACGGATC
TCTTGGTTCTGGCATCGATGAAGAACGCAGCGAAATGCGATAAGTAATGTGAATTGCAGAATTC
AGTGAATCATCGAATCTTTGAACGCACATTGCGCCCATTAGTATTCTAGTGGGCATGCCTGTTCG
AGCGTCATTTCAACCCTTAAGCCTAGCTTAGTGTTGGGAATCTACTTCTTTTATTAGTTGTAGTTC
CTGAAATACAACGGCGGATTTGTAGTATCCTCTGAGCGTAGTAA-
TTTTTTTCTCGCTTTTGTTAGGTGCTATAACTCCCAGCCGCTAAACCCCCAATTTTTTGTGGTTGAC
CTC??????????????????????????????TTCCCATTAT-------CATTTCCCTTCAC-
TGCAGCGTCATCATTTTCAATCTACGTGTTGAAAATTA-TTTTCGCTCCTTCCACAC--TTTTT--
TCGCTGGTTACCCCGCCGCGAGGCACCCGCACGACCCCGCGGTGCAAACGAAAAATTTCTTAT
CACAGCCCCACCTTGCACAAGCAACAATGCATTGCTCATGAGACCCACTT--
TGAACAATTGCTAATGCCTTCATATAGGAAGCCGCCGAGCTCGGTAAGGGTTCCTTCAAGTACG
CCTGGGTTCTTGACAAGCTCAAGGCCGAGCGTGAGCGTGGTATCACCATCGATATCGCTCTCTG
GAAGTTCGAGACCAACGAGTACAATGTCACCGTCATTGGTTAGTACCCCTCCACCTATGCC---
TGCCGCTCCATAAGGCACTTGACTAACTTTGCTTCATAGACGCTCCCGGTCACCGTGATTTCA???
??????TGCTGCCTTCTGGTATGTAACCTGTCTGCCTCGACACGGCCTTGATACGACGTTTTTCGTG
CCTGCACGACGGCCCCGAACAG-
TGAATTAGGTCAAGATAGAGGGAACATGATGCTAATAGGTCATTGATAGGCAAACCATCTCTGG
CGAGCACGGTCTCGACAGCAATGGAGTGTATGTACTATTTTCAATTCCTCCTGCTTCCTGTTGAG
CTTGTAGGCTGAC-
TCGATGGCCATTTAGCTACAACGGTACCTCCGAGCTCCAGCTCGAGCGTATGAGCGTCTACTTC
AACGAGGCTTCCGGTAACAAGTACGTTCCTCGTGCCGTCCTCGTCGATCTCGAGCCCGGTACCA
TGGATGCCGTCCGCGCCGGTCCCTTCGGCCAGCTCTTCCGCCCTGACAA?????????????????????
????????????????
>N_cavernicola_KUMCC_20_0269
CATTATAGAGTTTTCTAAACTCCCAACCCATGTGAACTTACCTTTTGTTGCCTCGGCAGAAGTTAT
AGGTCTTCTTATAGCTGCTGCCGGTGGACCATTAAACTCTTGTTATTTTATGTAATCTGAGCGTCT
TATTTTAATAAGTCAAAACTTTCAACAACGGATCTCTTGGTTCTGGCATCGATGAAGAACGCAGC
GAAATGCGATAAGTAATGTGAATTGCAGAATTCAGTGAATCATCGAATCTTTGAACGCACATTG
CGCCCATTAGTATTCTAGTGGGCATGCCTGTTCGAGCGTCATTTCAACCCTTAAGCCTAGCTTAG
TGTTGGGAATCTACTTCTCTTAGGAGTTGTAGTTCCTGAAATACAACGGCGGATTTGTAGTATCC
TCTGAGCGTAGTAA-
TTTTTTTCTCGCTTTTGTTAGGTGCTATAACTCCCAGCCGCTAAACCCCCAATTTTTTGTGGTTGAC
CTCGGATCAGGT??TTTCA
ACCTACGTGTTGAAAATTA-TCTTCGCTCCTTCCACAC--TTTTT--
TCGCTGGTTACCCCGCCGCGAGGCACCCGCACGACCCCGCGGTGCAAACGAAAAATTTCTTAT

CACAGCCCCACCTTGCACAAACAATCATGCATTGCTCATGAGACCCACTT--
TGAACAATTGCTAATGCCTTCATACAGGAAGCCGCCGAGCTCGGTAAGGGTTCCTTCAAGTACG
CCTGGGTTCTTGACAAGCTCAAGGCCGAGCGTGAGCGTGGTATCACCATCGATATCGCTCTCTG
GAAGTTCGAGACCAACGAGTACAATGTCACCGTCATTGGTTAGTACCCCTCCACCTATGCCATG
TGCTGCACCATAAGACACTTGACTAACCTTGCTTCATAGACGCTCCCGGTCACCGTGATTTCATC
AAGAACATGCTGCTTTCTGGTATGTAATCTGTCTGCCTCGACACGGCCTTAATACGACGTTTTTC
GTGCCTGCACGACGGCCCCGATCAG-
TGAATTAGGTCAAGATACAGGGAACATGATGCTAATAGGTCATTTATAGGCAAACCATCTCTGG
CGAGCACGGTCTCGACAGCAATGGAGTGTATGTACTATTTTCAATTCCTCCTGCTTCCTGTTGAG
CTTGTAGGCTGAC-
TCGATGGCCATTTAGCTACAACGGTACCTCCGAGCTCCAGCTCGAGCGTATGAGCGTCTACTTC
AACGAGGCTTCCGGCAACAAGTACGTTCCTCGTGCCGTCCTCGTCGATCTCGAGCCCGGTACCA
TGGATGCCGTCCGCGCCGGTCCCTTCGGCCAGCTCTTCCGCCCTGACAACTTCGTCTTCGGTCA
GTCCGGTGCTGGCAACAACTGG
>N_cavernicola_KUMCC_20_0332
CATTATAGAGTTTTCTAAACTCCCAACCCATGTGAACTTACCTTTTGTTGCCTCGGCAGAAGTTAT
AGGTCTTCTTATAGCTGCTGCCGGTGGACCATTAAACTCTTGTTATTTTATGTAATCTGAGCGTCT
TATTTTAATAAGTCAAAACTTTCAACAACGGATCTCTTGGTTCTGGCATCGATGAAGAACGCAGC
GAAATGCGATAAGTAATGTGAATTGCAGAATTCAGTGAATCATCGAATCTTTGAACGCACATTG
CGCCCATTAGTATTCTAGTGGGCATGCCTGTTCGAGCGTCATTTCAACCCTTAAGCCTAGCTTAG
TGTTGGGAATCTACTTCTCTTAGGAGTTGTAGTTCCTGAAATACAACGGCGGATTTGTAGTATCC
TCTGAGCGTAGTAA-
TTTTTTTCTCGCTTTTGTTAGGTGCTATAACTCCCAGCCGCTAAACCCCCAATTTTTTGTGGTTGAC
CTCGGATCAGGT???TTTCA
ACCTACGTGTTGAAAATTA-TCTTCGCTCCTTCCACAC--TTTTT--
TCGCTGGTTACCCCGCCGCGAGGCACCCGCACGACCCCGCGGTGCAAACGAAAAATTTCTTAT
CACAGCCCCACCTTGCACAAACAATCATGCATTGCTCATGAGACCCACTT--
TGAACAATTGCTAATGCCTTCATACAGGAAGCCGCCGAGCTCGGTAAGGGTTCCTTCAAGTACG
CCTGGGTTCTTGACAAGCTCAAGGCCGAGCGTGAGCGTGGTATCACCATCGATATCGCTCTCTG
GAAGTTCGAGACCAACGAGTACAATGTCACCGTCATTGGTTAGTACCCCTCCACCTATGCCATG
TGCTGCACCATAAGACACTTGACTAACCTTGCTTCATAGACGCTCCCGGTCACCGTGATTTCATC
AAGAACATGCTGCTTTCT-
GTATGTAATCTGTCTGCCTCGACACGGCCTTAATACGACGTTTTTCGTGCCTGCACGACGGCCCC
GATCAG-
TGAATTAGGTCAAGATACAGGGAACATGATGCTAATAGGTCATTTATAGGCAAACCATCTCTGG
CGAGCACGGTCTCGACAGCAATGGAGTGTATGTACTATTTTCAATTCCTCCTGCTTCCTGTTGAG
CTTGTAGGCTGAC-
TCGATGGCCATTTAGCTACAACGGTACCTCCGAGCTCCAGCTCGAGCGTATGAGCGTCTACTTC
AACGAGGCTTCCGGCAACAAGTACGTTCCTCGTGCCGTCCTCGTCGATCTCGAGCCCGGTACCA
TGGATGCCGTCCGCGCCGGTCCCTTCGGCCAGCTCTTCCGCCCTGACAACTTCGTCTTCGGTCA
GTCCGGTGCTGGCAACAACTGG
>N_chiangmaiensis_MFLUCC_18_0113
???
???

??
??
??
???????????????????????????????????AGAAGGTTAGTCATTTATTGATTCCCATCAT-------
CATCCCCCTTCAC-TTCAGCATCATAATTTTCAACCTACGTGTTGAAAATTA-
TTTTCGCTCCTTCCACAC--TTTTT--
TCGCTGGTTACCCCGCCGCGAGGCACCCGCACGACCCCGCGGTGCAAACGAAAAATTTCTTAT
CACAGCCCCACCTTGCACAAGCAACCATGCATTGCTCATGAGACCCACTT--
CGAACAATTGCTAATGCCTTCATACAGGAAGCCGCCGAGCTCGGTAAGGGTTCCTTCAAGTACG
CCTGGGTTCTTGACAAGCTCAAGGCCGAGCGTGAGCGTGGTATCACCATCGATATCGCTCTCTG
GAAGTTCGAGACCAACGAGTACAATGTCACCGTCATTGGTTAGTACCCCTCCACCAATGCCATA
TGCTGCTCCATAAGACACTTGACTAACCTTACTTCATAGACGCTCCCGGTCACCGTGATTTCATC
AAGAACA???????????????????ACTCGTCTGTCTCGAC-
CGGCCTCAATACGACGTTTTTCGTGCCTGCACGACAGCCCCGAACAG-
TGAATTAGGTCAAGATAGAGGGAACATGATGCTAATAGGTCATTGATAGGCAAACCATCTCTGG
CGAGCACGGTCTCGACAGCAATGGAGTGTATGTACTATTTTCAATTCCTCCTGCTTCCTGTTGAG
CTTGTAGGCTGAC-
TCGATGGCCATTTAGCTACAACGGTACCTCCGAGCTCCAGCTCGAGCGTATGAGTGTCTACTTC
AACGAGGCTTCCGGCAACAAGTACGTTCCTCGTGCCGTCCTCGTCGATCTCGAGCCCGGTACCA
TGGATGCCGTCCGCGCCGGTCCCTTCGGCCAGCTCTTCCGCCCTGACAACTTCGTCTTCGGTCA
GTCCGGTGCTGGCAACAACTGG
>N_chrysea_MFLUCC_12_0261
CATTATAGAGTTTTCTAAACTCCCAACCCATGTGAACTTACCTTTTGTTGCCTCGGCAGAAGTTAT
AGGTCTTCTTATAGCTGCTGCCGGTGGACCATTAAACTCTTGTTATTTTATGTAATCTGAGCGTCT
TATTTTAATAAGTCAAAACTTTCAACAACGGATCTCTTGGTTCTGGCATCGATGAAGAACGCAGC
GAAATGCGATAAGTAATGTGAATTGCAGAATTCAGTGAATCATCGAATCTTTGAACGCACATTG
CGCCCATTAGTATTCTAGTGGGCATGCCTGTTCGAGCGTCATTTCAACCCTTAAGCCTAGCTTAG
TGTTGGGAATCTACTTCTTTA--
TAGTTGTAGTTCCTGAAATACAACGGCGGATTTGTAGTATCCTCTGAGCGTAGTAA-
TTTTTTTCTCGCTTTTGTTAGGTGCTATAACTCCCAGCCGCTAAACCCCCAATTTTTTGTGGTTGAC
CTCGGATCAGGTAGAAGGTTAGTCATCTACTGATTCCCGTCAT-------CATTCTCCTTCAC-
TTCAGCGTCATGATTTTCAACCTACGTGTTGAAAATTA-TTTTCGCTCCTTCCACAC--TTTTT--
TCGCTGGTTACCCCGCCGCGAGGCACCCGCACGACCCCGCGGTGCAAACGAAAAATTTCTTAT
CACAGCCCCACCTTGCATAAGCAACCATGCATTGCTCATGAGATCCACCT--
TGAACAATCGCTAATGCCTTCATACAGGAAGCCGCCGAGCTCGGTAAGGGTTCCTTCAAGTACG
CCTGGGTTCTTGACAAGCTCAAGGCCGAGCGTGAGCGTGGTATCACCATCGATATCGCTCTCTG
GAAGTTCGAGACCAACGAGTACAATGTCACTGTCATTGGTTAGTACCCCTCCACCTATGCCATG
TGCTGCTCCATAAGACACTTGACTAACCTTGCTTTATAGACGCTCCCGGTCACCGTGATTTCATC
AAGAACATGCTGCTTTCTGGTATGTAACCTGTCTGCCTCGACACGGCCTTGATACGACGTTTTTC
GTGCCTGCACGACGGCCTCGAACAG-
TGAAATAGGTCAAGATAGAGGGAACATAATACTAATAGGTCATTTATAGGCAAACCATCTCTGG
CGAGCACGGTCTCGACAGCAATGGAGTGTATGTACTAACTTCAATTCCTCCTGCTTCCTGTTGAG
CTTGTAGGCTGAC-
TCGATGGCCATTTAGCTACAACGGTACCTCCGAGCTCCAGCTCGAGCGTATGAGCGTCTACTTC

AACGAGGCTTCCGGCAACAAGTACGTTCCTCGTGCCGTCCTCGTCGATCTCGAGCCCGGTACCA
TGGATGCCGTCCGCGCCGGTCCCTTCGGCCAGCTCTTCCGCCCTGACAACTTCGTCTTCGGTCA
GTCCGGTGCTGGCAACAACTGG
>N_clavispora_MFLUCC_12_0281
CATTATAGAGTTTTCTAAACTCCCAACCCATGTGAACTTACCTTTTGTTGCCTCGGCAGAAGTTAT
AGGTCTTCTTATAGCTGCTGCCGGTGGACCATTAAACTCTTGTTATTTTATGTAATCTGAGCGTCT
TATTTTAATAAGTCAAAACTTTCAACAACGGATCTCTTGGTTCTGGCATCGATGAAGAACGCAGC
GAAATGCGATAAGTAATGTGAATTGCAGAATTCAGTGAATCATCGAATCTTTGAACGCACATTG
CGCCCATTAGTATTCTAGTGGGCATGCCTGTTCGAGCGTCATTTCAACCCTTAAGCCTAGCTTAG
TGTTGGGAATCTACTTCTCTTAGGAGTTGTAGTTCCTGAAATACAACGGCGGATTTGTAGTATCC
TCTGAGCGTAGTAA-
TTTTTTTCTCGCTTTTGTTAGGTGCTATAACTCCCAGCCGCTAAACCCCCAATTTTTTGTGGTTGAC
CTCGGATCAGGTAGAAGGTTAGTCATCTATTGATTCCCATCAT-------CATTTCCCTTCAC-
TTCAGTGTTATAATTTTCAACCTACGTGTTGAAAATTA-TTTTCGCTCCTTCCACAC--TTTTT--
TCGCTGGTTACCCCGCCGCGAGGCACCCGCACGACCCCGCGGTGCAAACGAAAAATTTCTTAT
CACAGCCCCACCTTGCACAAGCAATCATGCATTGCTCATGAGACCCACTT--
TGAACAATTGCTAATGCCTTCATACAGGAAGCCGCCGAGCTCGGTAAGGGTTCCTTCAAGTACG
CCTGGGTTCTTGACAAGCTCAAGGCCGAGCGTGAGCGTGGTATCACCATCGATATCGCTCTCTG
GAAGTTCGAGACCAACGAGTACAATGTCACCGTCATTGGTTAGTACCCCTCCACCTATGCCATG
TGCTGCTCCATAAGACACTTGACTAACCTTGCTTCATAGACGCTCCCGGTCACCGTGATTTCATC
AAGAACATGCTGCTTTCTGGTATGTAATCTGTCTGCCTCGACACGGCCTTAATACGACGTTTTTC
GTACCTGCACGACGGCCCCGATCAG-
TGAATTAGGTCAAGATACAGGGAACATGATGCTAATAGGTCATTTATAGGCAAACCATCTCTGG
CGAGCACGGTCTCGACAGCAATGGAGTGTATGTACTATTTTCAATTCCTCCTGCTTCCTGTTGAG
CTTGTAGGCTGAC-
TCGATGGCCATTTAGCTACAACGGTACCTCCGAGCTCCAGCTCGAGCGTATGAGCGTCTACTTC
AACGAGGCTTCCGGCAACAAGTACGTTCCTCGTGCCGTCCTCGTCGATCTCGAGCCCGGTACCA
TGGATGCCGTCCGCGCCGGTCCCTTCGGCCAGCTCTTCCGCCCTGACAACTTCGTC-
TCGGTCAGTCC-GTGCTGGCAACAACTGG
>N_cocoes_MFLUCC_15_0152
CATTATAGAGTTTTCTAAACTCCCAACCCATGTGAACTTACCTTTTGTTGCCTCGGCAGAAGTTAT
AGGTCTTCTTATAGCTGCTGCCGGTGGACCATTAAACTCTTGTTATTTTATGTAATCTGAGCGTCT
TATTTTAATAAGTCAAAACTTTCAACAACGGATCTCTTGGTTCTGGCATCGATGAAGAACGCAGC
GAAATGCGATAAGTAATGTGAATTGCAGAATTCAGTGAATCATCGAATCTTTGAACGCACATTG
CGCCCATTAGTATTCTAGTGGGCATGCCTGTTCGAGCGTCATTTCAACCCTTAAGCCTAGCTTAG
TGTTGGGAATCTACTTCTCTTAGGAGTTGTAGTTCCTGAAATACAACGGCGGATTTGTAGTATCC
TCTGAGCGTAGTAA-
TTTTTTTCTCGCTTTTGTTAGGTGCTATAACTCCCAGCCGCTAAACCCCCAATTTTTTGTGG-
TGACCTCGGATCAG-T????????????????????ATCATCATCAT-----------
TCCCTTCACTTCAGGCATCATGATTTTCAACCTACGTGTTG-AAATTA-
TTTTCGCTCCTTCCACAC--TTTTT--
TCGCTGGTTACCCCGCCGCGAGGCACCCGCACGACCCCGCGGTGCAAACGAAAAATTTCTTAT
CACAGCCCCACCTTGCACAAGCAACCATGCATTGCTCATGAGACCCACTT--
TGAAAAATTGCGAATGCCTTCTTACAGGAAGCCGCCGAGATCGGTAAGGGTTCCTTCAAG?????

??
??
??
??
??
??
??
??????????
>N_coffea_arabicae_HGUP4015
CATTATAGAGTTTTCTAAACTCCCAACCCATGTGAACTTACCTTTTGTTGCCTCGGCAGAAGTTAT
AGGTCTTCTTATAGCTGCTGCCGGTGGACCATTAAACTCTTGTTATTTTATGTAATCTGAGCGTCT
TATTTTAATAAGTCAAAACTTTCAACAACGGATCTCTTGGTTCTGGCATCGATGAAGAACGCAGC
GAAATGCGATAAGTAATGTGAATTGCAGAATTCAGTGAATCATCGAATCTTTGAACGCACATTG
CGCCCATTAGTATTCTAGTGGGCATGCCTGTTCGAGCGTCATTTCAACCCTTAAGCCTAGCTTAG
TGTTGGGAATCTACTTCTTTA--
TAGTTGTAGTTCCTGAAATACAACGGCGGATTTGTAGTATCCTCTGAGCGTAGTAATTTTTTTTCT
CGCTTTTGTTAGGTGCTATAACTCCCAGCCGCTAAACCCCCAATTTTTTGTGGTTGACCTCGGAT
CAGGTAGAAGGTTAGTCATTTATTGATTCCCATCAT-------CATCCCCCTTCAC-
TTCAGCATCATAATTTTCAACCTACGTGTTGAAAATTA-TTTTCGCTCCTTCCACAC--TTTTT--
TCGCTGGTTACCCCGCCGCGAGGCACCCGCACGACCCCGCGGTGCAAACGAAAAATTTCTTAT
CACAGCCCCACCTTGCACAAGCAACCATGCATTGCTCATGAGACCCACTT--
TGAACAATTGCTAATGCCTTCATACAGGAAGCCGCCGAGCTCGGTAAGGGTTCCTTCAAGTACG
CCTGGGTTCTTGACAAGCTCAAGGCCGAGCGTGAGCGTGGTATCACCATCGATATCGCTCTCTG
GAAGTTCGAGACCAACGAGTACAATGTCACCGTCATTGGTTAGTACCCCTCCACCTATGCCATG
TGCTGCTCCATAAGACACTTGACTAACCTTGCTTCATAGACGCTCCCGGTCACCGTGATTTCATC
AAGAACA???????????AATACACAACGTGATCTGCTCGAC-
CGGCCTTAATACGACGTTTTTCGTGCCTGCACGACGGCCCCGAACAGTTGAAATAGGTCAAGAT
AGAGGGAACATAAGACTAATAGGTCATTTATAGGCAAACCATCTCTGGCGAGCACGGTCTCGA
CAGCAATGGAGTGTATGTACTAACTTCAATTCCTCCTGCTTCCTGTTGAGCTTGTAGGCTGAC-
TCGATGGCCATTTAGCTACAACGGTACCTCCGAGCTCCAGCTCGAGCGTATGAGCGTCTACTTC
AACGAGGCTTCCGGCAACAAGTACGTTCCTCGTGCTGTCCTCGTCGATCTCGAGCCCGGTACCA
TGGATGCCGTCCGCGCCGGTCCCTTCGGCCAGCTCTTCCGCCCTGACAACTTCGTCTTCGGTCA
GTCCGGTGCTGGCAACAACTGG
>N_coffeae_arabicae_HGUP_4019
CATTATAGAGTTTTCTAAACTCCCAACCCATGTGAACTTACCTTTTGTTGCCTCGGCAGAAGTTAT
AGGTCTTCTTATAGCTGCTGCCGGTGGACCATTAAACTCTTGTTATTTTATGTAATCTGAGCGTCT
TATTTTAATAAGTCAAAACTTTCAACAACGGATCTCTTGGTTCTGGCATCGATGAAGAACGCAGC
GAAATGCGATAAGTAATGTGAATTGCAGAATTCAGTGAATCATCGAATCTTTGAACGCACATTG
CGCCCATTAGTATTCTAGTGGGCATGCCTGTTCGAGCGTCATTTCAACCCTTAAGCCTAGCTTAG
TGTTGGGAATCTACTTCTTTA--
TAGTTGTAGTTCCTGAAATACAACGGCGGATTTGTAGTATCCTCTGAGCGTAGTAATTTTTTTTCT
CGCTTTTGTTAGGTGCTATAACTCCCAGCCGCTAAACCCCCAATTTTTTGTGGTTGACCTCGGAT
CAGGTAGAAGGTTAGTCATTTATTGATTCCCATCAT-------CATCCCCCTTCAC-
TTCAGCATCATAATTTTCAACCTACGTGTTGAAAATTA-TTTTCGCTCCTTCCACAC--TTTTT--

TCGCTGGTTACCCCGCCGCGAGGCACCCGCACGACCCCGCGGTGCAAACGAAAAATTTCTTAT
CACAGCCCCACCTTGCACAAGCAACCATGCATTGCTCATGAGACCCACTT--
TGAACAATTGCTAATGCCTTCATACAGGAAGCCGCCGAGCTCGGTAAGGGTTCCTTCAAGTACG
CCTGGGTTCTTGACAAGCTCAAGGCCGAGCGTGAGCGTGGTATCACCATCGATATCGCTCTCTG
GAAGTTCGAGACCAACGAGTACAATGTCACCGTCATTGGTTAGTACCCCTCCACCTATGCCATG
TGCTGCTCCATAAGACACTTGACTAACCTTGCTTCATAGACGCTCCCGGTCACCGTGATTTCATC
AAGAACA???????????GAGTTATTA-
CTGTCTGCCTCGACACGGCCTTAATACGACGTTTTTCGTGCCTGCACGACGGCCCCGAACAGTT
GAAATAGGTCAAGATAGAGGGAACATAAGACTAATAGGTCATTTATAGGCAAACCATCTCTGGC
GAGCACGGTCTCGACAGCAATGGAGTGTATGTACTAACTTCAATTCCTCCTGCTTCCTGTTGAGC
TTGTAGGCTGAC-
TCGATGGCCATTTAGCTACAACGGTACCTCCGAGCTCCAGCTCGAGCGTATGAGCGTCTACTTC
AACGAGGCTTCCGGCAACAAGTACGTTCCTCGTGCTGTCCTCGTCGATCTCGAGCCCGGTACCA
TGGATGCCGTCCGCGCCGGTCCCTTCGGCCAGCTCTTCCGCCCTGACAACTTCGTCTTCGGTCA
GTCCGGTGCTGGCAACAACTGG
>N_cubana_CBS_600_96
CATTATAGAGTTTTCTAAACTCCCAACCCATGTGAACTTACCTTTTGTTGCCTCGGCAGAAGTTAT
AGGTCTTCTTATAGCTGCTGCCGGTGGACCATTAAACTCTTGTTATTTTATGTAATCTGAGCGTCT
TATTTTAATAAGTCAAAACTTTCAACAACGGATCTCTTGGTTCTGGCATCGATGAAGAACGCAGC
GAAATGCGATAAGTAATGTGAATTGCAGAATTCAGTGAATCATCGAATCTTTGAACGCACATTG
CGCCCATTAGTATTCTAGTGGGCATGCCTGTTCGAGCGTCATTTCAACCCTTAAGCCTAGCTTAG
TGTTGGGAATCTACTTCTCTTAGGAGTTGTAGTTCCTGAAATACAACGGCGGATTTGTAGTATCC
TCTGAGCGTAGTAA-
TTTTTTTCTCGCTTTTGTTAGGTGCTATAACTCCCAGCCGCTAAACCCCCAATTTTTTGTGGTTGAC
CTCGGATCAGGT???AGGTTAGTCATCTATTGATTCCCATCAT-------CATTCCCCTTCAC-
TCCAGCGTCATGATTTTCAACCTACGCGTTGAAAATTA-TTTTCGCTCCTTCCACAC--TTTTT--
TCGCTGGTTACCCCGCCGCGAGGCACCCGCACGACCCCGCGGTGCAAACGAAAAATTTCTTAT
CACAGCCCCACCTTTCACAAGCAACCATGCATTGCTCATGAGACCCACTT--
TGAACAATTGCTAATGCCTTCATACAGGAAGCCGCCGAGCTCGGTAAGGGTTCCTTCAAGTACG
CCTGGGTTCTTGACAAGCTCAAGGCCGAGCGTGAGCGTGGTATCACCATCGATATCGCTCTCTG
GAAGTTCGAGACCAACGAGTACAATGTCACCGTCATTGGTTAGTACCCCTCCACCTATGCCATG
TGCTGCTCCATAAGACACTTGACTAACCTTGCTTCATAGACGCTCCCGGTCACCGTGATTTCATC
AAGAACATGCTGCCTTCTGGTATGTAACCTGTCTGTCTCGACACGGCCTCAATACGACGTTTTTC
GTGCCTGCACGACAGCCCCGAACAG-
TGAATTAGGTCAAGATAGAGGGAACATGATGCTAATAGGTCATTGATAGGCAAACCATCTCTGG
CGAGCACGGTCTCGACAGCAATGGAGTGTATGTACTATTTTCAATTCCTCCTGCTTCCTGTTGAG
CTTGTAGGCTGAC-
TCGATGGCCATTTAGCTACAACGGTACCTCCGAGCTCCAGCTCGAGCGTATGAGCGTCTACTTC
AACGAGGCTTCCGGCAACAAGTACGTTCCTCGTGCCGTCCTCGTCGATCTCGAGCCCGGTACCA
TGGATGCCGTCCGCGCCGGTCCCTTCGGCCAGCTCTTCCGCCCTGACAACTTCGTCTTCGGTCA
GT????????????????????
>N_dendrobii_MFLUCC_14_0106
CATTATAGAGTTTTCTAAACTCCCAACCCATGTGAACTTACCTTTTGTTGCCTCGGCAGAAGTTAT
AGGTCTTCTTATAGCTGCTGCCGGTGGACCATTAAACTCTTGTTATTTTATGTAATCTGAGCGTCT

TATTTTAATAAGTCAAAACTTTCAACAACGGATCTCTTGGTTCTGGCATCGATGAAGAACGCAGC
GAAATGCGATAAGTAATGTGAATTGCAGAATTCAGTGAATCATCGAATCTTTGAACGCACATTG
CGCCCATTAGTATTCTAGTGGGCATGCCTGTTCGAGCGTCATTTCAACCCTTAAGCCTAGCTTAG
TGTTGGGAATCTACTTCTCTTAGGAGTTGTAGTTCCTGAAATACAACGGCGGATTTGTAGTATCC
TCTGAGCGTAGTAA-
TTTTTTTCTCGCTTTTGTTAGGTGCTATAACTCCCAGCCGCTAAACCCCCAATTTTTT????????????
?????????AGAAGGTTAGTCATTTATTGATTCCCATCAT-------CATCCCCCTTCAC-
TTCAGCATCATAATTTTTAACCTACGTGTTGAAAATTA-TTTTCGCTCCTTCCACAC--TTTTT--
TCGCTGGTTACCCCGCCGCGAGGCACCCGCACGACCCCGCGGTGCAAACGAAAAATTTCTTAT
CACAGCCCCACCTTGCACAAGCAACCATGCATTGCTCATGAGACCCACTT--
TGAACAATTGCTAATGCCTTCATACAGGAAGCCGCCGAGCTCGGTAAGGGTTC???????????????
??
??
????????????????TGCTGCTTTCTGGTATGTAACCTGTCTGTCTCGTCACGGCCTCAATACGACGTT
TTTCGTGCCTGCACGACAGCCCCGAACAG-
TGAATTAGGTCAAGATAGAGGGAACATGATGCTAATAGGTCATTGATAGGCAAACCATCTCTGG
CGAGCACGGTCTCGACAGCAATGGAGTGTATGTACTATTTTCAATTCCTCCTGCTTCCTGTTGAG
CTTGTAGGCTGAC-
TCGATGGCCATTTAGCTACAACGGTACCTCCGAGCTCCAGCTCGAGCGCATGAGTGTCTACTTC
AACGAGGCTTCCGGCAACAAGTACGTTCCTCGTGCCGTCCTCGTCGATCTCGAGCCCGGTACCA
TGGATGCCGTCCGCGCCGGTCCCTTCGGCCAGCTCTTCCGCCCTGACAACTTCGTCTTCGGTCA
GTCCGGTGCTGGCAACAACTGG
>N_drenthii_BRIP_72263a
CATTATAGAGTTTTCTAAACTCCCAACCCATGTGAACTTACCTTTTGTTGCCTCGGCAGAAGTTAT
AGGTCTTCTTATAGCTGCTGCCGGTGGACCATTAAACTCTTGTTATTTTATGTAATCTGAGCGTCT
TATTTTAATAAGTCAAAACTTTCAACAACGGATCTCTTGGTTCTGGCATCGATGAAGAACGCAGC
GAAATGCGATAAGTAATGTGAATTGCAGAATTCAGTGAATCATCGAATCTTTGAACGCACATTG
CGCCCATTAGTATTCTAGTGGGCATGCCTGTTCGAGCGTCATTTCAACCCTTAAGCCTAGCTTAG
TGTTGGGAATCTACTTCTTTA--
TAGTTGTAGTTCCTGAAATACAACGGCGGATTTGTAGTATCCTCTGAGCGTAGTAA-
TTTTTTTCTCGCTTTTGTTAGGTGCTATAACTCCCAGCCGCTAAACCCCCAATTTTTTGTGGTTGAC
CTCGGATCAGGTAGAAGGTTAGTCATCTTTTGATTCCCATCAT-------CATTCCCCTCCAC-
TTCAGCGTCATGATTTTCAACCTACGTGTTGAAAATTA-TTTTCGCTCCTTCCACAC--TTTCT--
TCGCTGGTTACCCCGCCGCGAGGCACCCGCACGACCCCGCGGTGCAAACGAAAAATTTCTTAT
CACAGCCCCACCTTGCACAAGCAACCATGCATTGCTCATGAGACCCACTT--
TGAACAATTGCTAATGCCTTCATACAGGAAGCCGCCGAGCTCGGTAAGGGTTCCTTCAAGTACG
CCTGGGTTCTTGACAAGCTCAAGGCCGAGCGTGAGCGTGGTATCACCATCGATATCGCTCTCTG
GAAGTTCGAGACCAACGAGTACAATGTCACCGTCATTGGTTAGTACCCCTCCACCTATGCCATG
TGCTGCTCCATAAGACACTTGACTAACCTTGCTTCATAGACGCTCCCGGTCACCGTGATTTCATC
AAGAACA???????TTCTGGTATGTTACCTGTCTGCCTCGACACGGCCTTACTACGACGTTTTTCGT
GCCTGCACGACGGCCCCGAACAG-
TGAAATAGGTCAAGATAGAGGGAACATGATACTAATAGGTCATTTATAGGCAAACCATCTCTGG
CGAGCACGGTCTCGACAGCAATGGAGTGTATGTACTAACTTCAATTCCTCCTGCTTCCTGTTGAG
TTTGTAGGCTGAC-

TCGATGGCCATTTAGCTACAACGGTACCTCCGAGCTCCAGCTCGAGCGTATGAGCGTCTACTTC
AACGAGGCTTCCGGCAACAAGTACGTTCCTCGTGCCGTCCTCGTCGATCTCGAGCCCGGTACCA
TGGATGCCGTCCGCGCCGGTCCCTTCGGCCAGCTCTTCCGCCCTGACAACTTCGTCTTCGGTCA
GTCCGGTGCTGGCAACAACTGG
>N_drenthii_BRIP_72264a
CATTATAGAGTTTTCTAAACTCCCAACCCATGTGAACTTACCTTTTGTTGCCTCGGCAGAAGTTAT
AGGTCTTCTTATAGCTGCTGCCGGTGGACCATTAAACTCTTGTTATTTTATGTAATCTGAGCGTCT
TATTTTAATAAGTCAAAACTTTCAACAACGGATCTCTTGGTTCTGGCATCGATGAAGAACGCAGC
GAAATGCGATAAGTAATGTGAATTGCAGAATTCAGTGAATCATCGAATCTTTGAACGCACATTG
CGCCCATTAGTATTCTAGTGGGCATGCCTGTTCGAGCGTCATTTCAACCCTTAAGCCTAGCTTAG
TGTTGGGAATCTACTTCTTTA--
TAGTTGTAGTTCCTGAAATACAACGGCGGATTTGTAGTATCCTCTGAGCGTAGTAA-
TTTTTTTCTCGCTTTTGTTAGGTGCTATAACTCCCAGCCGCTAAACCCCCAATTTTTTGTGGTTGAC
CTCGGATCAGGTAGAAGGTTAGTCATCTTTTGATTCCCATCAT-------CATTCCCCTCCAC-
TTCAGCGTCATGATTTTCAACCTACGTGTTGAAAATTA-TTTTCGCTCCTTCCACAC--TTTCT--
TCGCTGGTTACCCCGCCGCGAGGCACCCGCACGACCCCGCGGTGCAAACGAAAAATTTCTTAT
CACAGCCCCACCTTGCACAAGCAACCATGCATTGCTCATGAGACCCACTT--
TGAACAATTGCTAATGCCTTCATACAGGAAGCCGCCGAGCTCGGTAAGGGTTCCTTCAAGTACG
CCTGGGTTCTTGACAAGCTCAAGGCCGAGCGTGAGCGTGGTATCACCATCGATATCGCTCTCTG
GAAGTTCGAGACCAACGAGTACAATGTCACCGTCATTGGTTAGTACCCCTCCACCTATGCCATG
TGCTGCTCCATAAGACACTTGACTAACCTTGCTTCATAGACGCTCCCGGTCACCGTGATTTCATC
AAGAACA????????TCTGGTATGTTACCTGTCTGCCTCGACACGGCCTTACTACGACGTTTTTCGT
GCCTGCACGACGGCCCCGAACAG-
TGAAATAGGTCAAGATAGAGGGAACATGATACTAATAGGTCATTTATAGGCAAACCATCTCTGG
CGAGCACGGTCTCGACAGCAATGGAGTGTATGTACTAACTTCAATTCCTCCTGCTTCCTGTTGAG
TTTGTAGGCTGAC-
TCGATGGCCATTTAGCTACAACGGTACCTCCGAGCTCCAGCTCGAGCGTATGAGCGTCTACTTC
AACGAGGCTTCCGGCAACAAGTACGTTCCTCGTGCCGTCCTCGTCGATCTCGAGCCCGGTACCA
TGGATGCCGTCCGCGCCGGTCCCTTCGGCCAGCTCTTCCGCCCTGACAACTTCGTCTTCGGTCA
GTCCGGTGCTGGCAACAACTGG
>N_egyptiaca_CBS_140162
CATTATAGAGTTTTCTAAACTCCCAACCCATGTGAACTTACCTTTTGTTGCCTCGGCAGAAGTTAT
AGGTCTTCTTATAGCTGCTGCCGGTGGACCATTAAACTCTTGTTATTTTATGTAATCTGAGCGTCT
TATTTTAATAAGTCAAAACTTTCAACAACGGATCTCTTGGTTCTGGCATCGATGAAGAACGCAGC
GAAATGCGATAAGTAATGTGAATTGCAGAATTCAGTGAATCATCGAATCTTTGAACGCACATTG
CGCCCATTAGTATTCTAGTGGGCATGCCTGTTCGAGCGTCATTTCAACCCTTAAGCCTAGCTTAG
TGTTGGGAATCTACTTCTTTA--
TAGTTGTAGTTCCTGAAATACAACGGCGGATTTGTAGTATCCTCTGAGCGTAGTAA-
TTTTTTTCTCGCTTTTGTTAGGTGCTATAACTCCCAGCCGCTAAACCCCCAATTTTTTGTGGTTGAC
CTCGGATCAGGTAGAAGGTTAGTCATCTACTGATTCCCGTCAT-------CATTCTCCTTCAC-
TTCAGCGTCATGATTTTTAACCTACGTGTTGAAAATTA-TTTTCGCTCCTTCCACAC--TTTTT--
TCGCTGGTTACCCCGCCGCGAGGCACCCGCACGACCCCGCGGTGCAAACGAAAAATTTCTTAT
CACAGCCCCACCTTGCATAAGCAACCATGCATTGCTCATGAGATCCACTT--
TGAACAATCGCTAATGCCTTCATACAGGAAGCCGCCGAGCTCGGTAAGGGTTCCTTCAAGTACG

CCTGGGTTCTTGACAAGCTCAAGGCCGAGCGTGAGCGTGGTATCACCATCGATATCGCTCTCTG
GAAGTTCGAGACCAACGAGTACAATGTCACCGTCATTGGTTAGTACCCCTCCACCTATGTTATGT
GCTGCTCCATAAGACACTTGACTAACCTTGCTTCATAGACGCTCCCGGTCACCGTGATTTCATCA
AGAACATGCTGCCTTCTGGTATGTAACCTGTCTGTCTCGACACGGCCTCAATACGACGTTTTTCG
TGCCTGCACGACAGCCCCGAACAG-
TGAATTAGGTCAAGATAGAGGGAACATGATGCTAATAGGTCATTGATAGGCAAACCATCTCTGG
CGAGCACGGTCTCGACAGCAATGGAGTGTATGTACTATTTTCAATTCCTCCTGCTTCCTGTTGAG
CTTGTAGGCTGAC-
TCGATGACCATTTAGCTACAACGGTACCTCCGAGCTCCAGCTCGAGCGTATGAGCGTCTACTTC
AACGAGGCTTCCGGCAACAAGTACGTTCCTCGTGCCGTCCTCGTCGATCTCGAGCCCGGTACCA
TGGATGCCGTCCGCGCCGGTCCCTTCGGCCAGCTCTTCCGCCCTGACAACTTCGTCTTCGGTCA
GTC???????????????????
>N_ellipsospora_MFLUCC_12_0283
CATTATAGAGTTTTCTAAACTCCCAACCCATGTGAACTTACCTTTTGTTGCCTCGGCAGAAGTTAT
AGGTCTTCTTATAGCTGCTGCCGGTGGACCATTAAACTCTTGTTATTTTATGTAATCTGAGCGTCT
TATTTTAATAAGTCAAAACTTTCAACAACGGATCTCTTGGTTCTGGCATCGATGAAGAACGCAGC
GAAATGCGATAAGTAATGTGAATTGCAGAATTCAGTGAATCATCGAATCTTTGAACGCACATTG
CGCCCATTAGTATTCTAGTGGGCATGCCTGTTCGAGCGTCATTTCAACCCTTAAGCCTAGCTTAG
TGTTGGGAATCTACTTCTCTTAGGAGTTGTAGTTCCTGAAATACAACGGCGGATTTGTAGTATCC
TCTGAGCGTAGTAA-
TTTTTTTCTCGCTTTTGTTAGGTGCTATAACTCCCAGCCGCTAAACCCCCAATTTTTTGTGGTTGAC
CTCGGATCAGGTAGAAGGTTAGTCATTTATTGATTCCAATCAT-------CATCCCCCTTCAC-
TTCAGCATCATAATTTTCAACCTACGTGTTGAAAATTA-TTTTCGCTCCTTCCACAC--TTTTT--
TCGCTGGTTACCCCGCCACGAGGCACCCGCACGACCCCGCGATGCAAACGAAAAATTTCTTAT
CACAGCCCCACCTTACACAAGCAACCATGCATTGCTCATGAGACCCACTT--
TGAACAATTGCTAATGCCTTCATACAGGAAGCCGCCGAGCTCGGTAAGGGTTCCTTCAAGTACG
CCTGGGTTCTTGACAAGCTCAAGGCCGAGCGTGAGCGTGGTATCACCATCGATATCGCTCTCTG
GAAGTTCGAGACCAACGAGTACAATGTCACCGTCATTGGTTAGTACCCCTCCACCTATGCCATG
TGCTGCTCCATAAGACACTTGACTAACCTTGCTTCATAGACGCTCCCGGTCACCGTGATTTCATC
AAGAACATGCTGCTTTCTGGTATGTAATCTGTCTGCCTCGACACGGCCTTAATACGACGTTTTTC
GTGCCTGCACGATGGCCCCGATCAG-
TGAATTAGGTCAAGATACAGGGAACATGATGCTAATAGGTCATTTATAGGCAAACCATCTCTGG
CGAGCACGGTCTCGACAGCAATGGAGTGTATGTACTATTTTCAATTCCTCCTGCTTCCTGTTGAG
CTTGTAGGCTGAC-
TCGATGGCCATTTAGCTACAACGGTACCTCCGAGCTCCAGCTCGAGCGTATGAGCGTCTACTTC
AACGAGGCTTCCGGCAACAAGTACGTTCCTCGTGCCGTCCTCGTCGATCTCGAGCCCGGTACCA
TGGATGCCGTCCGCGCCGGTCCCTTCGGCCAGCTCTTCCGCCCTGACAACTTCGTCTTCGGTCA
GTCCGGTGCTGGCAACAACTGG
>N_eucalypticola_CBS_264_37
CATTATAGAGTTTTCTAAACTCCCAACCCATGTGAACTTACCTTTTGTTGCCTCGGCAGAAGTTAT
AGGTCTTCTTATAGCTGCTGCCGGTGGACCATTAAACTCTTGTTATTTTATGTAATCTGAGCGTCT
TATTTTAATAAGTCAAAACTTTCAACAACGGATCTCTTGGTTCTGGCATCGATGAAGAACGCAGC
GAAATGCGATAAGTAATGTGAATTGCAGAATTCAGTGAATCATCGAATCTTTGAACGCACATTG
CGCCCATTAGTATTCTAGTGGGCATGCCTGTTCGAGCGTCATTTCAACCCTTAAGCCTAGCTTAG

TGTTGGGAATCTACTTCTTTA--
TAGTTGTAGTTCCTGAAATACAACGGCGGATTTGTAGTATCCTCTGAGCGTAGTAA-
TTTTTTTCTCGCTTTTGTTAGGTGCTATAACTCCCAGCCGCTAAACCCCCAATTTTTTGTGGTTGAC
CTCGGATCAGGTAGAAGGTTAGTCATCTATTGATTCCCATCAT-------CATTCCCCTTCAC-
TTCAGCGTCATGATTTTCAACATACGTGTTGAAAATTA-TTTTCGCTCCTTCCACACTTTTTTT--
TCGCTGGTTACCCCGCCGCGAGGCACCCGCACGACCCCGCGGTGCAAACGAAAAATTTCTTAT
CACAGCCCCACCTTGCACAAGCAACCATGCATTACTCATGAGACCCACTT--
TGAACAATTGCTAATTCCTTCATTCAGGAAGCCGCCGAGCTCGGTAAGGGTTCCTTCAAGTACG
CCTGGGTTCTTGACAAGCTCAAGGCCGAGCGTGAGCGTGGTATCACCATCGATATCGCTCTCTG
GAAGTTCGAGACCAACGAGTACAATGTCACCGTCATTGGTCAGTACCCCTCCACCTATGCCATG
TACTGCTCCATAAGACACTTGACTAACCTTGCTTCATAGACGCTCCCGGTCACCGTGATTTCATC
AAGAACATGCTGCCTTCTGGTATGTAACCTGTCTGTCTCGACACGGCCTCAATACGACGTTTTTC
GTGCCTGCACGACGGCCCCGAACAG-
TGAATTAGGTCAAGATAGAGGGAACATGATGCTAATAGGTCATTGATAGGCAAACCATCTCTGG
CGAGCACGGTCTCGACAGCAATGGAGTGTATGTACTATTTTTAATTCCTCCTGCTTCCTGTTAAG
CTTGTAGGCTGAC-
TCGATGGCCATTTAGCTACAACGGTACCTCCGAGCTCCAGCTCGAGCGTATGAGCGTCTACTTC
AACGAGGCTTCCGGCAACAAGTACGTTCCTCGTGCCGTCCTCGTCGATCTCGAGCCCGGTACCA
TGGATGCCGTCCGCGCCGGTCCCTTCGGCCAGCTCTTCCGCCCTGACAACTTCGTCTTCGGTCA
GTCCGGTGCTGGCAACAACTGG
>N_eucalyptorum_CBS_147684
?????????????????AACTCCCAACCCATGTGAACTTACCTTTTGTTGCCTCGGCAGAAGTTATAGGT
CTTCTTATAGCTGCTGCCGGTGGACCATTAAACTCTTGTTATTTTATGTAATCTGAGCGTCTTATTT
TAATAAGTCAAAACTTTCAACAACGGATCTCTTGGTTCTGGCATCGATGAAGAACGCAGCGAAA
TGCGATAAGTAATGTGAATTGCAGAATTCAGTGAATCATCGAATCTTTGAACGCACATTGCGCC
CATTAGTATTCTAGTGGGCATGCCTGTTCGAGCGTCATTTCAACCCTTAAGCCTAGCTTAGTGTT
GGGAATCTACTTCTTTTATTAGTTGTAGTTCCTGAAATACAACGGCGGATTTGTAGTATCCTCTGA
GCGTAGTAA-
TTTTTTTCTCGCTTTTGTTAGGTGCTATAACTCCCAGCCGCTAAACCCCCAATTTTTTGTGGTTGAC
CTCGGATCAGGT???????????????????GATTCCCATCAT-------CATTCCCCTTCAC-
TTCGGCATCATAATTTTCAACCTACGTGTTGAAAATTA-TTTTCGCTCTTTCCACAC--TTTTT--
TCGATGGTTACCCCGCCGCGAGGCACCCGCACGACCCCGCGGTGCAAACGAAAAATTTCTTAT
CACAGCCCCACCTTGCACAAGCAACCATGCATTGCTCATGAGACCCACTT--
TGAACAATTGCTAATGCCTTCATACAGGAAGCCGCCGAGCTCGGTAAGGGTTCCTTCAAGTACG
CCTGGGTTCTTGACAAGCTCAAGGCCGAGCGTGAGCGTGGTATCACCATCGATATCGCTCTCTG
GAAGTTCGAGACCAACGAGTACAATGTCACCGTCATTGGTTAGTACCCCTACACCCATGCCATG
TGCTGCTCCATAAGACACTTGACTAACCTTGCTTCATAGACGCTCCCGGTCACCGTGATTTCATC
AAGAACA?????????????????????????????????GACACGGCCTTAATACGACGTTTTTCGTGCCTGC
ACGACGGCCCCGAACAG-
TGATATAGGTCAGGATAGAGGGAACATGATGCTAATAGGTCATTGATAGGCAAACCATCTCTGG
CGAGCACGGTCTCGACAGCAATGGAGTGTATGTACTATTTTTAATTCCTCCTGCTTCCTGTTAAG
CTTGTAGGCTGAC-
TCGATGGCCATTTAGCTACAACGGTACCTCCGAGCTCCAGCTCGAGCGTATGAGCGTCTACTTC
AACGAGGCTTCCGGCAACAAGTACGTTCCTCGTGCCGTCCTCGTCGATCTCGAGCCCGGTACCA

TGGATGCCGTCCGCGCCGGTCCCTTCGGCCAGCTCTTCCGCCCTGACAACTTCGTCTTCGGTCA
GTCCGGTGCTGGCAACAACTGG
>N_eucalyptorum_PE194
CATTATAGAGTTTTCTAAACTCCCAACCCATGTGAACTTACCTTTTGTTGCCTCGGCAGAAGTTAT
AGGTCTTCTTATAGCTGCTGCCGGTGGACCATTAAACTCTTGTTATTTTATGTAATCTGAGCGTCT
TATTTTAATAAGTCAAAACTTTCAACAACGGATCTCTTGGTTCTGGCATCGATGAAGAACGCAGC
GAAATGCGATAAGTAATGTGAATTGCAGAATTCAGTGAATCATCGAATCTTTGAACGCACATTG
CGCCCATTAGTATTCTAGTGGGCATGCCTGTTCGAGCGTCATTTCAACCCTTAAGCCTAGCTTAG
TGTTGGGAATCTACTTCTTTTATTAGTTGTAGTTCCTGAAATACAACGGCGGATTTGTAGTATCCT
CTGAGCGTAGTAA-
TTTTTTTCTCGCTTTTGTTAGGTGCTATAACTCCCAGCCGCTAAACCCCCAATTTTTTGTGGTTGAC
CTCGGATCAGGT?????????????????TTGATTCCCATCAT-------CATTCCCCTTCAC-
TTCGGCATCATAATTTTCAACCTACGTGTTGAAAATTA-TTTTCGCTCTTTCCACAC--TTTTT--
TCGATGGTTACCCCGCCGCGAGGCACCCGCACGACCCCGCGGTGCAAACGAAAAATTTCTTAT
CACAGCCCCACCTTGCACAAGCAACCATGCATTGCTCATGAGACCCACTT--
TGAACAATTGCTAATGCCTTCATACAGGAAGCCGCCGAGCTCGGTAAGGGTTCCTTCAAGTACG
CCTGGGTTCTTGACAAGCTCAAGGCCGAGCGTGAGCGTGGTATCACCATCGATATCGCTCTCTG
GAAGTTCGAGACCAACGAGTACAATGTCACCGTCATTGGTTAGTACCCCTACACCCATGCCATG
TGCTGCTCCATAAGACACTTGACTAACCTTGCTTCATAGACGCTCCCGGTCACCGTGATTTCATC
AAGAACA??????????????????AACCTGTCTGCCTCGACACGGCCTTAATACGACGTTTTTCGTGC
CTGCACGACGGCCCCGAACAG-
TGATATAGGTCAGGATAGAGGGAACATGATGCTAATAGGTCATTGATAGGCAAACCATCTCTGG
CGAGCACGGTCTCGACAGCAATGGAGTGTATGTACTATTTTTAATTCCTCCTGCTTCCTGTTAAG
CTTGTAGGCTGAC-
TCGATGGCCATTTAGCTACAACGGTACCTCCGAGCTCCAGCTCGAGCGTATGAGCGTCTACTTC
AACGAGGCTTCCGGCAACAAGTACGTTCCTCGTGCCGTCCTCGTCGATCTCGAGCCCGGTACCA
TGGATGCCGTCCGCGCCGGTCCCTTCGGCCAGCTCTTCCGCCCTGACAACTTCGTCTTCGGTCA
GTCCGGTGCTGGCAACAACTGG
>N_foedans_CGMCC_3_9123
CATTATAGAGTTTTCTAAACTCCCAACCCATGTGAACTTACCTTTTGTTGCCTCGGCAGAAGTTAT
AGGTCTTCTTATAGCTGCTGCCGGTGGACCATTAAACTCTTGTTATTTTATGTAATCTGAGCGTCT
TATTTTAATAAGTCAAAACTTTCAACAACGGATCTCTTGGTTCTGGCATCGATGAAGAACGCAGC
GAAATGCGATAAGTAATGTGAATTGCAGAATTCAGTGAATCATCGAATCTTTGAACGCACATTG
CGCCCATTAGTATTCTAGTGGGCATGCCTGTTCGAGCGTCATTTCAACCCTTAAGCCTAGCTTAG
TGTTGGGAATCTACTTCTCTTAGGAGTTGTAGTTCCTGAAATACAACGGCGGATTTGTAGTATCC
TCTGAGCGTAGTAA-
TCTTTTTCTCGCTTTTGTTAGGTGCTATAACTCCCAGCCGCTAAACCCCCAATTTTTTGTGGTTGA
CCTCGGATCAGGTAGAAGGTTAGTCATGTATTGATTCCCATCAT----------CCCCCTTCAC-
TTCAGCATCATAAATTTCAACCTGCGTGTTGAAAATTA-TTTTCGCTCCTTCCACACTTTTTTT---
CGCTGGTTACCCCGCCGCGAGGCACCCGCACGACCCCGCGGTGCAAACGAAAAATTTCTTATC
ACAGCCCCACCTTGCACAAGCAACCATGCATTGCTCATGAGACCCACTT--
TGAACAATTGCTAATGCCTTCATACAGGAAGCCGCCGAGCTCGGTAAGGGTTCCTTCAAGTACG
CCTGGGTTCTTGACAAGCTCAAGGCCGAGCGTGAGCGTGGTATCACCATCGATATCGCTCTCTG
GAAGTTCGAGACCAACGAGTACAATGTCACCGTCATTGGTTAGTACCCCTCCACCTATGCCATG

TGCTGCTCCATAAGACACTTGACTAACCTTGCTTCAAAGACGCTCCCGGTCACCGTGATTTCATC
AAGAACATGCTGCTTTCTGGTATGTAACCTGTCTGCCTCGACACGGCCTTAATACGACGTTTTTC
GTGCCTGCACGACGGCCCCGAACAG-
TGAATTAGGTCAAGATAGAGGGAACATGATGCTAATAGGTCATTGATAGGCAAACCATCTCTGG
CGAGCACGGTCTCGACAGCAATGGAGTGTATGTACTATTTTTAATTCCTCCTGCTTCCTGTTAAG
CTTGTAGGCTGAC-
TCGATGGCCATTTAGCTACAACGGTACCTCCGAGCTCCAGCTCGAGCGTATGAGCGTCTACTTC
AACGAGGCTTCCGGCAACAAGTACGTTCCTCGTGCCGTCCTCGTCGATCTCGAGCCCGGTACCA
TGGATGCCGTCCGCGCCGGTCCCTTCGGCCAGCTCTTCCGCCCTGACAACTTCGTCTTCGGTCA
GTCCGGTGCTGGCAACAACTGG
>N_formicarum_CBS_115_83
CATTATAGAGTTTTCTAAACTCCCAACCCATGTGAACTTACCTTTTGTTGCCTCGGCAGAAGTTAT
AGGTCTTCTTATAGCTGCTGCCGGTGGACCATTAAACTCTTGTTATTTTATGTAATCTGAGCGTCT
TATTTTAATAAGTCAAAACTTTCAACAACGGATCTCTTGGTTCTGGCATCGATGAAGAACGCAGC
GAAATGCGATAAGTAATGTGAATTGCAGAATTCAGTGAATCATCGAATCTTTGAACGCACATTG
CGCCCATTAGTATTCTAGTGGGCATGCCTGTTCGAGCGTCATTTCAACCCTTAAGCCTAGCTTAG
TGTTGGGAATCTACTTCTTTTATTAGTTGTAGTTCCTGAAATACAACGGCGGATTTGTAGTATCCT
CTGAGCGTAGTAA-
TTTTTTTCTCGCTTTTGTTAGGTGCTATAACTCCCAGCCGCTAAACCCCCAATTTTTTGTGGTTGAC
CTCGGATCAGGTAGAAGGTTAGTCATCTATTGATTCCCATCAT-------CATTCCCCTTCTC-
TTCAGCGTCATGATTTTCAACCTACGCGTTGAAAATTA-TTTTCGCTCCTTCCACAC--TTTTT--
TCGCTGGTTACCCCGCCGCGAGGCACCCGCACGACCCCGCGGTGCAAACGAAAAATTTCTTAT
CACAGCCCCACCTTTCACAAGCAACCATGCATTGCTCATGAGACCCACTT--
TGAACGATTGCTAATGCCTTCATACAGGAAGCCGCCGAGCTCGGTAAGGGTTCCTTCAAGTACG
CCTGGGTTCTTGACAAGCTCAAGGCCGAGCGTGAGCGTGGTATCACCATCGATATCGCTCTCTG
GAAGTTCGAGACCAACGAGTACAATGTCACCGTCATTGGTTAGTACCCCTCCACCTATGCCATG
TGCTGCTCCATAAGACACTTGACTAACCTTGCTTCATAGACGCTCCCGGTCACCGTGATTTCATC
AAGAACATGCTGCCTTCTGGTATGTAATCTGTCTGCCTCGACACGGCCTTAATACGACGTTTTTC
GTGCCTGCACGACGGCCCCGATCAG-
TGAATTAGGTCAAGATACAGGGAACATGATGCTAATAGGTCATTTATAGGCAAACCATCTCTGG
CGAGCACGGTCTCGACAGCAATGGAGTGTATGTACTATTTTCAATTCCTCCTGCTTCCTGTTGAG
CTTGTAGGCTGAC-
TCGATGGCCATTTAGCTACAACGGTACCTCCGAGCTCCAGCTCGAGCGTATGAGCGTCTACTTC
AACGAGGCTTCCGGCAACAAGTACGTTCCTCGTGCCGTCCTCGTCGATCTCGAGCCCGGTACCA
TGGATGCCGTCCGCGCCGGTCCCTTCGGCCAGCTCTTCCGCCCTGACAACTTCGTCTTCGGTCA
GTCCGGTGCTGGCAACAACTGG
>N_formicarum_CBS_362_72
CATTATAGAGTTTTCTAAACTCCCAACCCATGTGAACTTACCTTTTGTTGCCTCGGCAGAAGTTAT
AGGTCTTCTTATAGCTGCTGCCGGTGGACCATTAAACTCTTGTTATTTTATGTAATCTGAGCGTCT
TATTTTAATAAGTCAAAACTTTCAACAACGGATCTCTTGGTTCTGGCATCGATGAAGAACGCAGC
GAAATGCGATAAGTAATGTGAATTGCAGAATTCAGTGAATCATCGAATCTTTGAACGCACATTG
CGCCCATTAGTATTCTAGTGGGCATGCCTGTTCGAGCGTCATTTCAACCCTTAAGCCTAGCTTAG
TGTTGGGAATCTACTTCTCTTAGGAGTTGTAGTTCCTGAAATACAACGGCGGATTTGTAGTATCC
TCTGAGCGTAGTAA-

TTTTTTTCTCGCTTTTGTTAGGTGCTATAACTCCCAGCCGCTAAACCCCCAATTTTTTGTGGTTGAC
CTCGGATCAGGTAGAAGGTTAGTCATCTATTGATTCCCATCAT-------CATTCACCTTCTC-
TTCAGCGTCATGATTTTCAACCTACGCGTTGAAAATTA-TTTTCGCTCCTTCCACAC--TTTTT--
TCGCTGGTTACCCCGCCGCGAGGCACCCGCACGACCCCGCGGTGCAAACGAAAAATTTCTTAT
CACAGCCCCACCTTTCACAAGCAACCATGCATTGCTCATGAGACCCACTT--
TGAACGATTGCTAATGCCTTCATACAGGAAGCCGCCGAGCTCGGTAAGGGTTCCTTCAAGTACG
CCTGGGTTCTTGACAAGCTCAAGGCCGAGCGTGAGCGTGGTATCACCATCGATATCGCTCTCTG
GAAGTTCGAGACCAACGAGTACAATGTCACCGTCATTGGTTAGTACCCCTCCACCTATGCCATG
TGCTGCTCCATAAGACACTTGACTAACCTTGCTTCATAGACGCTCCCGGTCACCGTGATTTCATC
AAGAACATGCTGCCTTCTGGTATGTAATCTGTCTGCCTCGACACGGCCTTAATACGACGTTTTTC
GTGCCTGCACGACGGCCCCGATCAG-
TGAATTAGGTCAAGATACAGGGAACATGATGCTAATAGGTCATTTATAGGCAAACCATCTCTGG
CGAGCACGGTCTCGACAGCAATGGAGTGTATGTACTATTTTCAATTCCTCCTGCTTCCTGTTGAG
CTTGTAGGCTGAC-
TCGATGGCCATTTAGCTACAACGGTACCTCCGAGCTCCAGCTCGAGCGTATGAGCGTCTACTTC
AACGAGGCTTCCGGCAACAAGTACGTTCCTCGTGCCGTCCTCGTCGATCTCGAGCCCGGTACCA
TGGATGCCGTCCGCGCCGGTCCCTTCGGCCAGCTCTTCCGCCCTGACAACTTCGTCTTCGGTCA
GTCCGGTGCTGGCAACAACTGG
>N_guajavae_FMB0026
CATTATAGAGTTTTCTAAACTCCCAACCCATGTGAACTTACCTTTTGTTGCCTCGGCAGAAGTTAT
AGGTCTTCTTATAGCTGCTGCCGGTGGACCATTAAACTCTTGTTATTTTATGTAATCTGAGCGTCT
TATTTTAATAAGTCAAAACTTTCAACAACGGATCTCTTGGTTCTGGCATCGATGAAGAACGCAGC
GAAATGCGATAAGTAATGTGAATTGCAGAATTCAGTGAATCATCGAATCTTTGAACGCACATTG
CGCCCATTAGTATTCTAGTGGGCATGCCTGTTCGAGCGTCATTTCAACCCTTAAGCCTAGCTTAG
TGTTGGGAATCTACTTCTTTA--
TAGTTGTAGTTCCTGAAATACAACGGCGGATTTGTAGTATCCTCTGAGCGTAGTAA-
TTTTTTTCTCGCTTTTGTCAGGTGCTATAACTCCCAGCCGCTAAACCCCCAATTTTTTGTGGTTGA
CCTCGGATCAGGT??
ACCTACGTGTTGAAAAATA-TTTTCGCTCCTTCCACAC--TTTTT---
CGCTGGTTACCCCGCCGCGAGGCACCCGCACGACCCCGCGGCGCAAACGAAAAATTTCTTATC
ACAGCCCCACCTTGCATAAGCAACCATGCATTGCTCATGAGATCCACTT--
TGAACAATTGCTAATGCCTTCATACAGGAAGCCGCCGAGCTCGGTAAGGGTTCCTTCAAGTACG
CCTGGGTTCTTGACAAGCTCAAGGCCGAGCGTGAGCGTGGTATCACCATCGATATCGCTCTCTG
GAAGTTCGAGACCAACGAGTACAATGTCACCGTCATTGGTTAGTACCCCTCCACCTATGCCATG
TGCTGCTGCATAAGACACTTGACTAACCTTGCTTCATAGACGCTCCCGGTCACCGTGATTTCATC
???????TGCTGCCTTCTGGTATGTTACCTGTCTGCCTCGACACGGCCTTACTACGACGTTTTTCGTG
CCTGCACGACGGCCCCGAACAG-
TGAAATAGGTCAAGATAGAGGGAACATGATACTAATAGGTCATTTATAGGCAAACCATCTCTGG
CGAGCACGGTCTCGACAGCAATGGAGTGTATGTACTAACTTCAATTCCTCCTGCTTCCTGTTGAG
TTTGTAGGCTGAC-
TCGATGGCCATTTAGCTACAACGGTACCTCCGAGCTCCAGCTCGAGCGTATGAGCGTCTACTTC
AACGAGGCTTCCGGCAACAAGTACGTTCCTCGTGCCGTCCTCGTCGATCTCGAG??????????????
??
>N_guajavicola_FMB0129

???????GAGTTTTCTAAACTCCCAACCCATGTGAACTTACCTTTTGTTGCCTCGGCAGAAGTTATA
GGTCTTCTTATAGCTGCTGCCGGTGGACCATTAAACTCTTGTTATTTTATGTAATCTGAGCGTCTT
ATTTTAATAAGTCAAAACTTTCAACAACGGATCTCTTGGTTCTGGCATCGATGAAGAACGCAGCG
AAATGCGATAAGTAATGTGAATTGCAGAATTCAGTGAATCATCGAATCTTTGAACGCACATTGC
GCCCATTAGTATTCTAGTGGGCATGCCTGTTCGAGCGTCATTTCAACCCTTAAGCCTAGCTTAGT
GTTGGGAATCTACTTCTTTA--
TAGTTGTAGTTCCTGAAATACAACGGCGGATTTGTAGTATCCTCTGAGCGTAGTAA-
TTTTTTTCTCGCTTTTGTTAGGTGCTATAACTCCCAGCCGCTAAACCCCCAATTTTTTGTGGTTGAC
CTCGGATCAGGT???A
CCTACGTGTTGAAAATTA-TTTTCGCTCCTTCCACAC--TTTTT--
TCGCTGGTTACCCCGCCGCGAGGCACCCGCACGACCCCGCGGTGCAAACGAAAAATTTCTTAT
CACAGCCCCACCTTGCACAAGCAACCATGCATTGCTCATGAGACCCACTT--
TGAACAATTGCTAATGCCTTCATACAGGAAGCCGCCGAGCTCGGTAAGGGTTCCTTCAAGTACG
CCTGGGTTCTTGACAAGCTCAAGGCCGAGCGTGAGCGTGGTATCACCATCGATATCGCTCTCTG
GAAGTTCGAGACCAACGAGTACAATGTCACCGTCATTGGTTAGTACCCCTCCACCTATGCCATG
TGCTGCACCATAAGACACTTGACTAACCTTG???TGCT
GCCTTCTGGTATGTAACCTGTCTGTCTCGACACGGCCTCAATACGACGTTTTTCGTGCCTGCACG
ACAGCCCCGAACAG-
TGAATTAGGTCAAGATAGAGGGAACATGATGCTAATAGGTCATTGATAGGCAAACCATCTCTGG
CGAGCACGGTCTCGACAGCAATGGAGTGTATGTACTATTTTCAATTCCTCCTGCTTCCTGTTGAG
CTTGTAGGCTGAC-
TCGATGGCCATTTAGCTACAACGGTACCTCCGAGCTCCAGCTCGAGCGTATGAGCGTCTACTTC
AACGAGGCTTCCGGCAACAAGTACGTTCCTCGTGCCGTCCTCGTCGATCTCGAGCCCGGTACCA
TGGATGCCGTCCGCGCCGGTCCCTTCGGCCAGCTCTTCCGCCCTGACAACTTCGTCTTCGGTCA
GTCCGGTGCTGGCAACAACTGG
>N_hadrolaeliae_VIC_47180
???CTCGGCAGAAGTTATAGGTCTTCTTATA
GCTGCTGCCGGTGGACCATTAAACTCTTGTTATTTTATGTAATCTGAGCGTCTTATTTTAATAAGT
CAAAACTTTCAACAACGGATCTCTTGGTTCTGGCATCGATGAAGAACGCAGCGAAATGCGATAA
GTAATGTGAATTGCAGAATTCAGTGAATCATCGAATCTTTGAACGCACATTGCGCCCATTAGTAT
TCTAGTGGGCATGCCTGTTCGAGCGTCATTTCAACCCTTAAGCCTAGCTTAGTGTTGGGAATCTA
CTTCTTTA--
TAGTTGTAGTTCCTGAAATACAACGGCGGATTTGTAGTATCCTCTGAGCGTAGTAA-
TTTTTTTCTCGCTTTTGTTAGGTGCTATAACTCCCAGCCGCTAAACCCCCAATTTTTTGTGGTTGAC
CTCGGATCAGGT????GGTTAGTCATCTATTGATTCCCATCAT-------CATTCCCCTTCAC-
TTCAGCGTCATGATTTTCAACATACGTGTTGAAAATTA-TTTTCGCTCCTTCCACACTTTTTTT--
TCGCTGGTTACCCCGCCGCGAGGCACCCGCACGACCCCGCGGTGCAAACGAAAAATTTCTTAT
CACAGCCCCACCTTGCACAAGCAACCATGCATTGCTCATGAGACCCACTT--
TGAACAATTGCTAATTCCTTCATTCAGGAAGCCGCCGAGCTCGGTAAGGGTTCCTTCAAGTACG
CCTGGGTTCTTGACAAGCTCAAGGCCGAGCGTGAGCGTGGTATCACCATCGATATCGCTCTCTG
GAAGTTCGAGACCAACGAGTACAATGTCACCGTCATTGGTCAGTACCCCTCCACCTATGCCATG
TACTGCTCCATAAGACACTTGACTAACCTTGCTTCATAGACGCTCCCGGTCACCGTGATTTCATC
AAG????TGCTGCCTTCTGGTATGTAACCTGTCTGCCTCGACACGGCCTTGATACGACGTTTTTCG
TGCCTGCACGACGGCCCCGAACAG-

TGAATTAGGTCAAGATACAGGGAACATGATGCTAATAGGTCATTTATAGGCAAACCATCTCTGG
CGAGCACGGTCTCGACAGCAATGGAGTGTATGTACCATTTTCAATCCCTCCTGCTTCCTGTTGAG
CTTGTAGGCTGAC-
TCGATGGCCATTTAGCTACAACGGTACCTCCGAGCTTCAGCTCGAGCGTATGAGCGTCTACTTC
AACGAGGCTTCTGGCAACAAGTACGTTCCTCGTGCCGTCCTCGTCGATCTCGAGCCCGGTACCA
TGGATGCCGTCCGCGCTGGTCCCTTCGGCCAGCTCTTCCGCCCTGACAACTTCGTCTTCGGTCA
GTCCGGTGCTGGCAACAA????
>N_hispanica_CBS_147686
?????????????????AACTCCC-
ACCCATGTGAACTTACCTTTTGTTGCCTCGGCAGAAGTTATAGGTCTTCTTATAGCTGCTGCCGG
TGGACCATTAAACTCTTGTTATTTTATGTAATCTGAGCGTCTTATTTTAATAAGTCAAAACTTTCAA
CAACGGATCTCTTGGTTCTGGCATCGATGAAGAACGCAGCGAAATGCGATAAGTAATGTGAATT
GCAGAATTCAGTGAATCATCGAATCTTTGAACGCACATTGCGCCCATTAGTATTCTAGTGGGCAT
GCCTGTTCGAGCGTCATTTCAACCCTTAAGCCTAGCTTAGTGTTGGGAATCTACTTCTTTTATTAG
TTGTAGTTCCTGAAATACAACGGCGGATTTGTAGTATCCTCTGAGCGTAGTAATTTTTTTTCTCGC
TTTTGTTAGGTGCTATAACTCCCAGCCGCTAAACCCCCAATTTTTTGTGGTTGACCTCGGATCAG
GT???????????????????GATTCCCGTCAT-------CATCCCCCTTCAC-
TTCAGCATCATAATTTTCAACCTACGTGTTGAAAATTA-TTCTCGCTCCTTCCACAC--TTTTT--
TCGCTGGTTACCCCGCCGCGAGGCACCCGCACGACCCCGCGGTGCAAACGAAAAATTTCTTAT
CACAGCCCCACCTTGCACAAGCAACCATGCATTGCTCATGAGACCCACTT--
TGAACAATTGCTAATGCCTTCATACAGGAAGCCGCCGAGCTCGGTAAGGGTTCCTTCAAGTACG
CCTGGGTTCTTGACAAGCTCAAGGCCGAGCGTGAGCGTGGTATCACCATCGATATCGCTCTCTG
GAAGTTCGAGACCAACGAGTACAATGTCACCGTCATTGGTTAGTACCCCTCCACCTATGCCATG
TGCTGCTCCATAAGACACTTGACTAACCTTGCTTCATAGACGCTCCCGGTCACCGTGATTTCATC
AAGAACA?????????????????????????????????GACACGGCCTTGATACGACGTTTTTCGTGCCTGC
ACGACGGCCCCGAACAG-
TGAATTAGGTCAAGATAGAGGGAACATGATGCTAATAGGTCATTGATAGGCAAACCATCTCTGG
CGAGCACGGTCTCGACAGCAATGGAGTGTATGTACTATTTTCAATTCCTCCTGCTTCCTGTTGAG
CTTGTAGGCTGAC-
TCGATGGCCATTTAGCTACAACGGTACCTCCGAGCTCCAGCTCGAGCGTATGAGCGTCTACTTC
AACGAGGCTTCCGGTAACAAGTACGTTCCTCGTGCCGTCCTCGTCGATCTCGAGCCCGGTACCA
TGGATGCCGTCCGCGCCGGTCCCTTCGGCCAGCTCTTCCGCCCTGACAACTTCGTCTTCGGTCA
GTCCGGTGCTGGCAACAACTGG
>N_honoluluana_CBS_114495
CATTATAGAGTTTTCTAAACTCCCAACCCATGTGAACTTACCTTTTGTTGCCTCGGCAGAAGTTAT
AGGTCTTCTTATAGCTGCTGCCGGTGGACCATTAAACTCTTGTTATTTTATGTAATCTGAGCGTCT
TATTTTAATAAGTCAAAACTTTCAACAACGGATCTCTTGGTTCTGGCATCGATGAAGAACGCAGC
GAAATGCGATAAGTAATGTGAATTGCAGAATTCAGTGAATCATCGAATCTTTGAACGCACATTG
CGCCCATTAGTATTCTAGTGGGCATGCCTGTTCGAGCGTCATTTCAACCCTTAAGCCTAGCTTAG
TGTTGGGAATCTACTTCTTTA--
TAGTTGTAGTTCCTGAAATACAACGGCGGATTTGTAGTATCCTCTGAGCGTAGTAA--
TTTTTTCTCGCTTTTGTTAGGTGCTATAACTCCCAGCCGCTAAACCCCCAATTTTTTGTGGTTGAC
CTCGGATCAGGTAGAAGGTTAGTCATCTATTGATTCCCATCAT-------CATTCCCCTTCAC-
TTCAGCGTCATGATTTTCAACATGCGTGTTGAAAATTA-TTTTCGCTCCTTCCACAC-TTTTTT--

TCGCTGGTTACCCCGCCGCGAGGCACCCGCACGACCCCGCGGTGCAAACGAAAAATTTCTTAT
CACAGCCCCACCTTGCACAAGCAACCATGCATTACTCATGAGACCCACTT--
TGAACAATTGCTAATTCCTTCATTCAGGAAGCCGCCGAGCTCGGTAAGGGTTCCTTCAAGTACG
CCTGGGTTCTTGACAAGCTCAAGGCCGAGCGTGAGCGTGGTATCACCATCGATATCGCTCTCTG
GAAGTTCGAGACCAACGAGTACAATGTCACCGTCATTGGTCAGTACCCTTCCACCTATGCCATG
TACTGCTCCATAAGACACTTGACTAACCTTGCTTCATAGACGCTCCCGGTCACCGTGATTTCATC
AAGAACATGCTGCCTTCTGGTATGTAACCTGTCTGCCTCGACACGGCCTTGATACGACGTTTTTC
GTGCCTGCACGACGGCCCCGAACAG-
TGAATTAGGTCAAGATACAGGGAACATGATGCTAATAGGTCATTTATAGGCAAACCATCTCTGG
CGAGCACGGTCTCGACAGCAATGGAGTGTATGTACCATTTTCAATCCCTCCTGCTTCCTGTTGAG
CTTGTAGGCTGAC-
TCGATGGCCATTTAGCTACAACGGTACCTCCGAGCTTCAGCTCGAGCGTATGAGCGTCTACTTC
AACGAGGCTTCTGGCAACAAGTACGTTCCTCGTGCCGTCCTCGTCGATCTCGAGCCCGGTACCA
TGGATGCCGTCCGCGCTGGTCCCTTCGGCCAGCTCTTCCGCCCTGACAACTTCGTCTTCGGTCA
GTCCGGTGCTGGCAACAACTGG
>N_hydeana_MFLUCC_20_0132
CATTATAGAGTTTTCTAAACTCCCAACCCATGTGAACTTACCTTTTGTTGCCTCGGCAGAAGTTAT
AGGTCTTCTTATAGCTGCTGCCGGTGGACCATTAAACTCTTGTTATTTTATGTAATCTGAGCGTCT
TATTTTAATAAGTCAAAACTTTCAACAACGGATCTCTTGGTTCTGGCATCGATGAAGAACGCAGC
GAAATGCGATAAGTAATGTGAATTGCAGAATTCAGTGAATCATCGAATCTTTGAACGCACATTG
CGCCCATTAGTATTCTAGTGGGCATGCCTGTTCGAGCGTCATTTCAACCCTTAAGCCTAGCTTAG
TGTTGGGAATCTACTTCTTTA--
TAGTTGTAGTTCCTGAAATACAACGGCGGATTTGTAGTATCCTCTGAGCGTAGTAA-
TTTTTTTCTCGCTTTTGTTAGGTGCTATAACTCCCACCCGCTAAACCCCCAATTTTTTGTGGATGAC
CTCGGATCCGGT????????????TATAATTGTTTTTCGTCTG--------ATTCTCCTTCAC-
TTCAGCGTCATGATTTTCAACCTACGTGTTGAAAATTA-TTTTCGCTCCTTCCACAC--TTTTT--
TCGCTGGTTACCCCGCCGCGAGGCACCCGCACGACCCCGCGGTGCAAACGAAAAATTTCTTAT
CACAGCCCCACCTTGCATAAGCAACCATGCATTGCTCATGAGATCCACTT--
TGAACAATTGCTAATGCCTTCATACAGGAAGCCGCCGAGCTCGGTAAGGGTTCCTTCAAGTACG
CCTGGGTTCTTGACAAGCTCAAGGCCGAGCGTGAGCGTGGTATCACCATCGATATCGCTCTCTG
GAAGTTCGAGACCAACGAGTACAATGTCACCGTCATTGGTTAGTACCCCTCCACCTGTGCCATG
TGCTGCTCCATAAGACACTTGACTAACCTTGCTTCATAGACGCTCCCGGTCACCGTGATTTCATC
AAG????TGCTGCTTTCTGGTATGTTACCTGTCTGCCTCGACACGGCCTC-
ATACGACGTTTTTCGTGCCTGCACGACAGCCCCGAACAG-
TGAATTAGGTCAAGATAGAGGGAACATGATGCTAATAGGTCATTGATAGGCAAACCATCTCTGG
CGAGCACGGTCTCGACAGCAATGGAGTGTATGTACTATTTTCAATTCCTCCTGCTTCCTGTTGAG
CTTGTAGGCTGAC-
TCGATGACCATTTAGCTACAACGGTACCTCCGAGCTCCAGCTCGAGCGTATGAGCGTCTACTTC
AACGAGGCTTCCGGCAACAAGTACGTTCCTCGTGCCGTCCTCGTCGATCTCGAGCCCGGTACCA
TGGATGCCGTCCGCGCCGGTCCCTTCGGCCAGCTCTTCCGCCCTGACAACTTCGTCTTCGGTCA
GTCCGGTGCTGGCAACAACTGG
>N_iberica_CBS_147688
CATTATAGAGTTTTCTAAACTCCCAACCCATGTGAACTTACCTTTTGTTGCCTCGGCAGAAGTTAT
AGGTCTTCTTATAGCTGCTGCCGGTGGACCATTAAACTCTTGTTATTTTATGTAATCTGAGCGTCT

TATTTTAATAAGTCAAAACTTTCAACAACGGATCTCTTGGTTCTGGCATCGATGAAGAACGCAGC
GAAATGCGATAAGTAATGTGAATTGCAGAATTCAGTGAATCATCGAATCTTTGAACGCACATTG
CGCCCATTAGTATTCTAGTGGGCATGCCTGTTCGAGCGTCATTTCAACCCTTAAGCCTAGCTTAG
TGTTGGGAATCTACTTCTTTTATTAGTTGTAGTTCCTGAAATACAACGGCGGATTTGTAGTATCCT
CTGAGCGTAGTAA-
TTTTTTTCTCGCTTTTGTTAGGTGCTATAACTCCCAGCCGCTAAACCCCCAATTTTTTGTGGTTGAC
CTCGGATCAGGT????????????????????ATTCCCGTCAT-------CATTCTCCTTCAC-
TTCAGCGTCATGATTTTCAACCTACGTGTTGAAAATTA-TTTTCGCTCCTTCCACAC--TTTTT--
TCGCTGGTTACCCCGCCGCGAGGCACCAGCACGACCCCGCGGTGCAAACGAAAAATTTCTTAT
CACAGCCCCACCTTGCATAAGCAACCATGCATTGCTCATGAGATCCACTT--
TGAACAATTGCTAATGCCTTCATACAGGAAGCCGCCGAGCTCGGTAAGGGTTCCTTCAAGTACG
CCTGGGTTCTTGACAAGCTCAAGGCCGAGCGTGAGCGTGGTATCACCATCGATATCGCTCTCTG
GAAGTTCGAGACCAACGAGTACAATGTCACCGTCATTGGTTAGTACCACTCCACCTATGCCATG
TGCTGCTCCATAAGACACTTGACTAACCTTGCTTCGTAGACGCTCCCGGTCACCGTGATTTCATC
AAGAACA???????????????????AACTGTCTGCCTCGACACGGCCTTGATACGACGTTTTTCGTGCC
TGCACGACGGCCCCGAACAG-
TGAATTAGGTCAAGATAGAGGGAACATGATGCTAATAGGTCATTGATAGGCAAACCATCTCTGG
CGAGCACGGTCTCGACAGCAATGGAGTGTATGTACTATTTTCAATTCCTCCTGCTTCCTGTTGAG
CTTGTAGGCTGAC-
TCGATGGCCATTTAGCTACAACGGTACCTCCGAGCTCCAGCTCGAGCGTATGAGCGTCTACTTC
AACGAGGCTTCCGGTAACAAGTACGTTCCTCGTGCCGTCCTCGTCGATCTCGAGCCCGGTACCA
TGGATGCCGTCCGCGCCGGTCCCTTCGGCCAGCTCTTCCGCCCTGACAACTTCGTCTTCGGTCA
GTCCGGTGCTGGCAACAACTGG
>N_iraniensis_CBS_137768
????????????????????????????????????CTTACCTTTTGTTGCCTCGGCAGAAGTTACAGGTTACCC
TGTAGCTGCTGCCGGCGGACCATTAAACTCTTGTTATTTTATGTAATCTGAGCGTCTTATTTTAAT
AAGTCAAAACTTTCAACAACGGATCTCTTGGTTCTGGCATCGATGAAGAACGCAGCGAAATGCG
ATAAGTAATGTGAATTGCAGAATTCAGTGAATCATCGAATCTTTGAACGCACATTGCGCCCATTA
GTATTCTAGTGGGCATGCCTGTTCGAGCGTCATTTCAACCCTTAAGCCTAGCTTAGTGTTGGGAA
TCTACCTCTTTCTTTTATTGTAGTTCCTGAAATACAACGGCGGATTTGTAGTATCCTCTGAGCGTA
GTAA-
ATCTTTTCTCGCTTTTGTCAGGTGCTGCGACTCCCAGCCGCTAAACCCCCAATTTTTTGTGGTTGA
CCTCGGATCAGGTAGAAGGTTAGTCATCTATTGATTCCCATCAT-------
CAGTCCGGTGCACAACTGGCATGAT--
TTTTCAGCCTGCGTGTTGAAAAAGATTTTTGGCACCTTACACTC--TTTTT--
TCGCTCGTTACCCCGCCGCAAGACACCTGCACGACCCCGCGGTGCAAACGAAAAATTTCTTATC
ACGGCCCCACCTTGCACGAGCAACCATGCATTGCTTATGAGACCCACTT--
TGAACATTAGCTAATGCCTTCACACAGGAAGCCGCCGAGCTCGGTAAGGGTTCCTTCAAGTACG
CCTGGGTTCTTGACAAGCTCAAGGCCGAGCGTGAGCGTGGTATCACCATCGATATCGCTCTCTG
GAAGTTCGAGACCAACGAGTACAATGTCACCGTCATTGGTTAGTACCCTACCATCTATGCCATG
TGCTGCTCAGTTAGACAGTT-ACTAACAATACT---
CAGACGCTCCCGGTCACCGTGATTTCATCAAGAACATGCTGCCTTCTGGTATGTTACCTGTCTGC
CTCGACACGGCCTTACTACGACGTTTTTCGTGCCTGCACGACGGCCCCGAACAG-
TGAAATAGGTCAAGATAGAGGGAACATAATGCTAATAGGTCATTTATAGGCAAACCATCTCTGG

CGAGCACGGTCTCGACAGCAATGGAGTGTATGTACCATTCTCAATTCGTCCTGCTTGTTGTT---
CTCGTAGGCTGAC-
TTGATGGCCATTTAGCTACAACGGTACCTCCGAGCTCCAGCTCGAGCGTATGAGCGTTTATTTCA
ACGAGGCTTCCGGCAACAAGTACGTTCCTCGTGCCGTCCTCGTCGATCTCGAGCCCGGTACCAT
GGATGCCGTCCGCGCCGGTCCCTTCGGCCAGCTCTTCCGCCCTGACAACTTCGTCTTCGGTCAG
TCCGGTGCCGGCAACAACTGG
>N_javaensis_CBS_257_31
CATTATAGAGTTTTCTAAACTCCCAACCCATGTGAACTTACCTTTTGTTGCCTCGGCAGAAGTTAT
AGGTCTTCTTATAGCTGCTGCCGGTGGACCATTAAACTCTTGTTATTTTATGTAATCTGAGCGTCT
TATTTTAATAAGTCAAAACTTTCAACAACGGATCTCTTGGTTCTGGCATCGATGAAGAACGCAGC
GAAATGCGATAAGTAATGTGAATTGCAGAATTCAGTGAATCATCGAATCTTTGAACGCACATTG
CGCCCATTAGTATTCTAGTGGGCATGCCTGTTCGAGCGTCATTTCAACCCTTAAGCCTAGCTTAG
TGTTGGGAATCTACTTCTTTTATTAGTTGTAGTTCCTGAAATACAACGGCGGATTTGTAGTATCCT
CTGAGCGTAGTAA-
TTTTTTTCTCGCTTTTGTTAGGTGCTATAACTCCCAGCCGCTAAACCCCCAATTTTTTGTGGTTGAC
CTCGGATCAGGT?GAAGGTTAGTCATTTATTGATTCCCATCAT----------CCCCCTTCAC-
TTCAGCATCATAATTTTCAACCTACGTGTTGAAAATTA-TTTTCGCTCCTTCCACAC-TTTTTT--
TCGCTGGTTACCCCGCCGCGAGGCACCCGCACGACCCCGCGGTGCAAACGAAAAATTTCTTAT
CACAGCCCCACCTTGCACAAGCAACCATGCATTGCTCATGAGACCCACTT--
TGAACAATTGCTAATGCCTTCATACAGGAAGCCGCCGAGCTCGGTAAGGGTTCCTTCAAGTACG
CCTGGGTTCTTGACAAGCTCAAGGCCGAGCGTGAGCGTGGTATCACCATCGATATCGCTCTCTG
GAAGTTCGAGACCAACGAGTACAATGTCACCGTCATTGGTTAGTACCCCTCCACCTATGCCATG
TGCTGCTCCATAAGACACTTGACTAACCTTGCTTCATAGACGCTCCCGGTCACCGTGATTTCATC
AAGAACATGCTGCCTTCTGGTATGTAACCTGTCTGTCTCGACACGGCCTCAATACGACGTTTTTC
GTGCCTGCACGACGGCCCCGAACAG-
TGAATTAGGTCAAGATAGAGGGAACATGATGCTAATAGGTCATTGATAGGCAAACCATCTCTGG
CGAGCACGGTCTCGACAGCAATGGAGTGTATGTACTATTTTTAATTCCTCCTGCTTCCTGTTAAG
CTTGTAGGCTGAC-
TCGATGGCCATTTAGCTACAACGGTACCTCCGAGCTCCAGCTCGAGCGTATGAGCGTCTACTTC
AACGAGGCTTCCGGCAACAAGTACGTTCCTCGTGCCGTCCTCGTCGATCTCGAGCCCGGTACCA
TGGATGCCGTCCGCGCCGGTCCCTTCGGCCAGCTCTTCCGCCCTGACAACTTCGTCTTCGGTCA
GTCCGGTGCTGGCAACAA????
>N_keteleeria_MFLUCC_13_0915
CATTATAGAGTTTTCTAAACTCCCAACCCATGTGAACTTACCTTTTGTTGCCTCGGCAGAAGTTAT
AGGTCTTCTTATAGCTGCTGCCGGTGGACCATTAAACTCTTGTTATTTTATGTAATCTGAGCGTCT
TATTTTAATAAGTCAAAACTTTCAACAACGGATCTCTTGGTTCTGGCATCGATGAAGAACGCAGC
GAAATGCGATAAGTAATGTGAATTGCAGAATTCAGTGAATCATCGAATCTTTGAACGCACATTG
CGCCCATTAGTATTCTAGTGGGCATGCCTGTTCGAGCGTCATTTCAACCCTTAAGCCTAGCTTAG
TGTTGGGAATCTACTTCTCTTAGGAGTTGTAGTTCCTGAAATACAACGGCGGATTTGTAGTATCC
TCTGAGCGTAGTAA-
TTTTTTTCTCGCTTTTGTTAGGTGCTATAACTCCCAGCCGCTAAACCCCCAATTTTTTGTGG-
TGACCTC-
GATGCAGT???
???

??
??
??
??TGT--------------------
ACTTGTCTG-CTCGA-ACGGCCTTAATACGACGTTTTTCGTGCCTGCACGACGGCCCCGATCAG-
TGAATTAGGTCAAGATACAGGGAACATGATGCTAATAGGTCATTTATAGGCAAACCATCTCTGG
CGAGCACGGTCTCGACAGCAATGGAGTGTATGTACTATTTTCAATTCCTCCTGCTTCCTGTTGAG
CTTGTAGGCTGAC-
TCGATGGCCATTTAGCTACAACGGTACCTCCGAGCTCCAGCTCGAGCGTATGAGCGTCTACTTC
AACGAGGCTTCCGGCAACAAGTACGTTCCTCGTGCCGTCCTCGTCGATCTCGAGCCCGGTACCA
TGGATGCCGTCCGCGCCGGTCCCTTCGGCCAGCTCTTCCGCCCTGACAACTTCGTCTTCGGTCA
GTCCGGTGCTGGCAACAACTGG
>N_longiappendiculata_MEAN_1315
CATTATAGAGTTTTCTAAACTCCCAACCCATGTGAACTTACCTTTTGTTGCCTCGGCAGAAGTTAT
AGGTCTTCTTATAGCTGCTGCCGGTGGACCATTAAACTCTTGTTATTTTATGTAATCTGAGCGTCT
TATTTTAATAAGTCAAAACTTTCAACAACGGATCTCTTGGTTCTGGCATCGATGAAGAACGCAGC
GAAATGCGATAAGTAATGTGAATTGCAGAATTCAGTGAATCATCGAATCTTTGAACGCACATTG
CGCCCATTAGTATTCTAGTGGGCATGCCTGTTCGAGCGTCATTTCAACCCTTAAGCCTAGCTTAG
TGTTGGGAATCTACTTCTTTTATTAGTTGTAGTTCCTGAAATACAACGGCGGATTTGTAGTATCCT
CTGAGCGTAGTAATTTTTTTTCTCGCTTTTGTTAGGTGCTATAACTCCCAGCCGCTAAACCCCCAA
TTTTTTGTGGTTGACCTCGGATCAGGT?????????????????????GATTCCCATTAT-------
CATTTCCCTTCAC-TGCAGCGTCATCATTTTCAATCTACATGTTGAAAATTA-
TTTTCGCTCCTTCCACAC--TTTTT--
TCGCTGGTTACCCCGCCGCGAGGCACCCGCACGACCCCGCGGTGCAAACGAAAAATTTCTTAT
CACAGCCCCACCTTGCACAAGCAACAATGCATTGCTCATGAGACCCACTT--
TGAACGATTGCTAATGCCTTCATACAGGAAGCCGCCGAGCTCGGTAAGGGTTCCTTCAAGTACG
CCTGGGTTCTTGACAAGCTCAAGGCCGAGCGTGAGCGTGGTATCACCATCGATATCGCTCTCTG
GAAGTTCGAGACCAACGAGTACAATGTCACCGTCATTGGTTAGTACCCCTCCACCTATGCC---
TGCCGCTCCATAAGGCACTTGACTAACTTTGCTTCATAGACGCTCCCGGTCACCGTGATTTCATC
AAGAACA????????????????????????TGGTCTGCCTCGACGCGGCCTTGATACGACGTTTTTCGTGCC
TGCACGACGGCCCCGAACAG-
TGAATTAGGTCAAGATAGAGGGAACATGATGCTAATAGGTCATTGATAGGCAAACCATCTCTGG
CGAGCACGGTCTCGACAGCAATGGAGTGTATGTACTATTTTCAATTCCTCCTGCTTCCTGTTGAG
CTTGTAGGCTGAC-
TCGATGGCCATTTAGCTACAACGGTACCTCCGAGCTCCAGCTCGAGCGTATGAGCGTCTACTTC
AACGAGGCTTCCGGTAACAAGTACGTTCCTCGTGCCGTCCTCGTCGATCTCGAGCCCGGTACCA
TGGATGCCGTCCGCGCCGGTCCCTTCGGCCAGCTCTTCCGCCCTGACAACTTCGTCTTCGGTCA
GTCCGGTGCTGGCAACAACTGG
>N_lusitanica_MEAN_1317
CATTATAGAGTTTTCTAAACTCCCAACCCATGTGAACTTACCTTTTGTTGCCTCGGCAGAAGTTAT
AGGTCTTCTTATAGCTGCTGCCGGTGGACCATTAAACTCTTGTTATTTTATGTAATCTGAGCGTCT
TATTTTAATAAGTCAAAACTTTCAACAACGGATCTCTTGGTTCTGGCATCGATGAAGAACGCAGC
GAAATGCGATAAGTAATGTGAATTGCAGAATTCAGTGAATCATCGAATCTTTGAACGCACATTG
CGCCCATTAGTATTCTAGTGGGCATGCCTGTTCGAGCGTCATTTCAACCCTTAAGCCTAGCTTAG

TGTTGGGAATCTACTTCTTTTATTAGTTGTAGTTCCTGAAATACAACGGCGGATTTGTAGTATCCT
CTGAGCGTAGTAA-
TTTTTTTCTCGCTTTTGTTAGGTGCTATAACTCCCAGCCGCTAAACCCCCAATTTTTTGTGGTTGAC
CTCGGATCAGGT???????????????????????????TCAT-------CATTCCCCTTCAC-
TTCGGCATCATAATTTTCAACCTACGTGTTGAAAATTA-TTTTCGCTCCTTCCACACTTTTTTT--
TCGCTGGTTACCCCGCCGCGAGGCACCCGCACGACCCCGCGGTGCAAATGAAAAATTTCTTATC
ACAGCCCCACTTTGCACAAGCAACCATGCATTGCTCATGGGACCCACTT--
TGAACAATTGCTAATGCCTTCATACAGGAAGCCGCCGAGCTCGGTAAGGGTTCCTTCAAGTACG
CCTGGGTTCTTGACAAGCTCAAGGCCGAGCGTGAGCGTGGTATCACCATCGATATCGCTCTCTG
GAAGTTCGAGACCAACGAGTACAATGTCACCGTCATTGGTCAGTACCCCTCCACCTATGCCATG
TGCTGCTCCATAAGACACTTGACTAACCTTGCTTCATAGACGCTCCCGGTCACCGTGATTTCATC
AAGAACA???TTATAACGACGTTTTTCGTGCCTGCACG
ACGGCCCCGAACAG-
TGAAATAGGTCAAGATAGAGGGAACATGATACTAATAGGTCATTTATAGGCAAACCATCTCTGG
CGAGCACGGTCTCGACAGCAATGGAGTGTATGGACTAACTTCAATTCCTCCAGCTTCCTGTTGA
GTTTGTAGGCTGAC-
TCGATGGCCATTTAGCTACAACGGTACCTCCGAGCTCCAGCTCGAGCGTATGAGCGTCTACTTC
AACGAGGCTTCCGGCAACAAGTACGTTCCTCGTGCCGTCCTCGTCGATCTCGAGCCCGGTACCA
TGGATGCCGTCCGCGCCGGTCCCTTCGGCCAGCTCTTCCGCCCTGACAACTTCGTCTTCGGTCA
GTCCGGTGCTGGCAACAACTGG
>N_lusitanica_MEAN_1320
???????????????TAA--TCCC-
ACCCATGTGAACTTACCTTTTGTTGCCTCGGCAGAAGTTATAGGTCTTCTTATAGCTGCTGCCGG
TGGACCATTAAACTCTTGTTATTTTATGTAATCTGAGCGTCTTATTTTAATAAGTCAAAACTTTCAA
CAACGGATCTCTTGGTTCTGGCATCGATGAAGAACGCAGCGAAATGCGATAAGTAATGTGAATT
GCAGAATTCAGTGAATCATCGAATCTTTGAACGCACATTGCGCCCATTAGTATTCTAGTGGGCAT
GCCTGTTCGAGCGTCATTTCAACCCTTAAGCCTAGCTTAGTGTTGGGAATCTACTTCTTTTATTAG
TTGTAGTTCCTGAAATACAACGGCGGATTTGTAGTATCCTCTGAGCGTAGTAA-
TTTTTTTCTCGCTTTTGTTAGGTGCTATAACTCCCAGCCGCTAAACCCCCAATTTTTTGTGGTTGAC
CTCGGATCAGGTAGAAGGTTAGTCATCTATTGATTCCCATCAT-------CATTCCCCTTCAC-
TTCGGCATCATAATTTTCAACCTACGTGTTGAAAATTA-TTTTCGCTCCTTCCACACTTTTTTT--
TCGCTGGTTACCCCGCCGCGAGGCACCCGCACGACCCCGCGGTGCAAATGAAAAATTTCTTATC
ACAGCCCCACTTTGCACAAGCAACCATGCATTGCTCATGGGACCCACTT--
TGAACAATTGCTAATGCCTTCATACAGGAAGCCGCCGAGCTCGGTAAGGGTTCCTTCAAGTACG
CCTGGGTTCTTGACAAGCTCAAGGCCGAGCGTGAGCGTGGTATCACCATCGATATCGCTCTCTG
GAAGTTCGAGACCAACGAGTACAATGTCACCGTCATTGGTCAGTACCCCTCCACCTATGCCATG
TGCTGCTCCATAAGACACTTGACTAACCTTGCTTCATAGACGCTCCCGGTCACCGTGATTTCATC
AAGAACA???????TTGTTGTA-----
CCTGTCTGCCTCGACACGGCCTTACTACGACGTTTTTCGTGCCTGCACGACGGCCCCGAACAG-
TGAAATAGGTCAAGATAGAGGGAACATGATACTAATAGGTCATTTATAGGCAAACCATCTCTGG
CGAGCACGGTCTCGACAGCAATGGAGTGTATGGACTAACTTCAATTCCTCCAGCTTCCTGTTGA
GTTTGTAGGCTGAC-
TCGATGGCCATTTAGCTACAACGGTACCTCCGAGCTCCAGCTCGAGCGTATGAGCGTCTACTTC
AACGAGGCTTCCGGCAACAAGTACGTTCCTCGTGCCGTCCTCGTCGATCTCGAGCCCGGTACCA

TGGATGCCGTCCGCGCCGGTCCCTTCGGCCAGCTCTTCCGCCCTGACAACTTCGTCTTCGGTCA
GTCCGGTGCTGGCAACAACTGG
>N_macadamiae_BRIP_63737c
??????????????CCAA--
TCCCAACCCATGTGAACTTACCTTTTGTTGCCTCGGCAGAAGTTATAGGTCTTCTTATAGCTGCTG
CCGGTGGACCATTAAACTCTTGTTATTTTATGTAATCTGAGCGTCTTATTTTAATAAGTCAAAACT
TTCAACAACGGATCTCTTGGTTCTGGCATCGATGAAGAACGCAGCGAAATGCGATAAGTAATGT
GAATTGCAGAATTCAGTGAATCATCGAATCTTTGAACGCACATTGCGCCCATTAGTATTCTAGTG
GGCATGCCTGTTCGAGCGTCATTTCAACCCTTAAGCCTAGCTTAGTGTTGGGAATCTACTTCTCT
TAGGAGTTGTAGTTCCTGAAATACAACGGCGGATTTGTAGTATCCTCTGAGCGTAGTAA-
TTTTTTTCTCGCTTTTGTTAGGGGGTATAACTCCCAGCCGCTAAACCCCCAATTTTTGTGGGTGA
CCTCGGATCAGG??
???????????AAAATTA-TTTTCGCTCCTTCCACAC--TTTTT--
TCGCTGGTTACCCCGCCGCGAGGCACCCGCACGACCCCGCGGTGCAAACGAAAAATTTCTTAT
CACAGCCCCACCTTGCATAAGCAACCATGCTTTGCTCATGAGATCCACTT--
TGAACAATTGCTAATGCCTTCATACAGGAAGCCGCCGAGCTCGGTAAGGGTTCCTTCAAGTACC
TGTG???
???
??ACACGACCTTAATACGACGTTTTTC
GTGCCTGCACGACGGCCCCGAACAG-
TGAAATAGGTCAAGATAGAGGGAACATAATACTAATAGGTCATTTATAGGCAAACCATCTCTGG
CGAGCACGGTCTCGACAGCAATGGAGTGTATGTACTAACTTCAATTCCTCCTGCTTCCTGTTGAG
CTTGTAGGCTGAC-
TTGATGGCCATTTAGCTACAACGGTACCTCCGAGCTCCAGCTCGAGCGTATGAGCGTCTACTTC
AACGAGGCTTCCGGCAACAAGTACGTTCCTCGTGCCGTCCTCGTCGATCTCGAGCCCGGTACCA
TGGATGCCGTCCGCGCCGGTCCCTTCGGCCAGCTCTTCCGCCCTGACAACTTCGTCTTCGGTCA
GTCCGGTGCTGGCAACAACTGG
>N_maddoxii_BRIP_72266a
CATTATAGAGTTTTCTAAACTCCCAACCCATGTGAACTTACCTTTTGTTGCCTCGGCAGAAGTTAT
AGGATTTCTTATAGCTGCTGCCGGTGGACCATTAAACTCTTGTTATTTTATGTAATCTGAGCGTCT
TATTTTAATAAGTCAAAACTTTCAACAACGGATCTCTTGGTTCTGGCATCGATGAAGAACGCAGC
GAAATGCGATAAGTAATGTGAATTGCAGAATTCAGTGAATCATCGAATCTTTGAACGCACATTG
CGCCCATTAGTATTCTAGTGGGCATGCCTGTTCGAGCGTCATTTCAACCCTTAAGCCTAGCTTAG
TGTTGGGAATCTACTTCTTTA--
TAGTTGTAGTTCCTGAAATACAACGGCGGATTTGTAGTATCCTCTGAGCGTAGTAA-
TTTTTTTCTCGCTTTTGTTAGGTGCTATAACTCCCAGCCGCTAAACCCCCAATTTTTGTGGTTGAC
CTCGGATCAGGTAGAAGGTTAGTCATTTATTGATTCCCATCAT-------CATCCCCCTTCAC-
TTCAGCATCATAATTTTCAACCTACGTGTTGAAAATTA-TTTTCGCTCCTTCCACAC--TTTTT--
TCGCTGGTTACCCCGCCGCGAGGCACCCGCACGACCCCGCGGTGCAAACGAAAAATTTCTTAT
CACAGCCCCACCTTGCACAAGCAACCATGCATTGCTCATGAGACCCACTT--
TGAACAATTGCTAATGCCTTCATACAGGAAGCCGCCGAGCTCGGTAAGGGTTCCTTCAAGTACG
CCTGGGTTCTTGACAAGCTCAAGGCCGAGCGTGAGCGTGGTATCACCATCGATATCGCTCTCTG
GAAGTTCGAGACCAACGAGTACAATGTCACCGTCATTGGTTAGTACCCCTCCACCTATGCTATG
TGCTGCTCCATAAGACACTTGACTAACTTTGCTTCATAGACGCTCCCGGTCACCGTGATTTCATC

AAGAACA????GCTTTCTGGTATGTAACCTGTCTGTCTCGACACGGCCTCAATACGACGTTTTTCG
TGCCTGCACGACGGCCCCGAACAG-
TGAATTAGGTCAAGATAGAGGGAACATGATGCTAATAGGTCATCGATAGGCAAACCATCTCTGG
CGAGCACGGTCTCGACAGCAATGGAGTGTATGTACTATTTTTAATTCCTCCTGCTTCCTGTTAAG
CTTGTAGGCTGAC-
TCGATGGCCATTTAGCTACAACGGTACCTCCGAGCTCCAGCTCGAGCGTATGAGCGTCTACTTC
AACGAGGCTTCCGGCAACAAGTACGTTCCTCGTGCCGTCCTCGTCGATCTCGAGCCCGGTACCA
TGGATGCCGTCCGCGCCGGTCCCTTCGGCCAGCTCTTCCGCCCTGACAACTTCGTCTTCGGTCA
GTCCGGTGCTGGCAACAACTGG
>N_magna_MFLUCC_12_0652
CATTATAGAGTTTTCTAAACTCCCAACCCATGTGAACTTACCTTTTGTTGCCTCGGCAGAAGCTAT
AGGACTTCTTATAGCTGCTGCCGGTGGACCACTAAACTCTTGTTATTTTATGGAATCTGAGCGTC
TTATTTTAATAAGTCAAAACTTTCAACAACGGATCTCTTGGTTCTGGCATCGATGAAGAACGCAG
CGAAATGCGATAAGTAATGTGAATTGCAGAATTCAGTGAATCATCGAATCTTTGAACGCACATT
GCGCCCATTAGTATTCTAGTGGGCATGCCTGTTCGAGCGTCATTTCAACCCTTAAGCCTAGCTTA
GTGTTGGGAATCTACTTCTTCTAGGAGTCGTAGTTCCTGAAATACAACGGCGGATTTATAGTGTC
CTCTGAGCGTAGTAA-
TTTTTTTCTCGCTTTTGTCAGGTGCTGTAACTCCCAGCCGCTAAACCCCTAATTTTTTGTGGTTGAC
CTCGGATCAGGTAGAAGGTTAGTC---TATTGATTACCATCAC-------CATTCCTTTTCAA-
TTCAGGGTCATGATTTTCAACC---ACGTTGAAAATTA-TTTTCGCTCCTTCCACACTTTTTTT--
CCGCTGGTTACCCCGCCGCGAGGCACCCGCACGACCCCGCGGTGCAAACGAAAAATTTCTTAT
CACAGCCCCACCTTGCACAAGCAACCATGCATTGCTCATGAGACCCACTT--
TGAATAATTGCTAATGCCTTGATACAGGAAGCTGCCGAGCTCGGTAAGGGTTCCTTCAAGTACG
CCTGGGTTCTTGACAAGCTCAAGGCCGAGCGTGAGCGTGGTATCACCATCGATATCGCTCTCTG
GAAGTTCGAGACCAACGAGTACAATGTCACCGTCATTGGTTAGTACCTCTCCACCTATGTCATGT
TCTGCACCATAAGACACTTGACTAACCTTGCTTTATAGACGCTCCCGGTCACCGTGATTTCATCA
AGAACATGCTGCCTTCTGGTATGTAACCTGTCTGCCTCGACACGGCCTTGATACGACGTTTTTG
TGCCTTCACGACGGCCCCGAACAG-
GGAATTAGGTCAAGATACAGGGAACATAATGCTAATAGGTCATTTATAGGCAAACCATCTCTGG
CGAGCACGGTCTCGACAGCAATGGAGTGTATGTACTATTTTCAATTCCTCCTGCTTCCTGCTGAG
CTTGTAGGCTGAC-
TCGATGGCCATTTAGCTACAACGGTACCTCCGAGCTCCAGCTCGAGCGTATGAGCGTCTACTTC
AACGAGGCTACCGGCAACAAGTATGTTCCTCGTGCCGTCCTCGTTGATCTCGAGCCCGGTACCA
TGGATGCCGTCCGCTCTCATCCGTG??
??????
>N_mesopotamica_CBS_336_86
CATTATAGAGTTTTCTAAACTCCCAACCCATGTGAACTTACCTTTTGTTGCCTCGGCAGAAGTTAT
AGGTCTTCTTATAGCTGCTGCCGGTGGACCATTAAACTCTTGTTATTTTATGTAATCTGAGCGTCT
TATTTTAATAAGTCAAAACTTTCAACAACGGATCTCTTGGTTCTGGCATCGATGAAGAACGCAGC
GAAATGCGATAAGTAATGTGAATTGCAGAATTCAGTGAATCATCGAATCTTTGAACGCACATTG
CGCCCATTAGTATTCTAGTGGGCATGCCTGTTCGAGCGTCATTTCAACCCTTAAGCCTAGCTTAG
TGTTGGGAATCTACTTCTTTTATTAGTTGTAGTTCCTGAAATACAACGGCGGATTTGTAGTATCCT
CTGAGCGTAGTAATTTCTTTTCTCGCTTTTGTTAGGTGCTATAACTCCCAGCCGCTAAACCCCCAA
TTTTTTGTGGTTGACCTCGGATCAGGT???????TAGTCATTTATTGATTCCCGTCAT-------

CATCCCCCTTCAC-TTCAGCATCATAATTTTCAACCTACGTGTTGAAAATTA-
TTTTCGCTCCTTCCACAC--TTTTT--
TCGCTGGTTACCCCGCCGCGAGGCACCCGCACGACCCCGCGGTGCGAACGAAAAATTTCTTAT
CACAGCCCCACCTTGCACAAGCAACCATGCATTGCTCATGAGACCCACTT--
TGAACAATTGCTAATGCCTTCATACAGGAAGCCGCCGAGCTCGGTAAGGGTTCCTTCAAGTACG
CCTGGGTTCTCGACAAGCTCAAGGCCGAGCGTGAGCGTGGTATCACCATCGATATCGCTCTCTG
GAAGTTCGAGACCAACGAGTACAATGTCACCGTCATTGGTTAGTACCCCTCCACCTATGCCATG
TGCTGCTCCATAAGACACTTGACTAACCCTGCTTCATAGACGCTCCCGGTCACCGTGATTTCATC
AAGA???TGCTGCCTTCTGGTATGTAACCTGTCTGTCTCGACACGGCCTCAATACGACGTTTTTCG
TGCCTGCACGACAGCCCCGAACAG-
TGAATTAGGTCAAGATAGAGGGAACATGATGCTAATAGGTCATTGATAGGCAAACCATCTCTGG
CGAGCACGGTCTCGACAGCAATGGAGTGTATGTACTATTTTTAATTCCTCCTGCTTCCTGTTGAG
CTTGTAGGCTGAC-
TCGATGGCCATTTAGCTACAACGGTACCTCCGAGCTCCAGCTCGAGCGTATGAGCGTCTACTTC
AACGAGGCTTCCGGCAACAAGTACGTTCCTCGTGCCGTCCTCGTCGATCTCGAGCCCGGTACCA
TGGATGCCGTCCGCGCCGGTCCCTTCGGCCAGCTCTTCCGCCCTGACAACTTCGTCTTCGGTCA
G?????????????????????
>N_musae_MFLUCC_15_0776
CATTATAGAGTTTTCTAAACTCCCAACCCATGTGAACTTACCTTTTGTTGCCTCGGCAGAAGTTAT
AGGTCTTCTTATAGCTGCTGCCGGTGGACTATTAAACTCTTGTTATTTTATGTAATCTGAGCGTCT
TATTTTAATAAGTCAAAACTTTCAACAACGGATCTCTTGGTTCTGGCATCGATGAAGAACGCAGC
GAAATGCGATAAGTAATGTGAATTGCAGAATTCAGTGAATCATCGAATCTTTGAACGCACATTG
CGCCCATTAGTATTCTAGTGGGCATGCCTGTTCGAGCGTCATTTCAACCCTTAAGCCTAGCTTAG
TGTTGGGAATCTACTTCTTTCATTAGTCGTAGTTCCTGAAATACAACGGCGGATTTGTGGTATCCT
CTGAGCGTAGTAATTTTTTTCCTCGCTTTTGTTAGGTGCTATGACTCCCAGCCGCTAAACCCCCA
ATTTTTTGTGG-TGACCTCGGATCAGGT???????????????????????????CATCAT--------
CATTCCCTTCAC-TTCAGCATCATAATTTTCAACCTACGTGTTGAAAATTA-
TTTTCGCTCCTTCCACAC--CTTTT--
TCGCTGGTTACCCCGCCGCGAGGCACCCGCACGACCCCGCGGTGCAAACGAAAAATTTCTTAT
CACAGCCCCACCTTGCATAAGCAACCATGCATTGCTCATGAGATCCACTT--
TGAACAATTGCTAATGCCTTCATACAGGAAGCCGCCGAGCTCGGTAAGGGTTCCTTCAAGTACG
CCTGGGTTCTTGACAAGCTCAAGGCCGAGCGTGAGCGTGGTATCACCATCGATATCGCTCTCTG
GAAGTTCGAGACCAACGAGTACAATGTCACCGTCATTGGTTAGTACCCCTCCACCTATGCCATG
TGCTGCTCCATAAGACATTTGACTAACCTTGCTTCATAGACGCTCCCGGTCACCGTGATTTCATC
AAGAACA?????????????????????????TCTG-CTCGA-
ACGGCCTTGATACGACGTTTTTCGTGCCTGCACGACGGCCCCGAACAG-
TGAATTAGGTCAAGATACAGGGAACATGATGCTAATAGGTCATTTATAGGCAAACCATCTCTGG
CGAGCACGGTCTCGACAGCAATGGAGTGTATGTACCATTTTCAATTCCTTCTGCTTCCTGTTGAG
CTTGTAGGCTGAC-
TCGATGGCCATTTAGCTACAACGGTACCTCCGAGCTTCAGCTCGAGCGTATGAGCGTCTACTTC
AACGAGGCTTCTGGCAACAAGTACGTTCCTCGTGCCGTCCTCGTCGATCTCGAGCCCGGTACCA
TGGATGCCGTCCGCGCCGGTCCCTTCGGCCAGCTCTTCCGCCCTGACAACTTCGTCTTCGGTCA
GTCCGGTGCTGGCAACAACTGG
>N_natalensis_CBS_138_41

CATTATAGAGTTTTCTAAACTCCCAACCCATGTGAACTTACCTTTTGTTGCCTCGGCAGAGGTTA
CGGGTTACCCTGTAGCTGCTGCCGGTGGACCATTAAACTCTTGTTATTTTATGTAATCTGAGCGT
CTTATATTAATAAGTCAAAACTTTCAACAACGGATCTCTTGGTTCTGGCATCGATGAAGAACGCA
GCGAAATGCGATAAGTAATGTGAATTGCAGAATTCAGTGAATCATCGAATCTTTGAACGCACAT
TGCGCCCATTAGTATTCTAGTGGGCATGCCTGTTCGAGCGTCATTTCAACCCTTAAGCCTAGCTT
AGTGTTGGGAATCTACCTCTCTTCGGAGTCGTAGTTCCTGAAATACAACGGCGGATTTGTAGTAT
CCTCTGAGCGTAGTAA-
ATTTTTTCTCGCTTTTGTTAGGTGCTGCAACTCCCAGCCGCTAAACCCCCTATTTTTTTGGTTGAC
CTCGGATCAGGTAGAAGGTTAGTCATCAATCGGTTCCCATCAT----------CTCCCTTCAC-
TCCAGCGTCATGATTTTCAGCCTACGTGTTGAAGATCAATTTTCGCTCCTTCCACACTTTTTTCTC
CCGATCGTTACCCCGCCGTGAGGCACCCGCACGACCCCGCGGTGCAAACGAAAAATTTCTTAT
CACAGCCCCACCTTGCACAAGCCGACATGCATTGCGTACGAGACCCACTT--
TAAACAACTGCTAATGTCTTCATACAGGAAGCCGCCGAGCTCGGTAAGGGTTCCTTCAAGTACG
CCTGGGTTCTTGACAAGCTCAAGGCCGAGCGTGAGCGTGGTATCACCATCGATATCGCTCTCTG
GAAGTTCGAGACCAACGAGTACAATGTCACTGTCATTGGTTAGTACCTTATCACCTATGCCATGT
GCTGCACAATAAGACTCTT-
ACTAATAGTACTCCTCAGACGCTCCCGGTCACCGTGATTTCATCAAGAACATGCTGCCTTCTGGT
ATGTAACCTGCCTACCTCGACCCGGCTTCAATACGACATTTTTCGTGACTTCACGACAGCCTCGA
ACAC-
TTGGTAGGGTCAAGATAGAGAGAACACGATGCTAATTGGTCATTTATAGGCAAACCATCTCTGG
CGAGCACGGTCTCGACAGCAATGGAGTGTATGTACCATTTTAAATTCCTTTTGCTTCTTGTTGAG
CTCGTAGGCTGAC-
TTGATCGCCCTTTAGCTACAACGGTACCTCCGAGCTCCAGCTCGAGCGTATGAGCGTCTACTTC
AACGAGGCTTCCGGCAACAAGTACGTTCCTCGTGCCGTCCTTGTCGATCTCGAGCCCGGTACCA
TGGATGCCGTCCGCGCCGGTCCCTTCGGCCAGCTCTTCCGCCCTGACAACTTCGTCTTCGGTCA
GTCCGGTGCTGGCAACAACTGG
>N_nebuloides_BRIP_66617
CATTATAGAGTTTTCTAAACTCCCAACCCATGTGAACTTACCTTTTGTTGCCTCGGCAGAAGTTAT
AGGTCTTCTTATAGCTGCTGCCGGTGGACCATTAAACTCTTGTTATTTTATGTAATCTGAGCGTCT
TATTTTAATAAGTCAAAACTTTCAACAACGGATCTCTTGGTTCTGGCATCGATGAAGAACGCAGC
GAAATGCGATAAGTAATGTGAATTGCAGAATTCAGTGAATCATCGAATCTTTGAACGCACATTG
CGCCCATTAGTATTCTAGTGGGCATGCCTGTTCGAGCGTCATTTCAACCCTTAAGCCTAGCTTAG
TGTTGGGAATCTACTTCTTTTATTAGTTGTAGTTCCTGAAATACAACGGCGGATTTGTAGTATCCT
CTGAGCGTAGTAA-
TTTTTTTCTCGCTTTTGTTAGGTGCTATAACTCCCAGCCGCTAAACCCCCAATTTTTTGTGGTTGAC
CTCGGATCAGGT?GAAGGTTAGTCATCTACTGATTCCCGTCAT-CATTATCATTCTCCTTCAC-
TTCAGCGTCATGATTTTCAACCTATGTGTTGAAAATTA-TTTTCGCTCCTTCCACAC--TTTTT--
TCGCTGGTTACCCCGCCGCGAGGCACCCGCACGACCCCGCGGTGCAAACGAAAAATTTCTTAT
CACAGCCCCACCTTGCATAAGCAACCATGCATTGCTCATGAGATCCACTT--
CGAACAATTGCTAATGCCTTCATACAGGAAGCCGCCGAGCTCGGTAAGGGTTCCTTCAAGTACG
CCTGGGTTCTTGACAAGCTCAAGGCCGAGCGTGAGCGTGGTATCACCATCGATATCGCTCTCTG
GAAGTTCGAGACCAACGAGTACAATGTCACCGTCATTGGTTAGTACCCCTCCACCTATGCCATG
TGCTGCTCCATAAGACACTTGACTAACCTTGCTTCATAGACGCTCCCGGTCACCGTGATTTCATC
AAGAACATGCTGCCTTCTGGTATGTTACCTGTCTGCCTCGACACGGCCTTACTACGACGTTTTTC

GTGCCTGCACGACGGCCCCGAACAG-
TGAAATAGGTCAAGATAGAGGGAACATGATACTAATAGGTCATTTATAGGCAAACCATCTCTGG
CGAGCACGGTCTCGACAGCAATGGAGTGTATGTACTAACTTCAATTCCTCCTGCTTCCTGTTGAG
TTTGTAGGCTGAC-
TCGATGGCCATTTAGCTACAACGGTACCTCCGAGCTCCAGCTCGAGCGTATGAGCGTCTACTTC
AACGAGGCTTCCGGCAACAAGTACGTTCCTCGTGCCGTCCTCGTCGATCTCGAGCCCGGTACCA
TGGATGCCGTCCGCGCCGGTCCCTTCGGCCAGCTCTTCCGCCCTGACAACTTCGTCTTCGGTCA
GTCCGGTGCTGGCAACAACTGG
>N_olumideae_BRIP_72273a
CATTATAGAGTTTTCTAAACTCCCAACCCATGTGAACTTACCTTTTGTTGCCTCGGCAGAAGTTAT
AGGTCTTCTTATAGCTGCTGCCGGTGGACCATTAAACTCTTGTTATTTTATGTAATCTGAGCGTCT
TATTTTAATAAGTCAAAACTTTCAACAACGGATCTCTTGGTTCTGGCATCGATGAAGAACGCAGC
GAAATGCGATAAGTAATGTGAATTGCAGAATTCAGTGAATCATCGAATCTTTGAACGCACATTG
CGCCCATTAGTATTCTAGTGGGCATGCCTGTTCGAGCGTCATTTCAACCCTTAAGCCTAGCTTAG
TGTTGGGAATCTACTTCTTTTATTAGTTGTAGTTCCTGAAATACAACGGCGGATTTGTAGTATCCT
CTGAGCGTAGTAA-
TTTTTTTCTCGCTTTTGTTAGGTGCTATAACTCCCAGCCGCTAAACCCCCAATTTTTTGTGGTTGAC
CTCGGATCAGGTAGAAGGTCAGTCATTCATTGATTCCCATCAT-------CATCCCCCTTCAC-
TTCAGCATCATAATTTTCAACCTACGTGTTGAAAATTA-TTTTCGCTCCTTCCACAC-TTTTTT--
CCGCTGGTTACCCCGCCGCGAGGCACCCGCACGACCCCGCGGTGCAAACGAAAAATTTCTTAT
CACAGCCCCACCTTGCACAAGCAACCATGCATTGCTCATGAGACCCACTT--
TGAACAATTGCTAATGCCTTCATACAGGAAGCCGCCGAGCTCGGTAAGGGTTCCTTCAAGTACG
CCTGGGTTCTTGACAAGCTCAAGGCCGAGCGTGAGCGTGGTATCACCATCGATATCGCTCTCTG
GAAGTTCGAGACCAACGAGTACAATGTCACCGTCATTGGTTAGTACCCCTCCACCTATGCCATG
TGCTGCTCCATGAGACACTTGACTAACCTTGCTTCATAGACGCTCCCGGTCACCGTGATTTCATC
AAGAACA???TGCTTTCTGGTATGTTACCTGTCTGCCTCGACACGGCCTTACTACGACGTTTTTCG
TGCCTGCACGACGGCCCCGAACAG-
TGAAATAGGTCAAGATAGAGGGAACATGATACTAATAGGTCATTTATAGGCAAACCATCTCTGG
CGAGCACGGTCTCGACAGCAATGGAGTGTATGTACTAACTTCAATTCCTCCTGCTTCCTGTTGAG
TTTGTAGGCTGAC-
TCGATGGCCATTTAGCTACAACGGTACCTCCGAGCTCCAGCTCGAGCGTATGAGCGTCTACTTC
AACGAGGCTTCCGGCAACAAGTACGTTCCTCGTGCCGTCCTCGTCGATCTCGAGCCCGGTACCA
TGGATGCCGTCCGCGCCGGTCCCTTCGGCCAGCTCTTCCGCCCTGACAACTTCGTCTTCGGTCA
GTCCGGTGCTGGCAACAACTGG
>N_pandanicola_KUMCC_17_0175
??
??
??
??
??
??ATTCCCCATCAATCCATTCCCCTCTT-------CA---
------ACTTTCAGCGTCATGATTTTCAACCCACGTGTTGAAAATTA-
TTTTCGCTCCTTCCACACTTTTTTT--
TCGGTGGTTACCCCGCCGCGAGGCACCCGCACGACCCCGCGGTGCAAACGAAAAATTTCTTAT

CACAGCCCCCCCTTGCACAAGCAACCATGCATTGCTCATGAGACCCACTT--
GAACCAATTGCTAATGTCTTCATACAGGAAGCCGCCGAGCTCGGTAAGGGTTCCTTCAAGTACG
CCTGGGTTCTTGACAAGCTCAAGGCCGAGCGTGAGCGTGGTATCACCATCGATATCGCTCTCTG
GAAGTTCGAGACCAACGAGTACAATGTCACCGTCATTGGTTAGTACCCCTCCACCTATGCCATG
TGCTGCTCCATAAGACACTTGACTAACCTTGCTTCATAGACGCTCCCGGTCACCGTGATTTCATC
AAGAACA??????????????AAATAGACTGTCTGTCTCGAC-CGGCCTC-
ATACGACGTTTTTCGTGCCTGCACGACAGCCCCGAACAG-
TGAATTAGGTCAAGATAGAGGGAACATGATGCTAATAGGTCATTGATAGGCAAACCATCTCTGG
CGAGCACGGTCTCGACAGCAATGGAGTGTATGTACTATTTTCAATTCCTCCTGCTTCCTGTTGAG
CTTGTAGGCTGAC-
TCGATGGCCATTTAGCTACAACGGTACCTCCGAGCTCCAGCTCGAGCGTATGAGCGTCTACTTC
AACGAGGCTTCCGGCAACAAGTACGTTCCTCGTGCCGTCCTCGTCGATCTCGAGCCCGGTACCA
TGGATGCCGTCCGCGCCGGTCCCTTCGGCCAGCTCTTCCGCCCTGACAACTTCGTCTTCGGTCA
GTCCGGTGCTGGCAACAACTGG
>N_pernambucana_GS_2014_RV01
CATTATAGAGTTTTCTAAACTCCCAACCCATGTGAACTTACCTTTTGTTGCCTCGGCAGAA-
TTATAGGTCTTCTTATAGCTGCTGCCGGTGGACCATTAAACTCTTGTTATTTTATGTAATCTGAGC
GTCTTATTTTAATAAGTCAAAACTTTCAACAACGGATCTCTTGGTTCTGGCATCGATGAAGAACG
CAGCGAAATGCGATAAGTAATGTGAATTGCAGAATTCAGTGAATCATCGAATCTTTGAACGCAC
ATTGCGCCCATTAGTATTCTAGTGGGCATGCCTGTTCGAGCGTCATTTCAACCCTTAAGCCTAGC
TTAGTGTTGGGAATCTACTTCTTTTATTAGTTGTAGTTCCTGAAATACAACGGCGGATTTGTAGTA
TCCTCTGAGCGTAGTAA-TTTTTTTCTCGCTTTTGTTAGGTGCTATAA-TCCCAGCCG-
TAAACCCCCAA???????????????????????????AGAAGGTTAGTCATTTATTGATTCCCATCAT----
---CATCCCCCTTCAC-CTCAGCATCATAATTTTCAACCTACGTGGTGAAAATTA-
TTTTCGCTCCTTCCACACTTTTTTT--
TCGCTGGTTACCCCGCCGCGAGGCACCCGCACGACCCCGCGGTGCAAACGAAAAATTTCTTAT
CACAGCCCCACCTTGCACAAGCAACCATGCATTGCTCATGAGACCCACTT--
TGAACAATTGCTAATGCCTTCATACAGGAAGCCGCCGAGCTCGGTAAGGGTTCCTTCAAGTACG
CCTGGGTTCTTGACAAGCTCAAGGCCGAGCGTGAGCGTGGTATCACCATCGATATCGCTCTCTG
GAAGTTCGAGACCAACGAGTACAATGTCACCGTCATTGGTTAGTACCCCTCCACCTATGCCATG
TGCTGCTCCATAAGACACTTGACTAACCTTGCTTCATAGACGCTCCCGGTCACCGTGATTTCATC
AAGAACA???
??
??
??
??
??
>N_perukae_FMB0127
??????????????CTAAACTCCCAACCCATGTGAACTTACCTTTTGTTGCCTCGGCAGAAGTTATAGG
TCTTCTTATAGCTGCTGCCGGTGGACCATTAAACTCTTGTTATTTTATGTAATCTGAGCGTCTTATT
TTAATAAGTCAAAACTTTCAACAACGGATCTCTTGGTTCTGGCATCGATGAAGAACGCAGCGAA
ATGCGATAAGTAATGTGAATTGCAGAATTCAGTGAATCATCGAATCTTTGAACGCACATTGCGC
CCATTAGTATTCTAGTGGGCATGCCTGTTCGAGCGTCATTTCAACCCTTAAGCCTAGCTTAGTGT
TGGGAATCTACTTCTTTA--

TAGTTGTAGTTCCTGAAATACAACGGCGGATTTGTAGTATCCTCTGAGCGTAGTAA-
TTTTTTTCTCGCTTTTGTCAGGTGCTATAACTCCCAGCCGCTAAACCCCCAATTTTTTGTGGTTGA
CCTCGGATCAGGT??
???TACGTGTTGAAAATTA-TTTTCGCTCCTTCCACAC--TTTTT--
TCGCTGGTTACCCCGCCGCGAGGCACCCGCACGACCCCGCGGTGCAAACGAAAAATTTCTTAT
CACAGCCCCACCTTGCACAAGCAACCATGCATTGCTCATGAGACCCACTT--
TGAACAATTGCTAATGCCTTCATACAGGAAGCCGCCGAGCTCGGTAAGGGTTCCTTCAAGTACG
CCTGGGTTCTTGACAAGCTCAAGGCCGAGCGTGAGCGTGGTATCACCATCGATATCGCTCTCTG
GAAGTTCGAGACCAACGAGTACAATGTCACCGTCATTGGTTAGTACCCCTCCACCTATGCCATG
TGCTGCACCATAAGACACTTGACTAACCTTGCTTCATAGACGCTCCCGGTCACCGTGATTTCATC
AAG????TGCTGCCTTCTGGTATGTAACCTGTCTGCCTCGACACGGCCTTAATACGACGTTTTTCG
TGCCTGCACGACGGCCCCGAACAG-
TGAATTAGGTCAAGATAGAGGGAACATGATGCTAATAGGTCATTGATAGGCAAACCATCTCTGG
CGAGCACGGTCTCGACAGCAATGGAGTGTATGTACTATTTTTAATTCCTCCTGCTTCCTGTTAAG
CTTGTAGGCTGAC-
TCGATGGCCATTTAGCTACAACGGTACCTCCGAGCTCCAGCTCGAGCGTATGAGCGTCTACTTC
AACGAGGCTTCCGGCAACAAGTACGTTCCTCGTGCCGTCCTCGTTGATCTCGAGCCCGGTACCA
TGGATGCCGTCCGCGCCGGTCCCTTCGGCCAGCTCTTCCGCCCTGACAACTTCGTCTTCGGTCA
GTCCGGTGCTGGCAACAACTGG
>N_petila_MFLUCC_17_1738
CATTATAGAGTTTTCTAAACTCCCAACCCATGTGAACTTACCTTTTGTTGCCTCGGCAGAAGTTAT
AGGTCTTCTTATAGCTGCTGCCGGTGGACCATTAAACTCTTGTTATTTTATGTAATCTGAGCGTCT
TATTTTAATAAGTCAAAACTTTCAACAACGGATCTCTTGGTTCTGGCATCGATGAAGAACGCAGC
GAAATGCGATAAGTAATGTGAATTGCAGAATTCAGTGAATCATCGAATCTTTGAACGCACATTG
CGCCCATTAGTATTCTAGTGGGCATGCCTGTTCGAGCGTCATTTCAACCCTTAAGCCTAGCTTAG
TGTTGGGAATCTACTTCTTTA--
TAGTTGTAGTTCCTGAAATACAACGGCGGATTTGTAGTATCCTCTGAGCGTAGTAA-
TTTTTTTCTCGCTTTTGTTAGGTGCTATAACTCCCAGCCGCTAAACCCCCAATTTTTTGTGGTTGAC
CTCGGATCAGGT??????TTAGTCATTTATTGATTCCCATCAT-------CATCCCCCTTCAC-
TTCAGCATCATAATTTTCAACCTACGTGTTGAAAATTA-TTTTCGCTCCTTCCACAC--TTTTT--
TCGCTGGTTACCCCGCCGCGAGGCACCCGCACGACCCCGCGGTGCAAACGAAAAATTTCTTAT
CACAGCCCCACCTTGCACAAGCAACCATGCATTGCTCATGAGACCCACTT--
TGAACAATTGCTAATGCCTTCATACAGGAAGCCGCCGAGCTCGGTAAGGGTTCCTTCAAGTACG
CCTGGGTTCTTGACAAGCTCAAGGCCGAGCGTGAGCGTGGTATCACCATCGATATCGCTCTCTG
GAAGTTCGAGACCAACGAGTACAATGTCACCGTCATTGGTTAGTACCCCTCCACCTATGCCATG
TGCTGCTCCATAAGACACTTGACTAACCTTGCTTCATAGACGCTCCCGGTCACCGTGATTTCATC
AAG????TGCTGCCTTCTGGTATGTAATCTGTCTGCCTCGACACGGCCTTAATACGACGTTTTTCGT
GCCTGCACGACGGCCCCGATCAG-
TGAATTAGGTCAAGATACAGGGAACATGATGCTAATAGGTCATTTATAGGCAAACCATCTCTGG
CGAGCACGGTCTCGACAGCAATGGAGTGTATGTACTATTTTCAATTCCTCCTGCTTCCTGTTGAG
CTTGTAGGCTGAC-
TCGATGGCCATTTAGCTACAACGGTACCTCCGAGCTCCAGCTCGAGCGTATGAGCGTCTACTTC
AACGAGGCTTCCGGCAACAAGTACGTCCCTCGTGCCGTCCTCGTCGATCTCGAGCCCGGTACC
ATGGATGCCGTCCGCGCCGGTCCCTTCGGCCAGCTCTTCCGCCCTGACAACTTCGTCTTCGGTC

AGTCCGGTGCTGGCAACAACTGG
>N_phangngaensis_MFLUCC_18_0119
ATTTATAGAGTTTTCTAAACTCCCAACCCATGTGAACTTACCTTTTGTTGCCTCGGCAGAAGTTAT
AGGTCTTCTTATAGCTGCTGCCGGTGGACCATTAAACTCTTGTTATTTATGTAATCTGAGCGTCT
TATTTTAATAAGTCAAAACTTTCAACAACGGATCTCTTGGTTCTGGCATCGATGAAGAACGCAGC
GAAATGCGATAAGTAATGTGAATTGCAGAATTCAGTGAATCATCGAATCTTTGAACGCACATTG
CGCCCATTAGTATTCTAGTGGGCATGCCTGTTCGAGCGTCATTTCAACCCTTAAGCCTAGCTTAG
TGTTGGGAATCTACTTCTTTA--
TAGTTGTAGTTCCTGAAATACAACGGCGGATTTGTAGTATCCTCTGAGCGTAGTAA-
TTTTTTTCTCGCTTTTGTTAGGTGCTATAACTCCCAGCCGCTAAACCCCCAATTTTTTGT?????????
?????????AGAAGGTTAGTCATTTATTGATTCCCATCAT-------CATCCCCCTTCAC-
TTCAGCATCATAATTTTCAACCTACGTGTTGAAAATTA-TTTTCGCTCCTTCCACAC--TTTTT--
TCGCTGGTTACCCCGCCGCGAGGCACCCGCACGACCCCGCGGTGCAAACGAAAAATTTCTTAT
CACAGCCCCACCTTGCACAAGCAACCATGCATTGCTCATGAGACCCACTT--
TGAACAATTGCTAATGCCTTCATACAGGAAGCCGCCGAGCTCGGTAAGGGTTCCTTCAAGTACG
CCTGGGTTCTTGACAAGCTCAAGGCCGAGCGTGAGCGTGGTATCACCATCGATATCGCTCTCTG
GAAGTTCGAGACCAACGAGTACAATGTCCCCGTCATTGGTTAGTACCCCTCCACCTATGCCATG
TGCTGCTCCATAAGACACTTGACTAACCTTGTTTCATAGACGCTCC-
GGCCACCGTGATTTCCTCAAGAACA??TTGTTTCCT----------CTG-----CTCGAC-CGGCCTT-
ATACGACGTTTTACGTGCCTGCACGACGGCCCCGATCAG-
TGAATTAGGTCAAGATACAGGGAACATGATGCTAATAGGTCATTTATAGGCAAACCATCTCTGG
CGAGCACGGTCTCGACAGCAATGGAGTGTATGTACTATTTTCAATTCCTCCTGCTTCCTGTTGAG
CTTGTAGGCTGAC-
TCGATGGCCATTTAGCTACAACGGTACCTCCGAGCTCCAGCTCGAGCGTATGAGCGTCTACTTC
AACGAGGCTTCCGGCAACAAGTACGTTCCTCGTGCCGTCCTCGTCGATCTCGAGCCCGGTACCA
TGGATGCCGTCCGCGCCGGTCCCTTCGGCCAGCTCTTCCGCCCTGACAACTTCGTCTTCGGTCA
GTCCGGTGCTGGCAACAACTGG
>N_piceana_CBS_394_48
CATTATAGAGTTTTCTAAACTCCCAACCCATGTGAACTTACCTTTTGTTGCCTCGGCAGGAGTTAT
AGGTCTTCTTATAGCTGCTGCCGGTGGACCATTAAACTCTTGTTATTTATGTAATCTGAGCGTCT
TATTTTAATAAGTCAAAACTTTCAACAACGGATCTCTTGGTTCTGGCATCGATGAAGAACGCAGC
GAAATGCGATAAGTAATGTGAATTGCAGAATTCAGTGAATCATCGAATCTTTGAACGCACATTG
CGCCCATTAGTATTCTAGTGGGCATGCCTGTTCGAGCGTCATTTCAACCCTTAAGCCTAGCTTAG
TGTTGGGAATCTACTTCTTTA--
TAGTTGTAGTTCCTGAAATACAACGGCGGATTTGTAGTATCCTCTGAGCGTAGTAA-
TTTTTTTCTCGCTTTTGTTAGGTGCTATAACTCCCAGCCGCTAAACCCCCAATTTTTTGTGGTTGAC
CTCGGATCAGGTAGAAGGTTAGTCATTTATTGATTCCCATCAT-------CATCCCCCTTCAC-
TTCAGCATCATAATTTTCAACCTACGTGTTGAAAATTA-TTTTCGCTCCTTCCACAC--TTTTG--
TCGCTGGTTACCCCGCCGCGAGGCACCCGCACGACCCCGCGGTGCAAACGAAAAATTTCTTAT
CACAGCCCCACCTTGCACAAGCAACCATGCATTGCTCATGAGACCCACTT--
TGAACAATTGCTAATGCCTTCATACAGGAAGCCGCCGAGCTCGGTAAGGGTTCCTTCAAGTACG
CCTGGGTTCTTGACAAGCTCAAGGCCGAGCGTGAGCGTGGTATCACCATCGATATCGCTCTCTG
GAAGTTCGAGACCAACGAGTACAATGTCACCGTCATTGGTTAGTACCCCTCCACCTATGCCATG
TGCTGCTCCATAAGACACTTGACTAACCTTGCTTCATAGACGCTCCCGGTCACCGTGATTTCATC

AAGAACATGCTGCCTTCTGGTATGTAATCTGTCTGCCTCGACACGGCCTTAATACGACGTTTTTC
GTGCCTGCACGACGGCCCCGATCAG-
TGAATTAGGTCAAGATACAGGGAACATGATGCTAATAGGTCATTTATAGGCAAACCATCTCTGG
CGAGCACGGTCTCGACAGCAATGGAGTGTATGTACTATTTTCAATTCCTCCTGCTTCCTGTTGAG
CTTGTAGGCTGAC-
TCGATGGCCATTTAGCTACAACGGTACCTCCGAGCTCCAGCTCGAGCGTATGAGCGTCTACTTC
AACGAGGCTTCCGGCAACAAGTACGTTCCTCGTGCCGTCCTCGTCGATCTCGAGCCCGGTACCA
TGGATGCCGTCCGCGCCGGTCCCTTCGGCCAGCTCTTCCGCCCTGACAACTTCGTCTTCGGTCA
GTCCGGTGCTGGCAACAACTG?
>N_protearum_CBS_114178
CATTATAGAGTTTTCTAAACTCCCAACCCATGTGAACTTACCTTTTGTTGCCTCGGCAGAAGTTAT
AGGTCTTCTTATAGCTGCTGCCGGTGGACCATTAAACTCTTGTTATTTTATGTAATCTGAGCGTCT
TATTTTAATAAGTCAAAACTTTCAACAACGGATCTCTTGGTTCTGGCATCGATGAAGAACGCAGC
GAAATGCGATAAGTAATGTGAATTGCAGAATTCAGTGAATCATCGAATCTTTGAACGCACATTG
CGCCCATTAGTATTCTAGTGGGCATGCCTGTTCGAGCGTCATTTCAACCCTTAAGCCTAGCTTAG
TGTTGGGAATCTACTTCTCTTAGGAGTTGTAGTTCCTGAAATACAACGGCGGATTTGTAGTATCC
TCTGAGCGTAGTAA-
TTTTTTTCTCGCTTTTGTTAGGTGCTATAACTCCCAGCCGCTAAACCCCCAATTTTCTGTGGTTGA
CCTCGGATCAGGT????????AGTCATTTATTGATTCCCATCAT----------CCCCCTTCAC-
TTCAGCATCATAATTTTCAACCTACGTGTTGAAAATTA-TTTTCGCTCCTTCCACAC-TTTTTT--
TCGCTGGTTACCCCGCCGCGAGGCACCCGCACGACCCCGCGGTGCAAACGAAAAATTTCTTAT
CACAGCCCCACCTTGCACAAGCAACCATGCATTGCTCATGAGACCCACTT--
TGAACAATTGCTAATGCCTTCATACAGGAAGCCGCCGAGCTCGGTAAGGGTTCCTTCAAGTACG
CCTGGGTTCTTGACAAGCTCAAGGCCGAGCGTGAGCGTGGTATCACCATCGATATCGCTCTCTG
GAAGTTCGAGACCAACGAGTACAATGTCACCGTCATTGGTTAGTACCCCTCCACCTATGCCATG
TGCTGCTCCATAAGACACTTGACTAACCTTGCTTCATAGACGCTCCCGGTCACCGTGATTTCATC
AAGAACATGCTGCCTTCTGGTATGTTACCTGTCTGCCTCGACACGGCCTTACTACGACGTTTTTC
GTGCCTGCACGACGGCCCCGAAAAG-
TGAAATAGGTCAAGATAGAGGGAACATGATACTAATAGGTCATTTATAGGCAAACCATCTCTGG
CGAGCACGGTCTCGACAGCAATGGAGTGTATGTACTAACTTCAATTCCTCCTGCTTCCTGTTGAG
TTTGTAGGCTGAC-
TCGATGGCCATTTAGCTACAACGGTACCTCCGAGCTCCAGCTCGAGCGTATGAGCGTCTACTTC
AACGAGGCTTCCGGCAACAAGTACGTTCCTCGTGCCGTCCTCGTCGATCTCGAGCCCGGTACCA
TGGATGCCGTCCGCGCCGGTCCCTTCGGCCAGCTCTTCCGCCCTGACAACTTCGTCTTCGGTCA
GTCCGGTGCTGGCAACAACTGG
>N_psidii_FMB0028
CATTATAGAGTTTTCTAAACTCCCAACCCATGTGAACTTACCTTTTGTTGCCTCGGCAGAAGTTAT
AGGTCTTCTTATAGCTGCTGCCGGTGGACCATTAAACTCTTGTTATTTTATGTAATCTGAGCGTCT
TATTTTAATAAGTCAAAACTTTCAACAACGGATCTCTTGGTTCTGGCATCGATGAAGAACGCAGC
GAAATGCGATAAGTAATGTGAATTGCAGAATTCAGTGAATCATCGAATCTTTGAACGCACATTG
CGCCCATTAGTATTCTAGTGGGCATGCCTGTTCGAGCGTCATTTCAACCCTTAAGCCTAGCTTAG
TGTTGGGAATCTACTTCTTTA--
TAGTTGTAGTTCCTGAAATACAACGGCGGATTTGTAGTATCCTCTGAGCGTAGTAA-
TTTTTTTCTCGCTTTTGTCAGGTGCTATAACTCCCAGCCGCTAAACCCCCAATTTTTTGTGGTTGA

CCTCGGATCAGGT??C-------------------
------TACGTGTTGAAAAATA-TTTTCGCTCCTTCCACAC--TTTTT---
CGCTGGTTACCCCGCCGCGAGGCACCCGCACGACCCCGCGGCGCAAACGAAAAATTTCTTATC
ACAGCCCCACCTTGCATAAGCAACCATGCATTGCTCATGAGATCCACTT--
TGAACAATTGCTAATGCCTTCATACAGGAAGCCGCCGAGCTCGGTAAGGGTTCCTTCAAGTACG
CCTGGGTTCTTGACAAGCTCAAGGCCGAGCGTGAGCGTGGTATCACCATCGATATCGCTCTCTG
GAAGTTCGAGACCAACGAGTACAATGTCACCGTCATTGGTTAGTACCCCTCCACCTATGCCATG
TGCTGCTGCATAAGACACTTGACTAACCTTGCTT??????????????????????????????????????TGCT
GCCTTCTGGTATGTAACCTGTCTGCCTCGACACGGCCTTAATACGACGTTTTTCGTGCCTGCACG
ACGGCCCCGAACAG-
TGAATTAGGTCAAGATAGAGGGAACATGATGCTAATAGGTCATTGATAGGCAAACCATCTCTGG
CGAGCACGGTCTCGACAGCAATGGAGTGTATGTACTATTTTTAATTCCTCCTGCTTCCTGTTAAG
CTTGTAGGCTGAC-
TCGATGGCCATTTAGCTACAACGGTACCTCCGAGCTCCAGCTCGAGCGTATGAGCGTCTACTTC
AACGAGGCTTCCGGCAACAAGTACGTTCCTCGTGCCGTCCTCGTCGATCTCGAGCCCGGTACCA
TGGATGCCGTCCGCGCCGGTCCCTTCGGCCAGCTCTTCCGCCCTGACAACTTCGTCTTCGGTCA
GTCCGGTGCTGGCAACAACTGG
>N_rhapidis_GUCC21501
CATTATAGAGTTTTCTAAACTCCCAACCCATGTGAACTTACCTTTTGTTGCCTCGGCAGAAGTTAT
AGGTCTTCTTATAGCTGCTGCCGGTGGACCATTAAACTCTTGTTATTTTATGTAATCTGAGCGTCT
TATTTTAATAAGTCAAAACTTTCAACAACGGATCTCTTGGTTCTGGCATCGATGAAGAACGCAGC
GAAATGCGATAAGTAATGTGAATTGCAGAATTCAGTGAATCATCGAATCTTTGAACGCACATTG
CGCCCATTAGTATTCTAGTGGGCATGCCTGTTCGAGCGTCATTTCAACCCTTAAGCCTAGCTTAG
TGTTGGGAATCTACTTCTCTTAGGAGTTGTAGTTCCTGAAATACAACGGCGGATTTGTAGTATCC
TCTGAGCGTAGTAA-
TTTTTTTCTCGCTTTTGTTAGGTGCTATAACTCCCAGCCGCTAAACCCCCAATTTTT-
GTGGTTGACCTCGGATCAG??AGAAGGTTAGTCATTTATTGATTCCCATCAT-------
CATCCCCCTTGAC-TTCAGCATCATAATTTTCAACCTACGTGTTGAAAATTA-
TTTTCGCTCCTTCCACAC--TTTTT--
TCGCTGGTTACCCCGCCGCGAGGCACCCGCACGACCCCGCGGTGCAAACGAAAAATTTCTTAT
CACAGCCCCACCTTGCACAAGCAACCATGCATTGCTCATGAGACCCACTT--
TGAACAATTGCTAATGCCTTCATACAGGAAGCCGCCGAGCTCGGTAAGGGTTCCTTCAAGTACG
CCTGGGTTCTTGACAAGCTCAAGGCCGAGCGTGAGCGTGGTATCACCATCGATATCGCTCTCTG
GAAGTTCGAGACCAACGAGTACAATGTCACCGTCATTGGTTAGTACCCCTCCGCCTATGCCATG
TGCTGCTCCATAAGACACTTGACTAACCTTGCTTCATAGACGCTCCCGGTCACCGTGATTTCATC
AAGAACATGCTGCCTTCTGGTATGTAATCTGTCTGCCTCGACACGGCCTTAATACGACGTTTTTC
GTGCCTGCACGACGGCCCCGATCAG-
TGAATTAGGTCAAGATACAGGGAACATGATGCTAATAGGTCATTTATAGGCAAACCATCTCTGG
CGAGCACGGTCTCGACAGCAATGGAGTGTATGTACTATTTTCAATTCCTCCTGCTTCCTGTTGAG
CTTGTAGGCTGAC-
TCGATGGCCATTTAGCTACAACGGTACCTCCGAGCTCCAGCTCGAGCGTATGAGCGTCTACTTC
AACGAGGCTTCCGGCAACAAGTACGTTCCTCGTGCCGTCCTCGTCGATCTCGAGCCCGGTACCA
TGGATGCCGTCCGCGCCGGTCCCTTCGGCCAGCTCTTCCGCCCTGACAACTTCGTCTTCGGTCA
GTCCGGTGCTGGCAACAACTGG

>N_rhizophorae_MFLUCC_17_1550
CATTATAGAGTTTTCTAAACTCCCAACCCATGTGAACTTACCTTTTGTTGCCTCGGCAGAAGTTAT
AGGTCTTCTTATAGCTGCTGCCGGTGGACCATTAAACTCTTGTTATTTTATGTAATCTGAGCGTCT
TATTTTAATAAGTCAAAACTTTCAACAACGGATCTCTTGGTTCTGGCATCGATGAAGAACGCAGC
GAAATGCGATAAGTAATGTGAATTGCAGAATTCAGTGAATCATCGAATCTTTGAACGCACATTG
CGCCCATTAGTATTCTAGTGGGCATGCCTGTTCGAGCGTCATTTCAACCCTTAAGCCTAGCTTAG
TGTTGGGAATCTACTTCTTTA--
TAGTTGTAGTTCCTGAAATACAACGGCGGATTTGTAGTATCCTCTGAGCGTAGTAA-
TTTTTTTCTCGCTTTTGTTAGGTGCTATAACTCCCAGCCGCTAAACCCCCAATTTTTTGTGGTTGAC
CTCGGATCAGGT??????TTAGTCATTTATTGATTCCCATCAT-------CATCCCCCTTCAC-
TTCAG---CATAATTTTCAACCTACGTGTTGAAAATTA-TTTTCGCTCCTTCCACAC--TTTTT--
TCGCTGGTTACCCCGCCGCGAGGCACCCGCACGACCCCGCGGTGCAAACGAAAAATTTCTTAT
CACAGCCCCACCTTGCACAAGCAACCATGCATTGCTCATGAGACCCACTT--
TGAACAATTGCTAATGCCTTCATACAGGAAGCCGCCGAGCTCGGTAAGGGTTCCTTCAAGTACG
CCTGGGTTCTTGACAAGCTCAAGGCCGAGCGTGAGCGTGGTATCACCATCGATATCGCTCTCTG
GAAGTTCGAGACCAACGAGTACAATGTCACCGTCATTGGTTAGTACCCCTCCACCTATGCCATG
TGCTGCACCATGAGACACTTGACTAACCTTGCTTCATAGACGCTCCCGGTCACCGTGATTTCATC
AAG????TGCTGCCTTCTGGTATGTAATCTGTCTGCCTCGACACGGCCTTAATACGACGTTTTTCGT
GCCTGCACGACGGCCCCGATCAG-
TGAATTAGGTCAAGATACAGGGAACATGATGCTAATAGGTCATTTATAGGCAAACCATCTCTGG
CGAGCACGGTCTCGACAGCAATGGAGTGTATGTACTATTTTCAATTCCTCCTGCTTCCTGTTGAG
CTTGTAGGCTGAC-
TCGATGGCCATTTAGCTACAACGGTACCTCCGAGCTCCAGCTCGAGCGTATGAGCGTCTACTTC
AACGAGGCTTCCGGCAACAAGTACGTTCCTCGTGCCGTCCTCGTCGATCTCGAGCCCGGTACCA
TGGATGCCGTCCGCGCCGGTCCCTTCGGCCAGCTCTTCCGCCCTGACAACTTCGTCTTCGGTCA
GTCCGGTGCTGGCAACAACTGG
>N_rhododendri_GUCC_21504
CATTATAGAGTTTTCTAAACTCCCAACCCATGTGAACTTACCTTTTGTTGCCTCGGCAGAAGTTAT
AGGTCTTCTTATAGCTGCTGCCGGTGGACCATTAAACTCTTGTTATTTTATGTAATCTGAGCGTCT
TATTTTAATAAGTCAAAACTTTCAACAACGGATCTCTTGGTTCTGGCATCGATGAAGAACGCAGC
GAAATGCGATAAGTAATGTGAATTGCAGAATTCAGTGAATCATCGAATCTTTGAACGCACATTG
CGCCCATTAGTATTCTAGTGGGCATGCCTGTTCGAGCGTCATTTCAACCCTTAAGCCTAGCTTAG
TGTTGGGAATCTACTTCTCTTAGGAGTTGTAGTTCCTGAAATACAACGGCGGATTTGTAGTATCC
TCTGAGCGTAGTAA-
TTTTTTTCTCGCTTTTGTTAGGTGCTATAACTCCCAGCCGCTAAACCCCCAATTTTTTGTGG-
TGACCTC-GATCAGTAAGAAGGTTAGTCATTTATTGATTCCCATCAT-------
CATCCCCCTTCAC-TTCAGCATCATAATTTTCAACCTACATGTTGGAAATTA-
TTTTCGCTCCTTCCACAC--TTTTT--
TCGCTGGTTACCCCGCCGCGAGGCACCCGCACGACCCCGCGGTGCAAACGAAAAATTTCTTAT
CACAGCCCCACCTTGCACAAGCAACCATGCATTGCTCATGAGACCCACTTTGTGAACAATTGCT
AATGCCTTCATACAGGAAGCCGCCGAGCTCGGTAAGGGTTCCTTCAAGTACGCCTGGGTTCTTG
ACAAGCTCAAGGCCGAGCGTGAGCGTGGTATCACCATCGATATCGCTCTCTGGAAGTTCGAGA
CCAACGAGTACAATGTCACCGTCATTGGTTAGTATCACTCCACCTATGCCATGTGCTGCTCCATA
AGACACTTGACTAACCCTGCTTCACAGACGCTCCCGGTCACCGTGATTTCATCAAGAACATGCT

GCCTTCTGGTATGTTACCTGTCTGCCTCGACACGGCCTTACTACGACGTTTTTCGTGCCTGCACG
ACGGCCCCGAACAG-
TGAAATAGGTCAAGATAGAGGGAACATGATACTAATAGGTCATTTATAGGCAAACCATCTCTGG
CGAGCACGGTCTCGACAGCAATGGAGTGTATGTACTAACTTCAATTCCTCCTGCTTCCTGTTGAG
TTTGTAGGCTGAC-
TCGATGGCCATTTAGCTACAACGGTACCTCCGAGCTCCAGCTCGAGCGTATGAGCGTCTACTTC
AACGAGGCTTCCGGCAACAAGTACGTTCCTCGTGCCGTCCTCGTCGATCTCGAGCCCGGTACCA
TGGATGCCGTCCGCGCCGGTCCCTTCGGCCAGCTCTTCCGCCCTGACAACTTCGTCTTCGGTCA
GTCCGGTGCTGGCAACAACTGG
>N_rhododendricola_KUN_HKAS_123204
CATTATAGAGTTTTCTAAACTCCCAACCCATGTGAACTTACCTTTTGTTGCCTCGGCAGGAGTTAT
AGGTCTTCTTATAGCTGCTGCCGGTGGACCATTAAACTCTTGTTATTTTATGTAATCTGAGCGTCT
TATTTTAATAAGTCAAAACTTTCAACAACGGATCTCTTGGTTCTGGCATCGATGAAGAACGCAGC
GAAATGCGATAAGTAATGTGAATTGCAGAATTCAGTGAATCATCGAATCTTTGAACGCACATTG
CGCCCATTAGTATTCTAGTGGGCATGCCTGTTCGAGCGTCATTTCAACCCTTAAGCCTAGCTTAG
TGTTGGGAATCTACTTCTTTA--
TAGTTGTAGTTCCTGAAATACAACGGCGGATTTGTAGTATCCTCTGAGCGTAGTAA-
TTTTTTTCTCGCTTTTGTTAGGTGCTATAACTCCCAGCCGCTAAACCCCCAATTTTTTGTGGTTGAC
CTCGGATCAGGT????????????ACTTGATGATTC--ATCAT-----------TCCCTTCAC-
TTCAGCATCATAATTTTCAACCTACATGTTGAAAATTA-TTTTCGCTCCTTCCACAC--TTTTT--
TCGCTGGTTACCCCGCCGCGAGGCACCCGCACGACCCCGCGGTGCAAACGAAAAATTTCTTAT
CACAGCCCCACCTTGCACAAGCAACCATGCATTGCTCATGAGACCCACTTTGTGAACAATTGCT
AATGCCTT-
AGATAG???
??
??GTGTGT---
CTGTCTG-CTCGACACGGCCTTACTACGACGTTTTTCGTGCCTGCACGACGGCCCCGAACAG-
TGAAATAGGTCAAGATAGAGGGAACATGATACTAATAGGTCATTTATAGGCAAACCATCTCTGG
CGAGCACGGTCTCGACAGCAATGGAGTGTATGTACTAACTTCAATTCCTCCTGCTTCCTGTTGAG
TTTGTAGGCTGAC-
TCGATGGCCATTTAGCTACAACGGTACCTCCGAGCTCCAGCTCGAGCGTATGAGCGTCTACTTC
AACGAGGCTTCCGGCAACAAGTACGTTCCTCGTGCCGTCCTCGTCGATCTCGAGCCCGGTACCA
TGGATGCCGTCCGCGCCGGTCCCTTCGGCCAGCTCTTCCGCCCTGACAACTTCGTCTTCGGTCA
GTCCGGTGCTGGCAACAACTGG
>N_rosae_CBS_101057
CATTATAGAGTTTTCTAAACTCCCAACCCATGTGAACTTACCTTTTGTTGCCTCGGCAGAAGTTAT
AGGTCTTCTTATAGCTGCTGCCGGTGGACCATTAAACTCTTGTTATTTTATGTAATCTGAGCGTCT
TATTTTAATAAGTCAAAACTTTCAACAACGGATCTCTTGGTTCTGGCATCGATGAAGAACGCAGC
GAAATGCGATAAGTAATGTGAATTGCAGAATTCAGTGAATCATCGAATCTTTGAACGCACATTG
CGCCCATTAGTATTCTAGTGGGCATGCCTGTTCGAGCGTCATTTCAACCCTTAAGCCTAGCTTAG
TGTTGGGAATCTACTTCTTTTATTAGTTGTAGTTCCTGAAATACAACGGCGGATTTGTAGTATCCT
CTGAGCGTAGTAATTTTTTTTCTCGCTTTTGTTAGGTGCTATAACTCCCAGCCGCTAAACCCCCAA
TTTTTTGTGGTTGACCTCGGATCAGGT??AAGGTTAGTCATTTATTGATTCCCGTCAT-------
CATCCCCCTTCAC-TTCAGCATCATAATTTTCAACCTACGTGTTGAAAATTA-

TTTTCGCTCCTTCCACAC--TTTTT--
TCGCTGGTTACCCCGCCGCGAGGCACCCGCACGACCCCGCGGTGCAAACGAAAAATTTCTTAT
CACAGCCCCACCTTGCACAAGCAACCATGCATTGCTCATGAGACCCACTT--
TGAACAATTGCTAATGCCTTCATACAGGAAGCCGCCGAGCTCGGTAAGGGTTCCTTCAAGTACG
CCTGGGTTCTTGACAAGCTCAAGGCCGAGCGTGAGCGTGGTATCACCATCGATATCGCTCTCTG
GAAGTTCGAGACCAACGAGTACAATGTCACCGTCATTGGTTAGTACCCCTCCACCTATGCCATG
TGCTGCTCCATAAGACACTTGACTAACCTTGCTTCATAGACGCTCCCGGTCACCGTGATTTCATC
AAGAACATGCTGCCTTCTGGTATGTAACCTGTCTGTCTCGACACGGCCTCAATACGACGTTTTTC
GTGCCTGCACGACGGCCCCGAACAG-
TGAATTAGGTCAAGATAGAGGGAACATGATGCTAATAGGTCATTGATAGGCAAACCATCTCTGG
CGAGCACGGTCTCGACAGCAATGGAGTGTATGTACTATTTTTAATTCCTCCTGCTTCCTGTTAAG
CTTGTAGGCTGAC-
TCGATGGCCATTTAGCTACAACGGTACCTCCGAGCTCCAGCTCGAGCGTATGAGCGTCTACTTC
AACGAGGCTTCCGGCAACAAGTACGTTCCTCGTGCCGTCCTCGTCGATCTCGAGCCCGGTACCA
TGGATGCCGTCCGCGCCGGTCCCTTCGGCCAGCTCTTCCGCCCTGACAACTTCGTCTTCGGTCA
GTCCGGTGCTGGCAACAAC???
>N_rosicola_CFCC_51992
CATTATAGAGTTTTCTAAACTCCCAACCCATGTGAACTTACCTTTTGTTGCCTCGGCAGAAGTTAT
AGGTCTTCTTATAGCTGCTGCCGGTGGACCATTAAACTCTTGTTATTTTATGTAATCTGAGCGTCT
TATTTTAATAAGTCAAAACTTTCAACAACGGATCTCTTGGTTCTGGCATCGATGAAGAACGCAGC
GAAATGCGATAAGTAATGTGAATTGCAGAATTCAGTGAATCATCGAATCTTTGAACGCACATTG
CGCCCATTAGTATTCTAGTGGGCATGCCTGTTCGAGCGTCATTTCAACCCTTAAGCCTAGCTTAG
TGTTGGGAATCTACTTCTTTTATTAGTTGTAGTTCCTGAAATACAACGGCGGATTTGTAGTATCCT
CTGAGCGTAGTAA-
TTTTTTTCTCGCTTTTGTCAGGTGCTATAACTCCCAGCCGCTAAACCCCCAATTTTTTGTGGTTGA
CCTCGGATCA???AGAAGGTTAGTCATTTATTGATTCCCATCAT-------CATCCCCCTTCAC-
TTCAGCATCATAATTTTCAACCTACGTGTTGAAAATTA-TTTTCGCTCCTTCCACAC--TTTTT--
TCGCTGGTTACCCCGCCGCGAGGCACCCGCACGACCCCGCGGTGCAAACGAAAAATTTCTTAT
CACAGCCCCACCTTGCACAAGCAACCATGCATTGCTCATGAGACCCACTT--
TGAACAATTGCTAATGCCTTCATACAGGAAGCCGCCGAGCTCGGTAAGGGTTCCTTCAAGTACG
CCTGGGTTCTTGACAAGCTCAAGGCCGAGCGTGAGCGTGGTATCACCATCGATATCGCTCTCTG
GAAGTTCGAGACCAACGAGTACAATGTCACCGTCATTGGTTAGTACCCCTCCACCTATGCCATG
TGCTGCACCATAAGACACTTGACTAACCTTGCTTCATAGACGCTCCCGGTCACCGTGATTTCATC
AAGAACATGCTGCCTTCTGGTATGTAATCTGTCTGCCTCGACACGGCCTTAATACGACGTTTTTC
GTGCCTGCACGACGGCCCCGATCAG-
TGAATTAGGTCAAGATACAGGGAACATGATGCTAATAGGTCATTTATAGGCAAACCATCTCTGG
CGAGCACGGTCTCGACAGCAATGGAGTGTATGTACTATTTTCAATTCCTCCTGCTTCCTGTTGAG
CTTGTAGGCTGAC-
TCGATGGCCATTTAGCTACAACGGTACCTCCGAGCTCCAGCTCGAGCGTATGAGCGTCTACTTC
AACGAGGCTTCCGGCAACAAGTACGTTCCTCGTGCCGTCCTCGTCGATCTCGAGCCCGGTACCA
TGGATGCCGTCCGCGCCGGTCCCTTCGGCCAGCTCTTCCGCCCTGACAA?????????????????????
????????????????
>N_samarangensis_MFLUCC_12_0233
CATTATAGAGTTTTCTAAACTCCCAACCCATGTGAACTTACCTTTTGTTGCCTCGGCAGAAGTTAT

AGGTCTTCTTATAGCTGCTGCCGGTGGACCATTAAACTCTTGTTATTTTATGTAATCTGAGCGTCT
TATTTTAATAAGTCAAAACTTTCAACAACGGATCTCTTGGTTCTGGCATCGATGAAGAACGCAGC
GAAATGCGATAAGTAATGTGAATTGCAGAATTCAGTGAATCATCGAATCTTTGAACGCACATTG
CGCCCATTAGTATTCTAGTGGGCATGCCTGTTCGAGCGTCATTTCAACCCTTAAGCCTAGCTTAG
TGTTGGGAATCTACTTCTTTA--
TAGTTGTAGTTCCTGAAATACAACGGCGGATTTGTAGTATCCTCTGAGCGTAGTAA-
TTTTTTTCTCGCTTTTGTTAGGTGCTATAACTCCCAGCCGCTAAACCCCCAATTTTTTGTGGTTGAC
CTCGGATCAGGTAGAAGGTTAGTCATTTATTGATTCCCATCAT-------CATCCCCCTTGAC-
TTCAGCATCATAATTTTCAACCTACGTGTTGAAAATTA-TTTTCGCTCCTTCCACAC--TTTTT--
TCGCTGGTTACCCCGCCGCGAGGCACCCGCACGACCCCGCGGTGCAAACGAAAAATTTCTTAT
CACAGCCCCACCTTGCACAAGCAACCATGCATTGCTCATGAGACCCACTT--
TGAACAATTGCTAATGCCTTCATACAGGAAGCCGCCGAGCTCGGTAAGGGTTCCTTCAAGTACG
CCTGGGTTCTTGACAAGCTCAAGGCCGAGCGTGAGCGTGGTATCACCATCGATATCGCTCTCTG
GAAGTTCGAGACCAACGAGTACAATGTCACCGTCATTGGTTAGTACCCCTCCGCCTATGCCATG
TGCTGCTCCATAAGACACTTGACTAACCTTGCTTCATAGACGCTCCCGGTCACCGTGATTTCATC
AAGAACATGCTGCTTTCTGGTATGTAATCTGTCTGCCTCGACACGGCCTTAATACGACGTTTTTC
GTGCCTGCACGACGGCCCCGATCAG-
TGAATTAGGTCAAGATACAGGGAACATGATGCTAATAGGTCATTTATAGGCAAACCATCTCTGG
CGAGCACGGTCTCGACAGCAATGGAGTGTATGTACTATTTTCAATTCCTCCTGCTTCCTGTTGAG
CTTGTAGGCTGAC-
TCGATGGCCATTTAGCTACAACGGTACCTCCGAGCTCCAGCTCGAGCGTATGAGCGTCTACTTC
AACGAGGCTTCCGGCAACAAGTACGTCCCTCGTGCCGTCCTCGTCGATCTCGAGCCCGGTACC
ATGGATGCCGTCCGCGCCGGTCCCTTCGGCCAGCTCTTCCGCCCTGACAACTTCGTCTTCGGTC
AGTCCGGTGCTGGCAACAACTGG
>N_saprophytica_GUCC_21506
CATTATAGAGTTTTCTAAACTCCCAACCCATGTGAACTTACCTTTTGTTGCCTCGGCAGAAGTTAT
AGGTCTTCTTATAGCTGCTGCCGGTGGACCATTAAACTCTTGTTATTTTATGTAATCTGAGCGTCT
TATTTTAATAAGTCAAAACTTTCAACAACGGATCTCTTGGTTCTGGCATCGATGAAGAACGCAGC
GAAATGCGATAAGTAATGTGAATTGCAGAATTCAGTGAATCATCGAATCTTTGAACGCACATTG
CGCCCATTAGTATTCTAGTGGGCATGCCTGTTCGAGCGTCATTTCAACCCTTAAGCCTAGCTTAG
TGTTGGGAATCTACTTCTCTTAGGAGTTGTAGTTCCTGAAATACAACGGCGGATTTGTAGTATCC
TCTGAGCGTAGTAA-
TTTTTTTCTCGCTTTTGTTAGGTGCTATAACTCCCAGCCGCTAAACCCCCAATTTTTTGTGG-
TGACCTCGGATCAGGTAGAAGGTTAGTCATTTATTGATTCCCATCAT-------
CATCCCCCTTCAC-TTCAGCATCATAATTTTCAACCTACATGTTGAAAATTA-
TTTTCGCTCCTTCCACAC--TTTTT--
TCGCTGGTTACCCCGCCGCGAGGCACCCGCACGACCCCGCGGTGCAAACGAAAAATTTCTTAT
CACAGCCCCACCTTGCACAAGCAACCATGCATTGCTCATGAGACCCACTTTGTGAACAATTGCT
AATGCCTTCATACAGGAAGCCGCCGAGCTCGGTAAGGGTTCCTTCAAGTACGCCTGGGTTCTTG
ACAAGCTCAAGGCCGAGCGTGAGCGTGGTATCACCATCGATATCGCTCTCTGGAAGTTCGAGA
CCAACGAGTACAATGTCACCGTCATTGGTTAGTACCACTCCACCTATGCCATGTGCTGCTCCATA
AGACACTTGACTAACCCTGCTTCACAGACGCTCCCGGTCACCGTGATTTCATCAAGAACATGCT
GCCTTCTGGTATGTAACCTGTCTGTCTCGACACGGCCTCAATACGACGTTTTTCGTGCCTGCACG
ACAGCCCCGAACAG-

TGAATTAGGTCAAGATAGAGGGAACATGATGCTAATAGGTCATTGATAGGCAAACCATCTCTGG
CGAGCACGGTCTCGACAGCAATGGAGTGTATGTACTATTTTCAATTCCTCCTGCTTCCTGTTGAG
CTTGTAGGCTGAC-
TCGATGGCCATTTAGCTACAACGGTACCTCCGAGCTCCAGCTCGAGCGTATGAGTGTCTACTTC
AACGAGGCTTCCGGCAACAAGTACGTTCCTCGTGCCGTCCTCGTCGATCTCGAGCCCGGTACCA
TGGATGCCGTCCGCGCCGGTCCCTTCGGCCAGCTCTTCCGCCCTGACAACTTCGTCTTCGGTCA
GTCCGGTGCTGGCAACAACTGG
>N_saprophytica_MFLUCC_12_0282
CATTATAGAGTTTTCTAAACTCCCAACCCATGTGAACTTACCTTTTGTTGCCTCGGCAGAAGTTAT
AGGTCTTCTTATAGCTGCTGCCGGTGGACCATTAAACTCTTGTTATTTTATGTAATCTGAGCGTCT
TATTTTAATAAGTCAAAACTTTCAACAACGGATCTCTTGGTTCTGGCATCGATGAAGAACGCAGC
GAAATGCGATAAGTAATGTGAATTGCAGAATTCAGTGAATCATCGAATCTTTGAACGCACATTG
CGCCCATTAGTATTCTAGTGGGCATGCCTGTTCGAGCGTCATTTCAACCCTTAAGCCTAGCTTAG
TGTTGGGAATCTACTTCTCTTAGGAGTTGTAGTTCCTGAAATACAACGGCGGATTTGTAGTATCC
TCTGAGCGTAGTAA-
TTTTTTTCTCGCTTTTGTTAGGTGCTATAACTCCCAGCCGCTAAACCCCCAATTTTTTGTGGTTGAC
CTCGGATCAGGTAGAAGGTTAGTCATTTATTGATTCCCATCAT-------CATCCCCCTTCAC-
TTCAGCATCATAATTTTCAACCTACGTGTTGAAAATTA-TTTTCGCTCCTTCCACAC--TTTTT--
TCGCTGGTTACCCCGCCGCGAGGCACCCGCACGACCCCGCGGTGCAAACGAAAAATTTCTTAT
CACAGCCCCACCTTGCACAAGCAACCATGCATTGCTCATGAGACCCACTT--
TGAACAATTGCTAATGCCTTCACACAGGAAGCCGCCGAGCTCGGTAAGGGTTCCTTCAAGTACG
CCTGGGTTCTTGACAAGCTCAAGGCCGAGCGTGAGCGTGGTATCACCATCGATATCGCTCTCTG
GAAGTTCGAGACCAACGAGTACAATGTCACCGTCATTGGTTAGTACCCCTCCACCTATGCCATG
TGCTGCTCCATAAGACCCTTGACTAACCTTGCTTCATAGACGCTCCCGGTCACCGTGATTTCATC
AAGAACATGCTGCCTTCTGGTATGTAACCTGTCTGTCTCGACACGGCCTCAATACGACGTTTTTC
GTGCCTGCACGACAGCCCCGAACAG-
TGAATTAGGTCAAGATAGAGGGAACATGATGCTGATAGGTCATTGATAGGCAAACCATCTCTGG
CGAGCACGGTCTCGACAGCAATGGAGTGTATGTACTATTTTCAATTCCTCCTGCTTCCTGTTGAG
CTTGTAGGCTGAC-
TCGATGGCCATTTAGCTACAACGGTACCTCCGAGCTCCAGCTCGAGCGTATGAGTGTCTACTTC
AACGAGGCTTCCGGCAACAAGTACGTTCCTCGTGCCGTCCTCGTCGATCTCGAGCCCGGTACCA
TGGATGCCGTCCGCGCCGGTCCCTTCGGCCAGCTCTTCCGCCCTGACAACTTCGTCTTCGGTCA
GTCCGGTGCTGGCAACAACTGG
>N_sichuanensis_CFCC_54338
CATTATAGAGTTTTCTAAACTCCCAACCCATGTGAACTTACCTTTTGTTGCCTCGGCAGAAGTTAT
AGGTCTTCTTATAGCTGCTGCCGGTGGACCATTAAACTCTTGTTATTTTATGTAATCTGAGCGTCT
TATTTTAATAAGTCAAAACTTTCAACAACGGATCTCTTGGTTCTGGCATCGATGAAGAACGCAGC
GAAATGCGATAAGTAATGTGAATTGCAGAATTCAGTGAATCATCGAATCTTTGAACGCACATTG
CGCCCATTAGTATTCTAGTGGGCATGCCTGTTCGAGCGTCATTTCAACCCTTAAGCCTAGCTTAG
TGTTGGGAATCTACTTCTCTTAGGAGTTGTAGTTCCTGAAATACAACGGCGGATTTGTAGTATCC
TCTGAGCGTAGTAA-
TTTTTTTCTCGCTTTTGTTAGGTGCTATAACTCCCAGCCGCTAAACCCCCAATTTTTTGTGGTTGAC
CTCGGATCAGGTAGAAGGTTAGTCATCTACTGATTCCCGTCAT-------CATTCTCCTTCAC-
TTCAGCGTCATGATTTTCAACCTACGTGTTGAAAATTA-TTTTCGCTCCTTCCACAC--TTTTT--

TCGCTGGTTACCCCGCCGCGAGGCACCCGCACGACCCCGCGGTGCAAGCGAAAAATTTCTTAT
CACAGCCCCACCTTGCATAAGCAACCATGCATTGCTCATGAGATCCACTT--
TGAACAATTGCTAATGCCTTCATACAGGAAGCCGCCGAGCTCGGTAAGGGTTCCTTCAAGTACG
CCTGGGTTCTTGACAAGCTCAAGGCCGAGCGTGAGCGTGGTATCACCATCGATATCGCTCTCTG
GAAGTTCGAGACCAACGAGTACAATGTCACCGTCATTGGTTAGTACCCCTCCACCTATGTCATG
TGCTGCTCCATAAGACACTTGACTAACCTTGCTTCATAGACGCTCCCGGTCACCGTGATTTCATC
AAGAACATGCTGCCTTCTGGTATGTAATCTGTCTGCCTCGACACGGCCTTAATACGACGTTTTTC
GTGCCTGCACGACGGCCCCGATCAG-
TGAATTAGGTCAAGATACAGGGAACATGATGCTAATAGGTCATTTATAGGCAAACCATCTCTGG
CGAGCACGGTCTCGACAGCAATGGAGTGTATGTACTATTTTCAATTCCTCCTGCTTCCTGTTGAG
CTTGTAGGCTGAC-
TCGATGGCCATTTAGCTACAACGGTACCTCCGAGCTCCAGCTCGAGCGTATGAGCGTCTACTTC
AACGAGGCTTCCGGCAACAAGTACGTTCCTCGTGCCGTCCTCGTCGATCTCGAGCCCGGTACCA
TGGATGCCGTCCGCGCCGGTCCCTTCGGCCAGCTCTTCCGCCCTGACAACTTCGTCTTCGGTCA
GTCCGGTGCTGGCAACAACTGG
>N_sichuanensis_SM15_1C
CATTATAGAGTTTTCTAAACTCCCAACCCATGTGAACTTACCTTTTGTTGCCTCGGCAGAAGTTAT
AGGTCTTCTTATAGCTGCTGCCGGTGGACCATTAAACTCTTGTTATTTTATGTAATCTGAGCGTCT
TATTTTAATAAGTCAAAACTTTCAACAACGGATCTCTTGGTTCTGGCATCGATGAAGAACGCAGC
GAAATGCGATAAGTAATGTGAATTGCAGAATTCAGTGAATCATCGAATCTTTGAACGCACATTG
CGCCCATTAGTATTCTAGTGGGCATGCCTGTTCGAGCGTCATTTCAACCCTTAAGCCTAGCTTAG
TGTTGGGAATCTACTTCTCTTAGGAGTTGTAGTTCCTGAAATACAACGGCGGATTTGTAGTATCC
TCTGAGCGTAGTAA-
TTTTTTTCTCGCTTTTGTTAGGTGCTATAACTCCCAGCCGCTAAACCCCCAATTTTTTGTGGTTGAC
CTCGGATCAGGTAGAAGGTTAGTCATCTACTGATTCCCGTCAT-------CATTCTCCTTCAC-
TTCAGCGTCATGATTTTCAACCTACGTGTTGAAAATTA-TTTTCGCTCCTTCCACAC--TTTTT--
TCGCTGGTTACCCCGCCGCGAGGCACCCGCACGACCCCGCGGTGCAAGCGAAAAATTTCTTAT
CACAGCCCCACCTTGCATAAGCAACCATGCATTGCTCATGAGATCCACTT--
TGAACAATTGCTAATGCCTTCATACAGGAAGCCGCCGAGCTCGGTAAGGGTTCCTTCAAGTACG
CCTGGGTTCTTGACAAGCTCAAGGCCGAGCGTGAGCGTGGTATCACCATCGATATCGCTCTCTG
GAAGTTCGAGACCAACGAGTACAATGTCACCGTCATTGGTTAGTACCCCTCCACCTATGTCATG
TGCTGCTCCATAAGACACTTGACTAACCTTGCTTCATAGACGCTCCCGGTCACCGTGATTTCATC
AAGAACATGCTGCCTTCTGGTATGTAATCTGTCTGCCTCGACACGGCCTTAATACGACGTTTTTC
GTGCCTGCACGACGGCCCCGATCAG-
TGAATTAGGTCAAGATACAGGGAACATGATGCTAATAGGTCATTTATAGGCAAACCATCTCTGG
CGAGCACGGTCTCGACAGCAATGGAGTGTATGTACTATTTTCAATTCCTCCTGCTTCCTGTTGAG
CTTGTAGGCTGAC-
TCGATGGCCATTTAGCTACAACGGTACCTCCGAGCTCCAGCTCGAGCGTATGAGCGTCTACTTC
AACGAGGCTTCCGGCAACAAGTACGTTCCTCGTGCCGTCCTCGTCGATCTCGAGCCCGGTACCA
TGGATGCCGTCCGCGCCGGTCCCTTCGGCCAGCTCTTCCGCCCTGACAACTTCGTCTTCGGTCA
GTCCGGTGCTGGCAACAACTGG
>N_siciliana_CBS_149117
CATTATAGAGTTTTCTAAACTCCCAACCCATGTGAACTTACCTTTTGTTGCCTCGGCAGAAGTTAT
AGGTCTTCTTATAGCTGCTGCCGGTGGACCATTAAACTCTTGTTATTTTATGTAATCTGAGCGTCT

TATTTTAATAAGTCAAAACTTTCAACAACGGATCTCTTGGTTCTGGCATCGATGAAGAACGCAGC
GAAATGCGATAAGTAATGTGAATTGCAGAATTCAGTGAATCATCGAATCTTTGAACGCACATTG
CGCCCATTAGTATTCTAGTGGGCATGCCTGTTCGAGCGTCATTTCAACCCTTAAGCCTAGCTTAG
TGTTGGGAATCTACTTCTTTTATTAGTTGTAGTTCCTGAAATACAACGGCGGATTTGTAGTATCCT
CTGAGCGTAGTAA-
TTTTTTTCTCGCTTTTGTTAGGTGCTATAACTCCCAGCCGCTAAACCCCCAATTTTTTGTGGTTGAC
CTCGGATCAGGT??????TTAGTCATCTGTTGATTCCTATCAT--------CATTCCCTTCAC-
ATCAGCGTCATGATTTTCAACCTACGTGTTGAAAATTA-TTTTCGCTCCTTCCACAC--TTTTT--
TCGCTGGTTACCCCGCCGCGAGGCACCCGCACGACCCCGCGGTGCAAACGAAAAATTTCTTAT
CACAGCCCCACCTTGCACAAGCAACCATGCATTGCTCATGAGACCCACTT--
TGAACAATTGCTAATGCCTTCATACAGGAAGCCGCCGAGCTCGGTAAGGGTTCCTTCAAGTACG
CCTGGGTTCTTGACAAGCTCAAGGCCGAGCGTGAGCGTGGTATCACCATCGATATCGCTCTCTG
GAAGTTCGAGACCAACGAGTACAATGTCACCGTCATTGGTTAGTACCCCTCCACCAATGCCATA
TGCTGCTCCATAAGACACTTGACTAACCTCACTTCATAGACGCTCCCGGTCACCGTGATTTCATC
AAGAACATGCTGCCTTCTGGTATGTAACCTGTCTGCCTCGACACGGCCTTGATACGACGTTTTTC
GTGCCTGCACGACGGCCCCGAACAG-
TGAATTAGGTCAAGATAGAGGGAACATGATGCTAATAGGTCATTGATAGGCAAACCATCTCTGG
CGAGCACGGTCTCGACAGCAATGGAGTGTATGTACTATTTTCAATTCCTCCTGCTTCCTGTTGAG
CTTGTAGGCTGAC-
TCGATGGCCATTTAGCTACAACGGTACCTCCGAGCTCCAGCTCGAGCGTATGAGCGTCTACTTC
AACGAGGCTTCCGGTAACAAGTACGTTCCTCGTGCCGTCCTCGTCGATCTCGAGCCCGGTACCA
TGGATGCCGTCCGCGCCGGTCCCTTCGGCCAGCTCTTCCGCCCTGACAACTTCGTCTTCGGTCA
GTCCGGTGCTGGCAACAACTGG
>N_sonneratae_MFLUCC_17_1745
CATTATAGAGTTTTCTAAACTCCCAACCCATGTGAACTTACCTTTTGTTGCCTCGGCAGAAGTTAT
AGGTCTTCTTATAGCTGCTGCCGGTGGACCATTAAACTCTTGTTATTTTATGTAATCTGAGCGTCT
TATTTTAATAAGTCAAAACTTTCAACAACGGATCTCTTGGTTCTGGCATCGATGAAGAACGCAGC
GAAATGCGATAAGTAATGTGAATTGCAGAATTCAGTGAATCATCGAATCTTTGAACGCACATTG
CGCCCATTAGTATTCTAGTGGGCATGCCTGTTCGAGCGTCATTTCAACCCTTAAGCCTAGCTTAG
TGTTGGGAATCTACTTCTTTA--
TAGTTGTAGTTCCTGAAATACAACGGCGGATTTGTAGTATCCTCTGAGCGTAGTAA-
TTTTTTTCTCGCTTTTGTTAGGTGCTATAACTCCCAGCCGCTAAACCCCCAATTTTTTGTGGTTGAC
CTCGGATCAGGT??????TTAGTCATTTATTGATTCCCATCATCATCCCCCATCCCCCTTCAC-
TTCAGCATCATAATTTTCAACCTACGTGTTGAAAATTA-TTTTCGCTCCTTCCACAC-TTTTTT--
CCGCTGGTTACCCCGCCGCGAGGCACCCGCACGACCCCGCGGTGCAAACGAAAAATTTCTTAT
CACAGCCCCACCTTGCACAAGCAACCATGCATTGCTCATGAGACCCACTT--
TGAACAATCGCTAATGCCTTCATACAGGAAGCCGCCGAGCTCGGTAAGGGTTCCTTCAAGTACG
CCTGGGTTCTTGACAAGCTCAAGGCCGAGCGTGAGCGTGGTATCACCATCGATATCGCTCTCTG
GAAGTTCGAGACCAACGAGTACAATGTCACCGTCATTGGTTAGTACCCCTCCACCTATGCCATG
TGCTGCTCCATAAGACACTTGACTAACCTTGCTTCATAGACGCTCCCGGTCACCGTGATTTCATC
AAG????TGCTGCCTTCTGGTATGTTACCTGTCTGCCTCGACACGGCCTTAATACGACGTTTTTCGT
GCCTGCACGACGGCCCCGAACAGTTGAAATAGGTCAAGATAGAGGGAACATAATACTAATAGG
TCATTTATAGGCAAACCATCTCTGGCGAGCACGGTCTCGACAGCAATGGAGTGTATGTACTAAC
TTCAATTCCTCCTGCTTCCTGTTGAGCTTGTAGGCTGAC-

TCGATGGCCATTTAGCTACAACGGTACCTCCGAGCTCCAGCTCGAGCGTATGAGCGTCTACTTC
AACGAGGCTTCCGGCAACAAGTACGTTCCTCGTGCTGTCCTCGTCGATCTCGAGCCCGGTACCA
TGGATGCCGTCCGCGCCGGTCCCTTCGGCCAGCTCTTCCGCCCTGACAACTTCGTCTTCGGTCA
GTCCGGTGCTGGCAACAACTGG
>N_steyaertii_IMI_192475
CATTATAGAGTTTTCTAAACTCCCAACCCATGTGAACTTACCTTTTGTTGCCTCGGCAGAAGTTAC
AGGTTACCCTGTAGCTGCTGCCGGTGGACCACTAAACTCTTGTTATTTTATGTAATCTGAGCGTC
TTATTTTAATAAGTCAAAACTTTCAACAACGGATCTCTTGGTTCTGGCATCGATGAAGAACGCAG
CGAAATGCGATAAGTAATGTGAATTGCAGAATTCAGTGAATCATCGAATCTTTGAACGCACATT
GCGCCCATTAGTATTCTAGTGGGCATGCCTGTTCGAGCGTCATTTCAACCCTTAAGCCTAGCTTA
GTGTTGGGAATCTACTTCTTTTATTAGTTGTAGTTCCTGAAATACAACGGCGGATTTGTAGTATCC
TCTGAGCGTAGTAA-
ATCTTTTCTCGCTTTTGTCAGGT???AG
AAGGTTAGTCATCTACTGATTCCCATCAT----------
TTTCATTCCCCTTGAGCGTCATGATTTTCGACCTACCTGTTGAAAATTATTTTTCGCTCCTTCCACA
T--TTTTT--
TCGCTGGTTACCCCGCCGCGAGGCACCCGCACGACCCCGCGGTGCAAACGAAAAATTTCCTAT
CACAGCCCCACCTTGCATAAGCAACTATGCACTGCTCATGAGACCCACTT--TGAA----------
----
CCGCCGAGCTCGGTAAGGGTTCCTTCAAGTACGCCTGGGTTCTTGACAAGCTCAAGGCCGAGC
GTGAGCGTGGTATCACCATCGATATCGCTCTCTGGAAGTTCGAGACCAACGAGTACAATGTCAC
CGTCATTGGTTAGTACCCTACCACCCATGCCATGTGCTGCTGAACAAGACAC-
CGACTAACATTGCTCCACAGACGCTCCCGGTCACCGTGATTTCATCAAGAACATGCTGCTTTCTG
GTATGTAGCCTGTCTACCTGGACACGGTCTC-
ATACGACATTTTTCGTGACTTCACGACGGCCTCGAATAG-
TTGATTGGGTCAAGATAGAGATAACATGATGCTAATGGGTCATTCATAGGCAAACCATCTCTGG
CGAGCACGGTCTCGACAGCAATGGAGTGTATGTACCATTTTAAATTCCTTTTGCTTCTTGTTGAG
CTCGTAGGCTGAC-
TTGATGGCCATTTAGCTACAACGGCACCTCCGAGCTCCAGCTCGAGCGTATGAGCGTCTACTTC
AACGAGGCTTCCGGCAACAAGTACGTTCCTCGTGCCGTCCTTGTCGATCTCGAGCCCGGTACCA
TGGATGCCGTCCGCGCCGGTCCCTTCGGCCAGCTCTTCCGCCCTGACAACTTCGTCTTCGGTCA
GTCCGGTGCTGGCAACAACTGG
>N_surinamensis_CBS_450_74
CATTATAGAGTTTTCTAAACTCCCAACCCATGTGAACTTACCTTTTGTTGCCTCGGCAGAAGTTAT
AGGTCTTCTTATAGCTGCTGCCGGTGGACCATTAAACTCTTGTTATTTTATGTAATCTGAGCGTCT
TATTTTAATAAGTCAAAACTTTCAACAACGGATCTCTTGGTTCTGGCATCGATGAAGAACGCAGC
GAAATGCGATAAGTAATGTGAATTGCAGAATTCAGTGAATCATCGAATCTTTGAACGCACATTG
CGCCCATTAGTATTCTAGTGGGCATGCCTGTTCGAGCGTCATTTCAACCCTTAAGCCTAGCTTAG
TGTTGGGAATCTACTTCTTTTATTAGTTGTAGTTCCTGAAATACAACGGCGGATTTGTAGTATCCT
CTGAGCGTAGTAA-
TTTTTTTCTCGCTTTTGTTAGGTGCTATAACTCCCAGCCGCTAAACCCCCAATTTTTTGTGGTTGAC
CTCGGATCAGGTAGAAGGTTAGTCATCTATTGATTCCCATCAT-------CATTCCCCTTCTC-
TTCAGCGTCATGATTTTCAACCTACGCGTTGAAAATTA-TTTTCGCTCCTTCCACAC--TTTTT--
TCGCTGGTTACCCCGCCGCGAGGCACCCGCACGACCCCGCGGTGCAAACGAAAAATTTCTTAT

CACAGCCCCACCTTTCACAAGCAACCATGCATTGCTCATGAGACCCACTT--
TGAACGATTGCTAATGCCTTCATACAGGAAGCCGCCGAGCTCGGTAAGGGTTCCTTCAAGTACG
CCTGGGTTCTTGACAAGCTCAAGGCCGAGCGTGAGCGTGGTATCACCATCGATATCGCTCTCTG
GAAGTTCGAGACCAACGAGTACAATGTCACCGTCATTGGTTAGTACCCCTCCACCTATGCCATG
TGCTGCTCCATAAGACACTTGACTAACCTTGCTTCATAGACGCTCCCGGTCACCGTGATTTCATC
AAGAA??TGCTGCCTTCTGGTATGTTACCTGTCTGCCTCGACACGGCCTTACTACGACGTTTTTCG
TGCCTGCACGACGGCCCCGAACAG-
TGAAATAGGTCAAGATAGAGGGAACATGATACTAATAGGTCATTTATAGGCAAACCATCTCTGG
CGAGCACGGTCTCGACAGCAATGGAGTGTATGTACTAACTTCAATTCCTCCTGCTTCCTGTTGAG
TTTGTAGGCTGAC-
TCGATGGCCATTTAGCTACAACGGTACCTCCGAGCTCCAGCTCGAGCGTATGAGCGTCTACTTC
AACGAGGCTTCCGGCAACAAGTACGTTCCTCGTGCCGTCCTCGTCGATCTCGAGCCCGGTACCA
TGGATGCCGTCCGCGCCGGTCCCTTCGGCCAGCTCTTCCGCCCTGACAACTTCGTCTTCGGTCA
GTCCGGTGCTGGCAACAAC???
>N_thailandica_MFLUCC_17_1730
CATTATAGAGTTTTCTAAACTCCCAACCCATGTGAACTTACCTTTTGTTGCCTCGGCAGAAGTTAT
AGGTCTTCCTATAGCTGCTGCCGGTGGACCATTAAACTCTTGTTATTTTATGTAATCTGAGCGTCT
TATTTTAATAAGTCAAAACTTTCAACAACGGATCTCTTGGTTCTGGCATCGATGAAGAACGCAGC
GAAATGCGATAAGTAATGTGAATTGCAGAATTCAGTGAATCATCGAATCTTTGAACGCACATTG
CGCCCATTAGTATTCTAGTGGGCATGCCTGTTCGAGCGTCATTTCAACCCTTAAGCCTAGCTTAG
TGTTGGGAATCTACTTCTTTA--
TAGTTGTAGTTCCTGAAATACAACGGCGGATTTGTAGTATCCTCTGAGCGTAGTAA-
TTTTTTTCTCGCTTTTGTTAGGTGCTATAACTCCCAGCCGCTAAACCCCCAATTTTTTGTGGTTGAC
CTCGGATCAGGT??????TTAGTCATTTATTGATTCCCATCATCATCCCCCATCCCCCTTCAC-
TTCAGCATCATAATTTTCAACCTACGTGTTGAAAATTA-TTTTCGCTCCTTCCACAC-TTTTTT--
CCGCTGGTTACCCCGCCGCGAGGCACCCGCACGACCCCGCGGTGCAAACGAAAAATTTCTTAT
CACAGCCCCACCTTGCACAAGCAACCATGCATTGCTCATGAGACCCACTT--
TGAACAATCGCTAATGCCTTCATACAGGAAGCCGCCGAGCTCGGTAAGGGTTCCTTCAAGTACG
CCTGGGTTCTTGACAAGCTCAAGGCCGAGCGTGAGCGTGGTATCACCATCGATATCGCTCTCTG
GAAGTTCGAGACCAACGAGTACAATGTCACCGTCATTGGTTAGTACCCCTCCACCTATGCCATG
TGCTGCTCCATAAGACACTTGACTAACCTTGCTTCATAGACGCTCCCGGTCACCGTGATTTCATC
AAG????TGCTGCCTTCTGGTATGTTACCTGTCTGCCTCGACACGGCCTTAATACGACGTTTTTCGT
GCCTGCACGACGGCCCCGAACAGTTGAAATAGGTCAAGATAGAGGGAACATAAGACTAATAGG
TCATTTATAGGCAAACCATCTCTGGCGAGCACGGTCTCGACAGCAATGGAGTGTATGTACTAAC
TTCAATTCCTCCTGCTTCCTGTTGAGCTTGTAGGCTGAC-
TCGATGGCCATTTAGCTACAACGGTACCTCCGAGCTCCAGCTCGAGCGTATGAGCGTCTACTTC
AACGAGGCTTCCGGCAACAAGTACGTTCCTCGTGCTGTCCTCGTCGATCTCGAGCCCGGTACCA
TGGATGCCGTCCGCGCCGGTCCCTTCGGCCAGCTCTTCCGCCCTGACAACTTCGTCTTCGGTCA
GTCCGGTGCTGGCAACAACTGG
>N_umbrinospora_MFLUCC_12_0285
CATTATAGAGTTTTCTAAACTCCCAACCCATGTGAACTTACCTTTTGTTGCCTCGGCAGAAGTTAT
AGGTCTTCTTATAGCTGCTGCCGGTGGACCATTAAACTCTTGTTATTTTATGTAATCTGAGCGTCT
TATTTTAATAAGTCAAAACTTTCAACAACGGATCTCTTGGTTCTGGCATCGATGAAGAACGCAGC
GAAATGCGATAAGTAATGTGAATTGCAGAATTCAGTGAATCATCGAATCTTTGAACGCACATTG

CGCCCATTAGTATTCTAGTGGGCATGCCTGTTCGAGCGTCATTTCAACCCTTAAGCCTAGCTTAG
TGTTGGGAATCTACTTCTTTA--
TAGTTGTAGTTCCTGAAATACAACGGCGGATTTGTAGTATCCTCTGAGCGTAGTAA-
TTTTTTTCTCGCTTTTGTTAGGTGCTATAACTCCCAGCCGCTAAACCCCCAATTTTTTGTGGTTGAC
CTCGGATCAGGTAGAAGGTTAGTCATCTACTGATTCCCGTCAT-------CATTCTCCTTCAC-
TTCAGCGTCATGATTTTCAACCTACGTGTTGAAAATTA-TTTTCGCTCCTTCCACAC--TTTTT--
TCGCTGGTTACCCCGCCGCGAGGCACCCGCACGACCCCGCGGTGCAAACGAAAAATTTCTTAT
CACAGCCCCACCTTGCATAAGCAACCATGCATTGCTCATGAGATCCACTT--
TGAACAATCGCTAATGCCTTCATACAGGAAGCCGCCGAGCTCGGTAAGGGTTCCTTCAAGTACG
CCTGGGTTCTTGACAAGCTCAAGGCCGAGCGTGAGCGTGGTATCACCATCGATATCGCTCTCTG
GAAGTTCGAGACCAACGAGTACAATGTCACCGTCATTGGTTAGTACCCCTCCACCTATGCCATG
TGCTGCTCCATAAGACACTTGACTAACCTTGCTTTATAGACGCTCCCGGTCACCGTGATTTCATC
AAGAACATGCTGCTTTCTGGTATGTAACCTGTCTG-CTCGAC-
CGGCCTTGATACGACGTTTTTCGTGCCTGCACGACGGCCTCGAACAG-
TGAAATAGGTCAAGATAGAGGGAACATAATACTAATAGGTCATTTATAGGCAAACCATCTCTGG
CGAGCACGGTCTCGACAGCAATGGAGTGTATGTACTAACTTCAATTCCTCCTGCTTCCTGTTGAG
CTTGTAGGCTGAC-
TCGATGGCCATTTAGCTACAACGGTACCTCCGAGCTCCAGCTCGAGCGTATGAGCGTCTACTTC
AACGAGGCTTCCGGCAACAAGTACGTTCCTCGTGCCGTCCTCGTCGATCTCGAGCCCGGTACCA
TGGATGCCGTCCGCGCCGGTCCCTTCGGCCAGCTCTTCCGCCCTGACAACTTCGTCTTCGGTCA
GTCCGGTGCTGGCAACAACTGG
>N_vheenae_BRIP_72293a
CATTATAGAGTTTTCTAAACTCCCAACCCATGTGAACTTACCTTTTGTTGCCTCGGCAGAAGTTAT
AGGTCTTCTTATAGCTGCTGCCGGTGGACCATTAAACTCTTGTTATTTTATGTAATCTGAGCGTCT
TATTTTAATAAGTCAAAACTTTCAACAACGGATCTCTTGGTTCTGGCATCGATGAAGAACGCAGC
GAAATGCGATAAGTAATGTGAATTGCAGAATTCAGTGAATCATCGAATCTTTGAACGCACATTG
CGCCCATTAGTATTCTAGTGGGCATGCCTGTTCGAGCGTCATTTCAACCCTTAAGCCTAGCTTAG
TGTTGGGAATCTACTTCTCTTCGGAGTTGTAGTTCCTGAAATACAACGGCGGATTTGTAGTATCC
TCTGAGCGTAGTAA-
TTTTTTTCTCGCTTTTGTTAGGTGCTATAACTCCCAGCCGCTAAACCCCCAATTTTTTGTGGTTGAC
CTCGGATCAGGTAGAAGGTTAGTCATCTACTGATTCCCGTCAT-CATTATCATTCTCCTTCAC-
TTCAGCGTCATGATTTTCAACCTATGTGTTGAAAATTA-TTTTCGCTCCTTCCACAC--TTTTT--
TCGCTGGTTACCCCGCCGCGAGGCACCCGCACGACCCCGCGGTGCAAACGAAAAATTTCTTAT
CACAGCCCCACCTTGCATAAGCAACCATGCATTGCTCATGAGATCCACTT--
CGAACAATTGCTAATGCCTTCATACAGGAAGCCGCCGAGCTCGGTAAGGGTTCCTTCAAGTACG
CCTGGGTTCTTGACAAGCTCAAGGCCGAGCGTGAGCGTGGTATCACCATCGATATCGCTCTCTG
GAAGTTCGAGACCAACGAGTACAATGTCACCGTCATTGGTTAGTACCCCTCCACCTATGCCATG
TGCTGCTCCATAAGACACTTGACTAACCTTGCTTCATAGACGCTCCCGGTCACCGTGATTTCATC
AAGAACA??????TTTCTGGTATGTAATCTGTCTGCCTCGACACGGCCTTAATACGACGTTTTTCGT
GCCTGCACGACGGCCCCGATCAG-
TGAATTAGGTCAAGATACAGGGAACATGATGCTAATAGGTCATTTATAGGCAAACCATCTCTGG
CGAGCACGGTCTCGACAGCAATGGAGTGTATGTACTATTTTCAATTCCTCCTGCTTCCTGTTGAG
CTTGTAGGCTGAC-
TCGATGGCCATTTAGCTACAACGGTACCTCCGAGCTCCAGCTCGAGCGTATGAGCGTCTACTTC

AACGAGGCTTCCGGCAACAAGTACGTTCCTCGTGCCGTCCTCGTCGATCTCGAGCCCGGTACCA
TGGATGCCGTCCGCGCCGGTCCCTTCGGCCAGCTCTTCCGCCCTGACAACTTCGTCTTCGGTCA
GTCCGGTGCTGG??????????
>N_vitis_MFLUCC_15_1265
??ATAGGTCTTCTTATAGCTG
CTGCCGGTGGACCATTAAACTCTTGTTATTTTATGTAATCTGAGCGTCTTATTTTAATAAGTCAAA
ACTTTCAACAACGGATCTCTTGGTTCTGGCATCGATGAAGAACGCAGCGAAATGCGATAAGTAA
TGTGAATTGCAGAATTCAGTGAATCATCGAATCTTTGAACGCACATTGCGCCCATTAGTATTCTA
GTGGGCATGCCTGTTCGAGCGTCATTTCAACCCTTAAGCCTAGCTTAGTGTTGGGAATCTACTTC
TTTTATTAGTTGTAGTTCCTGAAATACAACGGCGGATTTGTAGTATCCTCTGAGCGTAGTAA-
TTTTTTTCTCGCTTTTGTTAGGTGCTACAACTCCCAGCCGCTAAACCCCCAATTTTTTGTGGTTGA
CCTCGGATCAGGT???
??CGCTGGTTACCCCGCCGCGAGGCACCCGC
ACGACCCCGCGGTGCAAACGAAAAATTTCTTATCACAGCCCCACCTTGCATAAGCAACCATGCA
TTGCTCATGAGATCCACTT--
TGAACAATTGCTAATGCCTTCATACAGGAAGCCGCCGAGCTCGGTAAGGGTTCCTTCAAGTACG
CCTGGGTTCTTGACAAGCTCAAGGCCGAGCGTGAGCGTGGTATCACCATCGATATCGCTCTCTG
GAAGTTCGAGACCAACGAGTACAATGTCACCGTCATTGGTTAGTACCCCTCCACCTGTGCCATG
TGCTGCTCCATAAGACACTTGACTAACCTTGCTTCATAGACGCTCCCGGTCACCGTGATTTCATC
AAGA??
???????????????????AGAGGGAACATGATGCTAATAGGTCATTGATAGGCAAACCATCTCTGGCG
AGCACGGTCTCGACAGCAATGGAGTGTATGTACTATTTTTAATTCCTCCTGCTTCCTGTTAAGCTT
GTAGGCTGAC-
TCGATGGCCATTTAGCTACAACGGTACCTCCGAGCTCCAGCTCGAGCGTATGAGCGTCTACTTC
AACGAGGCTTCCGGCAACAAGTACGTTCCTCGTGCCGTCCTCGTCGATCTCGAGCCCGGTACCA
TGGATGCCGTCCGCGCCGGTCCCTTCGGCCAGCTCTTCCGCCCTGACAACTTCGTCTTCGGTCA
G?????????????????????
>N_zakeelii_BRIP_72282a
CATTATAGAGTTTTCTAAACTCCCAACCCATGTGAACTTACCTTTTGTTGCCTCGGCAGAAGTTAT
AGGTCTTCTTATAGCTGCTGCCGGTGGACCATTAAACTCTTGTTATTTTATGTAATCTGAGCGTCT
TATTTTAATAAGTCAAAACTTTCAACAACGGATCTCTTGGTTCTGGCATCGATGAAGAACGCAGC
GAAATGCGATAAGTAATGTGAATTGCAGAATTCAGTGAATCATCGAATCTTTGAACGCACATTG
CGCCCATTAGTATTCTAGTGGGCATGCCTGTTCGAGCGTCATTTCAACCCTTAAGCCTAGCTTAG
TGTTGGGAATCTACTTCTCTTAGGAGTTGTAGTTCCTGAAATACAACGGCGGATTTGTAGTATCC
TCTGAGCGTAGTAA-
TTTTTTTCTCGCTTTTGTTAGGTGCTATAACTCCCAGCCGCTAAACCCCCAATTTTTTGTGGTTGAC
CTCGGATCAGGTAGAAGGTTAGTCATCTACTGTTTCCCGTCAT-------CATTCTCCTTCAC-
TTCAGCGTCATGATTTTCAACCTACGTGCTGAAAATTA-TTTTCGCTCCTTCCACAC--TTTTT--
TCGCTGGTTACCCCGCCGCGAGGCACCCGCACGACCCCGCGGTGCAAACGAAAAATTTCTTAT
CACAGCCCCACCTTGCATAAGCAACCATGCATTGCTCATGAGATCCACTT--
TGAACAATTGCTAATGCCTTCGTACAGGAAGCCGCCGAGCTCGGTAAGGGTTCCTTCAAGTACG
CCTGGGTTCTTGACAAGCTCAAGGCCGAGCGTGAGCGTGGTATCACCATCGATATCGCTCTCTG
GAAGTTCGAGACCAACGAGTACAATGTCACCGTCATTGGTCAGTACCCCTCCACCTATGCCATG
TGCTGCTCCATAAGACACTTGACTAACCTTGCTTCATAGATGCTCCCGGTCACCGTGATTTCATC

AAGAACA????GCTTTCTGGTATGTAACCTGTCTGCCTCGACACGGCCTTAATACGACGTTTTTCG
TGCCTGCACGACGGCCCCGAACAG-
TGATATAGGTCAAGATAGAGGGAACATGATGCTAATAGGTCATTGATAGGCAAACCATCTCTGG
CGAGCACGGTCTCGACAGCAATGGAGTGTATGTACTATTTTTAATTCCTCCTGCTTCCTGTTAAG
CTTGTAGGCTGAC-
TCGATGGCCATTTAGCTACAACGGTACCTCCGAGCTCCAGCTCGAGCGTATGAGCGTCTACTTC
AACGAGGCTTCCGGCAACAAGTACGTTCCTCGTGCCGTCCTCGTCGATCTCGAGCCCGGTACCA
TGGATGCCGTCCGCGCCGGTCCCTTCGGCCAGCTCTTCCGCCCTGACAACTTCGTCTTCGGTCA
GTCCGGTGCTGGCAACAACTGG
>N_zimbabwana_CBS_111495
??
??
??
??
??
??????????????????????????????????????AGAAGGTTAGTCATCTATTGATTCCCATCAT-------
CATTCCCCTTCAC-TTCAGCGTCATGATTTTCAACATACGTGTTGAAAATTA-
TTTTCGCTCCTTCCACAC-TTTTTT--
TCGCTGGTTACCCCGCCGCGAGGCACCCGCACGACCCCGCGGTGCAAACGAAAAATTTCTTAT
CACAGCCCCACCTTGCACAAGCAACCATGCATTACTCATGAGACCCACTT--
TGAACAATTGCTAATTCCTTCATTCAGGAAGCCGCCGAGCTCGGTAAGGGTTCCTTCAAGTACG
CCTGGGTTCTTGACAAGCTCAAGGCCGAGCGTGAGCGTGGTATCACCATCGATATCGCTCTCTG
GAAGTTCGAGACCAACGAGTACAATGTCACCGTCATTGGTCAGTACCCTTCTACCTATGCCATG
TACTGCTCCATAAGACACTTGACTAACCTTGCTTCATAGACGCTCCCGGTCACCGTGATTTCA???
??????TGCTGCCTTCTGGTATGTAACCTGTCTGCCTCGACACGGCCTTGATACGACGTTTTTCGTG
CCTGCACGACGGCCCCGAACAG-
TGAATTAGGTCAAGATACAGGGAACATGATGCTAATAGGTCATTTATAGGCAAACCATCTCTGG
CGAGCACGGTCTCGACAGCAATGGAGTGTATGTACCATTTTCAATCCCTCCTGCTTCCTGTTGAG
CTTGTAGGCTGAC-
TCGATGGCCATTTAGCTACAACGGTACCTCCGAGCTTCAGCTCGAGCGTATGAGCGTCTACTTC
AACGAGGCTTCTGGCAACAAGTACGTTCCTCGTGCCGTCCTCGTCGATCTCGAGCCCGGTACCA
TGGATGCCGTCCGCGCCGGTCCCTTCGGCCAGCTCTTCCGCCCTGACAACTTCGTCTTCGGTCA
GTCCGGTGCTGGCAACAACTGG
>Neopestalotiopsis_acrostichi_MFLUCC_17_1754
CATTATAGAGTTTTCTAAACTCCCAACCCATGTGAACTTACCTTTTGTTGCCTCGGCAGAAGTTAT
AGGTCTTCTTATAGCTGCTGCCGGTGGACCATTAAACTCTTGTTATTTTATGTAATCTGAGCGTCT
TATTTTAATAAGTCAAAACTTTCAACAACGGATCTCTTGGTTCTGGCATCGATGAAGAACGCAGC
GAAATGCGATAAGTAATGTGAATTGCAGAATTCAGTGAATCATCGAATCTTTGAACGCACATTG
CGCCCATTAGTATTCTAGTGGGCATGCCTGTTCGAGCGTCATTTCAACCCTTAAGCCTAGCTTAG
TGTTGGGAATCTACTTCTTTTATTAGTTGTAGTTCCTGAAATACAACGGCGGATTTGTAGTATCCT
CTGAGCGTAGTAA-
TTTTTTTCTCGCTTTTGTTAGGTGCTATAACTCCCAGCCGCTAAACCCCCAATTTTTTGTGGTTGAC
CTCGGATCAGGT??????TTAGTCATTTATTGATTCCCATCAT-------CATCCCCCTTCAC-
TTCAGCATCATAATTTTTAACCTACGTGTTGAAAATTA-TTTTCGCTCCTTCCACAC--TTTTT--

TCGCTGGTTACCCCGCCGCGAGGCACCCGCACGACCCCGCGGTGCAAACGAAAAATTTCTTAT
CACAGCCCCACCTTGCACAAGCAACCATGCATTGCTCATGAGACCCACTT--
TGAACAATTGCTAATGCCTTCATACAGGAAGCCGCCGAGCTCGGTAAGGGTTCCTTCAAGTACG
CCTGGGTTCTTGACAAGCTCAAGGCCGAGCGTGAGCGTGGTATCACCATCGATATCGCTCTTTG
GAAGTTCGAGACCAACGAGTACAATGTCACCGTCATTGGTTAGTACCCCTCCACCTATGCCATG
TGCTGCACCATAAGACACTTGACTAACCTTGCTTCATAGACGCTCCCGGTCACCGTGATTTCATC
AAG????TGCTGCCTTCTGGTATGTTACCTGTCTGCCTCGACACGGCCTTACTACGACGTTTTTCGT
GCCTGCACGACGGCCCCGAACAG-
TGAAATAGGTCAAGATAGAGGGAACATGATACTAATAGGTCATTTATAGGCAAACCATCTCTGG
CGAGCACGGTCTCGACAGCAATGGAGTGTATGTACTAACTTCAATTCCTCCTGCTTCCTGTTGAG
TTTGTAGGCTGAC-
TCGATGGCCATTTAGCTACAACGGTACCTCCGAGCTCCAGCTCGAGCGTATGAGCGTCTACTTC
AACGAGGCTTCCGGCAACAAGTACGTTCCTCGTGCCGTCCTCGTCGATCTCGAGCCCGGTACCA
TGGATGCCGTCCGCGCCGGTCCCTTCGGCCAGCTCTTCCGCCCTGACAACTTCGTCTTCGGTCA
GTCCGGTGCTGGCAACAACTGG
>Neopestalotiopsis_dendrobii_MFLUCC_14_0132
CATTATAGAGTTTTCTAAACTCCCAACCCATGTGAACTTACCTTTTGTTGCCTCGGCAGAAGTTAT
AGGTCTTCTTATAGCTGCTGCCGGTGGACCATTAAACTCTTGTTATTTTATGTAATCTGAGCGTCT
TATTTTAATAAGTCAAAACTTTCAACAACGGATCTCTTGGTTCTGGCATCGATGAAGAACGCAGC
GAAATGCGATAAGTAATGTGAATTGCAGAATTCAGTGAATCATCGAATCTTTGAACGCACATTG
CGCCCATTAGTATTCTAGTGGGCATGCCTGTTCGAGCGTCATTTCAACCCTTAAGCCTAGCTTAG
TGTTGGGAATCTACTTCTCTTAGGAGTTGTAGTTCCTGAAATACAACGGCGGATTTGTAGTATCC
TCTGAGCGTAGTAA-
TTTTTTTCTCGCTTTTGTTAGGTGCTATAACTCCCAGCCGCTAAACCCCCAA???????????????????
????????AGAAGGTTAGTCATTTATTGATTCCCATCAT-------CATCCCCCTTCAC-
TTCAGCATCATAATTTTTAACCTACGTGTTGAAAATTA-TTTTCGCTCCTTCCACAC--TTTTT--
TCGCTGGTTACCCCGCCGCGAGGCACCCGCACGACCCCGCGGTGCAAACGAAAAATTTCTTAT
CACAGCCCCACCTTGCACAAGCAACCATGCATTGCTCATGAGACCCACTT--
TGAACAATTGCTAATGCCTTCATACAGGAAGCCGCCGAGCTCGGTAAGGGTTCCTTC??????????
??
??
??
??
??
??
??
????????
>Neopestalotiopsis_sp1_CFCC_54337
CATTATAGAGTTTTCTAAACTCCCAACCCATGTGAACTTACCTTTTGTTGCCTCGGCAGAAGTTAT
AGGTCTTCTTATAGCTGCTGCCGGTGGACCATTAAACTCTTGTTATTTTATGTAATCTGAGCGTCT
TATTTTAATAAGTCAAAACTTTCAACAACGGATCTCTTGGTTCTGGCATCGATGAAGAACGCAGC
GAAATGCGATAAGTAATGTGAATTGCAGAATTCAGTGAATCATCGAATCTTTGAACGCACATTG
CGCCCATTAGTATTCTAGTGGGCATGCCTGTTCGAGCGTCATTTCAACCATTAAGCCTAGCTTAG
TGTTGGGAATCTACTTCTCTTAGGAGTTGTAGTTCCTGAAATACAACGGCGGATTTGTAGTATCC

TCTGAGCGTAGTAA-
TTTTTTTCTCGCTTTTGTTAGGTGCTATAACTCCCAGCCGCTAAACCCCCAATTTTTTGTGGTTGAC
CTCGGATCAGGTAGAAGGTTAGTCATCTGTTGATTCCTATCAT--------CATTCCCTTCAC-
ATCAGCGTCATGATTTTCAACCTACGTGTTGAAAATTA-TTTTCGCTCCTTCCACAC--TTTTT--
TCGCTGGTTACCCCGCCGCGAGGCACCCGCACGACCCCGCGGTGCAAACGAAAAATTTCTTAT
CACAGCCCCACCTTGCACAAGCAACCATGCATTGCTCATGAGACCCACTT--
TGAACAATTGCTAATGCCTTCATACAGGAAGCTGCCGAGCTCGGTAAGGGTTCCTTCAAGTACG
CCTGGGTTCTTGACAAGCTCAAGGCCGAGCGTGAGCGTGGTATCACCATCGATATCGCTCTCTG
GAAGTTCGAGACCAACGAGTACAATGTCACCGTCATTGGTTAGTACCCCTCCACCAATGCCATA
TGCTGCTCCATAAGACACTTGACTAACCTTACTTCATAGACGCTCCCGGTCACCGTGATTTCATC
AAGAACATGCTGCCTTCTGGTATGTAATCTGTCTGCCTCGACACGGCCTCAATACGACGTTTTTC
GTGCCTGCACGACGGCCCCGATCAG-
TGAATTAGGTCAAGATACAGGGAACATGACGCTAATAGGTCATTTATAGGCAAACCATCTCTGG
CGAGCACGGTCTCGACAGCAATGGAGTGTATGTACTATTTTCAATTCCTCCTGCTTCCTGTTGAG
CTTGTAGGCTGAC-
TCGATGGCCATTTAGCTACAACGGTACCTCCGAGCTCCAGCTCGAGCGTATGAGCGTCTACTTC
AACGAGGCTTCCGGCAACAAGTACGTTCCTCGTGCCGTCCTCGTCGATCTCGAGCCCGGTACCA
TGGATGCCGTCCGCGCCGGTCCCTTCGGCCAGCTCTTCCGCCCTGACAACTTCGTCTTCGG?????
????????????????????
>Neopestalotiopsis_sp1_ZX12_1
CATTATAGAGTTTTCTAAACTCCCAACCCATGTGAACTTACCTTTTGTTGCCTCGGCAGAAGTTAT
AGGTCTTCTTATAGCTGCTGCCGGTGGACCATTAAACTCTTGTTATTTTATGTAATCTGAGCGTCT
TATTTTAATAAGTCAAAACTTTCAACAACGGATCTCTTGGTTCTGGCATCGATGAAGAACGCAGC
GAAATGCGATAAGTAATGTGAATTGCAGAATTCAGTGAATCATCGAATCTTTGAACGCACATTG
CGCCCATTAGTATTCTAGTGGGCATGCCTGTTCGAGCGTCATTTCAACCATTAAGCCTAGCTTAG
TGTTGGGAATCTACTTCTCTTAGGAGTTGTAGTTCCTGAAATACAACGGCGGATTTGTAGTATCC
TCTGAGCGTAGTAA-
TTTTTTTCTCGCTTTTGTTAGGTGCTATAACTCCCAGCCGCTAAACCCCCAATTTTTTGTGGTTGAC
CTCGGATCAGGTAGAAGGTTAGTCATCTGTTGATTCCTATCAT--------CATTCCCTTCAC-
ATCAGCGTCATGATTTTCAACCTACGTGTTGAAAATTA-TTTTCGCTCCTTCCACAC--TTTTT--
TCGCTGGTTACCCCGCCGCGAGGCACCCGCACGACCCCGCGGTGCAAACGAAAAATTTCTTAT
CACAGCCCCACCTTGCACAAGCAACCATGCATTGCTCATGAGACCCACTT--
TGAACAATTGCTAATGCCTTCATACAGGAAGCTGCCGAGCTCGGTAAGGGTTCCTTCAAGTACG
CCTGGGTTCTTGACAAGCTCAAGGCCGAGCGTGAGCGTGGTATCACCATCGATATCGCTCTCTG
GAAGTTCGAGACCAACGAGTACAATGTCACCGTCATTGGTTAGTACCCCTCCACCAATGCCATA
TGCTGCTCCATAAGACACTTGACTAACCTTACTTCATAGACGCTCCCGGTCACCGTGATTTCATC
AAGAACA???
??
??
??
??
??
>Neopestalotiopsis_sp2_CFCC_54340
CATTATAGAGTTTTCTAAACTCCCAACCCATGTGAACTTACCTTTTGTTGCCTCGGCAGAAGTTAT

AGGTCTTCTTATAGCTGCTGCCGGTGGACCATTCAACTCTTGTTATTTATGTAATCTGAGCGTCT
TATTTTAATAAGTCAAAACTTTCAACAACGGATCTCTTGGTTCTGGCATCGATGAAGAACGCAGC
GAAATGCGATAAGTAATGTGAATTGCAGAATTCAGTGAATCATCGAATCTTTGAACGCACATTG
CGCCCATTAGTATTCTAGTGGGCATGCCTGTTCGAGCGTCATTTCAACCCTTAAGCCTAGCTTAG
TGTTGGGAATCTACTTCTCTTAGGAGTTGTAGTTCCTGAAATACAACGGCGGATTTGTAGTATCC
TCTGAGCGTAGTAA-
TTTTTTTCTCGCTTTTGTTAGGTGCTATAACTCCCAGCCGCTAAACCCCCAATTTTTTGTGGTTGAC
CTCGGATCAGGTAGAAGGTTAGTCATTTATTGATTCCCATCAT----------TCCCCTTCAC-
TTCAGCATCATAATTTTCAACCTACATGTTGAAAATTA-TTTTCGCTCCTTCCACAC--TTTTT--
TCGCTGGTTACCCCGCCGCGAGGCACCCGCACGACCCCGCGGTGCAAACGAAAAATTTCTTAT
CACAGCCCCACCTTGCACAAGCAACCATGCATTGCTCATGAGACCCACTTTGTGAACAATTGCT
AATGCCTT-
ATATAGGAAGCCGCCGAGCTCGGTAAGGGTTCCTTCAAGTACGCCTGGGTTCTTGACAAGCTCA
AGGCCGAGCGTGAGCGTGGTATCACCATCGATATCGCTCTCTGGAAGTTCGAGACCAACGAGT
ACAATGTCACCGTCATTGGTTAGTACCACTCCACCTATGCCATGTGCTGCTCCATAAGACACTTG
ACTAACCCTGCTTCACAGACGCTCCCGGTCACCGTGATTTCATCAAGAACATGCTGCCTTCTGGT
ATGTAATCTGTCTGCCTCGACACGGCCTTAATACGACGTTTTTCGTGCCTGCACGACGGCCCCG
ATCAG-
TGAATTAGGTCAAGATACAGGGAACATGATGCTAATAGGTCATTTATAGGCAAACCATCTCTGG
CGAGCACGGTCTCGACAGCAATGGAGTGTATGTACTATTTTCAATTCCTCCTGCTTCCTGTTGAG
CTTGTAGGCTGAC-
TCGATGGCCATTTAGCTACAACGGTACCTCCGAGCTCCAGCTCGAGCGTATGAGCGTCTACTTC
AACGAGGCTTCCGGCAACAAGTACGTTCCTCGTGCCGTCCTCGTCGATCTCGAGCCCGGTACCA
TGGATGCCGTCCGCGCCGGTCCCTTCGGCCAGCTCTTCCGCCCTGACAACTTCGTCTTCGG?????
????????????????????
>Neopestalotiopsis_sp2_ZX22B
CATTATAGAGTTTTCTAAACTCCCAACCCATGTGAACTTACCTTTTGTTGCCTCGGCAGAAGTTAT
AGGTCTTCTTATAGCTGCTGCCGGTGGACCATTAAACTCTTGTTATTTATGTAATCTGAGCGTCT
TATTTTAATAAGTCAAAACTTTCAACAACGGATCTCTTGGTTCTGGCATCGATGAAGAACGCAGC
GAAATGCGATAAGTAATGTGAATTGCAGAATTCAGTGAATCATC-AATCTTTGAACG-
ACATTGC-CCCATTA-
TATTCTAGTGGGCATGCCTGTTCGAGCGTCATTTCAACCCTTAAGCCTAGCTTAGTGTTGGGAAT
CTACTTCTCTTAGGAGTTGTAGTTCCTGAAATACAACGGCGGATTTGTAGTATCCTCTGAGCGTA
GTAA-
TTTTTTTCTCGCTTTTGTTAAGTGCTATAACTCCCAGCCGCTAAACCCCCAATTTTTTGTGGTTGAC
CTCGGATCAGGTAGAAGGTTAGTCATTTATTGATTCCCATCAT-------CATCCCCCTTCAC-
TTCAGCATCATAATTTTCAACCTACATGTTGAAAATTA-TTTTCGCTCCTTCCACAC--TTTTT--
TCGCTGGTTACCCCGCCGCGAGGCACCCGCACGACCCCGCGGTGCAAACGAAAAATTTCTTAT
CACAGCCCCACCTTGCACAAGCAACCATGCATTGCTCATGAGACCCACTTTGTGAACAATTGCT
AATGCCTTCATACAGGAAGCCGCCGAGCTCGGTAAGGGTTCCTTCAAGTACGCCTGGGTTCTTG
ACAAGCTCAAGGCCGAGCGTGAGCGTGGTATCACCATCGATATCGCTCTCTGGAAGTTCGAGA
CCAACGAGTACAATGTCACCGTCATTGGTTAGTACCACTCCACCTATGCCATGTGCTGCTCCATA
AGACACTTGACTAACCCTGCTTCACAGACGCTCCCGGTCACCGTGATTTCATCAAGAACATGCT
GCCTTCTGGTATGTAATCTGTCTGCCTCGACACGGCCTTAATACGACGTTTTTCGTGCCTGCACG

ACGGCCCCGATCAG-
TGAATTAGGTCAAGATACAGGGAACATGATGCTAATAGGTCATTTATAGGCAAACCATCTCTGG
CGAGCACGGTCTCGACAGCAATGGAGTGTATGTACTATTTTCAATTCCTCCTGCTTCCTGTTGAG
CTTGTAGGCTGAC-
TCGATGGCCATTTAGCTACAACGGTACCTCCGAGCTCCAGCTCGAGCGTATGAGCGTCTACTTC
AACGAGGCTTCCGGCAACAAGTACGTTCCTCGTGCCGTCCTCGTCGATCTCGAGCCCGGTACCA
TGGATGCCGTCCGCGCCGGTCCCTTCGGCCAGCTCTTCCGCCCTGACAACTTCGTCTTCGGTCA
GTCCGGTGCTGGCAACAACTGG
>P_diversiseta_MFLUCC_12_0287
CATTATAGAGTTTTTTAAACTCCCAACCCATGTGAACTTACC-
ATTGTTGCCTCGGCAGAGGCTACGGCTTACCCTGTAGCGGCTGCCGGTGGACTACTAAACTCTT
GTTATTTTATGTAATCTGAGCGTCTTATTTTAATAAGTCAAAACTTTCAACAACGGATCTCTTGGTT
CTGGCATCGATGAAGAACGCAGCGAAATGCGATAAGTAATGTGAATTGCAGAATTCAGTGAAT
CATCGAATCTTTGAACGCACATTGCGCCCATTAGTATTCTAGTGGGCATGCCTGTTCGAGCGTCA
TTTCAACCCTTAAGCCTAGCTTAGTGTTGGGAGTCTACTGCTTTTACTAGCTGTAGCTCCTGAAAT
ACAACGGCGGATCTGCGATATCCTCTGAGCGTAGTAA-
TTTTTTTCTCGCTTTTGACTGGAGTTGCAGCGTCTAGCCGCTAAACCCCCAATTTTTAATGGTTGA
CCTCGGATCAGGTAGAAGGTTAGTCATCCTCACAATCCCATCAT--------
CATCTTCATCACCATCA-CCTCACCATTTTCAAC-TCAGTGCCGAAAATCA-
GTTTCGCACCGCCCACA---TTCCCAGACAC---TTACCCCGCCGCGTGGC---
CGCACGACCCCGCGGTGCAAACGAAAAATTTCTTATCA--
GCCCCACATCACACAAGCGGCCATGCACTTTTCGT--AACCCACAA--
TGAGCAATTGCTGACCCTGCCAAATAGGAAGCCGCCGAGCTCGGAAAGGGTTCCTTCAAGTAC
GCATGGGTTCTTGACAAGCTCAAGGCCGAGCGTGAGCGTGGTATCACCATCGATATCGCTCTCT
GGAAGTTCGAGACCAACGAGTACAATGTCACCGTCATTGGTCAGTATCCCTGCCCACAAGCTCT
CTCATGGATCCGAACTCAT---
ACTAACATCGCAATATAGATGCTCCCGGTCACCGTGATTTCATCAAGAACATGCTGCTTTCTGGT
ATGTAGCCCATCTACCTCGATACGGCCTCAATACGACGCCTCCCGCAACTCAACAACGACCTCA
ACTAC-TTGGTTGGAACCAAACGAAAG--
ACTTGATACTGACCCGTCTCTGATAGGCAAACCATCTCTGGCGAGCACGGCCTCGACAGCAATG
GAGTGTACGTATCCGTTCCTTGGCTACTTGCTTTCCCACGAACATGCTAGCTGACATCCGTGGTT
GTTCAGCTACAACGGTACCTCCGAGCTCCAGCTCGAGCGCATGAGCGTCTACTTCAACGAGGCT
TCCGGCAACAAGTACGTTCCTCGTGCCGTCCTCGTCGATCTCGAGCCCGGTACCATGGATGCCG
TCCGCGCCGGTCCTTTCGGTCAGCTCTTCCGCCCTGACAACTTCGTCTTCGGTCAGTCCGGTGCC
GGAAACAACTGG
>P_spathulata_CBS_356_86
CATTATAGAGTTTTCTAAACTCCCAACCCATGTGAACTTACC-
ACTGTTGCCTCGGCAGAAGCTACGGCTTACCCTGTAGCGGCTGCCGGTGGACTACTAAACTCTT
GTTATTTTATGTAATCTGAGCGTCTTATTTTAATAAGTCAAAACTTTCAACAACGGATCTCTTGGTT
CTGGCATCGATGAAGAACGCAGCGAAATGCGATAAGTAATGTGAATTGCAGAATTCAGTGAAT
CATCGAATCTTTGAACGCACATTGCGCCCATTAGTATTCTAGTGGGCATGCCTGTTCGAGCGTCA
TTTCAACCCTTAAGCCTAGCTTAGTGTTGGGAGCCTACGGCTTTTACTAGCTGTAGCTCCTGAAA
TACAACGGCGGATCTGCGATATCCTCTGAGCGTAGTAATTTTTTTTCTCGCTTTTGACTGGAGTTG
CAGCGTCTAGCCGCTAAACCCCCAATTTTTAATGGTTGACCTCGGATCAGGTAGAAGGTTAGTC

ATCCTCACGATCCCAACAT--------CATCACCATCACTATCA-CCTTACCATTTTCAAC-
TCGGTGCCGAAAATCAGTTTTCGCGCTGCCCACA---TTCCCAGACAC---
TTACCCCGCCGCGTGGC---CGCACGACCCCGCGGTGCAAACGAAAAATTTCTTATCA--
GCCCCACATCGCACAAACAGCCATGCACTTTTCAC--AATCCACAA--
TGAGCAATTGCTGACCCCGCCAAATAGGAAGCCGCCGAGCTCGGTAAGGGTTCCTTCAAGTAC
GCATGGGTTCTTGACAAGCTCAAGGCCGAGCGCGAGCGTGGTATCACCATCGATATCGCTCTCT
GGAAGTTCGAGACCAACGAGTACAATGTCACCGTCATTGGTCAGTATCCCTGTCCACCAGCTGT
TTCATGCATCCGAACTCGC---
ACTAACATGGCAATATAGATGCTCCCGGTCACCGTGATTTCATCAAGAACATGCTGCCTTCTGGT
ATGTAGCCTATCTACCTCGACACG-
CCTCAATACGACGCCCCCCGCAACTCGACGCCGACCTCAACTAC-
TTGGTTGGATCCAAACGGAAG--ACTTGATACTGACC-GTCTC--
ATAGGCAAACCATCTCTGGCGAGCACGGTCTCGACAGCAATGGAGTGTACGTGCCCTTTCCTTG
GCTACTTGCTTTCCCACGAACATACTAGCTAACCCTCGTGGTTGTTCAGCTACAACGGTACCTCC
GAGCTCCAGCTCGAGCGCATGAGCGTCTACTTCAACGAGGCTTCTGGCAACAAGTACGTTCCTC
GTGCCGTCCTTGTCGATCTCGAGCCCGGAACCATGGATGCCGTCCGCGCCGGTCCTTTCGGTCA
GCTCTTCCGCCCTGACAACTTCGTCTTCGGCCAGTCCGGTGCCGGAAACAACTGG
>TN01
???????GAGTTTTCTAAACTCCCAACCCATGTGAACTTACCTTTTGTTGCCTCGGCAGAAGTTATA
GGTCTTCTTATAGCTGCTGCCGGTGGACCATTAAACTCTTGTTATTTTATGTAATCTGAGCGTCTT
ATTTTAATAAGTCAAAACTTTCAACAACGGATCTCTTGGTTCTGGCATCGATGAAGAACGCAGCG
AAATGCGATAAGTAATGTGAATTGCAGAATTCAGTGAATCATCGAATCTTTGAACGCACATTGC
GCCCATTAGTATTCTAGTGGGCATGCCTGTTCGAGCGTCATTTCAACCCTTAAGCCTAGCTTAGT
GTTGGGAATCTACTTCTTTCATTAGTTGTAGTTCCTGAAATACAACGGCGGATTTGTAGTATCCTC
TGAGCGTAGTAA-
TTTTTTTCTCGCTTTTGTTAGGTGCTATAACTCCCAGCCGCTAAACCCCCAACTTTTTGTGGTTGA
CCTCGGATCAGGTAGAAGGTTAGTCATTTATTGATTCCCGCCAT-------CATCCCCCTTCAC-
TTCAGCATCATAATTTTCAACCTACGTGTTGAAAATTA-TTTTAGCTCCTTCCACAC--TTTTT--
TCGCTGGTTACCCCGCCGCGAGGCACCCGCACGACCCCGCGGTGCAAACGAAAAATTTCTTAT
CACAGCCCCACCTTGCACAAGCAACCATGCATTGCTCATGAGACCCACTT--
TGAACAATTGCTAATGCCTTCATACAGGAAGCCGCCGAGCTCGGTAAGGGTTCCTTCAAGTACG
CCTGGGTTCTTGACAAGCTCAAGGCCGAGCGTGAGCGTGGTATCACCATCGATATCGCTCTCTG
GAAGTTCGAGACCAACGAGTACAATGTCACCGTCATTGGTTAGTACCCCTCCACCTATGTCATG
TGCTGCTCCATAAGAC--
TTGACTAACCTTGCTTCATAGACGCTCCCGGTCACC??????????????????TGCTGCCTTCTGGTAT
GTAACCTGTCTGCCTCGACACGGCCTTAATACGACGTTTTTCGTGCCTGCACGACGGCCCCGAA
CAG-
TGATATAGGTCAAGATAGAGGGAACATGATGCTAATAGGTCATTGATAGGCAAACCATCTCTGG
CGAGCACGGTCTCGACAGCAATGGAGTGTATGTACTATTTTTAATTCCTCCTGCTTCCTGTTAAG
CTTGTAGGCTGAC-
TCGATGGCCATTTAGCTACAACGGTACCTCCGAGCTCCAGCTCGAGCGTATGAGCGTCTACTTC
AACGAGGCTTCCGGCAACAAGTACGTTCCTCGTGCCGTCCTCGTCGATCTCGAGCCCGGTACCA
TGGATGCCGTCCGCGCCGGTCCCTTCGGCCAGCT??
??????????

Pestalotiopsis alignment
>E33
AGGGATCATTATAGAGTTTTTTAAACTCCCAACCCATGTGAACTTACC-
ATTGTTGCCTCGGCAGAAGCTACCTGGT--
TACCTTACCTTGGAACGGCCTACCCTGTAGCGCCTTACCCTGGAACGGCCTACCCTGTAACGGC
TGCCGGTGGACTACCAAACTCTTGTTATTTTATTGTAATCTGAGCGTCTTATTTTAATAAGTCAAA
ACTTTCAACAACGGATCTCTTGGTTCTGGCATCGATGAAGAACGCAGCGAAATGCGATAAGTAA
TGTGAATTGCAGAATTCAGTGAATCATCGAATCTTTGAACGCACATTGCGCCCATTAGTATTCTA
GTGGGCATGCCTGTTCGAGCGTCATTTCAACCCTTAAGCCTAGCTTAGTGTTGGGAGCCTACTG-
-CTTTTGCTAGCGGTAGCTCCTGAAATACAACGGCGGATCTGCGATATCCTCTGAGCGTAGTAA-
TTTTTATCTCGCTTTTGACTGGAGTTGCAGCGTCTTTAGCCGCTAAACCCCCC-AA-
TTTTTAATGGTTGACCTC?????????????????????CC-ATCATTC------CCATC--------
CTCATCATCATCGCCT--CGCAAA----CA-TTTTCCAACC-GGTGCCGAGAATCT-G--------
TTTTCGCATCTGCC-CATTTTCCCAG--ACACTTACCCC--------
GCCGCACGACCCCGCGGTGCAAACGAAAAAATTCTTA--
TCACAGCCCCACATCGCACAAACATTTTGGCAGCCATGCACTTTCCAAGACCCACAATGAACAT
TTGCTGACCCCGCCAAATAGGAAGCCGCCGAGCTCGGAAAGGGTTCCTTCAAGTACGCATGGG
TTCTTGACAAGCTCAAGGCCGAGCGTGAGCGTGGTATCACCATCGATATCGCTCTCTGGAAGTT
CGAGACCAACGAGTACAATGTCACCGTCATTGGTTAGTATCCCTGTCCACACGATGTACTATGC
ATCTGAATGT-
ATACTAACATGGCAACACAGATGCTCCCGGTCACCGTGATTTCATCAATGCTGCCTTTTGGTATG
TAGCC-CATCTACCTCGACAC-GCCTCAATACGACAACCCCCCGCAACTCGACAAC--
GACGTTCTCAACAAGTGCTTGCTTGGAAACAAGGGAAAGACTTGATACTGACCGGTCCCTGATA
GGCAAACCATCTCTGGCGAGCACGGTCTCGACAGCAATGGAGTGTACGTACCCTTTCCTTGGCT
ACTTGCTTTCCCACGAACATCTCAGCTAACACTCG-
TGGTTGTTCAGCTACAACGGTACCTCCGAGCTCCAGCTCGAGCGCATGAGCGTCTACTTCAACG
AGGCTTCCGGCAACAAGTACGTTCCTCGTGCCGTCCTCGTCGATCTCGAGCCCGGTACCATGGA
TGCCGTCCGCGCCGGTCCTTTCGGTCAGCTCTTCCGCCCTGACAAC-
TCGTCTTCGGTCA??????????
>E52
????????TTATAGAGTTTTCTAAACTCCCAACCCATGTGAACTTACC-
ATTGTTGCCTCGGCAGAAGCTGCTCGGT-
ACACCCTACCTTGGAACGGCCTACCCTGTAGCGCCTTACCCTGGAACGGCTTACCCTGTAACGG
CTGCCGGTGGACTACCAAACTCTTGTTATTTTATTGTAATCTGAGCGTCTTATTTTAATAAGTCAA
AACTTTCAACAACGGATCTCTTGGTTCTGGCATCGATGAAGAACGCAGCGAAATGCGATAAGTA
ATGTGAATTGCAGAATTCAGTGAATCATCGAATCTTTGAACGCACATTGCGCCCATTAGTATTCT
AGTGGGCATGCCTGTTCGAGCGTCATTTCAACCCTTAAGCCTAGCTTAGTGTTGGGAGCCTACT
G--
CTTTTACTAGCTGTAGCTCCTGAAATACAACGGCGGATCTGCGATATCCTCTGAGCGTAGTAAAT
TTTTATCTCGCTTTTGACTGGAGTTGCAGCGTCTTTAGCCGCTAAATCCCCC-AA-
TTTTTAATGGTTGACCTC??????????????CGCAATCCC-ATCATCC---TCATCATC--------
ATCATCATCACCACCT--CGCAAA----CG-TTGCCACACC-GGTGCCGAAAATCTGG--------
TTTTCGCACCTGCC-CATTTTCCCAG--ACACTTACCCC--------
GCCGCACGACCCCGCGGTGCAAACGAAAAATTTCTTA--

TCATAGCCCCACATCACACAAACATTTTGGCAGCCACGCACTTTGCATGACCCACAATGAACAA
TTGCTGACCCCGCCAAATAGGAAGCCGCCGAGCTCGGAAAGGGTTCCTTCAAGTACGCATGGG
TTCTTGACAAGCTCAAGGCCGAGCGTGAGCGTGGTATCACCATTGATATCGCTCTCTGGAAGTT
CGAGACCAACGAGTACAATGTCACCGTCATTGGTTAGTATCCCTGTCCACAACATGTGTCATGT
CTCCAAACTCAAGACTAACCTTGCAATACAGACGCTCCCGGTCA???????????????TGCTGCCTTC
TGGTATGTAGCC-CATCTACCTCGACGC-GCCTCAATACGACACCCCCGGGCATCACGACAAC-
-GACATTCTCAAC---
TGCTTGTTTGGAACCATACGAAAGACTTGATACTGACCGGTCTATGATAGGCAAACCATCTCTG
GCGAGCACGGTCTCGACAGCAATGGAGTGTACGTACCCTTTCTCTGGCTACCCGCGTTCTCGTG
AACATGTCAGCTAACAGTCG-
TGCTTGTTTAGCTACAACGGTACCTCCGAGCTCCAGCTCGAGCGCATGAGCGTCTACTTCAACG
AGGCTTCCGGCAACAAGTACGTTCCTCGTGCCGTCCTCGTCGATCTCGAGCCCGGTACCATGGA
TGCCGTCCGCGCCGGTCCTTTCGGCCAGCT???????????????????????????????????????
>E53
AGGGATCATTATAGAGTTTTCTAAACTCCCAACCCATGTGAACTTACC-
ATTGTTGCCTCGGCAGAAGCTGCTCGGT-
ACACCCTACCTTGGAACGGCCTACCCTGTAGCGCCTTACCCTGGAACGGCTTACCCTGTAACGG
CTGCCGGTGGACTACCAAACTCTTGTTATTTTATTGTAATCTGAGCGTCTTATTTTAATAAGTCAA
AACTTTCAACAACGGATCTCTTGGTTCTGGCATCGATGAAGAACGCAGCGAAATGCGATAAGTA
ATGTGAATTGCAGAATTCAGTGAATCATCGAATCTTTGAACGCACATTGCGCCCATTAGTATTCT
AGTGGGCATGCCTGTTCGAGCGTCATTTCAACCCTTAAGCCTAGCTTAGTGTTGGGAGCCTACT
G--
CTTTTACTAGCTGTAGCTCCTGAAATACAACGGCGGATCTGCGATATCCTCTGAGCGTAGTAAAT
TTTTATCTCGCTTTTGACTGGAGTTGCAGCGTCTTTAGCCGCTAAATCCCCC-AA-
TTTTTAATGGTTGACCTC??????GTCATCCTCGCAATCCC-ATCATCCTCATCATCA-----------
TCATCATCACCACCT--CGCAAA----CG-TTGCCACACC-GGTGCCGAAAATCTGG--------
TTTTCGCACCTGCC-CATTTTCCCAG--ACACTTACCCC--------
GCCGCACGACCCCGCGGTGCAAACGAAAAATTTCTTA--
TCATAGCCCCACATCACACAAACATTTTGGCAGCCACGCACTTTGCATGACCCACAATGAACAA
TTGCTGACCCCGCCAAATAGGAAGCCGCCGAGCTCGGAAAGGGTTCCTTCAAGTACGCATGGG
TTCTTGACAAGCTCAAGGCCGAGCGTGAGCGTGGTATCACCATTGATATCGCTCTCTGGAAGTT
CGAGACCAACGAGTACAATGTCACCGTCATTGGTTAGTATCCCTGTCCACAACATGTGTCATGT
CTCCAAACTCAAGACTAACC???????????????????????????????????????TGCTGCCTTCTGGTATG
TAGCC-CATCTACCTCGACGC-GCCTCAATACGACACCCCCGGGCATCACGACAAC--
GACATTCTCAAC---
TGCTTGTTTGGAACCATACGAAAGACTTGATACTGACCGGTCTATGATAGGCAAACCATCTCTG
GCGAGCACGGTCTCGACAGCAATGGAGTGTACGTACCCTTTCTCTGGCTACCCGCGTTCTCGTG
AACATGTCAGCTAACAGTCG-
TGCTTGTTTAGCTACAACGGTACCTCCGAGCTCCAGCTCGAGCGCATGAGCGTCTACTTCAACG
AGGCTTCCGGCAACAAGTACGTTCCTCGTGCCGTCCTCGTCGATCTCGAGCCCGGTACCATGGA
TGCCGTCCGCGCCGGTCCTTTCGGCCAGCTCTTCCGCCCTGACAACTTCGTCTTCGGTCAGTCCG
?????
>E55
AGGGATCATTATAGAGTTTTCTAAACTCCCAACCCATGTGAACTTACC-

ATTGTTGCCTCGGCAGAAGCTGCTCGGT-
ACACCCTACCTTGGAACGGCCTACCCTGTAGCGCCTTACCCTGGAACGGCTTACCCTGTAACGG
CTGCCGGTGGACTACCAAACTCTTGTTATTTTATTGTAATCTGAGCGTCTTATTTTAATAAGTCAA
AACTTTCAACAACGGATCTCTTGGTTCTGGCATCGATGAAGAACGCAGCGAAATGCGATAAGTA
ATGTGAATTGCAGAATTCAGTGAATCATCGAATCTTTGAACGCACATTGCGCCCATTAGTATTCT
AGTGGGCATGCCTGTTCGAGCGTCATTTCAACCCTTAAGCCTAGCTTAGTGTTGGGAGCCTACT
G--
CTTTTACTAGCTGTAGCTCCTGAAATACAACGGCGGATCTGCGATATCCTCTGAGCGTAGTAAAT
TTTTATCTCGCTTTTGACTGGAGTTGCAGCGTCTTTAGCCGCTAAATCCCCC-AA-
TTTTTAATGGTTGACCTC?????????ATCCTCGCAATCCC-ATCATCCTCATCATCA-----------
TCATCATCACCACCT--CGCAAA----CG-TTGCCACACC-GGTGCCGAAAATCTGG--------
TTTTCGCACCTGCC-CATTTTCCCAG--ACACTTACCCC--------
GCCGCACGACCCCGCGGTGCAAACGAAAAATTTCTTA--
TCATAGCCCCACATCACACAAACATTTTGGCAGCCACGCACTTTGCATGACCCACAATGAACAA
TTGCTGACCCCGCCAAATAGGAAGCCGCCGAGCTCGGAAAGGGTTCCTTCAAGTACGCATGGG
TTCTTGACAAGCTCAAGGCCGAGCGTGAGCGTGGTATCACCATTGATATCGCTCTCTGGAAGTT
CGAGACCAACGAGTACAATGTCACCGTCATTGGTTAGTATCCCTGTCCACAACATGTGTCATGT
CTCCAAACTCAAGACTAACCTTGCAATACAGACGCTCCCGGTCACCGTGATTTCATCAATGCTG
CCTTCTGGTATGTAGCC-CATCTACCTCGACGC-
GCCTCAATACGACACCCCCGGGCATCACGACAAC--GACATTCTCAAC---
TGCTTGTTTGGAACCATACGAAAGACTTGATACTGACCGGTCTATGATAGGCAAACCATCTCTG
GCGAGCACGGTCTCGACAGCAATGGAGTGTACGTACCCTTTCTCTGGCTACCCGCGTTCTCGTG
AACATGTCAGCTAACAGTCG-
TGCTTGTTTAGCTACAACGGTACCTCCGAGCTCCAGCTCGAGCGCATGAGCGTCTACTTCAACG
AGGCTTCCGGCAACAAGTACGTTCCTCGTGCCGTCCTCGTCGATCTCGAGCCCGGTACCATGGA
TGCCGTCCGCGCCGGTCCTTTCGGCCAGCTCTTCCGCCCTGACAACTT??????????????????????
>JY1
AGGGATCATTATAGAGTTTTCTAAACTCCCAACCCATGTGAACTTACC-
ATTGTTGCCTCGGCAGAAGCTGCTCGGT-
GCACCTTACCTTGGAACGGCCTACCCTGTAGCGCCTTACCCTGGAACGGCTTACCCTGTAGCGG
CTGCCGGCGGACTACCAAACTCTTGTTATTTTATTGTAATCTGAGCGTCTTATTTTAATAAGTCAA
AACTTTCAACAACGGATCTCTTGGTTCTGGCATCGATGAAGAACGCAGCGAAATGCGATAAGTA
ATGTGAATTGCAGAATTCAGTGAATCATCGAATCTTTGAACGCACATTGCGCCCATTAGTATTCT
AGTGGGCATGCCTGTTCGAGCGTCATTTCAACCCTTAAGCCTAGCTTAGTGTTGGGAGCCTACT
G--
CTTTTACTAGCTGTAGCTCCTGAAATACAACGGCGGATCTGCGATATCCTCTGAGCGTAGTAA-
TTTTTATCTCGCTTTTGACTGGAGTTGCAGCGTCTTTAGCCGCTAAACCCCCC-AA-
TTTTTAATGGTTGACCTCAGGTTAGTCATCCTCGCAATCCC-ATCATCC------TCATC--------
TTCATCACCATCACCT--CGCAAA----CA-TTTCCACACC-GGTGCCGAAAATCT-G--------
TTTTCGCACCTGCC-CATTTTCCCAG--ACACTTACCCC--------
GCCGCACGACCCCGCGGTGCAAACGAAAAATTTCTTA--TCATAGCCCCACATCAC--
AAACATTTTGGCAGCCACGCACTCTGCATGACCCACAATGAACAATTGCTGACCCCGCCAAATA
GGAAGCCGCCGAGCTCGGAAAGGGTTCCTTCAAGTACGCATGGGTTCTTGACAAGCTCAAGGC
CGAGCGTGAGCGTGGTATCACCATTGATATCGCTCTCTGGAAGTTCGAGACCAACGAGTACAAT

GTCACCGTCATTGGTTAGTATCCCTGCCCACAACATGTGTCATGTCTCCGAACTCGAGACTAACC
TTACAATACAGACGCTCCCGGTCACCGTGATTTCATCAATGCTGCCTTCTGGTATGTAGCC-
CATCTACCCCGACGC-GTCTCAATACGACA-CCCCCGGCAACTCGACAAC--GACGTTCTCAAC-
--
TGCTTGGTTGAAACCAAATGAAAGACTTGATACTGATCGGTCTCTGATAGGCAAACCATCTCTG
GCGAGCACGGTCTCGACAGCAATGGAGTGTACGTACCCTTTCCCTGGCTACTCGCTTTCTCGTG
AACATGTCAGCTAACACTCG-
TGCTTGTTCAGCTACAACGGTACCTCCGAGCTCCAGCTCGAGCGCATGAGCGTCTACTTCAACG
AGGCTTCCGGCAACAAGTACGTTCCTCGTGCCGTCCTCGTCGATCTCGAGCCCGGTACCATGGA
TGCCGTCCGCGCCGGTCCTTTCGGTCAGCTCTTCCGCC?????????????????????????????????
>L19_1
????????????????????????CTCCCAACCCATGTGAACTTACC-
ATTGTTGCCTCGGCAGAAGCTGCTCGGT-
GCACCCTACCTTGGAACGGCCTACCCTGTAGCGCCTTACCCTGGAACGGCTTACCCTGTAACGG
CTGCCGGTGGACTACCAAACTCTTGTTATTTTATTGTAATCTGAGCGTCTTATTTTAATAAGTCAA
AACTTTCAACAACGGATCTCTTGGTTCTGGCATCGATGAAGAACGCAGCGAAATGCGATAAGTA
ATGTGAATTGCAGAATTCAGTGAATCATCGAATCTTTGAACGCACATTGCGCCCATTAGTATTCT
AGTGGGCATGCCTGTTCGAGCGTCATTTCAACCCTTAAGCCTAGCTTAGTGTTGGGAGCCTACT
G--
CTTTTGCTAGCTGTAGCTCCTGAAATACAACGGCGGATCTGCGATATCCTCTGAGCGTAGTAAAT
TTTTATCTCGCTTTTGACTGGAGTTGCAGCGTCTTTGGCCGCTAAATCCCCC-AA-
TTTTTAATGGT?????????????????TCCTCGCAATCCC-ATCATCC------TCATC--------
CTCATCATCATCACTT--CGCAAA----CATTTCCCACACC-GGTGTCGAAAATCTGG--------
TTTTCGCACCTGCC-CATTTTCTCAG--ACACTTACCCC--------
GCCGCACGACCCCGCGGTGCAAACGAAAAATTTCTTA--
TCACAGCCCCACATCACACAAACATTTTGGCAGCCACGCACCTTGCATGACCCACAATGAACAA
TTGCTGACCCCGCCAAATAGGAAGCCGCCGAGCTCGGAAAGGGTTCCTTCAAGTACGCATGGG
TTCTTGACAAGCTCAAGGCCGAGCGTGAGCGTGGTATCACCATTGATATCGCTCTCTGGAAGTT
CGAGACCAACGAGTACAATGTCACCGTCATTGGTTAGTATCCCTGCCCACAATATGTGTCATGT
CTCTGAACTCAAGACTAACCTTGCAATACAGACGCTCCCGGTCACCGTGATTTCATCAA????????
?????????????C-CATCTACCTCGACGC-GCCTCAATACGACA-CCCCCGGCAACTCGACAAC--
GACATTCTCAAC---
TGCTTGTTTGGAACCATACGAAAGACTTCATACTGACCGGTCTATGATAGGCAAACCATCTCTGG
CGAGCACGGTCTCGACAGCAATGGAGTGTACGTACCCTTGATCTCGCTACTCGCTTTCTCGTGA
ACATGTCAGCTAACAGTCG-
TGCTTGTTCAGCTACAACGGTACCTCCGAGCTCCAGCTCGAGCGCATGAGCGTCTACTTCAACG
AGGCTTCCGGCAACAAGTACGTTCCTCGTGCCGTCCTCGTCGATCTCGAGCCCGGTACCATGGA
TGCCGTCCGCGCCGGTCCTTTCGGTCAGCTCTTCCGCCCTGACAACTTCGTCTTCGGTCAGTCCG
GTGCC
>M13
AGGGATCATTATAGAGTTTTCTAAACTCCCAACCCATGTGAACTTACC-
ATTGTTGCCTCGGCAGAAGCTGCTCGGT-
GCACCTTACCTTGGAACGGCCTACCCTGTAGCGCCTTACCCTGGAACGGCTTACCCTGTAGCGG
CTGCCGGTGGACTACCAAACTCTTGTTATTTTATTGTGATCTGAGCGTCTTATTTTAATAAGTCAA

AACTTTCAACAACGGATCTCTTGGTTCTGGCATCGATGAAGAACGCAGCGAAATGCGATAAGTA
ATGTGAATTGCAGAATTCAGTGAATCATCGAATCTTTGAACGCACATTGCGCCCATTAGTATTCT
AGTGGGCATGCCTGTTCGAGCGTCATTTCAACCCTTAAGCCTAGCTTAGTGTTGGGAGCCTACT
G--
CTTTTGCTAGCTGTAGCTCCTGAAATACAACGGCGGATCTGCGATATCCTCTGAGCGTAGTAA-
TTTTTATCTCGCTTTTGACTGGAGTTGCAGCGTCTTTAGCCGCTAAACCCCCC-AA-
TTTTTAATGGTTGACCTCAGGTTAGTCATCCTCGCAATCCC-ATCAT-----------------------
---CATCATCACCT--CGCAAA----CA-TTTCCACGCC-GGTGCCGAAAATCTGG--------
TTTTCGCACCTGCC-CATTTTCCCGG--ACACTTACCCC--------
GCCGCACGACCCCGCGGTGCAAACGAAAAATTTCTTA--
TCATGGCCCCACATCACACAAACATTTTGGCAGCCACGCACTTTGCAAGACCCACAACGAACAA
TTGCTGACCCCGCCAAATAGGAAGCCGCCGAGCTCGGAAAGGGTTCCTTCAAGTACGCATGGG
TTCTTGACAAGCTCAAGGCCGAGCGTGAGCGTGGTATCACCATTGATATCGCTCTCTGGAAGTT
CGAGACCAACGAGTACAATGTCACCGTCATTGGTTAGTATCCCTGTCCACAACATGCATCATGT
CTCCGAACTCAAGACTAACCTTGCAATACAGATGCTCCCGGTCACCGTGATTTCATCAATGCTG
CCTTCTGGTATGTAGCC-CATCTACCCCGACGC-GTCTCAATACGAAA-
CCCCCGGCAACTCGACAAC--GACGTTCTCAAC---
TGCTTGGTTGAAACCAAATGAAAGACTTGATACTGATCGGTCTCTGATAGGCAAACCATCTCTG
GCGAGCACGGTCTCGACAGCAATGGAGTGTACGTACCCTTTCCCTGGCTACTCGCTTTCTCGTG
AACATGTCAGCTAACACTCG-
TGCTTGTTCAGCTACAACGGTACCTCCGAGCTCCAGCTCGAGCGCATGAGCGTCTACTTCAACG
AGGCTTCCGGCAACAAGTACGTTCCTCGTGCCGTCCTCGTCGATCTCGAGCCCGGTACCATGGA
TGCCGTCCGCGCCGGTCCTTTCGGTCAGCT??
>M18
????????TTATAGAGTTTTCTAAACTCCCAACCCATGTGAACTTACC-
ATTGTTGCCTCGGCAGAAGCTGCTCGGT-
GCACCTTACCTTGGAACGGCCTACCCTGTAGCGCCTTACCCTGGAACGGCTTACCCTGTAGCGG
CTGCCGGTGGACTACCAAACTCTTGTTATTTTATTGTAATCTGAGCGTCTTATTTTAATAAGTCAA
AACTTTCAACAACGGATCTCTTGGTTCTGGCATCGATGAAGAACGCAGCGAAATGCGATAAGTA
ATGTGAATTGCAGAATTCAGTGAATCATCGAATCTTTGAACGCACATTGCGCCCATTAGTATTCT
AGTGGGCATGCCTGTTCGAGCGTCATTTCAACCCTTAAGCCTAGCTTAGTGTTGGGAGCCTACT
G--
CTTTTACTAGCTGTATCTCCTGAAATACAACGGCGGATCTGCGATATCCTCTGAGCGTAGTAA-
TTTTTATCTCGCTTTTGACTGGAGTTGCAGCGTCCTTAGCCGCTAAATCCCCC-
AATTTTTTAATGGTTGACCTCAGGTTAGTCATCCTCACAATCCC-ATCATCC-----------------
---TCATCATCATCACCT--CGCAAA----CA-TTTCCACACC-GGTGCCAAAAATCTGG--------
TTTTCGCACCTGCC-CATTTTCCCAG--ACACTTACCCC--------
GCCGCACGACCCCGCGGTGCAAACGAAAAATTTCTTA--
TCATAGCCCCACATCACACAAACATTTCGACAGCCGCGCACTTTGCAAGACCCACAATGAACAA
TTGCTGACCCCGCCAAATAGGAAGCCGCCGAGCTCGGAAAGGGTTCCTTCAAGTACGCATGGG
TTCTTGACAAGCTCAAGGCCGAGCGTGAGCGTGGTATCACCATTGATATCGCTCTCTGGAAGTT
CGAGACCAACGAGTACAATGTCACCGTCATTGGTTAGTATCCCTGTCCACAACATGTGTCATGT
CTCCGAACTCAAGACTAACCTTACAACACAGACGCTCCCGGTCACCGTGATTTCATCAATGCTG
CCTTCTGGTATGTAGCC-CATCTACCCCGACGC-GTCTCAATACGACA-

CCCCCGGCAACTCGACAAC--GACGTTCTCAAC---
TGCTTGGTTGAAACCAAATGAAAGACTTGATACTGACCGGTCTCTGATAGGCAAACCATCTCTG
GCGAGCACGGTCTCGACAGCAATGGAGTGTACGTACCCTTTCCCTGGCTACTCGCTTTCTCGTG
AACATGTCAGCTAACACTCG-
TGCTTGTTCAGCTACAACGGTACCTCCGAGCTCCAGCTCGAGCGCATGAGCGTCTACTTCAACG
AGGCTTCCGGCAACAAGTACGTTCCTCGTGCCGTCCTCGTCGATCTCGAGCCCGGTACCATGGA
TGCCGTCCGCGCCGGTCCTTTCGGTCAGCTC???
>M26
AGGGATCATTATAGAGTTTTCTAAACTCCCAACCCATGTGAACTTACC-
ATTGTTGCCTCGGCAGAAGCTGCTCGGT-
GCACCTTACCTTGGAACGGCCTACCCTGTAGCGCCTTACCCTGGAACGGCTTACCCTGTAGCGG
CTGCCGGCGGACTACCAAACTCTTGTTATTTTATTGTAATCTGAGCGTCTTATTTTAATAAGTCAA
AACTTTCAACAACGGATCTCTTGGTTCTGGCATCGATGAAGAACGCAGCGAAATGCGATAAGTA
ATGTGAATTGCAGAATTCAGTGAATCATCGAATCTTTGAACGCACATTGCGCCCATTAGTATTCT
AGTGGGCATGCCTGTTCGAGCGTCATTTCAACCCTTAAGCCTAGCTTAGTGTTGGGAGCCTACT
G--
CTTTTACTAGCTGTAGCTCCTGAAATACAACGGCGGATCTGCGATATCCTCTGAGCGTAGTAA-
TTTTTATCTCGCTTTTGACTGGAGTTGCAGCGTCTTTAGCCGCTAAACCCCCC-AA-
TTTTTAATGGTTGACCTCAGGTTAGTCATCCTCGCAATCCC-ATCAT------------------------
---CATCATCACCT--CGCAA-----CA-TTTCCACGCC-GGTGCCGAAAATCTGG--------
TTTTCGCACCTGCC-CATTTTCCCGG--ACACTTACCCC--------
GCCGCACGACCCCGCGGTGCAAACGAAAAATTTCTTA--
TCATGGCCCCACATCACACAAACATTTTGGCAGCCACGCACTTTGCAAGACCCACAACGAACAA
TTGCTGACCCCGCCAAATAGGAAGCCGCCGAGCTCGGAAAGGGTTCCTTCAAGTACGCATGGG
TTCTTGACAAGCTCAAGGCCGAGCGTGAGCGTGGTATCACCATTGATATCGCTCTCTGGAAGTT
CGAGACCAACGAGTACAATGTCACCGTCATTGGTTAGTATCCCTGTCCACAACATGCATCATGT
CTCCGAACTCAAGACTAACCTTGCAATACAGATGCTCCCGGTCACCGTGATTTCATCAATGCTG
CCTTCTGGTATGTAGCC-CATCTACCCCGACGC-GTCTCAATACGACA-
CCCCCGGCAACTCGACAAC--GACGTTCTCAAC---
TGCTTGGTTGAAACCAAATGAAAGACTTGATACTGATCGGTCTCTGATAGGCAAACCATCTCTG
GCGAGCACGGTCTCGACAGCAATGGAGTGTACGTACCCTTTCCCTGGCTACTCGCTTTCTCGTG
AACATGTCAGCTAACACTCG-
TGCTTGTTCAGCTACAACGGTACCTCCGAGCTCCAGCTCGAGCGCATGAGCGTCTACTTCAACG
AGGCTTCCGGCAACAAGTACGTTCCTCGTGCCGTCCTCGTCGATCTCGAGCCCGGTACCATGGA
TGCCGTCCGCGCCGGTCCTTTCGGTCAGCT??
>N_cubana_CBS_600_96
AGGGATCATTATAGAGTTTTCTAAACTCCCAACCCATGTGAACTTACCTTTTGTTGCCTCGGCAG
AAGTT---
ATAGGTCTTCTTATAGCTGCTGCCGGTGGACCATTAAACTCTTGTTATTTTAT-
GTAATCTGAGCGTCTTATTTTAATAAGTCAAAACTTTCAACAACGGATCTCTTGGTTCTGGCATCG
ATGAAGAACGCAGCGAAATGCGATAAGTAATGTGAATTGCAGAATTCAGTGAATCATCGAATCT
TTGAACGCACATTGCGCCCATTAGTATTCTAGTGGGCATGCCTGTTCGAGCGTCATTTCAACCCT
TAAGCCTAGCTTAGTGTTGGGAATCTACTT--
CTCTTAGGAGTTGTAGTTCCTGAAATACAACGGCGGATTTGTAGTATCCTCTGAGCGTAGTAA-

TTTTTTTCTCGCTTTTGTTAGGTGCTATAACTCCC--AGCCGCTAAACCCCC--AA-
TTTTTTGTGGTTGACCTCAGGTTAGTCATCTATTGATTCCC-ATCATCA---------------------
-TTCCCCTTCACTC----CAGCGTCATGATTTTCAACCTACGCGTTGAAAATTA----------
TTTTCGCTCCTTCCACACTTTTTTCG--
CTGGTTACCCCGCCGCGAGGCCGCACGACCCCGCGGTGCAAACGAAAAATTTCTTA--
TCACAGCCCCACCTTTCACAAGCA--------
ACCATGCATTGCTCATGACCCACTTTGAACAATTGCTAATGCCTTCATACAGGAAGCCGCCGAG
CTCGGTAAGGGTTCCTTCAAGTACGCCTGGGTTCTTGACAAGCTCAAGGCCGAGCGTGAGCGTG
GTATCACCATCGATATCGCTCTCTGGAAGTTCGAGACCAACGAGTACAATGTCACCGTCATTGG
TTAGTACCCCTCCACCTACCATGTGCTGCTCCATAAGACACTTGACTAACCTTGCTTCATAGACG
CTCCCGGTCACCGTGATTTCATCAATGCTGCCTTCTGGTATGTAACC-
TGTCTGTCTCGACACGGCCTCAATACGACG-TTTTTCGTGCCTGCA-----
CGACAGCCCCGAACAGTATTAGGTCAAGATAGAGGGA---
ACATGATGCTAATAGGTCATTGATAGGCAAACCATCTCTGGCGAGCACGGTCTCGACAGCAATG
GAGTGTATGTACTATTTTCAATTCCTCCTGCTTCCTGTTGAGCTTGTAGGCTGAC--
TCGATGGCCATTTAGCTACAACGGTACCTCCGAGCTCCAGCTCGAGCGTATGAGCGTCTACTTC
AACGAGGCTTCCGGCAACAAGTACGTTCCTCGTGCCGTCCTCGTCGATCTCGAGCCCGGTACCA
TGGATGCCGTCCGCGCCGGTCCCTTCGGCCAGCTCTTCCGCCCTGACAACTTCGTCTTCGGTCA
GT????????
>N_protearum_CBS_114178
AGGGATCATTATAGAGTTTTCTAAACTCCCAACCCATGTGAACTTACCTTTTGTTGCCTCGGCAG
AAGTT---
ATAGGTCTTCTTATAGCTGCTGCCGGTGGACCATTAAACTCTTGTTATTTTAT-
GTAATCTGAGCGTCTTATTTTAATAAGTCAAAACTTTCAACAACGGATCTCTTGGTTCTGGCATCG
ATGAAGAACGCAGCGAAATGCGATAAGTAATGTGAATTGCAGAATTCAGTGAATCATCGAATCT
TTGAACGCACATTGCGCCCATTAGTATTCTAGTGGGCATGCCTGTTCGAGCGTCATTTCAACCCT
TAAGCCTAGCTTAGTGTTGGGAATCTACTT--
CTCTTAGGAGTTGTAGTTCCTGAAATACAACGGCGGATTTGTAGTATCCTCTGAGCGTAGTAA-
TTTTTTTCTCGCTTTTGTTAGGTGCTATAACTCCC--AGCCGCTAAACCCCC--AA-
TTTTCTGTGGTTGACCTC?????AGTCATTTATTGATTCCC-ATCATCC----------------------
--CCCTTCACTT----CAGCATCATAATTTTCAACCTACGTGTTGAAAATTA----------
TTTTCGCTCCTTCCACACTTTTTTTCG-
CTGGTTACCCCGCCGCGAGGCCGCACGACCCCGCGGTGCAAACGAAAAATTTCTTA--
TCACAGCCCCACCTTGCACAAGCA--------
ACCATGCATTGCTCATGACCCACTTTGAACAATTGCTAATGCCTTCATACAGGAAGCCGCCGAG
CTCGGTAAGGGTTCCTTCAAGTACGCCTGGGTTCTTGACAAGCTCAAGGCCGAGCGTGAGCGTG
GTATCACCATCGATATCGCTCTCTGGAAGTTCGAGACCAACGAGTACAATGTCACCGTCATTGG
TTAGTACCCCTCCACCTACCATGTGCTGCTCCATAAGACACTTGACTAACCTTGCTTCATAGACG
CTCCCGGTCACCGTGATTTCATCAATGCTGCCTTCTGGTATGTTACC-
TGTCTGCCTCGACACGGCCTTACTACGACG-TTTTTCGTGCCTGCA-----
CGACGGCCCCGAAAAGTAATAGGTCAAGATAGAGGGA---
ACATGATACTAATAGGTCATTTATAGGCAAACCATCTCTGGCGAGCACGGTCTCGACAGCAATG
GAGTGTATGTACTAACTTCAATTCCTCCTGCTTCCTGTTGAGTTTGTAGGCTGAC--
TCGATGGCCATTTAGCTACAACGGTACCTCCGAGCTCCAGCTCGAGCGTATGAGCGTCTACTTC

AACGAGGCTTCCGGCAACAAGTACGTTCCTCGTGCCGTCCTCGTCGATCTCGAGCCCGGTACCA
TGGATGCCGTCCGCGCCGGTCCCTTCGGCCAGCTCTTCCGCCCTGACAACTTCGTCTTCGGTCA
GTCCGGTGCT
>P_adusta_ICMP_6088
??????CATTATAGAGTTTTCTAAACTCCCAACCCATGTGAACTTACC-
ATTGTTGCCTCGGCAGAAGCTGCTCGGT-
GCACCTTACCTTGGAACGGCCTACCCTGTAGCGCCTTACCCTGGAACGGCTTACCCTGCAACGG
CTGCCGGTGGACTACCAAACTCTTGTTATTTTATGGTTATCTGAGCGTCTTATTTTAATAAGTCAA
AACTTTCAACAACGGATCTCTTGGTTCTGGCATCGATGAAGAACGCAGCGAAATGCGATAAGTA
ATGTGAATTGCAGAATTCAGTGAATCATCGAATCTTTGAACGCACATTGCGCCCATTAGTATTCT
AGTGGGCATGCCTGTTCGAGCGTCATTTCAACCCTTAAGCCTAGCTTAGTGTTGGGAGCCTACT
G--
CTTTTGCTAGCTGTAGCTCCTGAAATACAACGGCGGATCTGCGATATCCTCTGAGCGTAGTAA-
TTTTTATCTCGCTTTTGACTGGAGTTGCAGCGTCTTTAGCCGCTAAACCCCCC-AA-
TTTTTAATGGTTGACCTCAGGTTAGTCATGCTCACA-TCCC-ATCATCC------TCATC---------
--ATCATCATCGCCT--CGCAAA----CA-TTTTCCAACC-GGTGCCGAAATTCT-G--------
TTTTCGCACCTGCC-CATTTTTCCAG--ACACTTATCCC--------
GCCGCACGACCCCGCGGTGCAAACGAAAAATTTCTTATCTCACAGCCCCACTTCACAC-
AACATTTTGGCAGCCACGCACTTTGCATGACCCACAATGAACAATTGCTGACCCCGCCAAATAG
GAAGCCGCCGAGCTCGGAAAGGGTTCCTTCAAGTACGCATGGGTTCTTGACAAGCTCAAGGCC
GAGCGTGAGCGTGGTATCACCATTGATATCGCTCTCTGGAAGTTCGAGACCAACGAGTACAATG
TCACCGTCATTGGTTAGTATCCCTGTCCATAGAAAGTATCATGTGTCCGAACTC-
AGACTAACATCGCAATACAGACGCTCCCGGTCACCGTGATTTCATCAATGCTGCTTTCTGGTATG
TAGCC-CAACTACCTCGACAC-GCCTCAATACGACA-CCCTCCACAACTCGACGAC--
GGCATTCTCGGC---
TACTTGGTTGGAACCAAACGAAAGACTTGATACTGACCGGTCTCTGATAGGCAAACCATCTCTG
GCGAGCACGGTCTCGACAGCAATGGAGTGTACGTACCGCTTCCTTGGCTACTTGCTTTCCCACG
GACTTGTTAGCTAACACTCG-
TGCTTGCTCAGCTACAACGGCACCTCCGAGCTCCAGCTCGAGCGCATGAGCGTCTACTTCAACG
AGGCTTCCGGCAACAAGTACGTTCCTCGTGCTGTCCTCGTCGATCTCGAGCCCGGTACCATGGA
CGCCGTCCGCGCCGGTCCTTTCGGCCAGCTCTTCCGCCCTGACAACTTCGTCTTCGGTCAGTCC
GGTGCC
>P_adusta_MFLUCC_10_0146
??????CATTATAGAGTTTTCTAAACTCCCAACCCATGTGAACTTACC-
ATTGTTGCCTCGGCAGAAGCTGCTCGGT-
GCACCCTACCTTGGAACGGCCTACCCTGTAGCGCCTTACCCTGGAACGGCTTACCCTGCAACGG
CTGCCGGTGGACTACCAAACTCTTGTTATTTTATGGTTATCTGAGCGTCTTATTTTAATAAGTCAA
AACTTTCAACAACGGATCTCTTGGTTCTGGCATCGATGAAGAACGCAGCGAAATGCGATAAGTA
ATGTGAATTGCAGAATTCAGTGAATCATCGAATCTTTGAACGCACATTGCGCCCATTAGTATTCT
AGTGGGCATGCCTGTTCGAGCGTCATTTCAACCCTTAAGCCTAGCTTAGTGTTGGGAGCCTACT
G--
CTTTTGCTAGCTGTAGCTCCTGAAATACAACGGCGGATCTGCGATATCCTCTGAGCGTAGTAA-
TTTTTATCTCGCTTTTGACTGGAGTTGCAGCGTCTTTAGCCGCTAAACCCCCC-AA-
TTTTTAATGGTTGACCTCAGGTTAGTCATGCTCACA-TCCC-ATCATCC------TCATC---------

--ATCATCATCGCCT--CGCAAA----CA-TTTTCCAACC-GGTGCCGAAATTCT-G--------
TTTTCGCACCTGCC-CATTTTTCCAG--ACACTTACCCC--------
GCCGCACGACCCCGCGGTGCAAACGAAAAATTTCTTATCTCACAGCCCCACTTCACAC-
AACATTTTGGCAGCCACGCACTTTGCATGACCCACAATGAACAATTGCTGACCCCGCCAAATAG
GAAGCCGCCGAGCTCGGAAAGGGTTCCTTCAAGTACGCATGGGTTCTTGACAAGCTCAAGGCC
GAGCGTGAGCGTGGTATCACCATTGATATCGCTCTCTGGAAGTTCGAGACCAACGAGTACAATG
TCACCGTCATTGGTTAGTATCCCTGTCCATAGAAAGTATCATGTGTCCGAACTC-
AGACTAACATCGCAATACAGACGCTCCCGGTCACCGTGATTTCATCAATGCTGCTTTCTGGTATG
TAGCC-CAACTACCTCGACAC-GCCTCAATACGACA-CCCTCCACAACTCGACGAC--
GGCATTCTCGGC---
TACTTGGTTGGAACCAAACGAAAGACTTGATACTGACCGGTCTCTGATAGGCAAACCATCTCTG
GCGAGCACGGTCTCGACAGCAATGGAGTGTACGTACCGCTTCCTTGGCTACTTGCTTTCCCACG
GACTTGTTAGCTAACACTCG-
TGCTTGCTCAGCTACAACGGCACCTCCGAGCTCCAGCTCGAGCGCATGAGCGTCTACTTCAACG
AGGCTTCCGGCAACAAGTACGTTCCTCGTGCTGTCCTCGTCGATCTCGAGCCCGGTACCATGGA
CGCCGTCCGCGCCGGTCCTTTCGGCCAGCTCTTCCGCCCTGACAACTTCGTCTTCGGTCAGTCC
GGTGCC
>P_aggestorum_LC6301
???????????????????????????????ACCCATGTGAACTTACC-
ATTGTTGCCTCGGCAGAAGCTGCTCGGT-
ATACCCTACCTTGGAACGGCCTACCCTGTAGCGCCTTACCCTGGAACGGCTTACCCTGCAACGG
CTGCCGGTGGACTACTAAACTCTTGTTATTTTATTGTAATCTGAGCGTCTTATTTTAATAAGTCAA
AACTTTCAACAACGGATCTCTTGGTTCTGGCATCGATGAAGAACGCAGCGAAATGCGATAAGTA
ATGTGAATTGCAGAATTCAGTGAATCATCGAATCTTTGAACGCACATTGCGCCCATTAGTATTCT
AGTGGGCATGCCTGTTCGAGCGTCATTTCAACCCTTAAGCCTAGCTTAGTGTTGGGAGCCTACT
G--
CTTTTGCTAGCTGTAGCTCCTGAAATACAACGGCGGATCTGCGATATCCTCTGAGCGTAGTAA-
TTTTTATCTCGCTTTTGACTGGAGTTGCAGCGTCTTTAGCCGCTAAACCCCCC-AA-
TTTTTAATGGTTGACCTCAGGTTAGTCATGCTCACAGTCCC-ATCATCC------TCATC---------
--ATAATCATCGACT--CGCAAA----CA-TTTTCCAACC-GGTGCCGAAATTCT-G--------
TTTTCGCACCTGCC-CATTTTCCTAG--ACACTTACCCC--------
GCCGCACGACCCCGCGGTGCAAACGAAAAATTTCTTA--TCACAGCCCCACTTCACAC-
AACATTTTGGCAGCCACGCACTTTGCATGACCCACAATGAACAATTGCTGACCCCGCCAAATAG
GAAGCCGCCGAGCTCGGAAAGGGTTCCTTCAAGTACGCATGGGTTCTTGACAAGCTCAAGGCC
GAGCGTGAGCGTGGTATCACCATTGATATCGCTCTCTGGAAGTTCGAGACCAACGAGTACAATG
TCACCGTCATTGGTTAGTATCCCTGTCCACAGAAAGTATCATGTGTCCGAACTC-
AGACTAACATCGCAATACAGACGCTCCCGGTCACCGTGATTTCATCAATGCTGCCTTCTGGTAT
GTAGCC-CATCTACCTCGGCAC-GCCTCAATACGACA-CCCTCCGCAACTCGACGAC--
GACATTCTCGGC---
TACTTGGTTGGAACCGAACGAAAGACTTGATACTGACCGGTCTCTGATAGGCAAACCATCTCTG
GCGAGCACGGTCTCGACAGCAATGGAGTGTACGTACCATTTCCTTGCCTACTTGCTTTCCCACG
AACATGTTAGCTAACACTCG-
TGCTTGCTCAGCTACAACGGCACCTCCGAGCTCCAGCTCGAGCGCATGAGCGTCTACTTCAACG
AGGCGTCCGGCAACAAGTACGTTCCTCGTGCCGTCCTCGTCGATCTCGAGCCCGGTACCATGGA

CGCCGTCCGCGCCGGTCCTTTCGGCCAGCTCTTCCGCCCTGACAACTTCGTCTTCGGTCAGTCC
GGTGCC
>P_anacardiacearum_IFRDCC_2397
??????CATTATAGAGTTTTCTAAACTCCCAACCCATGTGAACTTACCAATTGTTGCCTCGGCAGAG
GCTACCCGGT---
ACCTTACCTTGGTGCGGCCTACCCTGTAGCGCCTTACCCTGGAACGGGCTACCCTGTAGCGGCT
GCCGGTGGACTACCAAACTCTTGTTATTTTATGGTTATCTGAGCGTCTTATTTTAATAAGTCAAAA
CTTTCAACAACGGATCTCTTGGTTCTGGCATCGATGAAGAACGCAGCGAAATGCGATAAGTAAT
GTGAATTGCAGAATTCAGTGAATCATCGAATCTTTGAACGCACATTGCGCCCATTAGTATTCTAG
TGGGCATGCCTGTTCGAGCGTCATTTCAACCCTTAAGCCTAGCTTAGTGTTGGGAGCCTACTG--
CTTTTACTAGCTGTAGCTCCTGAAATACAACGGCGGATCTGCGATATCCTCTGAGCGTAGTAATT
TTTTTTCTCGCTTTTGACTGGAGTTGCAGCGTCCTTAGCCGCTAAACCCCCC-AA-
TTTTTAATGG-TGACCTCAGGTTAGTCATCCTCACAATCCC-ATCAGCA-------CATT---------
----CATGGCCTCCC--ATCGAA----GA-TCTCCGACCC-GATGTCGAAAATAT----
TTTCGTTTTTCGCACCTTCC-CACATTTTC----
CCACTTACCCCGCCGCATGGCCGCACGACCCCGCGGTGCAAACGAAAAAATTCTTA--T--
CAGCCCCACCTCGCACAAACATTTTGGCAGCCACGCACTTT-
CATGACCCATGATGAGCCATTGCTGACCCCGCCAAACAGGAAGCCGCCGAGCTCGGAAAGGGT
TCCTTCAAGTACGCCTGGGTTCTTGACAAGCTCAAGGCCGAGCGTGAGCGTGGTATCACCATCG
ATATCGCTCTCTGGAAGTTCGAGACCAACGAGTACAATGTCACCGTCATTGGTTAGTATCCCTGT
CCACAACAACTCTCATGCATCCGAACTC-
AGACTAACATGACAATACAGACGCTCCCGGTCACCGTGACTTCATCAATGCTGCTTTCTGGTAT
GTACCCCCATCGACCTCGACAC-GCCCCGACACGACG-
CCTCCCGCAACTCGACAACGCGGCGTTCTCAAC---
TACTTGGTTGGAACCAAACGAAAGACTTGATACTGACCAGTCTTTGATAGGCAAACCATTTCTG
GCGAGCACGGTCTCGACAGCAATGGAGTGTACGTACCCTTTCCTTGGCTCCTTGCTTTCCCATGA
GCATGTTGACTAACACTCG-
TGGTTGTTCAGCTACAACGGTACCTCTGAGCTCCAGCTCGAGCGCATGAGCGTCTACTTCAACG
AGGCTTCCGGCAACAAGTACGTTCCCCGTGCTGTCCTCGTCGATCTCGAGCCCGGTACCATGGA
TGCCGTCCGCGCCGGTCCTTTCGGCCAGCTCTTCCGCCCTGACAACTTCGTCTTCGGTCAGTCCG
GTGCT
>P_arceuthobii_CBS_434_65
AGGGATCATTATAGAGTTTTCTAAACTCCCAACCCATGTGAACTTACT-
ATTGTTGCCTCGGCAGAGGCTACCCGGT----
ACCTACCTTGGAACGGCCTACCCTGTAGCGCCTTACCCGGGAACGGATTACCCTGTAGCGGCTG
CCGGTGGACCACTAAACTCTTGTTATTTTATTGTAATCTGAGCGTCTTATTTTAATAAGTCAAAAC
TTTCAACAACGGATCTCTTGGTTCTGGCATCGATGAAGAACGCAGCGAAATGCGATAAGTAATG
TGAATTGCAGAATTCAGTGAATCATCGAATCTTTGAACGCACATTGCGCCCATTAGTATTCTAGT
GGGCATGCCTGTTCGAGCGTCATTTCAACCCTTAAGCCTAGCTTAGTGTTGGGAGCCTACTG--
CTTTTACTAGCTGTAGCTCCTGAAATACAACGGCGGATCTGCGATATCCTCTGAGCGTAGTAA-
TTTTTTTCTCGCTTTTGACTGGAGTTGCAGCGTCCTTTGCCGCTAAACCCCCA-AA-
TTTTTAATGGTTGACCTCAGGTTAGTCATCCTCCTGATCCC-CTCATCA-------------------
TCGTCATCACCACTTTCCCCTCC----AACATCTTCAACC--GGGTCGAAAATCT--
ATTTTTTCTATCCGCACCTGGG-CATATTCTG----

GCACTTACCCCGCCGCGTGGCCGCACGACCCCGCGGTGCCAACGAAAAATTTCTTA--
TCACAGCCCCACCTCGCACAAACATTTTGGCCGCTATGC-
CTTTTCAATACCCACTTTGAGCACTTGCTGACACCGCCAAACAGGAAGCCGCCGAGCTCGGTAA
GGGTTCCTTCAAGTACGCCTGGGTTCTTGACAAGCTCAAGGCCGAGCGTGAGCGTGGTATCACC
ATCGATATCGCTCTCTGGAAGTTCGAGACCAACGAGTACAATGTCACCGTCATTGGTTAGTATCC
ACGTCCACATCAAGCGTCATGCACCCGTTCAC-
AGACTAACATGGCAATACAGACGCTCCCGGTCACCGTGATTTCATCAATGCTGCCTTCTGGTAT
GTAGCCCCACCTACCTCGACAC-GGCTCAGCACGACG-
CCTCCCGCAACTCTACAACACGACCTTCTCAAC---
TATTTGGTTGGAACCCAGCGAAAGACTTGATACTGACCGGTCTTCGATAGGCAAACCATTTCTG
GCGAGCACGGTCTCGACAGCAATGGAGTGTACGTACCCCTTCCTTGGCTACTTGCTTTCCCACG
AACATGTTGGCTAACACACG-
AGCTTGTCCAGCTACAACGGTACCTCCGAGCTCCAGCTCGAGCGCATGAGCGTCTACTTCAACG
AGGCTTCCGGCAACAAGTACGTTCCTCGTGCCGTCCTCGTCGATCTCGAGCCCGGTACCATGGA
TGCCGTCCGCGCCGGTCCTTTCGGTCAGCTCTTCCGCCCTGACAACTTCGTCTTTGGTCAGTCCG
GTGCT
>P_arengae_CBS_331_92
AGGGATCATTATAGAGTTTTCTAAACTCCCAACCCATGTGAACTTACC-
ATTGTTGCCTCGGCAGAAGCTACCCTGTAGCGTCTTACCCGGGAACGGCCTACCCTGTAGCGCC
TTACCCTGGAACGACCTACCCTGTAGCGGCTGCCGGTGGACTACTCAACTCTTGTTATTTTATGG
TTATCTGAGCGTCTTATTTTAATAAGTCAAAACTTTCAACAACGGATCTCTTGGTTCTGGCATCGA
TGAAGAACGCAGCGAAATGCGATAAGTAATGTGAATTGCAGAATTCAGTGAATCATCGAATCTT
TGAACGCACATTGCGCCCATTAGTATTCTAGTGGGCATGCCTGTTCGAGCGTCATTTCAACCCTT
AAGCCTAGCTTAGTGTTGGGAGCCTACTG--
CTTTTACTAGCTGTAGCTCCTGAAATACAACGGCGGACCTGCGATATCCTCTGAGCGTAGTAA-
TTCTTTTCTCGCTTTTGACTGGAGTTGCAGCGTCCTTGGCCGCTAAACCCCCC-AA-
TTTTTAATGGTTGACCTCAGGTTAGTCAT---CACGATCCC-ATCTTCA--------------------
TCTTCATCAGCGCCT----CAAA----CAATTCCATCATCTGGTGCCGAAAATCA--------
TTTTTTCGCACCTTCC-CACGTTCTCTG--
ACACTTACCCCGCCGCGTGGCCGCACGACCCCGCGGTGCAAACGAAAAATTTCTTA--
TCACAGCCCCACCTCAC--AAACATTTTGGCAGCCACGCACTGT-
CATGACCCACCATGAACCTATACTGACCCCGCCAAATAGGAAGCCGCCGAGCTCGGTAAGGGT
TCCTTCAAGTACGCCTGGGTTCTTGACAAGCTCAAGGCCGAGCGTGAGCGTGGTATCACCATCG
ATATCGCTCTCTGGAAGTTCGAGACCAACGAGTACAATGTCACCGTCATTGGTTAGTATCCCTGT
CTACAACATGTATCATGCCTTCGACCTC-
AGACTAACATGGTAATACAGACGCTCCCGGTCACCGTGACTTCATCAATGCTGCTTTCTGGTATG
TACCC-TATATATCTCGACAC-GCCTCATTACGACG-
CCTCCCGCAACCCGACCACGCGACATTCTCAAC---
TACTCGGTTGGAATCACACGAAAGACTTGATACTGACCCGTCATTGATAGGCAAACCATCTCTG
GCGAGCACGGTCTCGACAGCAATGGAGTGTACGTACCCTTTCCTTGGCTGCTTGCT-----
ACGAACATGTTAGCTGACACTCG-
TGATTGTTCAGCTACAACGGTACCTCTGAGCTCCAGCTGGAGCGCATGAGCGTCTACTTCAACG
AGGCTTCCGGCAACAAGTACGTTCCTCGTGCCGTCCTCGTCGATCTCGAGCCCGGTACCATGGA
CGCCGTCCGTGCCGGTCCTTTCGGTCAGCTCTTCCGCCCTGACAACTTCGTCTTCGGTCAGTCCG

GTGCC
>P_australasiae_CBS_11141
AGGGATCATTATAGAGTTTTCTAAACTCCCAACCCATGTGAACTTACC-
ATTGTTGCCTCGGCAGAAGCTGCTCGGT-
GCACCCTACCTTGGAACGGCCTACCCTGTAGCGCCTTACCCTGGAACGGCTTACCCTGTAACGG
CTGCCGGTGGACTACCAAACTCTTGTTATTTTATTGTAATCTGAGCGTCTTATTTTAATAAGTCAA
AACTTTCAACAACGGATCTCTTGGTTCTGGCATCGATGAAGAACGCAGCGAAATGCGATAAGTA
ATGTGAATTGCAGAATTCAGTGAATCATCGAATCTTTGAACGCACATTGCGCCCATTAGTATTCT
AGTGGGCATGCCTGTTCGAGCGTCATTTCAACCCTTAAGCCTAGCTTAGTGTTGGGAGCCTACT
G--
CTTTTGCTAGCTGTAGCTCCTGAAATACAACGGCGGATCTGCGATATCCTCTGAGCGTAGTAAAT
TTTTATCTCGCTTTTGACTGGAGTTGCAGCGTCTTTGGCCGCTAAATCCCCC-AA-
TTTTTAATGGTTGACCTCAGGTTAGTCATCCTCGCAATCCCCATCATCC------TCATC--------
CTCATTATCACCACCT--CGCAAA----CA-TTTCCACACC-GGTGCCGAAAATCTGG--------
TTTTCGCACCTGCC-CATTTTCCCAG--ACACTTACCCC--------
GCCGCACGACCCCGCGGTGCAAACGAAAAATTTCTTA--
TCACAGCCCCACATCACACAAACATTTTGGCAGCCACGCACTTTGCATGACCCACAATGAACAA
TTGCTGACCCCGCCAAATAGGAAGCCGCCGAGCTCGGAAAGGGTTCCTTCAAGTACGCATGGG
TTCTTGACAAGCTCAAGGCCGAGCGTGAGCGTGGTATCACCATTGATATCGCTCTCTGGAAGTT
CGAGACCAACGAGTACAATGTCACCGTCATCGGTTAGTATCCCTGTCCACAACATGTGTCATGT
CTCTGAACTCAAGACTAACCTTGCAATACAGACGCTCCCGGTCACCGTGATTTCATCAATGCTG
CCTTCTGGTATGTAGCC-CATCTACCTCGACGC-GCCTCAATACGACA-
CCCCCGGCAACTCGGCAAC--GACATTCTCAAA---
TGCTTGTTTGGAACCATACGAAAGACTTGATACTGACCGGCCTATGATAGGCAAACCATCTCTG
GCGAGCACGGTCTCGACAGCAATGGAGTGTACGTACCCTTTCCCTGGCTACTCGCTTTCCCGTG
AACATGTCAGCTAACAGTCG-
TGCTTGTGCAGCTACAACGGTACCTCCGAGCTCCAGCTCGAGCGCATGAGCGTCTACTTCAACG
AGGCTTCCGGCAACAAGTACGTTCCTCGTGCCGTCCTCGTCGATCTCGAGCCCGGTACCATGGA
TGCCGTCCGCGCCGGTCCTTTCGGTCAGCTCTTCCGCCCTGACAACTTCGTCTTCGGTCAGTCCG
GTGCC
>P_australasiae_CBS_114126
AGGGATCATTATAGAGTTTTCTAAACTCCCAACCCATGTGAACTTACC-
ATTGTTGCCTCGGCAGAAGCTGCTCGGT-
GCACCCTACCTTGGAACGGCCTACCCTGTAGCGCCTTACCCTGGAACGGCTTACCCTGTAACGG
CTGCCGGTGGACTACCAAACTCTTGTTATTTTATTGTAATCTGAGCGTCTTATTTTAATAAGTCAA
AACTTTCAACAACGGATCTCTTGGTTCTGGCATCGATGAAGAACGCAGCGAAATGCGATAAGTA
ATGTGAATTGCAGAATTCAGTGAATCATCGAATCTTTGAACGCACATTGCGCCCATTAGTATTCT
AGTGGGCATGCCTGTTCGAGCGTCATTTCAACCCTTAAGCCTAGCTTAGTGTTGGGAGCCTACT
G--
CTTTTGCTAGCTGTAGCTCCTGAAATACAACGGCGGATCTGCGATATCCTCTGAGCGTAGTAAAT
TTTTATCTCGCTTTTGACTGGAGTTGCAGCGTCTTTGGCCGCTAAATCCCCC-AA-
TTTTTAATGGTTGACCTCAGGTTAGTCATCCTCGCAATCCCCATCATCC------TCATC--------
CTCATTATCACCACCT--CGCAAA----CA-TTTCCACACC-GGTGCCGAAAATCTGG--------
TTTTCGCACCTGCC-CATTTTCCCAG--ACACTTACCCC--------

GCCGCACGACCCCGCGGTGCAAACGAAAAATTTCTTA--
TCACAGCCCCACATCACACAAACATTTTGGCAGCCACGCACTTTGCATGACCCACAATGAACAA
TTGCTGACCCCGCCAAATAGGAAGCCGCCGAGCTCGGAAAGGGTTCCTTCAAGTACGCATGGG
TTCTTGACAAGCTCAAGGCCGAGCGTGAGCGTGGTATCACCATTGATATCGCTCTCTGGAAGTT
CGAGACCAACGAGTACAATGTCACCGTCATTGGTTAGCATCCCTGTCCACAACATGTGTCATGT
CTCTGAACTCAAGACTAACCTTGCAATACAGACGCTCCCGGTCACCGTGATTTCATCAATGCTG
CCTTCTGGTATGTAGCC-CATCTACCTCGACGC-GCCTCAATACGACA-
CCCCCGGCAACTCGGCAAC--GACATTCTCAAA---
TGCTTGTTTGGAACCATACGAAAGACTTGATACTGACCGGCCTATGATAGGCAAACCATCTCTG
GCGAGCACGGTCTCGACAGCAATGGAGTGTACGTACCCTTTCCCTGGCTACTCGCTTTCCCGTG
AACATGTCAGCTAACAGTCG-
TGCTTGTGCAGCTACAACGGTACCTCCGAGCTCCAGCTCGAGCGCATGAGCGTCTACTTCAACG
AGGCTTCCGGCAACAAGTACGTTCCTCGTGCCGTCCTCGTCGATCTCGAGCCCGGTACCATGGA
TGCCGTCCGCGCCGGTCCTTTCGGTCAGCTCTTCCGCCCTGACAACTTCGTCTTCGGTCAGTCCG
GTGCC
>P_australis_CBS_114193
AGGGATCATTATAGAGTTTTCTAAACTCCCAACCCATGTGAACTTACC-
ATTGTTGCCTCGGCAGAAGCTACCTGGT--
TACCCTACCTTGGAACGGCCTACCCTGTAGCGCCTTACCCTGGAACGGCCTACCCTGTAACGGC
TGCCGGTGGACTACCAAACTCTTGTTATTTTATTGTAATCTGAGCGTCTTATTTTAATAAGTCAAA
ACTTTCAACAACGGATCTCTTGGTTCTGGCATCGATGAAGAACGCAGCGAAATGCGATAAGTAA
TGTGAATTGCAGAATTCAGTGAATCATCGAATCTTTGAACGCACATTGCGCCCATTAGTATTCTA
GTGGGCATGCCTGTTCGAGCGTCATTTCAACCCTTAAGCCTAGCTTAGTGTTGGGAGCCTACTG-
-CTTTTGCTAGCGGTAGCTCCTGAAATACAACGGCGGATCTGCGATATCCTCTGAGCGTAGTAA-
ATTTTATCTCGCTTTTGACTGGAGTTGCAGCGTCTTTAGCCGCTAAACCCCCC-
AATTTTTTAATGGTTGACCTCAGGTTAG---TCCTCACAATCCC-ATCATTC------CCATC------
-----CTCATCATCGCCT--CGCACA----CA---TTCCAACC-GGTGCCGAAAATTT-G--------
TTTTCGCACCTGCC-CATTTTCCCAG--ACACTTACCCC--------
GCCGCACGACCCCGCGGTGCAAACGAAAAATTTCTTA--
TCACAGCCCCACATCACACAAACATTTTGGCAGCCATGCACTTTCCAGGACCCACAATGAACAA
TTGCTGACCCCGCCAAATAGGAAGCCGCCGAGCTCGGAAAGGGTTCCTTCAAGTACGCATGGG
TTCTTGACAAGCTCAAGGCCGAGCGTGAGCGTGGTATCACCATTGATATCGCTCTCTGGAAGTT
CGAGACCAACGAGTACAATGTCACCGTCATTGGTTAGTAACCCTGTCCATGCGATGTACCATGC
GTCTGAATGT-
ATGCTAACATGGCAACACAGATGCTCCCGGTCACCGTGATTTCATCAATGCTGCCTTTTGGTATG
TAGCC-CATCTACCTCGACAC-GCCTCAATACGACAACCCCCCGCAATTCGACAAC--
GACGTTCTCAACGACTGCTTGATTGGAAACAAGGGAAAGACTTGATACTGACCGGTCCCTGATA
GGCAAACCATCTCTGGCGAGCACGGTCTCGACAGCAATGGAGTGTACGTACCCTTTCCTTGGCT
ACTTGCTTTGCCACGAACATCTCAGCTAACACTCG-
TGGTTGTTCAGCTACAACGGTACCTCCGAGCTCCAGCTCGAGCGCATGAGCGTCTACTTCAACG
AGGCTTCCGGCAACAAGTACGTTCCTCGTGCCGTCCTCGTCGATCTCGAGCCCGGTACCATGGA
CGCCGTCCGCGCCGGTCCTTTCGGTCAGCTCTTCCGCCCTGACAACTTCGTCTTCGGTCAGTCCG
GTGCC
>P_biciliata_CAA1011

AGGGATCATTATAGAGTTTTCTAAACTCCCAACCCATGTGAACTTACC-
ATTGTTGCCTCGGCAGAAGCTGCTCGGT-
ACACCTTACCTTGGAACGGCCTACCCTGTAGCGCCTTACCCTGGAACGGCTTACCCTGTAACGG
CTGCCGGTGGACTACCAAACTCTTGTTATTTTATTGTAATCTGAGCGTCTTATTTTAATAAGTCAA
AACTTTCAACAACGGATCTCTTGGTTCTGGCATCGATGAAGAACGCAGCGAAATGCGATAAGTA
ATGTGAATTGCAGAATTCAGTGAATCATCGAATCTTTGAACGCACATTGCGCCCATTAGTATTCT
AGTGGGCATGCCTGTTCGAGCGTCATTTCAACCCTTAAGCCTAGCTTAGTGTTGGGAGCCTACT
G--
CTTTTACTAGCTGTAGCTCCTGAAATACAACGGCGGATCTGCGATATCCTCTGAGCGTAGTAA-
TTTTTATCTCGCTTTTGACTGGAGTTGCAGCGTCTTTAGCCGCTAAATCCCCC-AA-
TTTTTAATGGTTGACCTCAGGTTAGTCATCCTCGCAATCCC-ATCATCC------CCATC--------
CTCATCAACATCACCT--CGCAAA----CA-TTTCCACACC-GGTGCCGAAAATCTGG--------
TTTCCGCACCTTCC-CATTTTCCCAG--ACACTTACCCC--------
GCCGCACGACCCCGCGGTGCAAACGAAAAATTTCTTA--TCGCAGCCCCACATCAC--
AAACGTTTTGGCAGCCACGCACTTTGCATGACCCACAATGAACAATTGCTGACCCCGCCAAATA
GGAAGCCGCCGAGCTCGGAAAGGGTTCCTTCAAGTACGCATGGGTTCTTGACAAGCTCAAGGC
CGAGCGTGAGCGTGGTATCACCATTGATATCGCTCTCTGGAAGTTCGAGACCAACGAGTACAAT
GTCACCGTCATTGGTTAGTATCCCTGTCCACAACATGTGTCATGTCACCGAACTCAAGACTAACC
TTGCAATACAGACGCTCCCGGTCACCGTGATTTCATCAA??????????TGGTATGTAGCC-
CATCTACCTCGACGC-GCCTCGATACGACA-CCCCCGGCAACTCGACAAC--GACATTCTCAAC-
--
TGCTTGTTTGGAACCATACGAAAGACTTGATACTGACCGGTCTATGATAGGCAAACCATCTCTG
GCGAGCACGGTCTCGACAGCAATGGAGTGTACGTACCCTTTCCCTGGCTACTCGCTTTCTCGTG
AACATGTCAGCTAACAGTCG-
TGCTTGTTCAGCTACAACGGTACCTCCGAGCTCCAGCTCGAGCGCATGAGCGTCTACTTCAACG
AGGCTTCCGGCAACAAGTACGTTCCTCGTGCCGTCCTCGTCGATCTCGAGCCCGGTACCATGGA
TGCCGTCCGCGCCGGTCCTTTCGGTCAGCTCTTCCGCCCTGACAACTTCGTTTTCGGTCAGTCCG
GTGCC
>P_biciliata_CBS_124463
AGGGATCATTATAGAGTTTTCTAAACTCCCAACCCATGTGAACTTACC-
ATTGTTGCCTCGGCAGAAGCTGCTCGGT-
ACACCTTACCTTGGAACGGCCTACCCTGTAGCGCCTTACCCTGGAACGGCTTACCCTGTAACGG
CTGCCGGTGGACTACCAAACTCTTGTTATTTTATTGTAATCTGAGCGTCTTATTTTAATAAGTCAA
AACTTTCAACAACGGATCTCTTGGTTCTGGCATCGATGAAGAACGCAGCGAAATGCGATAAGTA
ATGTGAATTGCAGAATTCAGTGAATCATCGAATCTTTGAACGCACATTGCGCCCATTAGTATTCT
AGTGGGCATGCCTGTTCGAGCGTCATTTCAACCCTTAAGCCTAGCTTAGTGTTGGGAGCCTACT
G--
CTTTTACTAGCTGTAGCTCCTGAAATACAACGGCGGATCTGCGATATCCTCTGAGCGTAGTAA-
TTTTTATCTCGCTTTTGACTGGAGTTGCAGCGTCTTTAGCCGCTAAATCCCCC-AA-
TTTTTAATGGTTGACCTCAGGTTAGTCATCCTCGCAATCCC-ATCATCC------CCATC--------
CTCATCAACATCACCT--CGCAAA----CA-TTTCCACACC-GGTGCCGAAAATCTGG--------
TTTCCGCACCTGCC-CATTTTCCCAG--ACACTTACCCC--------
GCCGCACGACCCCGCGGTGCAAACGAAAAATTTCTTA--TCGCAGCCCCACATCACA-
AAACGTTTTGGCAGCCACGCACTTTGCATGACCCACAATGAACAATTGCTGACCCCGCCAAATA

GGAAGCCGCCGAGCTCGGAAAGGGTTCCTTCAAGTACGCATGGGTTCTTGACAAGCTCAAGGC
CGAGCGTGAGCGTGGTATCACCATTGATATCGCTCTCTGGAAGTTCGAGACCAACGAGTACAAT
GTCACCGTCATTGGTTAGTATCCCTGTCCACAACATGTGTCATGTCACCGAACTCAAGACTAACC
TTGCAATACAGACGCTCCCGGTCACCGTGATTTCATCAATGCTGCCTTCTGGTATGTAGCC-
CATCTACCTCGACGC-GCCTCGATACGACA-CCCCCGGCAACTCGACAAC--GACATTCTCAAC-
--
TGCTTGTTTGGAACCATACGAAAGACTTGATACTGACCGGTCTATGATAGGCAAACCATCTCTG
GCGAGCACGGTCTCGACAGCAATGGAGTGTACGTACCCTTTCCCTGGCTACTCGCTTTCTCGTG
AACATGTCAGCTAACAGTCG-
TGCTTGTTCAGCTACAACGGTACCTCCGAGCTCCAGCTCGAGCGCATGAGCGTCTACTTCAACG
AGGCTTCCGGCAACAAGTACGTTCCTCGTGCCGTCCTCGTCGATCTCGAGCCCGGTACCATGGA
TGCCGTCCGCGCCGGTCCTTTCGGTCAGCTCTTCCGCCCTGACAACTTCGTTTTCGGTCAGTCCG
GTGCC
>P_brachiata_LC2988
??????????????????????????????ACCCATGTGAACTTACC-
ATTGTTGCCTCGGCAGAAGCTGCTCGGT-
GCACCCTACCTTGGAACGGCCTACCCTGTAGCGCCTTACCCTGGAACGGCCTACCCTGTAGCGG
CTGCCGGTGGACTACCAAACTCTTGTTATTTTATTGTAATCTGAGCGTCTTATTTTAATAAGTCAA
AACTTTCAACAACGGATCTCTTGGTTCTGGCATCGATGAAGAACGCAGCGAAATGCGATAAGTA
ATGTGAATTGCAGAATTCAGTGAATCATCGAATCTTTGAACGCACATTGCGCCCATTAGTATTCT
AGTGGGCATGCCTGTTCGAGCGTCATTTCAACCCTTAAGCCTAGCTTAGTGTTGGGAGCCTACT
G--
CTTTTGCTAGCTGTAGCTCCTGAAATACAACGGCGGATCTGCGATATCCTCTGAGCGTAGTAA-
TTTTTATCTCGCTTTTGACTGGAGTTGCAGCGTCTTTAGCCGCTAAATCCCCC-AA-
TTTTTAATGGTTGACCTCAGGTTAGTCATCCTCGCAATCCC-ATCATCC------TCATC-----
CTCATCATCATCACCACCT--CGCAAA----CG-TTGCCACACC-GGTGGCGAAAATCTGG-----
---TTTTCGCACCTGCC-CATTTTCCCAG--ACACTTACCCC--------
GCCGCACGACCCCGCGGTGCAAACGAAAAATTTCTTA--
TCATAGCCCCACATCACACAAACATTTTGGCAGCCACGCACTTTGCATGACCCACAATGAACAA
TTGCTGACCCCGCCAAATAGGAAGCCGCCGAGCTCGGAAAGGGTTCCTTCAAGTACGCATGGG
TTCTTGACAAGCTCAAGGCCGAGCGTGAGCGTGGTATCACCATTGATATCGCTCTCTGGAAGTT
CGAGACCAACGAGTACAATGTCACCGTCATTGGTTAGTATCCCTGTCCACAACATGTGTCATGT
CTCCAAACTCAAGACTAACCTTGCAATACAGACGCTCCCGGTCACCGTGATTTCATCAATGCTG
CCTTCTGGTATGTAGCC-CATCTACCTCGACGC-GCCTCGATACGACA-
CCCCCGGCAACTCGACAAC--GACATTCTCAAC---
TGCTTGTTTGGAACCATACGAAAGACTTGATACTGACCGGTCTATGATAGGCAAACCATCTCTG
GCGAGCACGGTCTCGACAGCAATGGAGTGTACGTACCCTTTCCCTGGCTACTCGCTTTCTCGTG
AACATGTCAGCTAACAGTCG-
TGCTTGTTCAGCTACAACGGTACCTCCGAGCTCCAGCTCGAGCGCATGAGCGTCTACTTCAACG
AGGCTTCCGGCAACAAGTACGTTCCTCGTGCCGTCCTCGTCGATCTCGAGCCCGGTACCATGGA
TGCCGTCCGCGCCGGTCCTTTCGGTCAGCTCTTCCGCCCTGACAACTTCGTTTTCGGTCAGTCCG
?????
>P_brachiata_LC8189
AGGGATCATTATAGAGTTTTCTAAACTCCCAACCCATGTGAACTTACC-

ATTGTTGCCTCGGCAGAAGCTGCTCGGT-
GCACCCTACCTTGGAACGGCCTACCCTGTAGCGCCTTACCCTGGAACGGCCTACCCTGTAGCGG
CTGCCGGTGGACTACCAAACTCTTGTTATTTTATTGTAATCTGAGCGTCTTATTTTAATAAGTCAA
AACTTTCAACAACGGATCTCTTGGTTCTGGCATCGATGAAGAACGCAGCGAAATGCGATAAGTA
ATGTGAATTGCAGAATTCAGTGAATCATCGAATCTTTGAACGCACATTGCGCCCATTAGTATTCT
AGTGGGCATGCCTGTTCGAGCGTCATTTCAACCCTTAAGCCTAGCTTAGTGTTGGGAGCCTACT
G--
CTTTTGCTAGCTGTAGCTCCTGAAATACAACGGCGGATCTGCGATATCCTCTGAGCGTAGTAA-
TTTTTATCTCGCTTTTGACTGGAGTTGCAGCGTCTTTAGCCGCTAAATCCCCC-AA-
TTTTTAATGGTTGACCTCAGGTTAGTCATCCTCGCAATCCC-ATCATCCTCATCCTCATC--------
---ATCATCACCACCT--CGCAAA----CG-TTGCCACACC-GGTGGCGAAAATCTGG--------
TTTTCGCACCTGCC-CATTTTCCCAG--ACACTTACCCC--------
GCCGCACGACCCCGCGGTGCAAACGAAAAATTTCTTA--
TCATAGCCCCACATCACACAAACATTTTGGCAGCCACGCACTTTGCATGACCCACAATGAACAA
TTGCTGACCCCGCCAAATAGGAAGCCGCCGAGCTCGGAAAGGGTTCCTTCAAGTACGCATGGG
TTCTTGACAAGCTCAAGGCCGAGCGTGAGCGTGGTATCACCATTGATATCGCTCTCTGGAAGTT
CGAGACCAACGAGTACAATGTCACCGTCATTGGTTAGTATCCCTGTCCACAACATGTGTCATGT
CTCCAAACTCAAGACTAACCTTGCAATACAGACGCTCCCGGTCACCGTGATTTCATCAATGCTG
CCTTCTGGTATGTAGCC-CATCTACCTCGACGC-GCCTCGATACGACA-
CCCCCGGCAACTCGACAAC--GACATTCTCAAC---
TGCTTGTTTGGAACCATACGAAAGACTTGATACTGACCGGTCTATGATAGGCAAACCATCTCTG
GCGAGCACGGTCTCGACAGCAATGGAGTGTACGTACCCTTTCCCTGGCTACTCGCTTTCTCGTG
AACATGTCAGCTAACAGTCG-
TGCTTGTTCAGCTACAACGGTACCTCCGAGCTCCAGCTCGAGCGCATGAGCGTCTACTTCAACG
AGGCTTCCGGCAACAAGTACGTTCCTCGTGCCGTCCTCGTCGATCTCGAGCCCGGTACCATGGA
TGCCGTCCGCGCCGGTCCTTTCGGTCAGCTCTTCCGCCCTGACAACTTCGTTTTCGGTCAGTCCG
GTGCC
>P_brassicae_CBS_170_26
??????CATTATAGAGTTTTCTAAACTCCCAACCCATGTGAACTTACC-
ATTGTTGCCTCGGCAGAAGCTACCTGGT--
TACCCTACCTTGGAACGGCCTACCCTGTAGCGCCTTACCCTGGAACGGCCTACCCTGTAACGGC
TGCCGGTGGACTACCAAACTCTTGTTATTTTATTGTAATCTGAGCGTCTTATTTTAATAAGTCAAA
ACTTTCAACAACGGATCTCTTGGTTCTGGCATCGATGAAGAACGCAGCGAAATGCGATAAGTAA
TGTGAATTGCAGAATTCAGTGAATCATCGAATCTTTGAACGCACATTGCGCCCATTAGTATTCTA
GTGGGCATGCCTGTTCGAGCGTCATTTCAACCCTTAAGCCTAGCTTAGTGTTGGGAGCCTACCG-
-CTTTTGCTAGCGGTAGCTCCTGAAATACAACGGCGGATCTGCGATATCCTCTGAGCGTAGTAA-
TTTTTATCTCGCTTTTGACTGGAGTTGCAGCGTCTTTAGCCGCTAAACCCCCCAAATTTTTTAATG
GTTGACCTC?????????ATCCACACAATCCC-ATCATTC------
CCACCATTCCCATCTTATCATCATCGCCT----CAAA----CA-TCTTCCAACC-
GGTGCCGAAAATCT-G--------TTTTCGCACCTGCC-CATTTTCCCAG--ACACTTACCCC-----
---GCCGCACGACCCCGCGGTGCAAACGAAAAATTTCTTA--
TCACAGCCCCACATCGCACAAACATTTTGGCAGCCATGCACTTTCCAAGACCCACAATGAACAA
TTGCTGACCCCGCCAAATAGGAAGCCGCCGAGCTCGGAAAGGGTTCCTTCAAGTACGCATGGG
TTCTTGACAAGCTCAAGGCCGAGCGTGAGCGTGGTATCACCATTGATATCGCTCTCTGGAAGTT

CGAGACCAACGAGTACAATGTCACCGTCATTGGTTAGTATCCCTGCCCACACCATGTATCATGC
ATCCGACCTT-
GTGCTAACATGGCAACACAGATGCTCCCGGTCACCGTGACTTCATCAA??????????????????????
??
??
??
??
??
>P_camelliae_MFLUCC_12_0277
??????CATTATAGAGTTTTTTAAACTCCCAACCCATGTGAACTTACC-
ATTGTTGCCTCGGCAGAAGCTACCCGGT---
ACCTTACCTTGGAACGGCCTACCCTGTAGCGCCTTACCCTGGAACGGCTTACCCTGCAGCGGCT
GCCGGTGGACTACCAAACTCTTGTTATTTTTATGTTATCTGAGCGTCTTATTTTAATAAGTCAAAA
CTTTCAACAACGGATCTCTTGGTTCTGGCATCGATGAAGAACGCAGCGAAATGCGATAAGTAAT
GTGAATTGCAGAATTCAGTGAATCATCGAATCTTTGAACGCACATTGCGCCCATTAGTATTCTAG
TGGGCATGCCTGTTCGAGCGTCATTTCAACCCTTAAGCCTAGCTTAGTGTTGGGAGCCTACTG--
CTTTTGTTAGCTGTAGCTCCTGAAATACAACGGCGGATCTGCGATATCCTCTGAGCGTAGTAATT
TTTTTCCTCGCTTTTGACTGGAGTTGCAGCGTCTTTAGCCGCTAAACCCCCC-AA--
TTTTAATGGTTGACCTCAGGATAGTTATCCTCACAATTCC-ATCATCC---------------TCACT-
----ATCATTACCT--CGCAAA----TA-CTTCCAAACC-GGTGTCGAAAATAT-
GTGCTTCTTTTTTCGCGCCTGCC-CACATCCCCAG--ACACTTACCCC--------
GCCGCACGACCCCGCGGTGCAAACGAAAAATTTCTTA--
TCACAGCCCCACACCGCACAAACATTTTGGCAGCCATGCACTTTTCATAACCCAC-
ACGAGCATTTGCTGACCCCGCCAAATAGGAAGCCGCCGAGCTCGGAAAGGGTTCCTTCAAGTA
CGCATGGGTTCTTGACAAGCTCAAGGCCGAGCGTGAGCGTGGTATCACCATCGATATCGCTCTC
TGGAAGTTCGAGACCAACGAGTACAATGTCACCGTCATTGGTTAGTATCCCTGTCC---
ACATCCATCATGCATCCGAAATC-
AGACTAACACGGCACCACAGATGCTCCCGGTCACCGTGATTTCATCAATGCTGCTTTCTGGTAT
GTAGCT-CATCTACCTCGACGC-GCCTCAGCACGACG-CCTCTCGCAACTCGACAAC--
GACGTTATCAAC---
TACTTGGTTGAAACCAAGCGAAAGACTTGATACTGACAGGCCTCTGATAGGCAAACCATCTCTG
GCGAGCACGGTCTCGACAGCAATGGAGTGTACGTACCGTACCCTTGGCTACCTGCT-
TCCCACGAACATGTCAGCTAACACTCG----
TGGTTCAGCTACAACGGTACCTCCGAGCTCCAGCTCGAGCGCATGAGCGTCTACTTCAACGAGG
CTTCCGGCAACAAGTACGTTCCTCGTGCCGTCCTCGTCGATCTCGAGCCCGGTACCATGGATGC
CGTCCGCGCCGGTCCTTTCGGTCAGCTCTTCCGCCCTGACAACTTCGTCTTCGGTCAGTCCGGTG
CC
>P_camelliae_oleiferae_CSUFTCC08
???????????????????????????????????TGTGAACTTACC-
ATTGTTGCCTCGGCAGAAGCTGCTCGGT-
GCACCCTACCTTGGAACGGCCTACCCTGTAGCGCCTTACCCTGGAACGGCTTACCCTGTAGCGG
CTGCCGGTGGACTACTAAACTCTTGTTATTTTATTGTAATCTGAGCGTCTTATTTTAATAAGTCAA
AACTTTCAACAACGGATCTCTTGGTTCTGGCATCGATGAAGAACGCAGCGAAATGCGATAAGTA
ATGTGAATTGCAGAATTCAGTGAATCATCGAATCTTTGAACGCACATTGCGCCCATTAGTATTCT

AGTGGGCATGCCTGTTCGAGCGTCATTTCAACCCTTAAGCCTAGCTTAGTGTTGGGAGCCTACT
G--
CTTTTACTAGCTGTAGCTCCTGAAATACAACGGCGGATCTGCGATATCCTCTGAGCGTAGTAA-
TTTTTATCTCGCTTTTGACTGGAGTTGCAGCGTCTTTAGCCGCTAAATCCCCC-AA-
TTTTTAATGGTTGACCTCAGGTTAGTCATCCTCGCAATCCC-ATCATCC------CCATC--------
CTCATCAACATCACCT--CGCAAA----CA-TTTCCACACC-GGTGCCGAAAATCTGG--------
TTTCCGCACCTGCC-CATTTTCCCAG--ACACTTACCCC--------
GCCGCACGACCCCGCGGTGCAAACGAAAAATTTCTTA--TCGCAGCCCCACATCAC--
AAACGTTTTGGCAGCCACGCACTTTGCATGACCCACAATGAACAATTGCTGACCCCGCCAAATA
GGAAGCCGCCGAGCTCGGAAAGGGTTCCTTCAAGTACGCATGGGTTCTTGACAAGCTCAAGGC
CGAGCGTGAGCGTGGTATCACCATTGATATCGCTCTCTGGAAGTTCGAGACCAACGAGTACAAT
GTCACCGTCATTGGTTAGTATCCCTGTCCACAACATGTGTCATGTCACCGAACTCAAGACTAACC
TTGCAATACAGACGCTCCCGGTCACCGTGATTTCATCAATGCTGCCTTCTGGTATGTAGCC-
CATCTACCTCGACGC-GCCTCAATACGACA-CCCCCGGCAACTCGACAAC--GACATTCTCAAC-
--
TGCTTGTTTGGAACCATACGAAAGACTTGATACTGACCGGTCTATGATAGGCAAACCATCTCTG
GCGAGCACGGTCTCGACAGCAATGGAGTGTACGTACCCTTTCCCTGGCTACTCGCTTTCTCGTG
AACATGTCAGCTAACAGTTG-
TGCTTGTTCAGCTACAACGGTACCTCCGAGCTCCAGCTCGAGCGCATGAGCGTCTACTTCAACG
AGGCTTCCGGCAACAAGTACGTTCCTCGTGCCGTCCTCGTCGATCTCGAGCCCGGTACCATGGA
TGCCGTCCGCGCCGGTCCTTTCGGTCAGCTCTTCCGCCCTGACAACTTCGTCTTCGGTCAGTCCG
GTGCC
>P_camelliae_oleiferae_CSUFTCC09
??TGTGAACTTACC-
ATTGTTGCCTCGGCAGAAGCTGCTCGGT-
GCACCCTACCTTGGAACGGCCTACCCTGTAGCGCCTTACCCTGGAACGGCTTACCCTGTAGCGG
CTGCCGGTGGACTACTAAACTCTTGTTATTTTATTGTAATCTGAGCGTCTTATTTTAATAAGTCAA
AACTTTCAACAACGGATCTCTTGGTTCTGGCATCGATGAAGAACGCAGCGAAATGCGATAAGTA
ATGTGAATTGCAGAATTCAGTGAATCATCGAATCTTTGAACGCACATTGCGCCCATTAGTATTCT
AGTGGGCATGCCTGTTCGAGCGTCATTTCAACCCTTAAGCCTAGCTTAGTGTTGGGAGCCTACT
G--
CTTTTACTAGCTGTAGCTCCTGAAATACAACGGCGGATCTGCGATATCCTCTGAGCGTAGTAA-
TTTTTATCTCGCTTTTGACTGGAGTTGCAGCGTCTTTAGCCGCTAAATCCCCC-AA-
TTTTTAATGGTTGACCTCAGGTTAGTCATCCTCGCAATCCC-ATCATCC------CCATC--------
CTCATCAACATCACCT--CGCAAA----CA-TTTCCACACC-GGTGCCGAAAATCTGG--------
TTTCCGCACCTGCC-CATTTTCCCAG--ACACTTACCCC--------
GCCGCACGACCCCGCGGTGCAAACGAAAAATTTCTTA--TCGCAGCCCCACATCAC--
AAACGTTTTGGCAGCCACGCACTTTGCATGACCCACAATGAACAATTGCTGACCCCGCCAAATA
GGAAGCCGCCGAGCTCGGAAAGGGTTCCTTCAAGTACGCATGGGTTCTTGACAAGCTCAAGGC
CGAGCGTGAGCGTGGTATCACCATTGATATCGCTCTCTGGAAGTTCGAGACCAACGAGTACAAT
GTCACCGTCATTGGTTAGTATCCCTGTCCACAACATGTGTCATGTCACCGAACTCAAGACTAACC
TTGCAATACAGACGCTCCCGGTCACCGTGATTTCATCAATGCTGCCTTCTGGTATGTAGCC-
CATCTACCTCGACGC-GCCTCAATACGACA-CCCCCGGCAACTCGACAAC--GACATTCTCAAC-
--

TGCTTGTTTGGAACCATACGAAAGACTTGATACTGACCGGTCTATGATAGGCAAACCATCTCTG
GCGAGCACGGTCTCGACAGCAATGGAGTGTACGTACCCTTTCCCTGGCTACTCGCTTTCTCGTG
AACATGTCAGCTAACAGTTG-
TGCTTGTTCAGCTACAACGGTACCTCCGAGCTCCAGCTCGAGCGCATGAGCGTCTACTTCAACG
AGGCTTCCGGCAACAAGTACGTTCCTCGTGCCGTCCTCGTCGATCTCGAGCCCGGTACCATGGA
TGCCGTCCGCGCCGGTCCTTTCGGTCAGCTCTTCCGCCCTGACAACTTCGTCTTCGGTCAGTCCG
GTGCC
>P_chamaeropis_CBS_186_71
AGGGATCATTATAGAGTTTTTTAAACTCCCAACCCATGTGAACTTACC-
ATTGTTGCCTCGGCAGAAGCTACCTGGT--
TACCTTACCTTGGAACGGCCTACCCTGTAGCGCCTTACCCTGGAACGGCCTACCCTGTAACGGC
TGCCGGTGGACTACCAAACTCTTGTTATTTTATTGTAATCTGAGCGTCTTATTTTAATAAGTCAAA
ACTTTCAACAACGGATCTCTTGGTTCTGGCATCGATGAAGAACGCAGCGAAATGCGATAAGTAA
TGTGAATTGCAGAATTCAGTGAATCATCGAATCTTTGAACGCACATTGCGCCCATTAGTATTCTA
GTGGGCATGCCTGTTCGAGCGTCATTTCAACCCTTAAGCCTAGCTTAGTGTTGGGAGCCTACTG-
-CTTTTGCTAGCGGTAGCTCCTGAAATACAACGGCGGATCTGCGATATCCTCTGAGCGTAGTAA-
TTTTTATCTCGCTTTTGACTGGAGTTGCAGCGTCTTTAGCCGCTAAACCCCCC-AA-
TTTTTAATGGTTGACCTCAGGTTAGTCATCCTCAAA-TCCC-ATCATTC------CCATC--------
CTCATCATCATCGCCT--CGCAAA----CA-TTTTCCAACC-GGTGCCGAGAATCT-G--------
TTTTCGCATCTGCC-CATTTTCCCAG--ACACTTACCCC--------
GCCGCACGACCCCGCGGTGCAAACGAAAAAATTCTTA--
TCACAGCCCCACATCGCACAAACATTTTGGCAGCCATGCACTTTCCAAGACCCACAATGAACAT
TTGCTGACCCCGCCAAATAGGAAGCCGCCGAGCTCGGAAAGGGTTCCTTCAAGTACGCATGGG
TTCTTGACAAGCTCAAGGCCGAGCGTGAGCGTGGTATCACCATCGATATCGCTCTCTGGAAGTT
CGAGACCAACGAGTACAATGTCACCGTCATTGGTTAGTATCCCTGTCCACACGATGTACTATGC
ATCTGAATGT-
ATACTAACATGGCAACACAGATGCTCCCGGTCACCGTGATTTCATCAATGCTGCCTTTTGGTATG
TAGCC-CATCTACCTCGACAC-GCCTCAATACGACAACCCCCCGCAACTCGACAAC--
GACGTTCTCAACAAGTGCTTGCTTGGAAACAAGGGAAAGACTTGATACTGACCGGTCCCTGATA
GGCAAACCATCTCTGGCGAGCACGGTCTCGACAGCAATGGAGTGTACGTACCCTTTCCTTGGCT
ACTTGCTTTCCCACGAACATCTCAGCTAACACTCG-
TGGTTGTTCAGCTACAACGGTACCTCCGAGCTCCAGCTCGAGCGCATGAGCGTCTACTTCAACG
AGGCTTCCGGCAACAAGTACGTTCCTCGTGCCGTCCTCGTCGATCTCGAGCCCGGTACCATGGA
TGCCGTCCGCGCCGGTCCTTTCGGTCAGCTCTTCCGCCCTGACAACTTCGTCTTCGGTCAGTCCG
GTGCC
>P_chinensis_MFLUCC_12_0273
??????CATTATAGAGTTTTCTAAACTCCCAACCCATGTGAACTTACC-
ATTGTTGCCTCGGCAGAAGCTACCTGGT--
TACCCTACCTTGGAACGGCCTACCCTGTAGCGCCTTACCCTGGAACGGCCTACCCTGTAACGGC
TGCCGGTGGACTACCAAACTCTTGTTATTTTATTGTAATCTGAGCGTCTTATTTTAATAAGTCAAA
ACTTTCAACAACGGATCTCTTGGTTCTGGCATCGATGAAGAACGCAGCGAAATGCGATAAGTAA
TGTGAATTGCAGAATTCAGTGAATCATCGAATCTTTGAACGCACATTGCGCCCATTAGTATTCTA
GTGGGCATGCCTGTTCGAGCGTCATTTCAACCCTTAAGCCTAGCTTAGTGTTGGGAGCCTACCG-
-CTTTTGCTAGCGGTAGCTCCTGAAATACAACGGCGGATCTGCGATATCCTCTGAGCGTAGTAA-

TTTTTATCTCGCTTTTGACTGGAGTTGCAGCGTCTTTAGCCGCTAAACCCCCCAAATTTTTTAATG
GTTGACCTC??
??
??
??
??
??
??
??
??
??
???
>P_clavata_MFLUCC_12_0268
??????CATTATAGAGTTTTCTAAACTCCCAACCCATGTGAACTTACC-
ATTGTTGCCTCGGCAGAAGCTGCTCGGT-
GCACCTTACCCTGGAACGGCCTACCCTGTAGCGCCTTACCCTGGAACGGCTTACCCTGTAGCGG
CTGCCGGTGGACTACCAAACTCTTGTTATTTTATTGTAATCTGAGCGTCTTATTTTAATAAGTCAA
AACTTTCAACAACGGATCTCTTGGTTCTGGCATCGATGAAGAACGCAGCGAAATGCGATAAGTA
ATGTGAATTGCAGAATTCAGTGAATCATCGAATCTTTGAACGCACATTGCGCCCATTAGTATTCT
AGTGGGCATGCCTGTTCGAGCGTCATTTCAACCCTTAAGCCTAGCTTAGTGTTGGGAGCCTACT
G--
CTTTTGCTAGCTGTAGCTCCTGAAATACAACGGCGGATCTGCGATATCCTCTGAGCGTAGTAA-
TTTTTATCTCGCTTTTGACTGGAGTTGCAGCGTCTTTAGCCGCTAAATCCCCC-AA-
TTTTTAATGGTTGACCTCAGGTTAGTCATTCTCACAATCCC-ATCATTC------CCATC--------
CTCATCATCATCACCT--CGCAAA----CA-TTTTCCAACT-GGTGCCGAAAATCT-G--------
TTTTCGCACCTGCC-CATTTTCCCAG--ACACTTACCCC--------
GCCGCACGACCCCGCGGTGCAAACGAAAAATTTCTTA--
TCACAGCCCCACATCGCACAAACATTTTGGCAGTCATGCACTTTCCAAGACCCACAATGAACAA
TTGCTGACCCCGCCAAATAGGAAGCCGCCGAGCTCGGAAAGGGTTCCTTCAAGTACGCATGGG
TTCTTGACAAGCTCAAGGCCGAGCGTGAGCGTGGTATCACCATTGATATCGCTCTCTGGAAGTT
CGAGACCAACGAGTACAATGTCACCGTCATTGGTTAGTATCCCTGTCCACACGATGTACCATGC
ATCCGACCTT-
GTACTAACATGGCAATCCAGATGCTCCCGGTCACCGTGATTTCATCAATGCTGCTTTCTGGTACG
TAGTC-CATCTACCTCGACAC-GCCTCAATACGACAACCCTCCGCAACTCGACAAC--
GACCTTCTCAACAACTGCTTGGTTGGAACCAAAGAAAAGACCTGATACTGACCGGTCTCTGATA
GGCAAACCATCTCTGGCGAGCACGGTCTCGACAGCAATGGAGTGTACGTACCCTTTCCTTGGCT
ACTTGCTTTCCCACGAACATCTCAGCTAATACCCG-
TGATTGTTCAGCTACAACGGTACCTCTGAGCTCCAGCTCGAGCGCATGAGCGTCTACTTCAACG
AGGCTTCCGGCAACAAGTACGTTCCTCGTGCCGTCCTCGTCGATCTCGAGCCCGGTACCATGGA
TGCCGTCCGCGCCGGTCCTTTCGGTCAGCTCTTCCGCCCTGACAACTTTGTCTTCGGTCAGTCCG
GTGCC
>P_colombiensis_CBS_118553
AGGGATCATTATAGAGTTTTCTAAACTCCCAACCCATGTGAACTTACC-
ATTGTTGCCTCGGCAGAAGCTGCTCGGT-

ATACCCTACCTTGGAACGGCCTACCCTGTAGCGCCTTACCCTGGAACGGCTTACCCTGCAACGG
CTGCCGGTGGACTACTAAACTCTTGTTATTTTATTGTAATCTGAGCGTCTTATTTTAATAAGTCAA
AACTTTCAACAACGGATCTCTTGGTTCTGGCATCGATGAAGAACGCAGCGAAATGCGATAAGTA
ATGTGAATTGCAGAATTCAGTGAATCATCGAATCTTTGAACGCACATTGCGCCCATTAGTATTCT
AGTGGGCATGCCTGTTCGAGCGTCATTTCAACCCTTAAGCCTAGCTTAGTGTTGGGAGCCTACT
G--
CTTTTACTAGCTGTAGCTCCTGAAATACAACGGCGGATCTGCGATATCCTCTGAGCGTAGTAA-
TTTTTATCTCGCTTTTGACTGGAGTTGCAGCGTCTTTAGCCGCTAAACCCCCC-AA-
TTTTTAATGGTTGACCTCAGGTTAGTCATGCTCACAGTCCC-ATCATCC------TCATC---------
--ATAATCATCGACT--CGCAAA----CA-TTTTCCAACC-GGTGCCGAAATTCT-G--------
TTTTCGCACCTGCC-CATTTTCCCAG--ACACTTACCCC--------
GCCGCACGACCCCGCGGTGCAAACGAAAAATTTCTTA--TCACAGCCCCACTTCACAC-
AACATTTTGGCAGCCACGCACTTTGCATGACCCACAGTGAACAATTGCTAACCCCGCCAAATAG
GAAGCCGCCGAGCTCGGAAAGGGTTCCTTCAAGTACGCATGGGTTCTTGACAAGCTCAAGGCC
GAGCGTGAGCGTGGTATCACCATTGATATCGCTCTCTGGAAGTTCGAGACCAACGAGTACAATG
TCACCGTCATTGGTTAGTATCCCTGTTCACAGAATGTACCATGTCTCCGAACTC-
AGACTAACATCACAACACAGACGCTCCCGGTCACCGTGATTTCATCAATGCTGCCTTCTGGTAT
GTAGCC-CATCTACTTCGGCAC-GCCTCAATACGACA-CCCTCCGCAACTCGACGAC--
GACATTCTCGGC---
TACTTGGTTGGAACCGAACGAAAGACTTGATACTGACCGGTCTCTGATAGGCAAACCATCTCTG
GCGAGCACGGTCTCGACAGCAATGGAGTGTACGTACCATTTCCTTGCCTACTTGCTTTCCCACG
AACATGTTAGCTAACACTCG-
TGCTTGCTCAGCTACAACGGCACCTCCGAGCTCCAGCTCGAGCGCATGAGCGTCTACTTCAACG
AGGCGTCCGGCAACAAGTACGTTCCTCGTGCCGTCCTCGTCGATCTCGAGCCCGGTACCATGGA
CGCCGTCCGCGCCGGTCCTTTCGGCCAGCTCTTCCGCCCTGACAACTTCGTCTTCGGTCAGTCC
GGTGCC
>P_digitalis_ICMP_5434
??????CATTATAGAGTTTTCTAAACTCCCAACCCATGTGAACTTACC-
ATTGTTGCCTCGGCAGAAGCTGCTCGGT-
GCACCCTACCTTGGAACGGCCTACCCTGTAGCGCCTTACCCTGGAACGGCTTACCCTGTAGCGG
CTGCCGGTGGACTACCAAACTCTTGTTATTTTATTGTAATCTGAGCGTCTTATTTTAATAAGTCAA
AACTTTCAACAACGGATCTCTTGGTTCTGGCATCGATGAAGAACGCAGCGAAATGCGATAAGTA
ATGTGAATTGCAGAATTCAGTGAATCATCGAATCTTTGAACGCACATTGCGCCCATTAGTATTCT
AGTGGGCATGCCTGTTCGAGCGTCATTTCAACCCTTAAGCCTAGCTTAGTGTTGGGAGCCTACT
G--
CTTTTGCTAGCTGTAGCTCCTGAAATACAACGGCGGATCTGCGATATCCTCTGAGCGTAGTAA-
TTTTTATCTCGCTTTTGACTGGAGTTGCAGCGTCTTTAGCCGCTAAATCCCCCAAA-
TTTTTAATGGTTGACCTC??
??
??
??
??
??
TGCTGCTTTCTGGTATGTAGCC-CATCTACCTCTACGC-GCCTCAATACGACA-

CCCCCGGCAACTCGACGAC--GACGTTCTCAAC---
TGCTCGGTTGGAACCAAATGAAAGACTTGATACTGACCGGTCTCTGATAGGCAAACCATCTCTG
GCGAGCACGGTCTCGACAGCAATGGAGTGTACGTACCCTTTCCCTGGCTACTCGCTTTCCCACG
AACATGCCAGCTAACACTCG-
TGGTTGTGAAGCTACAACGGTACCTCCGAGCTCCAGCTCGAGCGCATGAGCGTCTACTTCAACG
AGGCTTCCGGCAACAAGTACGTTCCTCGTGCCGTCCTCGTCGATCTCGAGCCCGGTACCATGGA
TGCCGTCCGCGCCGGTCCTTTCGGTCAGCTCTTCCGCCCTGACAACTTCGTCTTCGGTCAGTCCG
GTGCC
>P_dilucida_LC3232
???????????????????????????????ACCCATGTGAACTTACC-
ATTGTTGCCTCGGCAGAAGCTGCTCGGT-
ATACCTTACCTTGGAACGGCCTACCCTGTAGCGCCTTACCCTGGAACGGCTTACCCTGTGACGG
CTGCCGGTGGACTACCAAACTCTTGTTATTTTATTGTAATCTGAGCGTCTTATTTTAATAAGTCAA
AACTTTCAACAACGGATCTCTTGGTTCTGGCATCGATGAAGAACGCAGCGAAATGCGATAAGTA
ATGTGAATTGCAGAATTCAGTGAATCATCGAATCTTTGAACGCACATTGCGCCCATTAGTATTCT
AGTGGGCATGCCTGTTCGAGCGTCATTTCAACCCTTAAGCCTAGCTTAGTGTTGGGAGCCTACT
G--
CTTTTGTTAGCTGTAGCTCCTGAAATACAACGGCGGATCTGCGATATCCTCTGAGCGTAGTAA-
TTTTTATCTCGCTTTTGACTGGAGTTGCAGCGTCTTTAGCCGCTAAACCCCCC-
AATTTTTAAATGGTTGACCTCAGGTTAGTCATCCTCGCAATCCC-ATCATCC------CCATC-----
---CTCCTCATAATCGCCT--CGCAAA----CA--TTTCCTCCC-GGTGCCGAAAATCC-G-------
-TTCGCGCACCTGCC-CATTTTTCCAA--ACACTTACCCC--------
GCCGCACGACCCCGCGGTGCAAACGAAAAATTTCTTA--
TCACAGCCCCACATCGCACAAACATTTTGGCATCCATGCACGTTCCATGACCCACCATGAACAA
TTGCTGACCCCGCCAAATAGGAAGCCGCCGAGCTCGGAAAGGGTTCCTTCAAGTACGCATGGG
TTCTTGACAAGCTCAAGGCCGAGCGTGAGCGTGGTATCACCATTGATATCGCTCTCTGGAAGTT
CGAGACCAACGAGTACAATGTCACTGTCATTGGTTAGTATCCCTGCTCACAACATGTATCATGTC
TCCGAATTT-
AGACTAACATTACGATCCAGACGCTCCCGGTCACCGTGATTTCATCAA????GCTTTCTGGTATGT
AGCC-CATCTACCTCGACAC-GCCTCAATACGACG-CCTCCCGCAACTCGACAAC--
GACGTTCTCACC---
TACTTGGTCGGAACCAAACAAAAGACATGATACTGACCGGTCTCTGATAGGCAAACCATTTCTG
GCGAGCACGGTCTCGACAGCAATGGAGTGTACGTACCCTTTCCTTGGCTACTTGCTTTCCCATG
AACGTGTTAGCTAACACTCG-
TCCTTGCTCAGCTACAACGGTACCTCCGAGCTCCAGCTCGAGCGCATGAGCGTCTACTTCAACG
AGGCTTCCGGCAACAAGTACGTTCCTCGTGCCGTCCTCGTCGATCTCGAGCCCGGTACCATGGA
TGCCGTCCGCGCCGGTCCTTTCGGTCAGCTCTTCCGCCCTGACAACTTCGTCTTCGGTCAGTCCG
GTGCC
>P_diploclisiae_CBS_115587
AGGGATCATTATAGAGTTTTCTAAACTCCCAACCCATGTGAACTTACC-
ATTGTTGCCTCGGCAGAAGCTGCTCGGT-
GCACCTTACCTTGGAACGGCCTACCCTGTAGCGCCTTACCCTGGAACGGCTTACCCTGCAACGG
CTGCCGGTGGACTACCAAACTCTTGTTATTTTATGGTTATCTGAGCGTCTTATTTTAATAAGTCAA
AACTTTCAACAACGGATCTCTTGGTTCTGGCATCGATGAAGAACGCAGCGAAATGCGATAAGTA

ATGTGAATTGCAGAATTCAGTGAATCATCGAATCTTTGAACGCACATTGCGCCCATTAGTATTCT
AGTGGGCATGCCTGTTCGAGCGTCATTTCAACCCTTAAGCCTAGCTTAGTGTTGGGAGCCTACT
G--
CTTTTGCTAGCTGTAGCTCCTGAAATACAACGGCGGATCTGCGATATCCTCTGAGCGTAGTAA-
TTTTTATCTCGCTTTTGACTGGAGTTGCAGCGTCTTTAGCCGCTAAACCCCCC-AA-
TTTTTAATGGTTGACCTCAGGTTAGTCATGCTCACAGTCCC-ATCATCC------TCATC---------
--ACCATCATCGCCT--CGCAAA----CA-TTTTCCAACC-GTTGCCGAAATTCT-G--------
TTTTCGCACCTGCC-CATTTTCCCAG--ACAATTACCCC--------
GCCGCACGACCCCGCGGTGCAAACGAAAAATTTCTTA--TCACAGCCCCACTTCACAC-
AACATTTTGGCAGCCACGCACTTTGCATGACCCACAATGAACAATTGCTGACCCCGCCAAATAG
GAAGCCGCCGAGCTCGGAAAGGGTTCCTTCAAGTACGCATGGGTTCTTGACAAGCTCAAGGCC
GAGCGTGAGCGTGGTATCACCATTGATATCGCTCTCTGGAAGTTCGAGACCAACGAGTACAATG
TCACCGTCATTGGTTAGTATCCCTGTCCACAGAAAGTATCATGTGTCCGAACTC-
AGACTAACATCGCGATACAGACGCTCCCGGTCACCGTGATTTCATCAATGCTGCCTTCTGGTAT
GTAGTC-CATCTACCTCGACAC-GCCTCAATACGACA-CCCTCCGCAACTCGACGAC--
GGCATTCTCGGC---
TACTTGGTTGGAACCAAACGAAAGACTTGATACTGACCGGTCTCTGATAGGCAAACCATCTCTG
GCGAGCACGGTCTCGACAGCAATGGAGTGTACGTACCGTTTCCTTGGCTACTTGCTTTCCCACG
AACATGTTAGCTAACACTCG-
TGCTTGCTCAGCTACAACGGCACCTCCGAGCTCCAGCTCGAGCGCATGAGCGTCTACTTCAACG
AGGCTTCCGGCAACAAGTACGTTCCTCGTGCTGTCCTCGTCGATCTCGAGCCCGGTACCATGGA
CGCCGTCCGCGCCGGTCCTTTCGGCCAGCTCTTCCGCCCTGACAACTTCGTCTTCGGTCAGTCC
GGTGCC
>P_diversiseta_MFLUCC_12_0287
??????CATTATAGAGTTTTTTAAACTCCCAACCCATGTGAACTTACC-
ATTGTTGCCTCGGCAGAGGCTACCCGGT-
ACACCTTACCCTGGAACGGCCTACCCTGTAGCGCCTTACCCTGGAACGGCTTACCCTGTAGCGG
CTGCCGGTGGACTACTAAACTCTTGTTATTTTATTGTAATCTGAGCGTCTTATTTTAATAAGTCAA
AACTTTCAACAACGGATCTCTTGGTTCTGGCATCGATGAAGAACGCAGCGAAATGCGATAAGTA
ATGTGAATTGCAGAATTCAGTGAATCATCGAATCTTTGAACGCACATTGCGCCCATTAGTATTCT
AGTGGGCATGCCTGTTCGAGCGTCATTTCAACCCTTAAGCCTAGCTTAGTGTTGGGAGTCTACTG
--
CTTTTACTAGCTGTAGCTCCTGAAATACAACGGCGGATCTGCGATATCCTCTGAGCGTAGTAA-
TTTTTTTCTCGCTTTTGACTGGAGTTGCAGCGTCTTTAGCCGCTAAACCCCC--AA-
TTTTTAATGGTTGACCTCAGGTTAGTCATCCTCACAATCCC-ATCATCA-----------------
TCTTCATCACCATCACCT--CGCACA----CA-TTTTCAACTC-AGTGCCGAAAATCA----------
GTTTCGCACCTGCC-CACATTCCCAG--
ACACTTACCCCGCCGCGTGGCCGCACGACCCCGCGGTGCAAACGAAAAATTTCTTA--T--
CAGCCCCACATCACACAAACATTTTGGCGGCCATGCACTTTTCGTAACCCACAATGAGCAATTG
CTGACCCTGCCAAATAGGAAGCCGCCGAGCTCGGAAAGGGTTCCTTCAAGTACGCATGGGTTC
TTGACAAGCTCAAGGCCGAGCGTGAGCGTGGTATCACCATCGATATCGCTCTCTGGAAGTTCGA
GACCAACGAGTACAATGTCACCGTCATTGGTCAGTATCCCTGCCCACAAGCTCTCTCATGGATC
CGAACTC-
ATACTAACATCGCAATATAGATGCTCCCGGTCACCGTGATTTCATCAATGCTGCTTTCTGGTATG

TAGCC-CATCTACCTCGATAC-GCCTCAATACGACG-CCTCCCGCAACTCAACAAC--
GACGTTCTCAAC---
TACTTGGTTGGAACCAAACGAAAGACTTGATACTGACCCGTCTCTGATAGGCAAACCATCTCTG
GCGAGCACGGCCTCGACAGCAATGGAGTGTACGTATCCGTTCCTTGGCTACTTGCTTTCCCACG
AACATGCTAGCTGACATCCG-
TGGTTGTTCAGCTACAACGGTACCTCCGAGCTCCAGCTCGAGCGCATGAGCGTCTACTTCAACG
AGGCTTCCGGCAACAAGTACGTTCCTCGTGCCGTCCTCGTCGATCTCGAGCCCGGTACCATGGA
TGCCGTCCGCGCCGGTCCTTTCGGTCAGCTCTTCCGCCCTGACAACTTCGTCTTCGGTCAGTCCG
GTGCC
>P_dracaenae_HGUP4037
???
???
???
???
???
???
???????????????????????????ATCCTCA--------------------TCACCATCATCGCCT--
CGCAAA----CA-TTTTCCAACC-GTTGCCGAAATTCT-G--------TTTTCGCACCTGCC-
CATTTTCCCAG--ACAATTACCCC--------
GCCGCACGACCCCGCGGTGCAAACGAAAAATTTCTTA--TCACAGCCCCACTTCACAC-
AACATTTTGGCAGCCACGCACTTTGCATGACCCACAATGAACAATTGCTGACCCCGCCAAATAG
GAAGCCGCCGAGCTCGGAAAGGGTTCCTTCAAGTACGCATGGGTTCTTGACAAGCTCAAGGCC
GAGCGTGAGCGTGGTATCACCATTGATATCGCTCTCTGGAAGTTCGAGACCAACGAGTACAATG
TCACCGTCATTGGTTAGTATCCCTGTCCACAGAAAGTATCATGTGTCCGAACTC-
AGACTAACATCGCGATACAGACGCTCCCGGTCACCGTGATTTCATCAA???????ATCAGGT-----
----CATCTA-CTCGACAC-GCCTC-ATACGACA-CCCTCCGCAACTCGACGAC--
GACATTCTCGGC---TACTTGGTTGGAACCAAACGAAAGAC-
TTATACTGACCGGTCTCTGATAGGCAAACCATCTCTGGCGAGCACGGTCTCGACAGCAATGGAG
TGTACGTACCATTTCCTTGCCTACTTGCTTTCCCACGGACTTGTTAGCTAACACTCG-
TGCTTGCTCAGCTACAACGGCACCTCCGAGCTCCAGCTCGAGCGCATGAGCGTCTACTTCAACG
AGGCTTCCGGCAACAAGTACGTTCCTCGTGCTGTCCTCGTCGATCTCGAGCCCGGTACCATGGA
CGCCGTCCGTGCCGGTCCTTTCGGCCAGCTCTTCCGCCCTGACAACTTCGTCTTCGGTCAGTCCG
GTGCC
>P_dracaenicola_MFLUCC_18_0913
??????CATTATAGAGTTTTCTAAACTCCCAACCCATGTGAACTTACC-
ATTGTTGCCTCGGCAGAAGCTGCTCGGC-
GCGCCTTACCTTGGAACGGCCTACCCTGTAGCGCCTTACCCTGGAACGGCTTACCCTGCAACGG
CTGCCGGTGGACTACCAAACTCTTGTTATTTTATTGTTATCTGAGCGTCTTATTTTAATAAGTCAA
AACTTTCAACAACGGATCTCTTGGTTCTGGCATCGATGAAGAACGCAGCGAAATGCGATAAGTA
ATGTGAATTGCAGAATTCAGTGAATCATCGAATCTTTGAACGCACATTGCGCCCATTAGTATTCT
AGTGGGCATGCCTGTTCGAGCGTCATTTCAACCCTTAAGCCTAGCTTAGTGTTGGGAGCCTACT
G--
CTTTTGCTAGCTGTAGCTCCTGAAATACAACGGCGGATCTGCGATATCCTCTGAGCGTAGTAA-
TTTTTATCTCGCTTTTGACTGGAGTTGCAGCGTCTTTAGCCGCTAAACCCCCC-AA-

TTTTTAATGGTTGACCTC????????????????????CCC-ATCATCC------TCATC-----------
ATCATCATCGCCT--CGCAAA----CA--TTTCCAACC-GGTGCCGAAATTCT----------
TTTTCGCACCTGCC-CATTTTTCCAG--ACACTTACCCC--------
GCCGCACGACCCCGCGGTGCAAACGAAAAATTTCTTA--TCACAGCCCCACTTCACAC-
AACATTTTGGCGGCCACGCACTTTCCATGACCCACAATGAACAATTGCTGACCCCGCCAAATAG
GAAGCCGCCGAGCTCGGTAAGGGTTCCTTCAAGTACGCATGGGTTCTTGACAAGCTCAAGGCC
GAGCGTGAGCGTGGTATCACCATTGATATCGCTCTCTGGAAGTTCGAGACCAACGAGTACAATG
TCACCGTCATTGGTTAGTATCCCTGTCCACAGAATGTATCATGTGTCCGAACTC-
AGACTAACATCGCAATACAGACGCTCCCGGTCACCGTGATTTCATCAATGCTGCCTTCTGGTAT
GTAGCC-CATCTACCTCGACGC-GCCTCAATACGACA-CCCTCGGCAACTCGACGAC--
GACATTCTCGGC---
TACTTGGTTGGAACCAAACGAAAGACTTGATACTGACCGGTCTCTGATAGGCAAACCATCTCTG
GCGAGCACGGTCTCGACAGCAATGGAGTGTACGTACCGTTTCCTTGGCTACTTGCTTTCCCACG
GACTTGTTAGCTAACACTCG-
TGCTTGCTCAGCTACAACGGCACCTCCGAGCTCCAGCTCGAGCGCATGAGCGTCTACTTCAACG
AGGCTTCCGGCAACAAGTACGTTCCTCGTGCTGTCCTCGTCGATCTCGAGCCCGGTACCATGGA
CGCCGTCCGCGCCGGTCCTTTCGGCCAGCTCTTCCGCCCTGACAACTTCGTCTTCGGTCAGTCC
GG????
>P_dracontomelon_MFUCC_10_0149
??????CATTATAGAGTTTTCTAAACTCCCAACCCATGTGAACTTACC-
ATTGTTGCCTCGGCAGAAGCTGCTCGGT-
GCACCTTACCTTGGAACGGCCTACCCTGTAGCGCCTTACCC-
GGAACGGCTTACCCTGTAGCGGCTGCCGGCGGACTACCAAACTCTTGTTATTTTATTGTAATCTG
AGCGTCTTATTTTAATAAGTCAAAACTTTCAACAACGGATCTCTTGGTTCTGGCATCGATGAAGA
ACGCAGCGAAATGCGATAAGTAATGTGAATTGCAGAATTCAGTGAATCATCGAATCTTTGAACG
CACATTGCGCCCATTAGTATTCTAGTGGGCATGCCTGTTCGAGCGTCATTTCAACCCTTAAGCCT
AGCTTAGTGTTGGGAGCCTACTG--
CTTTTGCTAGCTGTAGCTCCTGAAATACAACGGCGGATCTGCGATATCCTCTGAGCGTAGTAA-
TTTTTATCTCGCTTTTGACTGGAGTTGCAGCGTCTTTAGCCGCTAAACCCCCC-AA-
TTTTTAATGGTTGACCTC????TAGTCATGCTCACA-TCCC-ATCATCC--------------------
TCATCATCATCACCT--CGCAAA----CA-TTTCCACGCC-GGTGCCGAAAATCTGG--------
TTTTCGCACCTGCC-CATTTTCCCGG--ACACTTACCCC--------
GCCGCACGACCCCGCGGTGCAAACGAAAAATTTCTTA--
TCATAGCCCCACATCACACAAACATTTTGGCAGCCACGCACTTTGCAAGACCCACAACGAACAA
TTGCTGACCCCGCCAAATAGGAAGCCGCCGAGCTCGGAAAGGGTTCCTTCAAGTACGCATGGG
TTCTTGACAAGCTCAAGGCCGAGCGTGAGCGTGGTATCACCATTGATATCGCTCTCTGGAAGTT
CGAGACCAACGAGTACAATGTCACCGTCATTGGTTAGTATCCCCGTCCACAACATGCATCATGT
CTCCGAACTCAAGACTAACCTTGCCATACAGATGCTCCCGGTCACCGTGATTTCATCAA????????
??
??
??
??
??
>P_endophytica_MFLUCC_18_0932

??????CATTATAGAGTTTTCTAAACTCCCAACCCATGTGAACTTACC-
ATTGTTGCCTCGGCAGAAGCTACCTGGT--
TACCCTACCTTGGAACGGCCTACCCTGTAGCGCCTTACCCTGGAACGGCTTACCCTGTAACGGC
TGCCGGTGGACTACCAAACTCTTGTTATTTTATTGTAATCTGAGCGTCTTATTTTAATAAGTCAAA
ACTTTCAACAACGGATCTCTTGGTTCTGGCATCGATGAAGAACGCAGCGAAATGCGATAAGTAA
TGTGAATTGCAGAATTCAGTGAATCATCGAATCTTTGAACGCACATTGCGCCCATTAGTATTCTA
GTGGGCATGCCTGTTCGAGCGTCATTTCAACCCTTAAGCCTAGCTTAGTGTTGGGAGCCTACTG-
-CTTTTGCTAGCTGTAGCTCCTGAAATACAACGGCGGATCTGCGATATCCTCTGAGCGTAGTAA-
TTTTTATCTCGCTTTTGACTGGAGTTGCAGCGTCTTTAGCCGCTAAACCCCCC-AA-
TTTTTAATGGT-GACCTCATCTCCCCCATCCA-----TTCC---CATCC--------------------
TTATCATTATCACCT--CGCAAA----TA-TTGTCCGACT-GGTGCCGAAAATAT-G--------
TTTTCGCACCTGCC-CATTTTCCCAG--ACACTTACCCC--------
GCCGCACGACCCCGCGGTGCAAACGAAAAATTTCTTA--
TCACAGCCCCACATCGCACAAACATTTTGGCAGCCATGCACTTTACAAGACCCACAATGAACAA
TTGCTGACCCCTCCAAATAGGAAGCCGCCGAGCTCGGAAAGGGTTCCTTCAAGTACGCATGGG
TCCTTGACAAGCTCAAGGCCGAGCGTGAGCGTGGTATCACCATTGATATCGCTCTCTGGAAGTT
CGAGACCAACGAGTACAATGTCACCGTCATTGGTTAGTGTCCCTGTCCACACGATGTATCATGC
ATCCGAACGT-
ACACTAACATGGCAATACAGATGCTCCCGGTCACCGTGATTTCATCAA????????????????????????
??
??
??
??
??
>P_ericacearum_IFRDCC_2439
??????CATTATAGAGTTTTCTAAACTCCCAACCCATGTGAACTTACC-
ATTGTTGCCTCGGCAGAGGCTACCCGGT----
ACCTACCCTGGAACGGCCTACCCTGTAGCGCCTTACCCGGGAACGGGCTACCCTGTAGCGGCT
GCCGGTGGACTACCAAACTCTTGTTATTTTATGGTTATCTGAGCGTCTTATTTTAATAAGTCAAAA
CTTTCAACAACGGATCTCTTGGTTCTGGCATCGATGAAGAACGCAGCGAAATGCGATAAGTAAT
GTGAATTGCAGAATTCAGTGAATCATCGAATCTTTGAACGCACATTGCGCCCATTAGTACTCTAG
TGGGCATGCCTGTTCGAGCGTCATTTCAACCCTTAAGCCTAGCTTAGTGTTGGGAGCCTACTG--
CTTTTGCTAGCTGTAGCTCCTGAAATACAACGGCGGATCTGCGGTATCCTCTGAGCGTAGTAA-
TTTTTTTCTCGCTTTTGACTGGAATTGCAGCGTCCTTAGCCGCTAAATCCCCC-AA-
TTTCTAATGGTTGACCTCAGGTTGGTTATCCTCAAAATCCC-ATCATCA--------------------
TGCCCATCATCGCCTCCCTCAAA----TA-TTTTCAACTC-GGTGCCGAGAACCA---------
TTATTCGCACCTGCC-CACATTCTCTGACACACTTACCCC--------
GCCGCGCGACCCCGCGGTGCAAACGAAAAATTTCTTA--
TCACAGCCCCACCTCGCACAAACATTTTCGCAGCCATGCA-
TCTCCATGATCCACAATGAGCAACTGCTGACCCCGCCAAACAGGAAGCCGCCGAGCTCGGTAA
GGGTTCCTTCAAGTACGCCTGGGTTCTTGACAAGCTCAAGGCCGAGCGTGAGCGTGGTATCACC
ATCGATATCGCTCTCTGGAAGTTCGAGACCAACGAGTACAATGTCACCGTCATTGGTTAGTCTAC
CCGTCCACAAGAAGGATCATTCATCCGACATC-
ATACTAACATGGCAATACAGACGCTCCCGGTCACCGTGACTTCATCAATGCTGCTTTCTGGTATG

TAGCC-TATCTACCTCGACAC-GCCTCAACACGACG-CCTCCCGCAACTCGACAAC--
GACGTTCTCAAC---
TATCTGGTTGGAACCAAACGAAAGACTTGATACTGACCCGTCTTTGATAGGCAAACCATCTCTG
GCGAGCACGGTCTCGACAGCAATGGAGTGTACGTACCC-TTCCTTGGCTACCTACTTTTCGATG-
--------GCTAATACTCG----
TCGTTCAGCTACAACGGTACCTCCGAGCTCCAGCTCGAGCGCATGAGCGTCTACTTCAACGAGG
CTTCCGGCAACAAGTACGTTCCTCGTGCCGTCCTCGTCGATCTCGAGCCCGGTACCATGGATGC
CGTCCGCGCCGGTCCTTTCGGTCAGCTCTTCCGCCCTGACAACTTCGTCTTCGGTCAGTCCGGTG
CC
>P_etonensis_BRIP_66615
AGGGATCATTATAGAGTTTTCTAAACTCCCAACCCATGTGAACTTACC-
ATTGTTGCCTCGGCAGAAGCTGCTCGGT-
GCACCTTACCTTGGAACGGCCTACCCTGTAGCGCCTTACCCTGGAACGGCTTACCCTGTAGCGG
CTGCCGGCGGACTACCAAACTCTTGTTATTTTATTGTAATCTGAGCGTCTTATTTTAATAAGTCAA
AACTTTCAACAACGGATCTCTTGGTTCTGGCATCGATGAAGAACGCAGCGAAATGCGATAAGTA
ATGTGAATTGCAGAATTCAGTGAATCATCGAATCTTTGAACGCACATTGCGCCCATTAGTATTCT
AGTGGGCATGCCTGTTCGAGCGTCATTTCAACCCTTAAGCCTAGCTTAGTGTTGGGAGCCTACT
G--
CTTTTGCTAGCTGTAGCTCCTGAAATACAACGGCGGATCTGCGATATCCTCTGAGCGTAGTAA-
TTTTTATCTCGCTTTTGACTGGAGTTGCAGCGTCTTTAGCCGCTAAACCCCCC-AA-
TTTTTAATGGTTGACCTCAGGTTAGTCATCCTCGCAATCCC-ATCATCC------TCATC---------
--ATCATCATCACCT--CACAAA----CA-TTTCCACACC-GGTGCCGAAAATCTGG--------
TTTTCGCACCTGCC-CATTTTCCCAT--ACACTTACCCC--------
GCCGCACGACCCCGCGGTGCAAACGAAAAATTTCTTA--TCATAGCCCCACATCAC--
AAACATTTTGGCAGCCACGCACTCTGCATGACCCACAATGAACAATTGCTGACCCCGCCAAATA
GGAAGCCGCCGAGCTCGGAAAGGGTTCCTTCAAGTACGCATGGGTTCTTGACAAGCTCAAGGC
CGAGCGTGAGCGTGGTATCACCATTGATATCGCTCTCTGGAAGTTCGAGACCAACGAGTACAAT
GTCACCGTCATTGGTTAGTATCCCTGCCCACAACTTGTGTCATGTCTCCGAACTCAAGACTAACC
TTACAATACAGACGCTCCCGGTCACCGTGATTTCATCAATGCTGCCTTCTGGTATGTAGCC-
CATCTACCTCGACGC-GTCTCAATACGACA-CCCCCGGCAACTCGACAAC--GACGTTCTCAAC-
--
TGCTTGGTTGAAACCAAATGAAAGACTTGATACTGACCGGTCTATGATAGGCAAACCATTTCTG
GCGAGCACGGTCTCGACAGCAATGGAGTGTACGTACCCTTTCCCTGGCTACTCGCCTTCTCGTG
AACATGTCAGCTAACAGTCG-
TGCTTGTTCAGCTACAACGGTACCTCCGAGCTCCAGCTCGAGCGCATGAGCGTCTACTTCAACG
AGGCTTCCGGCAACAAGTACGTTCCTCGTGCCGTCCTCGTCGATCTCGAGCCCGGTACCATGGA
TGCCGTCCGCGCCGGTCCTTTCGGTCAGCTCTTCCGCCCTGACAACTTCGTCTTCGGTCAGTCCG
GTGCC
>P_formosana_NTUCC_17_009
??????CATTATAGAGTTTTCTAAACTCCCAACCCATGTGAACTTACC-
ATTGTTGCCTCGGCAGAAGCTGCTCGGT-
GCACCTTACCTTGGAACGGCCTACCCTGTAGCGCCTTACCCTGGAACGGCTTACCCTGTAGCGG
CTGCCGGTGGACTACCAAACTCTTGTTATTTTATTGTAATCTGAGCGTCTTATTTTAATAAGTCAA
AACTTTCAACAACGGATCTCTTGGTTCTGGCATCGATGAAGAACGCAGCGAAATGCGATAAGTA

ATGTGAATTGCAGAATTCAGTGAATCATCGAATCTTTGAACGCACATTGCGCCCATTAGTATTCT
AGTGGGCATGCCTGTTCGAGCGTCATTTCAACCCTTAAGCCTAGCTTAGTGTTGGGAGCCTACT
G--
CTTTTACTAGCTGTAGCTCCTGAAATACAACGGCGGATCTGCGATATCCTCTGAGCGTAGTAA-
TTTTTATCTCGCTTTTGACTGGAGTTGCAGCGTCTTTAGCCGCTAAACCCCCC-AA--
TTTTAATGGT???????????????????????????ATCT-ATCATCC------TCATC--------
TTCATCATCATCACCT--CGCAAA----GA-TTTCCACACC-GGTGCCGAAAATCT-G--------
TTTTCGCACCTGCC-CATTTTCCCAG--ACACTTACCCC--------
GCCGCACGACCCCGCGGTGCAAACGAAAAATTTCTTA--TCATAGCCCCACATCAC--
AAACATTTTGGCAGCCACGCACTCTGCATGACCCACAATGAACAATTGCTGACCCCGCCAAATA
GGAAGCCGCTGAGCTCGGAAAGGGTTCCTTCAAGTACGCATGGGTTCTTGACAAGCTCAAGGC
CGAGCGTGAGCGTGGTATCACCATTGATATCGCTCTCTGGAAGTTCGAGACCAACGAGTACAAT
GTCACCGTCATTGGTTAGTATCCCTGCCCACAACATGTGTCATGTCTCCGAACTCAAGACTAACC
TTACAATACAGACGCTCCCGGTCACCGTGATTTCATCAA????????????????????????????????????
????????CATACGACA-CCCCCGGCAACTCGACAAC--GACGTTCTCAAC---
TGCTTGGTTGAAACCAAATGAAAGACTTGATACTGACCGGTCTCTGATAGGCAAACCATCTCTG
GCGAGCACGGTCTCGACAGCAATGGAGTGTACGTACCCTTTCCCTGGCTACTCGCTTTCTCGTG
AACATGTCAGCTAACACTCG-
TGCTTGTTCAGCTACAACGGTACCTCCGAGCTCCAGCTCGAGCGCATGAGCGTCTACTTCAACG
AGGCTTCCGGCAACAAGTACGTTCCTCGTGCCGTCCTCGTCGATCTCGAGCCCGGTACCATGGA
TGCCGTCCGCGCCGGTCCTTTCGGTCAGCTCTTCCGCCCTGACAACTTCGTCTTCGGTCAGTCCG
GTGCC
>P_furcata_MFLUCC_12_0054
??????CATTATAGAGTTTTTTAAACTCCCAACCCATGTGAACTTACC-
ATTGTTGCCTCGGCAGAAGCTACCCGGT---
ACCTTACCTTGGAACGGCCTACCCTGTAGCGCCTTACCCTGGAACGGCCTACCCTGTAACGGCT
GCCGGTGGACTACCAAACTCTTGTTATTTTTATGTTATCTGAGCGTCTTATTTTAATAAGTCAAAA
CTTTCAACAACGGATCTCTTGGTTCTGGCATCGATGAAGAACGCAGCGAAATGCGATAAGTAAT
GTGAATTGCAGAATTCAGTGAATCATCGAATCTTTGAACGCACATTGCGCCCATTAGTATTCTAG
TGGGCATGCCTGTTCGAGCGTCATTTCAACCCTTAAGCCTAGCTTAGTGTTGGGAGCCTACTG--
CTTTTGTTAGCTGTAGCTCCTGAAATACAACGGCGGATCTGCGATATCCTCTGAGCGTAGTAATT
TTTTTCCTCGCTTTTGACTGGAGTTGCAGCGTCTTTAGCCGCTAAACCCCCC-AA-
TTTTTAATGGTTGACCTCAGGTTAGTTATCCTCACAATCCC-ATCATCC-------------------
TCACCATCATTGCCT--CGCAAA----CA-TTTCCAAACC-GGCGTCGAAAATCTGG--
TGTGTTTTTTCGCGCCTGCC-CACATTCCCAG--ACACTTACCCC--------
GCCGCACGACCCCGCGGTGCAAACGAAAAATTTCTTA--
TCACAGCCCCACATCGCACAAACATTCTGGCAGCCATGAACTTTACATAACCCAT-
ACGAGCATCTGCTGACCCCGCCAAATAGGAAGCCGCCGAGCTCGGAAAGGGTTCCTTCAAGTA
CGCATGGGTTCTTGACAAGCTCAAGGCCGAGCGTGAGCGTGGTATCACCATCGATATCGCTCTC
TGGAAGTTCGAGACCAACGAGTACAATGTCACCGTCATTGGTTAGTATCCCTGTCCACAACATC
CGACATGCATCCAAAATC-
AGACTAACACGGCACCACAGATGCTCCCGGTCACCGTGATTTCATCAATGCTGCTTTCTGGTAT
GTAGTT-CATCTACCTCGACGC-GCCTCAGCACGACG-CCTCTCGCAACTCGACAAC--
GACGTTATCAAC---TACTTGGTT-

AAACCAAACGAAAGACTTGATACTGACAGGCCTCTGATAGGCAAACCATCTCTGGCGAGCACG
GTCTCGACAGCAATGGAGTGTACGTACCCTTCCCTTGGCTACCTGCT-
TCCCACGAACATGTCAGCTAACACTCG----
TGGTTCAGCTACAACGGTACCTCCGAGCTCCAGCTCGAGCGCATGAGCGTCTACTTCAACGAGG
CTTCCGGCAACAAGTACGTTCCTCGTGCCGTCCTCGTCGATCTCGAGCCCGGTACCATGGATGC
CGTCCGCGCCGGTCCTTTCGGTCAGCTCTTCCGCCCTGACAACTTCGTCTTCGGTCAGTCCGGTG
CC
>P_gaultheria_IFRD_411_014
??????CATTATAGAGTTTT--AAACTCCCAACCCATGTGAACTTACC-
ACTGTTGCCTCGGCAGAGGCTACCCGGT-
ACACCCTACCCTGGAACGGCCTACCCTGTAGCGCCTTACCCTGGAACGGCTTACCCTGTAGCGG
CTGCCGGTGGACTACCAAACTCTTGTTATTTTATTGTAATCTGAGCGTCTTATTTTAATAAGTCAA
AACTTTCAACAACGGATCTCTTGGTTCTGGCATCGATGAAGAACGCAGCGAAATGCGATAAGTA
ATGTGAATTGCAGAATTCAGTGAATCATCGAATCTTTGAACGCACATTGCGCCCATTAGTACTCT
AGTGGGCATGCCTGTTCGAGCGTCATTTCAACCCTTAAGCCTAGCTTAGTGTTGGGAGCCTGCA
G--
CCTTTACTGGCTGCAGCTCCTGAAATACAACGGCGGATCTGCGATATCCTCTGAGCGTAGTAAT
TTTTTTTCTCGCTTTTGACTGGAGTTGCACCGTCTTTACCCCCTAAACCCCCC-AA-
TTTTTAATGGTTGACCTCATGTTAGTCATCCTCAAAATCCC-ATCATCA---------------------
--TCACCATCACCTGGCACGCA-------TTTCCAACGC-GGTGCCGAAAATCA-G--------
TTTTCGCACTTGCC-CATATTCCCAG--
ACACTTACCCCGCCGCGTGGCCGCACGACCCCGCGATGCAAACGAAAAATTTCTTA--T--
CAGCCCCACATCGCACAAACATTTTGGCAGCCATGCACTTTTCATAATCCACAATGAGCAATTG
CTGACCCCGTCAAATAGGAAGCCGCCGAGCTCGGTAAGGGTTCCTTCAAGTACGCATGGGTTCT
TGACAAGCTCAAGGCCGAGCGTGAGCGTGGTATCACCATCGATATCGCTCTCTGGAAGTTCGA
GACCAACGAGTACAATGTCACCGTCATTGGTCAGTATCCCTGTCCACTAGCTGTTGCATGCATCC
GAACTC-
AGACTAATATGGCAATATAGATGCTCCCGGTCACCGTGATTTCATCAATGCTGCTTTCTGGTATG
TAGCC-TATCTACCTCGACAC-GCCTCAATATGATG-CCTCCCGCAACTCGACGCC--
GACGTTCTCAAA---TACTTGGTTGGATCCAAACGGAAGACTTGATACTGACCC-
TCTCTGATAGGCAAACCATCTCTGGCGAGCACGGTCTCGACAGCAATGGAGTGTACGTGCCCTT
TCCTTGGCTACCTGCTTTCCCACGAACATGCTAGCTAACCCTCG-
TGGTTGTTCAGCTACAACGGTACCTCCGAGCTCCAGCTCGAGCGCATGAGCGTCTACTTCAACG
AGGCTTCCGGCAACAAGTACGTTCCTCGTGCCGTCCTCGTCGATCTCGAGCCCGGTACCATGGA
TGCCGTTCGCGCCGGTCCTTTCGGTCAGCTCTTCCGCCCTGACAACTTCGTCTTCGGCCAGTCCG
GT-CC
>P_gibbosa_NOF_3175
AGGGATCATTATAGAGTTTT--AAACTCCCAACCCATGTGAACTTACC-
ACTGTTGCCTCGGCAGAGGCTACCCGGT-
ACACCCTACCCTGGAACGGCCTACCCTGTAGCGCCTTACCCTGGAACGGCTTACCCTGTAGCGG
CTGCCGGTGGACTACCAAACTCTTGTTATTTTATTGTAATCTGAGCGTCTTATTTTAATAAGTCAA
AACTTTCAACAACGGATCTCTTGGTTCTGGCATCGATGAAGAACGCAGCGAAATGCGATAAGTA
ATGTGAATTGCAGAATTCAGTGAATCATCGAATCTTTGAACGCACATTGCGCCCATTAGTACTCT
AGTGGGCATGCCTGTTCGAGCGTCATTTCAACCCTTAAGCCTAGCTTAGTGTTGGGAGCCTGCA

G--
CCTTTACTGGCTGCAGCTCCTGAAATACAACGGCGGATCTGCGATATCCTCTGAGCGTAGTAAT
TTTTTTTCTCGCTTTTGACTGGAGTTGCAGCGTCTTTAGCCGCTAAACCCCCC-AA-
TTTTTAATGGTTGACCTCAGGTTAGTCATCCTCAAAATCCC-ATCATCA--------------------
---TCACCATCACCTGGCACGCA-------TTTTCAACGC-GGTGCCGAAAATCA-G--------
TTTTCGCACTTGCC-CATATTCCCAG--
ACACTTACCCCGCCGCGTGGCCGCACGACCCCGCGATGCAAACGAAAAATTTCTTA--T--
CAGCCCCACATCGCACAAACATTTTGGCAGCCATGCACTTTTCATAATCCACAATGAGCAATTG
CTGACCCCGTCAAATAGGAAGCCGCCGAGCTCGGTAAGGGTTCCTTCAAGTACGCATGGGTTCT
TGACAAGCTCAAGGCCGAGCGTGAGCGTGGTATCACCATCGATATCGCTCTCTGGAAGTTCGA
GACCAACGAGTACAATGTCACCGTCATTGGTCAGTATCCCTGTCCACTAGCTGTTGCATGCATCC
GAACTC-
AGACTAATATGGCAATATAGATGCTCCCGGTCACCGTGATTTCATCAA??????????TGGTATGTA
GCC-TATCTACCTCGACAC-GCCTCAATATGATG-CCTCCCGCAACTCGACGCC--
GACGTTCTCAAA---TACTTGGTTGGATCCAAACGGAAGACTTGATACTGACCC-
TCTCTGATAGGCAAACCATCTCTGGCGAGCACGGTCTCGACAGCAATGGAGTGTACGTGCCCTT
TCCTTGGCTACCTGCTTTCCCACGAACATGCTAGCTAACCCTCG-
TGGTTGTTCAGCTACAACGGTACCTCCGAGCTCCAGCTCGAGCGCATGAGCGTCTACTTCAACG
AGGCTTCCGGCAACAAGTACGTTCCTCGTGCCGTCCTCGTCGATCTCGAGCCCGGTACCATGGA
TGCCGTTCGCGCCGGTCCTTTCGGTCAGCTCTTCCGCCCTGACAACTTCGTCTTCGGCCAGTCCG
GTGCC
>P_grevilleae_CBS_114127
AGGGATCATTATAGAGTTTTCTAAACTCCCAACCCATGTGAACTTACC-
ATTGTTGCCTCGGCAGAAGCTGCTCGGT-
GCACCCTACCTTGGAACGGCCTACCCTGTAGCGCCTTACCCTGGAACGGCTTACCCTGTAGCGG
CTGCCGGTGGACTACCAAACTCTTGTTATTTTATTGTAATCTGAGCGTCTTATTTTAATAAGTCAA
AACTTTCAACAACGGATCTCTTGGTTCTGGCATCGATGAAGAACGCAGCGAAATGCGATAAGTA
ATGTGAATTGCAGAATTCAGTGAATCATCGAATCTTTGAACGCACATTGCGCCCATTAGTATTCT
AGTGGGCATGCCTGTTCGAGCGTCATTTCAACCCTTAAGCCTAGCTTAGTGTTGGGAGCCTACT
G--
CTTTTGCTAGCTGTAGCTCCTGAAATACAACGGCGGATCTGCGATATCCTCTGAGCGTAGTAAAT
TTTTATCTCGCTTTTGACTGGAGTTGCAGCGTCTTTAGCCGCTAAATCCCCC-AA-
TTTTTAATGGTTGACCTCAGGTTAGTCATACTCGCAATCCC-ATCATCC------CCATG--------
CTCATCATCATCACCT--CGCAAA----CA-TTTCCACACC-GGTGCCGAAAATCTGG--------
TTTTCGCACCTGCC-CATTTTCCCAG--ACACTTACCCC--------
GCCGCACGACCCCGCGGTGCAAACGAAAAATTTCTTA--
TCACAGCCCCACATCACACAAACATTTTGGCAGCCACGCACTTTGTATGACCCACAATGAACAA
TTGCTGACCCCGCCAAATAGGAAGCCGCCGAGCTCGGAAAGGGTTCCTTCAAGTACGCATGGG
TTCTTGACAAGCTCAAGGCCGAGCGTGAGCGTGGTATCACCATTGATATCGCTCTCTGGAAGTT
CGAGACCAACGAGTACAATGTCACCGTCATTGGTTAGTATCCCTGCCCACAACATGCATCATGT
CTCCGAACTCAAGACTAATCTTGCAATACAGACGCTCCCGGTCACCGTGATTTCATCAATGCTG
CCTTCTGGTATGTAGCC-CATCTACCTCGACGC-
GCCTCGATACGACACCCCCCGGCAACTCGACAAC--GACATTCTCAAC---
TGCTTGTTTGGAATCATACGAAAGACTTGATACTGACCGGTCTATGATAGGCAAACCATCTCTGG

CGAGCACGGTCTCGACAGCAATGGAGTGTACGTACCCTTTCCTTGGCTACTTGCTTTCTCGTGAA
CATGTCAGCTAACACTCG-
TGCTTGTTCAGCTACAACGGTACCTCCGAGCTCCAGCTCGAGCGCATGAGCGTCTACTTCAACG
AGGCTTCCGGCAACAAGTACGTTCCTCGTGCCGTCCTCGTCGATCTCGAGCCCGGTACCATGGA
TGCCGTCCGCGCCGGTCCTTTCGGTCAGCTCTTCCGCCCTGACAACTTCGTATTCGGTCAGTCCG
GTGCC
>P_hawaiiensis_CBS_114491
AGGGATCATTATAGAGTTTTCTAAACTCCCAACCCATGTGAACTTACCAATTGTTGCCTCGGCAG
AGGCTACCCGGT---
ACCTTACCTTGGTACGGCCTACCCTGTAGCGCCTTACCCTGGAACGGGCTACCCTGTAGCGGCT
GCCGGTGGACTACCAAACTCTTGTTATTTTATGGTTATCTGAGCGTCTTATTTTAATAAGTCAAAA
CTTTCAACAACGGATCTCTTGGTTCTGGCATCGATGAAGAACGCAGCGAAATGCGATAAGTAAT
GTGAATTGCAGAATTCAGTGAATCATCGAATCTTTGAACGCACATTGCGCCCATTAGTACTCTAG
TGGGCATGCCTGTTCGAGCGTCATTTCAACCCTTAAGCCTAGCTTAGTGTTGGGAGCCTACTG--
CTTTTACTAGCTGTAGCTCCTGAAATACAACGGCGGATCTGCGATATCCTCTGAGCGTAGTAA-
TTTTTTTCTCGCTTTTGACTGGAGTTGCAGCGTCCTTTGCCGCTAAACCCCCC-AA-
TTTTTAATGGTTGACCTCAGGTTAGTCATCCTCACAATCCC-ATCATCA--------------------
-CATCCGCGGCTTCC-ACTCGAA----CA-TTTCCCACCC-GACGCCGAAAATAT-----
TTTCGCTTGCGCATCTTCC-CACATTCTC----
CCACTTACCCCGCCGCGTGGCCGCACGACCCCGCGGTGCAAACGAAAAATTTCTTA--T--
CGGCCCCACCTCGCATAAACATTTTGGCAGCCACTCACTTC-
CATGACCCACGATGAGCCATTGCTGACCCCGCCAAATAGGAAGCCGCCGAGCTCGGAAAGGGT
TCCTTCAAGTACGCCTGGGTTCTTGACAAGCTCAAGGCCGAGCGTGAGCGTGGTATCACCATCG
ATATCGCTCTCTGGAAGTTCGAGACCAACGAGTACAATGTCACCGTCATTGGTTAGTATCCCTGT
CCACAACAAGTCTCATGCATCCGAACTC-
AGACTAACATGGCAATACAGACGCTCCCGGTCACCGTGACTTCATCAATGCTGCCTTCTGGTAT
GTACCCCCATCGACCTCGACAC-GCCCCGACACGACG-
CCTCCCGCAGCTCGACAACGCGGCGTTCTCAAC---
TACTTGGTTGAGACCAAACGAAAGACTTGATACTGACCAGTCTTTGATAGGCAAACCATTTCTG
GCGAGCACGGTCTCGACAGCAATGGAGTGTACGTACCCTTTCCTTGGCTCCTTGCTTTCCCATGA
GCATGTTGACTAACACTCG-
TGGTCGTTCAGCTACAACGGTACCTCTGAGCTCCAGCTCGAGCGCATGAGCGTCTACTTCAACG
AGGCTTCCGGCAACAAGTACGTTCCTCGTGCCGTCCTCGTCGATCTCGAGCCCGGTACCATGGA
TGCCGTCCGCGCCGGTCCTTTCGGTCAGCTCTTCCGCCCTGACAACTTCGTCTTCG????????????
??
>P_hollandica_CBS_265_33
AGGGATCATTATAGAGTTTTCTAAACTCCCAACCCATGTGAACTTACC-
ATTGTTGCCTCGGCAGAAGCTACCTGGT--
TACCCTACCTTGGAACGGCCTACCCTGTAGCGCCTTACCCTGGAACGGCCTACCCTGTAACGGC
TGCCGGTGGACTACCAAACTCTTGTTATTTTATTGTAATCTGAGCGTCTTATTTTAATAAGTCAAA
ACTTTCAACAACGGATCTCTTGGTTCTGGCATCGATGAAGAACGCAGCGAAATGCGATAAGTAA
TGTGAATTGCAGAATTCAGTGAATCATCGAATCTTTGAACGCACATTGCGCCCATTAGTATTCTA
GTGGGCATGCCTGTTCGAGCGTCATTTCAACCCTTAAGCCTAGCTTAGTGTTGGGAGCCTACCG-
-CTTTTGCTAGCGGTAGCTCCTGAAATACAACGGCGGATCTGCGATATCCTCTGAGCGTAGTAA-

TTTTTATCTCGCTTTTGACTGGAGTTGCAGCGTCTTTAGCCGCTAAACCCCCCAAATTTTTTAATG
GTTGACCTCAGGTTAGTCATCCACACAATCCC-ATCATTC------
CCACCATTCCCATCTTATCATCATCGCCT----CAAA----CA-TCTTCCAACC-
GGTGCCGAAAATCT-G--------TTTTCGCACCTGCC-CATTTTCCCAG--ACACTTACCCC-----
---GCCGCACGACCCCGCGGTGCAAACGAAAAATTTCTTA--
TCACAGCCCCACATCGCACAAACATTTTGGCAGCCATGCACTTTCCAAGACCCACAATGAACAA
TTGCTGACCCCGCCAAATAGGAAGCCGCCGAGCTCGGAAAGGGTTCCTTCAAGTACGCATGGG
TTCTTGACAAGCTCAAGGCCGAGCGTGAGCGTGGTATCACCATTGATATCGCTCTCTGGAAGTT
CGAGACCAACGAGTACAATGTCACCGTCATTGGTTAGTATCCCTGCCCACACCATGTATCATGC
ATCCGACCTT-
GTGCTAACATGGCAACACAGATGCTCCC????????????????????TGCTGCCTTTTGGTATGTAGCC
-CATCTACCTCGACAC-GCCTCAATACGACAACCCCCCGCAACTCGACAAC--
GACGTTCTCAACAACTGCTTGGTTGGAAACAAAGGAAAGACTTGATACTGACCGGTCCTTGATA
GGCAAACCATCTCTGGCGAGCACGGTCTCGACAGCAATGGAGTGTACGTACCCTTTCCTTGGCT
ACTTGCTTTCCCACGAACATCTCAGCTAACACTCG-
TGGTTGTTCAGCTACAACGGTACCTCCGAGCTCCAGCTCGAGCGCATGAGCGTCTACTTCAACG
AGGCTTCCGGCAACAAGTACGTTCCTCGTGCCGTCCTCGTCGATCTCGAGCCCGGTACCATGGA
TGCCGTCCGCGCCGGTCCTTTCGGTCAGCTCTTCCGCCCTGACAACTTCGTCTTCGGTCAGTCCG
GTGCC
>P_hollandica_MEAN_1091
????ATCATTATAGAGTTTTCTAAACTCCCAACCCATGTGAACTTACC-
ATTGTTGCCTCGGCAGAAGCTACCTGGT--
TACCCTACCTTGGAACGGCCTACCCTGTAGCGCCTTACCCTGGAACGGCCTACCCTGTAACGGC
TGCCGGTGGACTACCAAACTCTTGTTATTTTATTGTAATCTGAGCGTCTTATTTTAATAAGTCAAA
ACTTTCAACAACGGATCTCTTGGTTCTGGCATCGATGAAGAACGCAGCGAAATGCGATAAGTAA
TGTGAATTGCAGAATTCAGTGAATCATCGAATCTTTGAACGCACATTGCGCCCATTAGTATTCTA
GTGGGCATGCCTGTTCGAGCGTCATTTCAACCCTTAAGCCTAGCTTAGTGTTGGGAGCCTACCG-
-CTTTTGCTAGCGGTAGCTCCTGAAATACAACGGCGGATCTGCGATATCCTCTGAGCGTAGTAA-
TTTTTATCTCGCTTTTGACTGGAGTTGCAGCGTCTTTAGCCGCTAAACCCCCCAAATTTTTTAATG
GTTGACCTC???????????CCACACAATCCC-ATCATTC------
CCACCATTCCCATCTTATCATCATCGCCT----CAAA----CA-TCTTCCAACC-
GGTGCCGAAAATCT-G--------TTTTCGCACCTGCC-CATTTTCCCAG--ACACTTACCCC-----
---GCCGCACGACCCCGCGGTGCAAACGAAAAATTTCTTA--
TCACAGCCCCACATCGCACAAACATTTTGGCAGCCATGCACTTTCCAAGACCCACAATGAACAA
TTGCTGACCCCGCCAAATAGGAAGCCGCCGAGCTCGGAAAGGGTTCCTTCAAGTACGCATGGG
TTCTTGACAAGCTCAAGGCCGAGCGTGAGCGTGGTATCACCATTGATATCGCTCTCTGGAAGTT
CGAGACCAACGAGTACAATGTCACCGTCATTGGTTAGTATCCCTGCCCACACCATGTATCATGC
ATCCGACCTT-
GTGCTAACATGGCAACACAGATGCTCCCGGTCACCGTGACTTCATCAATGCTGCCTTTTGGTATG
TAGCC-CATCTACCTCGACAC-GCCTCAATACGACAACCCCCCGCAACTCGACAAC--
GACGTTCTCAACAACTGCTTGGTTGGAAACAAAGGAAAGACTTGATACTGACCGGTCCTTGATA
GGCAAACCATCTCTGGCGAGCACGGTCTCGACAGCAATGGAGTGTACGTACCCTTTCCTTGGCT
ACTTGCTTTCCCACGAACATCTCAGCTAACACTCG-
TGGTTGTTCAGCTACAACGGTACCTCCGAGCTCCAGCTCGAGCGCATGAGCGTCTACTTCAACG

AGGCTTCCGGCAACAAGTACGTTCCTCGTGCCGTCCTCGTCGATCTCGAGCCCGGTACCATGGA
TGCCGTCCGCGCCGGTCCTTTCGGTCAGCTCTTCCG??????????????????????????????????
>P_humus_CBS_336_97
AGGGATCATTATAGAGTTTTCTAAACTCCCAACCCATGTGAACTTACC-
ATTGTTGCCTCGGCAGAAGCTGCTCGGC-
GCGCCTTACCTTGGAACGGCCTACCCTGTAGCGCCTTACCCTGGAACGGCTTACCCTGCAACGG
CTGCCGGTGGACTACCAAACTCTTGTTATTTTATGGTTATCTGAGCGTCTTATTTTAATAAGTCAA
AACTTTCAACAACGGATCTCTTGGTTCTGGCATCGATGAAGAACGCAGCGAAATGCGATAAGTA
ATGTGAATTGCAGAATTCAGTGAATCATCGAATCTTTGAACGCACATTGCGCCCATTAGTATTCT
AGTGGGCATGCCTGTTCGAGCGTCATTTCAACCCTTAAGCCTAGCTTAGTGTTGGGAGCCTACT
G--
CTTTTGCTAGCTGTAGCTCCTGAAATACAACGGCGGATCTGCGATATCCTCTGAGCGTAGTAA-
TTTTTATCTCGCTTTTGACTGGAGTTGCAGCGTCTTTAGCCGCTAAACCCCCC-AA-
TTTTTAATGGTTGACCTCAGGTTAGTCATGCTCACAGTCCC-ATCATCC------TCATC---------
--ACCATCATCGCCT--CGCAAA----CA-TTTTCCAACC-GGTGCCGAAATTCT-G--------
TTTTCGCACCTGCC-CATTTTCCCAG--ACAATTACCCC--------
GCCGCACGACCCCGCGGTGCAAACGAAAAATTTCTTA--TCACAGCCCCACTTCACAC-
AACATTTTGGCGGCCACGCACTTTGCATGACCCACAATGAACAATTGCTGACCCCGCCAAATAG
GAAGCCGCCGAGCTCGGAAAGGGTTCCTTCAAGTACGCATGGGTTCTTGACAAGCTCAAGGCC
GAGCGTGAGCGTGGTATCACCATTGATATCGCTCTCTGGAAGTTCGAGACCAACGAGTACAATG
TCACCGTCATTGGTTAGTATCCCTGTCCACAGAAAGTATCATGTGTCCGAACTC-
AGACTAACATCGCAATACAGACGCTCCCGGTCACCGTGATTTCATCAATGCTGCCTTCTGGTAT
GTAGTC-CATCTACCTCGACAC-GCCTCAATACGACA-CCCTCCGCAACTCGACGAC--
GGCATTCTCGGC---
TACTTGGTTGGAGCCAAACGAAAGACTTGATACTGACCGGTCTCTGATAGGCAAACCATCTCTG
GCGAGCACGGTCTCGACAGCAATGGAGTGTACGTACCGTTTCCTTGGCTACTTGCTTTCCCACG
AACATGTTAGCTAACACTCG-
TGCTTGCTCAGCTACAACGGCACCTCCGAGCTCCAGCTCGAGCGCATGAGCGTCTACTTCAACG
AGGCTTCCGGCAACAAGTACGTTCCTCGTGCTGTCCTCGTCGATCTCGAGCCCGGTACCATGGA
CGCCGTCCGCGCCGGTCCTTTCGGCCAGCTCTTCCGCCCTGACAACTTCGTCTTCGGTCAGTCC
GGTGCC
>P_hunanensis_CSUFTCC15
????????????????????????????????????TGTGAACTTACC-
ATTGTTGCCTCGGCAGAAGCTGCTCGGT-
GCACCTTACCCTGGAACGGCCTACCCTGTAGCGCCTTACCCTGGAACGGCCTACCCTGTAGCGG
CTGCCGGTGGACTACCAAACTCTTGTTATTGTATTGTAATCTGAGCGTCTTATTTTAATAAGTCAA
AACTTTCAACAACGGATCTCTTGGTTCTGGCATCGATGAAGAACGCAGCGAAATGCGATAAGTA
ATGTGAATTGCAGAATTCAGTGAATCATCGAATCTTTGAACGCACATTGCGCCCATTAGTATTCT
AGTGGGCATGCCTGTTCGAGCGTCATTTCAACCCTTAAGCCTAGCTTAGTGTTGGGAGCCTACT
G--
CTTTTACTAGCTGTAGCTCCTGAAATACAACGGCGGATCTGCGATATCCTCTGAGCGTAGTAAAT
TTTTATCTCGCTTTTGACTGGAGTTGCAGCGTCTTTAGCCGCTAAATCCCCC-AA-
TTTTTAATGGTTGACCTCAGGTTAGTCATCCTCGCAATCCC-ATCATCC------TCATT--------
CTCATCATCATCACCT--CGCAAA----CA-TTTCCACACC-GGTGCCGAAAATCTGG--------

TTTTCGCACCTGCC-CATTTTCCCAG--ACACTTACCCC--------
GCCGCACGACCCCGCGGTGCAAACGAAAAATTTCTTA--
TCACAGCCCCACATCACACAAACATTTTGGCAGCCACGCACTTTGCATGACCCACAATGAACAA
TTGCTGACCCCGCCAAATAGGAAGCCGCCGAGCTCGGAAAGGGTTCCTTCAAGTACGCATGGG
TTCTTGACAAGCTCAAGGCCGAGCGTGAGCGTGGTATCACCATTGATATCGCTCTCTGGAAGTT
CGAGACCAACGAGTACAATGTCACCGTCATTGGTTAGTATCCCTGCCCACAACATGTATCATGT
CTTCGAACTCAAGACTAACCTTGCAATACAGACGCTCCCGGTCACCGTGATTTCATCAATGCTG
CCTTCTGGTATGTAGCC-CATCTACCTCGACGC-GCCTCAATACGACA-
CCACCGGCAACTCGACAAC--GACATTCTCAAC---
TGCTTGGTTGAAACCAAAAGAAAGACCTGATACTGACCGGTCTTTGATAGGCAAACCATCTCTG
GCGAGCACGGTCTCGACAGCAATGGAGTGTACGTACCCTTTCCCTGGCTACTCGTTTTTCCACG
AACATGTCAGCTAACAGTCG-
TGCTTGTTCAGCTACAACGGTACCTCCGAGCTCCAGCTCGAGCGCATGAGCGTCTACTTCAACG
AGGCTTCCGGTAACAAGTACGTTCCTCGTGCCGTCCTCGTCGATCTCGAGCCCGGTACCATGGA
TGCCGTCCGCGCCGGCCCTTTCGGTCAGCTCTTCCGCCCTGACAACTTCGTCTTCGGCCAGTCC
GGTGCC
>P_hunanensis_CSUFTCC18
????????????????????????????????????TGTGAACTTACC-
ATTGTTGCCTCGGCAGAAGCTGCTCGGT-
GCACCTTACCCTGGAACGGCCTACCCTGTAGCGCCTTACCCTGGAACGGCCTACCCTGTAGCGG
CTGCCGGTGGACTACCAAACTCTTGTTATTGTATTGTAATCTGAGCGTCTTATTTTAATAAGTCAA
AACTTTCAACAACGGATCTCTTGGTTCTGGCATCGATGAAGAACGCAGCGAAATGCGATAAGTA
ATGTGAATTGCAGAATTCAGTGAATCATCGAATCTTTGAACGCACATTGCGCCCATTAGTATTCT
AGTGGGCATGCCTGTTCGAGCGTCATTTCAACCCTTAAGCCTAGCTTAGTGTTGGGAGCCTACT
G--
CTTTTACTAGCTGTAGCTCCTGAAATACAACGGCGGATCTGCGATATCCTCTGAGCGTAGTAAAT
TTTTATCTCGCTTTTGACTGGAGTTGCAGCGTCTTTAGCCGCTAAATCCCCC-AA-
TTTTTAATGGTTGACCTCAGGTTAGTCATCCTCGCAATCCC-ATCATCC------TCATT--------
CTCATCATCATCACCT--CGCAAA----CA-TTTCCACACC-GGTGCCGAAAATCTGG--------
TTTTCGCACCTGCC-CATTTTCCCAG--ACACTTACCCC--------
GCCGCACGACCCCGCGGTGCAAACGAAAAATTTCTTA--
TCACAGCCCCACATCACACAAACATTTTGGCAGCCACGCACTTTGCATGACCCACAATGAACAA
TTGCTGACCCCGCCAAATAGGAAGCCGCCGAGCTCGGAAAGGGTTCCTTCAAGTACGCATGGG
TTCTTGACAAGCTCAAGGCCGAGCGTGAGCGTGGTATCACCATTGATATCGCTCTCTGGAAGTT
CGAGACCAACGAGTACAATGTCACCGTCATTGGTTAGTATCCCTGCCCACAACATGTATCATGT
CTTCGAACTCAAGACTAACCTTGCAATACAGACGCTCCCGGTCACCGTGATTTCATCAATGCTG
CCTTCTGGTATGTAGCC-CATCTACCTCGACGC-GCCTCAATACGACA-
CCACCGGCAACTCGACAAC--GACATTCTCAAC---
TGCTTGGTTGAAACCAAAAGAAAGACCTGATACTGACCGGTCTTTGATAGGCAAACCATCTCTG
GCGAGCACGGTCTCGACAGCAATGGAGTGTACGTACCCTTTCCCTGGCTACTCGTTTTTCCACG
AACATGTCAGCTAACAGTCG-
TGCTTGTTCAGCTACAACGGTACCTCCGAGCTCCAGCTCGAGCGCATGAGCGTCTACTTCAACG
AGGCTTCCGGTAACAAGTACGTTCCTCGTGCCGTCCTCGTCGATCTCGAGCCCGGTACCATGGA
TGCCGTCCGCGCCGGCCCTTTCGGTCAGCTCTTCCGCCCTGACAACTTCGTCTTCGGCCAGTCC

GGTGCC
>P_hydei_MFLUCC_20_0135
??????CATTATAGAGTTTTCTAAACTCCCAACCCATGTGAACTTACC-
ATTGTTGCCTCGGCAGAAGCTGCTCGGT-
GCACCCTACCTTGGAACGGCCTACCCTGTAGCGCCTTACCCTGGAACGGCTTACCCTGTAACGG
CTGCCGGTGGACTACCAAACTCTTGTTATTTTATTGTAATCTGAGCGTCTTATTTTAATAAGTCAA
AACTTTCAACAACGGATCTCTTGGTTCTGGCATCAATGAAGAACGCAGCGAAATGCGATAAGTA
ATGTGAATTGCAAAATTCAGTGAATCATCGAATCTTTGAACGCACATTGCGCCCATTAGTATTCT
AGTGGGCATGCCTGTTCGAACGTCATTTCAACCCTTAAGCCTAGCTTAGTGTTGGGAGCCTACTG
--
CTTTTGCTAGCTGTAGCTCCTGAAATACAACGGCGGATCTGCGATATCCTCTGAGCGTAGTAAAT
TTTTATCTCGCTTTTGACTGGAGTTGCAGCGTCTTTGGCCGCTAAATCCCCC-AA-
TTTTT???????????????????GTCTTGCGCA---TTCC-TTCATCC------TCTCC---------
TCATCATCATCACTT--CGCAAA----CATTTCCCACACC-GGTGTCGAAAATCTGG--------
TTTTCGCACCTGCC-CATTTTCTCAG--ACACTTACCCC--------
GCCGCACGACCCCGCGGTGCAAACGAAAAATTTCTTA--
TCACAGCCCCACATCACACAAACATTTTGGCAGCCACGCACCTTGCATGACCCACAATGAACAA
TTGCTGACCCCGCCAAATAGGAAGCCGCCGAGCTCGGAAAGGGTTCCTTCAAGTACGCATGGG
TTCTTGACAAGCTCAAGGCCGAGCGTGAGCGTGGTATCACCATTGATATCGCTCTCTGGAAGTT
CGAGACCAACGAGTACAATGTCACCGTCATTGGTTAGTATCCCTGCCCACAATATGTGTCATGT
CTCTGAACTCAAGACTAACCTTGCAATACAGACGCTCCCGGTCACCGTGATTTCATCAA????????
?????????????C-CATCTACCTCGACGC-GCCTCAATACGACA-CCCCCGGCAACTCGACAAC--
GACATTCTCAAC---
TGCTTGTTTGGAACCATACGAAAGACTTCATACTGACCGGTCTATGATAGGCAAACCATCTCTGG
CGAGCACGGTCTCGACAGCAATGGAGTGTACGTACCCTTGATCTCGCTACTCGCTTTCTCGTGA
ACATGTCAGCTAACAGTCG-
TGCTTGTTCAGCTACAACGGTACCTCCGAGCTCCAGCTCGAGCGCATGAGCGTCTACTTCAACG
AGGCTTCCGGCAACAAGTACGTTCCTCGTGCCGTCCTCGTCGATCTCGAGCCCGGTACCATGGA
TGCCGTCCGCGCCGGTCCTTTCGGTCAGCTCTTCCGCCCTGACAACTTCGTCTTCGGTCAGTCCG
GTGCC
>P_iberica_CAA1006
???????????????GTTTTCTAAACTCCCAACCCATGTGAACTTACC-
ATTGTTGCCTCGGCAGAAGCTGCTCGGT-
GCACCTTACCCTGGAACGGCCTACCCTGTAGCGCC---------------
TTACCCTGTAGCGGCTGCCGGTGGACTACCAAACTCTTGTTATTTTATTGTAATCTGAGCGTCTTA
TTTTAATAAGTCAAAACTTTCAACAACGGATCTCTTGGTTCTGGCATCGATGAAGAACGCAGCGA
AATGCGATAAGTAATGTGAATTGCAGAATTCAGTGAATCATCGAATCTTTGAACGCACATTGCG
CCCATTAGTATTCTAGTGGGCATGCCTGTTCGAGCGTCATTTCAACCCTTAAGCCTAGCTTAGTG
TTGGGAGCCTACTG--
CTTTTGCTAGCGGTAGCTCCTGAAATACAACGGCGGATCTGCGATATCCTCTGAGCGTAGTAA-
TTTTTATCTCGCTTTTGACTGGAGTTGCAGCGTCTTTAGCCGCTAAACCCCCC-AA-
TTTTTAATGGTTGACCTCAGGTTAGTCATCCTCACAATCCC-ATCATCC-------------------
CCATCATCATCGTCT--CGCAAA----CA-TTTTCCAACT-GGTGCCGAAAATCT-G--------
TTTTCGCACCTGCC-CATTTTCCCAG--ACACTTACCCC--------

GCCGCACGACCCCGCGGTGCAAACGAAAAATTTCTTA--
TCACAGCCCCACATCGCACAAACATTTTGGCAGCCATGCACTTTCCATGACCCACAATGAACAA
TTGCTGACCCCGCCAAATAGGAAGCCGCCGAGCTCGGAAAGGGTTCCTTCAAGTACGCATGGG
TTCTTGACAAGCTCAAGGCCGAGCGTGAGCGTGGTATCACCATTGATATCGCTCTCTGGAAGTT
CGAGACCAACGAGTACAATGTCACCGTCATTGGTTAGTATCCCGGTCCACACCATGCACCATGC
ATCCGACCTT-
GTACTAACATCACAATACAGATGCTCCCGGTCACCGTGATTTCATCAATGCTGCCTTCTGGTACG
TAGTC-AATCTACCTCGACAC-GCCTCAATACGACAACCCTCCGCAATTCGACAAC--
GACCTTCTCAACAACTGCTTGGTTGGAACCAAAGAAAAGACCTGATACTGACCGGTCTCTGATA
GGCAAACCATCTCTGGCGAGCACGGTCTCGACAGCAATGGAGTGTACGTACCCTTTCCTTGGCT
ACTTGCTTTCCCACGAACATCTCAGCTAATACCCG-
TGGTTGTTCAGCTACAACGGTACCTCTGAGCTCCAGCTCGAGCGCATGAGCGTCTACTTCAACG
AGGCTTCCGGCAACAAGTACGTTCCTCGTGCCGTCCTCGTCGATCTCGAGCCCGGCACCATGGA
TGCCGTCCGCGCCGGTCCTTTCGGTCAGCTCTTCCGCCCTGACAACTTTGTCTTCGGTCAGTCCG
GTGCC
>P_inflexa_MFLUCC_12_0270
??????CATTATAGAGTTTTCTAAACTCCCAACCCATGTGAACTTACC-
ATTGTTGCCTCGGCAGAAGCTGCTCGGT-
GCACCTTACCTTGGAACGGCCTACCCTGTAGCGCCTTACCCTGGAACGGCTTACCCTGTAACGG
CTGCCGGTGGACTACCAAACTCTTGTTATTTTATTGTAATCTGAGCGTCTTAT-
TTAATAAGTCAAAACTTTCAACAACGGATCTCTTGGTTCTGGCATCGATGAAGAACGCAGCGAA
ATGCGATAAGTAATGTGAATTGCAGAATTCAGTGAATCATCGAATCTTTGAACGCACATTGCGC
CCATTAGTATTCTAGTGGGCATGCCTGTTCGAGCGTCATTTCAACCCTTAAGCCTAGCTTAGCGT
TGGGAGCCTACTG--
CTTTTATTAGCTGTAGCTCCTGAAATACAACGGCGGATCTGCGATATCCTCTGAGCGTAGTAA-
TTTTTATCTCGCTTTTGACTGGAGTTGCAGCGTCTTTAGCCGCTAAATCCCCC-AA-
TTTTTAATGGTTGACCTCAGGTTAGTCATCCTCACAATCCC-ATCATCC------CCATC--------
TTCATCATCATCACCT--GGCAAA----TACTTTTCCCAAC-GGTGCCGAAAATCT-G--------
TTTTCGCACCTGCC-CACTTTCCCAG--ACACGTACCCC--------
GCCGCACGACCCCGCGGTGCAAACGAAAAAATTCTTA--
TCACAGCCCCACATCGCACAACCATTTTGGCAGCCATGCACTTTTCATGACCCACAACGACCAT
TCGCTGACCCCGCCAAACAGGAAGCCGCCGAGCTCGGAAAGGGTTCCTTCAAGTACGCATGGG
TTCTTGACAAGCTCAAGGCCGAGCGCGAGCGTGGTATCACCATTGATATCGCTCTCTGGAAGTT
CGAGACCAACCAGTACAATGTCACCGTCATTGGTTAGTATCCCTGTCCACAACATGCATCATGC
ATCCGAACTC-
ATACTAACATGGCAATACAGATGCTCCCGGTCACCGTGACTTCATCAATGCTGCTTTCTGGTATG
TAGCC-CA-CCACCTCGACAC-GGCTCAGCACGACG-CCTCCCGCAACTCCACGAC--
GACGTTTTCAAC---
TACTTGGTTGAAACCAAACGAAAGACTCGATACTGACCGGTCTCTGATAGGCAAACCATCTCTG
GCGAGCACGGTCTCGACAGCAATGGAGTGTACGTACCCTTTCCTTGGCTACTTGCTTTCCCACG
AACATGTCAGCTAACACTCG-
TGCTTGCTCAGCTACAACGGTACCTCCGAGCTCCAGCTCGAGCGCATGAGCGTCTACTTCAACG
AGGCTTCCGGTAACAAGTACGTTCCTCGTGCCGTCCTCGTCGATCTCGAGCCCGGTACCATGGA
TGCCGTCCGCGCCGGTCCTTTCGGTCAGCTCTTCCGCCCTGACAACTTCGTCTTCGGTCAGTCCG

GTGCC
>P_intermedia_MFLUCC_12_0259
??????CATTATAGAGTTTTTTAAC-TCCCAACCCATGTGAACTTACC-
ATTGTTGCCTCGGCAGAAGCTACCTGGT--
TACCTTACCTTGGAACGGCCTACCCTGTAGCGCCTTACCCTGGAACGGCCTACCCTGTAACGGC
TGCCGGTGGACTACCAAACTCTTGTTATTTTATTGTAATCTGAGCGTCTTATTTTAATAAGTCAAA
ACTTTCAACAACGGATCTCTTGGTTCTGGCATCGATGAAGAACGCAGCGAAATGCGATAAGTAA
TGTGAATTGCAGAATTCAGTGAATCATCGAATCTTTGAACGCACATTGCGCCCATTAGTATTCTA
GTGGGCATGCCTGTTCGAGCGTCATTTCAACCCTTAAGCCTAGCTTAGTGTTGGGAGCCTACTG-
-CTTTTGCTAGCGGTAGCTCCTGAAATACAACGGCGGATCTGCGATATCCTCTGAGCGTAGTAA-
TTTTTATCTCGCTTTTGACTGGAGTTGCAGCGTCTTTAGCCGCTAAACCCCCC-AA-
TTTTTAATGGTTGACCTCAGGTTAGTCATCCTCAAA-TCCC-ATCATTC------CCATC--------
CTCATCATCATCGCCT--CGCAAA----CA-TTTTCCAACC-GGTGCCGAGAATCT-G--------
TTTTCGCATCTGCC-CATTTTCCCAG--ACACTTACCCC--------
GCCGCACGACCCCGCGGTGCAAACGAAAAATTTCTTA--
TCACAGCCCCACATCGCACAAACATTTTGGCAGCCATGCACTTTCCAAGACCCACAATGAACAT
TTGCTGACCCCGCCAAATAGGAAGCCGCCGAGCTCGGAAAGGGTTCCTTCAAGTACGCATGGG
TTCTTGACAAGCTCAAGGCCGAGCGTGAGCGTGGTATCACCATCGATATCGCTCTCTGGAAGTT
CGAGACCAACGAGTACAATGTCACCGTCATTGGTTAGTATTCCTGTCCACACGATGTACTATGC
ATCTGAATGT-
ATACTAACATGGCAACACAGATGCTCCCGGTCACCGTGATTTCATCAATGCTGCTTTCTGGTATG
TTACC-TGTCTGCCTCGACACGGCCTTAATACGACAACCCCCCGCAACTCGACAAC--
GACGTTCTCAACAAGTGCTTGGTTGGAAACAAGGGAAAGACTTGATACTGACCGGTCCCTGATA
GGCAAACCATCTCTGGCGAACACGGTCTCGACAGCAATGGAGTGTACGTACCCTTTCCTTAGCT
ACTTGCTTTCCCACGAACATCTTAGCTAACACTCG-
TGGTTGTTCAGCTACAACGGTACCTCCGAGCTCCAGCTCGAGCGCATGAGCGTCTACTTCAACG
AGGCATCCGGCAACAAGTACGTTCCTCGTGCCGTCCTCGTCGATCTCGAGCCCGGTACCATGGA
TGCCGTCCGCGCCGGTCCTTTCGGTCAGCTCTTCCGCCCTGACAACTTCGTCTTCGGTCAGTCCG
GTGCC
>P_italiana_MFLUCC_12_0657
???
?????????????????????????????????????CCTACCCTGTAACGGTTGCCGGTGGACTACCAAACTCT
TGTTATTTTATTGTAATCTGAGCGTCTTATTTTAATAAGTCAAAACTTTCAACAACGGATCTCTTGG
TTCTGGCATCGATGAAGAACGCACCGAAATGCGACAAGTAATGTGAATTGCAGAATTCAGTGAA
TCATCGAATCTTTGAACGCACATTGCGCCCATTAGTATTCTAGTGGGCATGCCTGTTCGAGCGTC
TTTTCCACCCTTAAGCCTAGCTTAGTGTTGGGAGCCTACCG--
CTTTTGCTATCGGTATCTCCTGAAATACAACGGCGGATCTGCGATATCCTCTGATCGTAGTAA-
TTTTTATCTCGCTTTTGACTGGAGTTGCAGCGTCTTTAGCCGCTAAACCCCCCTAATTTTTTAATG
GTTGACCTC????TAGTCATCCACACAATCCC-ATCATTC-------
CCCCATTCCCATCTTATCATCATCGCCT----CAAA----CA-TCTTCCAACC-
GGTGCCGAAAATCT-G--------TTTTCGCACCTGCC-CATTTTCCCAG--ACACTTACCCC-----
---GCCGCACGACCCCGCGGTGCAAACGAAAAATTTCTTA--
TCACAGCCCCACATCGCACAAACATTTTGGCAGCCATGCACTTTCCAAGACCCACAATGAACAA
TTGCTGACCCCGCCAAATAGGAAGCCGCCGAGCTCGGAAAGGGTTCCTTCAAGTACGCATGGG

TTCTTGACAAGCTCAAGGCCGAGCGTGAGCGTGGTATCACCATTGATATCGCTCTCTGGAAGTT
CGAGACCAATGAGTACAATGTCACCGTCATTGGTTAGTATCCCTGCCCACACCATGTATCATGC
ATCCGACCTT-
GTGCTAACATGGCAACACAGATGCTCCCGGTCACCGTGACTTCATCAATGCTGCCTTTTGGTATG
TAGCC-CATCTACCTCGACAC-GCCTCAATACGACACCCCCCGGCAACTCGACAAC--
GACGTTCTCAACAACTGCTTGGTTGGAAACAAAGGAAAGACTTGATACTGACCGGTCCTTGATA
GGCAAACCATCTCTGGCGAGCACGGTCTCGACAGCAATGGAGTGTACGTACCCTTTCCTTGGCT
ACTTGCTTTCCCACGAACATCTCAGCTAACACTCG-
TGGTTGTTCAGCTACAACGGTACCTCCGAGCTCCAGCTCGAGCGCATGAGCGTCTACTTCAACG
AGGCTTCCGGCAACAAGTACGTTCCTCGTGCCGTCCTCGTCGATCTCGAGCCCGGTACCATGGA
TGCCGTCCGCGCCGGTCCTTTCGGTCAGCTCTTCCGCCCTGACAACTTCGTCTTCGGTCAGTCCG
GTGCC
>P_jesteri_CBS_109350
??????CATTATAGAGTTTTTCAAACTCCCAACCCATGTGAACTTACC-
ACTGTTGCCTCGGCAGAGGCTACCCGGT----
ACCTACCTCGGAACGGCCTACCCTGTAGCGCCTTACCTGGGAGCGG-
TTACCCTGTAGCGGCTGCCGGTGGACTACCAAACTCTTGTTATTTTATTGTAATCTGAGCGTCTTA
TTTTAATAAGTCAAAACTTTCAACAACGGATCTCTTGGTTCTGGCATCGATGAAGAACGCAGCGA
AATGCGATAAGTAATGTGAATTGCAGAATTCAGTGAATCATCGAATCTTTGAACGCACATTGCG
CCCATTAGTATTCTAGTGGGCATGCCTGTTCGAGCGTCATTTCAACCCTTAAGCCTAGCTTAGTG
TTGGGAGCCTACTAG-
CCCTCGCGGCCTGTAGCTCCTGAAATACAACGGCGGATCTGCGATATCCTCTGAGCGTAGTAAT
CTTACCTCTCGCTTTTGTCAGGATTCCCAGCATCT--AGCCGCTAAACCCCCC-AA-
TTTTTAATGGTTGACCTC??
??
??
??
??
??
TGCTGCCTTCTGGTACGTACCC-CGACTGCCTCGACAC-GCCTCATCACGACG-
CCTCTGAGTACCCTAC-----GACGATTCGAAC----
AACTAGTGGCAGCAACATGGGAGGCATGATACTGACTGATCTTTGACAGGCAAACTATCTCTGG
TGAGCACGGTCTCGACAGCAATGGAGTGTACGTACCATTTCCTTGGCCACTCGCTTTCTGAAGA
ACATGCAAGCTAAATTTCG-
TGGTCGATTAGTTACAACGGTACCTCCGAGCTCCAGCTCGAGCGTATGAGCGTCTACTTCAACG
AGGCTTCCGGCAACAAGTACGTTCCCCGTGCCGTCCTCGTCGATCTCGAGCCCGGTACCATGGA
TGCCGTCCGCGCTGGTCCCTTCGGTCAGCTCTTCCGCCCTGACAACTTTGTTTTCGGTCAATCCG
GTGCT
>P_jiangxiensis_LC4399
????????????????????????????????ACCCATGTGAACTTACC-
ATTGTTGCCTCGGCAGAAGCTACCTGGT--
TACCTTACCTTGGAACGGCCTACCCTGTAGCGCCTTACCCTGGAACGGCCTACCCTGTAACGGC
TGCCGGTGGACTACCAAACTCTTGTTATTTTATTGTAATCTGAGCGTCTTATTTTAATAAGTCAAA
ACTTTCAACAACGGATCTCTTGGTTCTGGCATCGATGAAGAACGCAGCGAAATGCGATAAGTAA

TGTGAATTGCAGAATTCAGTGAATCATCGAATCTTTGAACGCACATTGCGCCCATTAGTATTCTA
GTGGGCATGCCTGTTCGAGCGTCATTTCAACCCTTAAGCCTAGCTTAGTGTTGGGAGCCTACTG-
-CTTTTGCTAGCGGTAGCTCCTGAAATACAACGGCGGATCTGCGATATCCTCTGAGCGTAGTAA-
TTTTTATCTCGCTTTTGACTGGAGTTGCAGCGTCTTTAGCCGCTAAACCCCCC-AA-
TTTTTAATGGTTGACCTCAGGTTAGTTATCCTCACAATCCC-ATCATTC------CCATC---------
--ATCATCGTCGCCT--CGCGAA----CA-TTTCCCAACC-GGTGCCGAGAATCT-G--------
TTTTCGCACCTGCC-CATTTTCCCAG--ACACTTACCCC--------
GCCGCACGACCCCGCGGTGCAAACGAAAAATTTCTTA--
TCACAGCCCCACATCGCACAAACATTTTGGCAGCCATGCACCTTCCAAGACCCACAATGAACAA
TTGCTGACCCCGCCAAATAGGAAGCCGCCGAGCTCGGAAAGGGTTCCTTCAAGTACGCATGGG
TTCTTGACAAGCTCAAGGCCGAGCGTGAGCGTGGTATCACCATCGATATCGCTCTCTGGAAGTT
CGAGACCAACGAGTACAATGTCACCGTCATTGGTTAGTAACCTTGTCCATACGATGTACCATGC
ATCTGAATGT-
ATACTAACATGGCAAAACAGATGCTCCCGGTCACCGTGATTTCATCAATGCTGCCTTTTGGTATG
TAGCC-CATCTACCTCGACAC-GCCTCAATACGACAACCCCCCGCAACTCGACAAC--
GACGTTCTCAACAAGTGCTTGGTTGGAAACAAGGGAAAGACTTGATACTGACCGGTCCCTGATA
GGCAAACCATCTCTGGCGAGCACGGTCTCGACAGCAATGGAGTGTACGTACCCTTTCCTTGGCT
ACTTGCTTTCCCACGAACATCTCAGCTAACACTCG-
TGGTTTTTCAGCTACAACGGTACCTCCGAGCTCCAGCTCGAGCGCATGAGCGTCTACTTCAACG
AGGCTTCCGGCAACAAGTACGTTCCTCGTGCCGTCCTCGTCGATCTCGAGCCCGGTACCATGGA
TGCCGTCCGCGCCGGTCCTTTCGGTCAGCTCTTCCGCCCTGACAACTTCGTCTTCGGTCAGTCCG
GT???
>P_jinchanghensis_LC6636
????????????????????????????????????TGTGAACTTACC-
ATTGTTGCCTCGGCAGAAGCTGCTCGGT-
ATACCCTACCTTGGAACGGCCTACCCTGTAGCGCCTTACCCTGGAACGGCTTACCCTGCAACGG
CTGCCGGTGGACTACTAAACTCTTGTTATTTTATTGTAATCTGAGCGTCTTATTTTAATAAGTCAA
AACTTTCAACAACGGATCTCTTGGTTCTGGCATCGATGAAGAACGCAGCGAAATGCGATAAGTA
ATGTGAATTGCAGAATTCAGTGAATCATCGAATCTTTGAACGCACATTGCGCCCATTAGTATTCT
AGTGGGCATGCCTGTTCGAGCGTCATTTCAACCCTTAAGCCTAGCTTAGTGTTGGGAGCCTACT
G--
CTTTTGCTAGCTGTAGCTCCTGAAATACAACGGCGGATCTGCGATATCCTCTGAGCGTAGTAA-
TTTTTATCTCGCTTTTGACTGGAGTTGCAGCGTCTTTAGCCGCTAAACCCCCC-AA-
TTTTTAATGGTTGACCTCAGGTTAGTCATCCTCGCAATCCC-ATCATCC--------------------
CCATTCTCAT-ACCT--CGCAAA----CG-TTTTCCAACC-GGTGCCGAAAATCT-G--------
TTTTCGCACCTGCC-CATTTTCCCAG--ACACTTACCCC--------
GCCGCACGACCCCGCGGTGCAAACGAAAAATTTCTTA--TCACAGCCCCACTTCACAC-
AACATTTTGGCAGCCACGCACTTTGCATGACCCACAGTGAACAATTGCTAACCCCGCCAAATAG
GAAGCCGCCGAGCTCGGAAAGGGTTCCTTCAAGTACGCATGGGTTCTTGACAAGCTCAAGGCC
GAGCGTGAGCGTGGTATCACCATTGATATCGCTCTCTGGAAGTTCGAGACCAACGAGTACAATG
TCACCGTCATTGGTTAGTATCCCTGTTCACAGAATGTACCATGTCTCCGAACTC-
AGACTAACATCACAACACAGACGCTCCCGGTCACCGTGATTTCATCAATGCTGCCTTCTGGTAT
GTAGCC-CATCTACCTCGGCAC-GCCTCAATACGACA-CCCTCCGCAACTCGACGAC--
GACATTCTCGGC---

TACTTGGTTGGAACCGAACGAAAGACTTGATACTGACCGGTCTCTGATAGGCAAACCATCTCTG
GCGAGCACGGTCTCGACAGCAATGGAGTGTACGTACCATTTCCTTGCCTACTTGCTTTCCCACG
AACATGTTAGCTAACACTCG-
TGCTTGCTCAGCTACAACGGCACCTCCGAGCTCCAGCTCGAGCGCATGAGCGTCTACTTCAACG
AGGCGTCCGGCAACAAGTACGTTCCTCGTGCCGTCCTCGTCGATCTCGAGCCCGGTACCATGGA
CGCCGTCCGCGCCGGTCCTTTCGGCCAGCTCTTCCGCCCTGACAACTTCGTCTTCGGTCAGTCC
GGTGCC
>P_kaki_KNU_PT_1804
AGGGATCATTATAGAGTTTTCTAAACTCCCAACCCATGTGAACTTACC-
ATTGTTGCCTCGGCAGAAGCTGCTCGGT-
GCACCCTACCTTGGAACGGCCTACCCTGTAGCGCCTTACCCTGGAACGGCTTACCCTGTAACGG
CTGCCGGTGGACTACCAAACTCTTGTTATTTTATTGTAATCTGAGCGTCTTATTTTAATAAGTCAA
AACTTTCAACAACGGATCTCTTGGTTCTGGCATCGATGAAGAACGCAGCGAAATGCGATAAGTA
ATGTGAATTGCAGAATTCAGTGAATCATCGAATCTTTGAACGCACATTGCGCCCATTAGTATTCT
AGTGGGCATGCCTGTTCGAGCGTCATTTCAACCCTTAAGCCTAGCTTAGTGTTGGGAGCCTACT
G--
CTTTTGCTAGCTGTAGCTCCTGAAATACAACGGCGGATCTGCGATATCCTCTGAGCGTAGTAAAT
TTTTATCTCGCTTTTGACTGGAGTTGCAGCGTCTTTGGCCGCTAAATCCCCC-AA-
TTTTTAATGGTTGACCTCAGGTTAGTCATCCTCGCAATCCC-ATCATCC------TCATC--------
CTCATCATCATCACTT--CGCAAA----CATTTCCCACACC-GGTGTCGAAAATCTGG--------
TTTTCGCACCTGCC-CATTTTCTCAG--ACACTTACCCC--------
GCCGCACGACCCCGCGGTGCAAACGAAAAATTTCTTA--
TCACAGCCCCACATCACACAAACATTTTGGCAGCCACGCACCTTGCATGACCCACAATGAACAA
TTGCTGACCCCGCCAAATAGGAAGCCGCCGAGCTCGGAAAGGGTTCCTTCAAGTACGCATGGG
TTCTTGACAAGCTCAAGGCCGAGCGTGAGCGTGGTATCACCATTGATATCGCTCTCTGGAAGTT
CGAGACCAACGAGTACAATGTCACCGTCATTGGTTAGTATCCCTGCCCACAATATGTGTCATGT
CTCTGAACTCAAGACTAACCTTGCAATACAGACGCTCCCGGTCACCGTGATTTCATCAA????????
??????ATGTAGCC-CATCTACCTCGACGC-GCCTCAATACGACACCCCCCGGCAACTCGACAAC-
-GACATTCTTAAC---
TGCTTGTTTGGAACCATACAAAAGACTTGATACTGACCGGTCTATGATAGGCAAACCATCTCTG
GCGAGCACGGTCTCGACAGCAATGGAGTGTATGTACCCTTTCCCTGGCTACTCGCTTTCTCGTG
AACATGTCAGCTAACAGTCG-
TGCTTGTTCAGCTACAACGGTACCTCCGAGCTCCAGCTCGAGCGCATGAGCGTCTACTTCAACG
AGGCTTCCGGCAACAAGTACGTTCCTCGTGCCGTCCTCGTCGATCTCGAGCCCGGTACCATGGA
TGCCGTCCGCGCCGGTCCTTTCGGTCAGCTCTTCCGCCCTGACAACTTCGTCTTCGGTCAGTCCG
GTGCC
>P_kandelicola_NCYU_19_0355
AGGGATCATTATAGAGTTTTCTAAACTCCCAACCCATGTGAACTTACC-
ATTGTTGCCTCGGCAGAAGCTGCTCGGT-
GCACCTTACCTTGGAACGGCCTACCCTGTAGCGCCTTACCCTGGAACGGCTTACCCTGTAGCGG
CTGCCGGTGGACTACCAAACTCTTGTTATTTTATTGTAATCTGAGCGTCTTATTTTAATAAGTCAA
AACTTTCAACAACGGATCTCTTGGTTCTGGCATCGATGAAGAACGCAGCGAAATGCGATAAGTA
ATGTGAATTGCAGAATTCAGTGAATCATCGAATCTTTGAACGCACATTGCGCCCATTAGTATTCT
AGTGGGCATGCCTGTTCGAGCGTCATTTCAACCCTTAAGCCTAGCTTAGTGTTGGGAGCCTACT

G--
CTTTTACTAGCTGTAGCTCCTGAAATACAACGGCGGATCTGCGATATCCTCTGAGCGTAGTAA-
TTTTTATCTCGCTTTTGACTGGAGTTGCAGCGTCTTTAGCCGCTAAACCCCCC-AA--
TTTTAATGGTTGACCTC???TTAGTCATCCTCGCAATCCC-ATCATCC------TCATC--------
TTCATCATCATCACCT--CGCAAA----GA-TTTCCACATC-GGTGCCGAAAATCT-G--------
TTTTCGCACCTGCC-CATTTTCCCAG--ACACATACCCC--------
GCCGCACGACCCCGCGGTGCAAACGAAAAATTTCTTA--TCATAGCCCCACATCAC--
AAACATTTTGGCAGCCACGCACTCTGCATGACCCACAATGAACAATTGCTGACCCCGCCAAATA
GGAAGCCGCCGAGCTCGGTAAGGG-
TCCTTCAAGTAAA??
??
???????????????????????????????TGCTGCCTTCTGGTATGTAGCC-CATCTACCTCGACGC-
GCCTCAATACGACA-CCCCCGGCAACTCGACAAC--GACATTCTCAAC---
TGCTTGGTTGAAACCATACGAAAGACTTGGTACTGACCGGTCTCTGATAGGCAAACCATCTCTG
GCGAGCACGGTCTCGACAGCAATGGAGTGTACGTACCCTTTCCC-
GGCTACTCGCTTTCTCGTGAACATATCAGCTAACACTCG-
TGCTTGTTCAGCTACAACGGTACCTCCGAGCTCCAGCTCGAGCGCATGAGCGTCTACTTCAACG
AGGCTTCCGGCAACAAGTACGTTCCTCGTGCCGTCCTCGTCGATCTCGAGCCCGGTACCATGGA
TGCCGTCCGCGCCGGTCCTTTCGGTCAGCTCTTCCGCCCTGACAACTTCGTCTTCGGTCAGTCCG
GTGCC
>P_kenyana_CBS_442_67
AGGGATCATTATAGAGTTTTCTAAACTCCCAACCCATGTGAACTTACC-
ATTGTTGCCTCGGCAGAAGCTGCTCGGT-
GCACCCTACCTTGGAACGGCCTACCCTGTAGCGCCTTACCCTGGAACGGCTTACCCTGTAACGG
CTGCCGGTGGACTACCAAACTCTTGTTATTTTATTGTAATCTGAGCGTCTTATTTTAATAAGTCAA
AACTTTCAACAACGGATCTCTTGGTTCTGGCATCGATGAAGAACGCAGCGAAATGCGATAAGTA
ATGTGAATTGCAGAATTCAGTGAATCATCGAATCTTTGAACGCACATTGCGCCCATTAGTATTCT
AGTGGGCATGCCTGTTCGAGCGTCATTTCAACCCTTAAGCCTAGCTTAGTGTTGGGAGCCTACT
G--
CTTTTGCTAGCTGTAGCTCCTGAAATACAACGGCGGATCTGCGATATCCTCTGAGCGTAGTAAAT
TTTTATCTCGCTTTTGACTGGAGTTGCAGCGTCTTTGGCCGCTAAATCCCCC-AA-
TTTTTAATGGTTGACCTCAGGTTAGTCATCCTCGCAATCCC-ATCATCC------TCATC--------
CTCATCATCATCACTT--CGCAAA----CATTTCCCACACC-GGTGTCGAAAATCTGG--------
TTTTCGCACCTGCC-CATTTTCTCAG--ACACTTACCCC--------
GCCGCACGACCCCGCGGTGCAAACGAAAAATTTCTTA--
TCACAGCCCCACATCACACAAACATTTTGGCAGCCACGCACCTTGCATGACCCACAATGAACAA
TTGCTGACCCCGCCAAATAGGAAGCCGCCGAGCTCGGAAAGGGTTCCTTCAAGTACGCATGGG
TTCTTGACAAGCTCAAGGCCGAGCGTGAGCGTGGTATCACCATTGATATCGCTCTCTGGAAGTT
CGAGACCAACGAGTACAATGTCACCGTCATTGGTTAGTATCCCTGCCCACAATATGTGTCATGT
CTCTGAACTCAAGACTAACCTTGCAATACAGACGCTCCCGGTCACCGTGATTTCATCAATGCTG
CCTTCTGGTATGTAGCC-CATCTACCTCGACGC-GCCTCAATACGACA-
CCCCCGGCAACTCGACCAC--GATAATCTCAAC---
TGCTTGGTTGGAACCATACGAAAGACTCGATACTGACCGGTCTATGATAGGCAAACCATCTCTG
GCGAGCACGGTCTCGACAGCAATGGAGTGTACGTACCCTTTCCCTGGCTGTTCGCTTTCTCGTG

AACATGTCAGCTAACAGTCG-
TGCTTGTTCAGCTACAACGGTACCTCCGAGCTCCAGCTCGAGCGCATGAGCGTCTACTTCAACG
AGGCTTCCGGCAACAAGTACGTTCCTCGTGCCGTCCTCGTCGATCTCGAGCCCGGTACCATGGA
TGCCGTCCGCGCCGGTCCTTTCGGTCAGCTCTTCCGCCCTGACAACTTCGTCTTCGGTCAGTCCG
GTGCC
>P_knightiae_CBS_111963
AGGGATCATTATAGAGTTTTCTAAACTCCCAACCCATGTGAACTTACC-
ATTGTTGCCTCGGCAGAAGCTGCTCGGT-
ACACCCTACCTTGGAACGGCCTACCCTGTAGCGCCTTACCCTGGAACGGCTTACCCTGTAGCGG
CTGCCGGTGGACTACCAAACTCTTGTTATTTTATTGTAATCTGAGCGTCTTATTTTAATAAGTCAA
AACTTTCAACAACGGATCTCTTGGTTCTGGCATCGATGAAGAACGCAGCGAAATGCGATAAGTA
ATGTGAATTGCAGAATTCAGTGAATCATCGAATCTTTGAACGCACATTGCGCCCATTAGTATTCT
AGTGGGCATGCCTGTTCGAGCGTCATTTCAACCCTTAAGCCTAGCTTAGTGTTGGGAGCCTACT
G--
CTTTTACTAGCTGTAGCTCCTGAAATACAACGGCGGATCTGCGATATCCTCTGAGCGTAGTAA-
TTTTTATCTCGCTTTTGACTGGAGTTGCAGCGTCTTTAGCCGCTAAATCCCCC-AA-
TTTTTAATGGTTGACCTCAGGTTAGTCATCCTCGCAATCCC-ATCATCG------TCATC--------
CTCATCATCATCACCT--CGCAAA----CA-TTTCCACACC-GGTGCCGAAAATCTGG--------
TTTTCGCACCTGCC-CATTTTCCCAC--ACACTTACCCC--------
GCCGCACGACCCCGCGGTGCAAACGAAAAATTTCTTA--
TCATAGCCCCACATCACACAAACATTTTGGCAGCCACGCACTTTGCAAGACCCACAATGAACAA
TTGCTGACCCCGCCAAATAGGAAGCCGCCGAGCTCGGAAAGGGTTCCTTCAAGTACGCATGGG
TTCTTGACAAGCTCAAGGCCGAGCGTGAGCGTGGTATCACCATTGATATCGCTCTCTGGAAGTT
CGAGACCAACGAGTACAATGTCACCGTCATTGGTTAGTATCCCTGTCCACAACATGCATCATGT
CTTTGAACTCAAGACTAACCTTGCACTACAGACGCTCCCGGTCACCGTGATTTCATCAATGCTGC
CTTCTGGTATGTAGCC-CATCTACCTCGACGC-
GCCTCGATACGACACCCCCCGGCAACTCGACAAC--GACATTCTCAAC---
TGCTTGTTTGGAATCATACGAAAGACTTGATACTGACCGGTCTATGATAGGCAAACCATCTCTGG
CGAGCACGGTCTCGACAGCAATGGAGTGTACGTACCCTTTCCTTGGCTACTTGCTTTCTCGTGAA
CATGTCAGCTAACACTCG-
TGCTTGTTCAGCTACAACGGTACCTCCGAGCTCCAGCTCGAGCGCATGAGCGTCTACTTCAACG
AGGCTTCCGGCAACAAGTACGTTCCTCGTGCCGTCCTCGTCGATCTCGAGCCCGGTACCATGGA
TGCCGTCCGCGCCGGTCCTTTCGGTCAGCTCTTCCGCCCTGACAACTTCGTATTCGGTCAGTCCG
GTGCC
>P_knightiae_CBS_114138
AGGGATCATTATAGAGTTTTCTAAACTCCCAACCCATGTGAACTTACC-
ATTGTTGCCTCGGCAGAAGCTGCTCGGT-
ACACCCTACCTTGGAACGGCCTACCCTGTAGCGCCTTACCCTGGAACGGCTTACCCTGTAGCGG
CTGCCGGTGGACTACCAAACTCTTGTTATTTTATTGTAATCTGAGCGTCTTATTTTAATAAGTCAA
AACTTTCAACAACGGATCTCTTGGTTCTGGCATCGATGAAGAACGCAGCGAAATGCGATAAGTA
ATGTGAATTGCAGAATTCAGTGAATCATCGAATCTTTGAACGCACATTGCGCCCATTAGTATTCT
AGTGGGCATGCCTGTTCGAGCGTCATTTCAACCCTTAAGCCTAGCTTAGTGTTGGGAGCCTACT
G--
CTTTTACTAGCTGTAGCTCCTGAAATACAACGGCGGATCTGCGATATCCTCTGAGCGTAGTAA-

TTTTTATCTCGCTTTTGACTGGAGTTGCAGCGTCTTTAGCCGCTAAATCCCCC-AA-
TTTTTAATGGTTGACCTCAGGTTAGTCATCCTCGCAATCCC-ATCATCG------TCATC--------
CTCATCATCATCACCT--CGCAAA----CA-TTTCCACACC-GGTGCCGAAAATCTGG--------
TTTTCGCACCTGCC-CATTTTCCCAC--ACACTTACCCC--------
GCCGCACGACCCCGCGGTGCAAACGAAAAATTTCTTA--
TCATAGCCCCACATCACACAAACATTTTGGCAGCCACGCACTTTGCAAGACCCACAATGAACAA
TTGCTGACCCCGCCAAATAGGAAGCCGCCGAGCTCGGAAAGGGTTCCTTCAAGTACGCATGGG
TTCTTGACAAGCTCAAGGCCGAGCGTGAGCGTGGTATCACCATTGATATCGCTCTCTGGAAGTT
CGAGACCAACGAGTACAATGTCACCGTCATTGGTTAGTATCCCTGTCCACAACATGCATCATGT
CTTTGAACTCAAGACTAACCTTGCACTACAGACGCTCCCGGTCACCGTGATTTCATCAATGCTGC
CTTCTGGTATGTAGCC-CATCTACCTCGACGC-
GCCTCGATACGACACCCCCCGGCAACTCGACAAC--GACATTCTCAAC---
TGCTTGTTTGGAATCATACGAAAGACTTGATACTGACCGGTCTATGATAGGCAAACCATCTCTGG
CGAGCACGGTCTCGACAGCAATGGAGTGTACGTACCCTTTCCTTGGCTACTTGCTTTCTCGTGAA
CATGTCAGCTAACACTCG-
TGCTTGTTCAGCTACAACGGTACCTCCGAGCTCCAGCTCGAGCGCATGAGCGTCTACTTCAACG
AGGCTTCCGGCAACAAGTACGTTCCTCGTGCCGTCCTCGTCGATCTCGAGCCCGGTACCATGGA
TGCCGTCCGCGCCGGTCCTTTCGGTCAGCTCTTCCGCCCTGACAACTTCGTATTCGGTCAGTCCG
GTGCC
>P_licualacola_HGUP4057
AGGGATCATTATAGAGTTTTCTAAACTCCCAACCCATGTGAACTTACC-
ATTGTTGCCTCGGCAGAAGCTGCTCGGT-
ATACCCTACCTTGGAACGGCCTACCCTGTAGCGCCTTACCCTGGAACGGCTTACCCTGCAACGG
CTGCCGGTGGACTACCAAACTCTTGTTATTTTATTGTAATCTGAGCGTCTTATTTTAATAAGTCAA
AACTTTCAACAACGGATCTCTTGGTTCTGGCATCGATGAAGAACGCAGCGAAATGCGATAAGTA
ATGTGAATTGCAGAATTCAGTGAATCATCGAATCTTTGAACGCACATTGCGCCCATTAGTATTCT
AGTGGGCATGCCTGTTCGAGCGTCATTTCAACCCTTAAGCCTAGCTTAGTGTTGGGAGCCTACT
G--
CTTTTGCTAGTTGTAGCTCCTGAAATACAACGGCGGATCTGCGATATCCTCTGAGCGTAGTAA-
TTTTTATCTCGCTTTTGACTGGAGTTGCAGCGTCTTTAGCCGCTAAACCCCCC-AA-
TTTTTAATGGTTGACCTCAGGTTAGTCATGCTCACA-TCCC-ATCATCC------TCATC---------
--ATCATCATCGCCT--CGCAAA----CA-TTTTCCAACC-GGTGCCGAAATTCT-G--------
TTTTCGCACCTGCC-CATTTTTCCAG--ACACTTACCCC--------
GCCGCACGACCCCGCGGTGCAAACGAAAAATTTCTTATCTCACAGCCCCACTTCACAC-
AACATTTTGGCAGCCACGCACTTTGCATGACCCACAATGAACAATTGCTGACCCCGCCAAATAG
GAAGCCGCCGAGCTCGGAAAGGGTTCCTTCAAGTACGCATGGGTTCTTGACAAGCTCAAGGCC
GAGCGTGAGCGTGGTATCACCATTGATATCGCTCTCTGGAAGTTCGAGACCAACGAGTACAATG
TCACCGTCATTGGTTAGTATCCCTGTCCATAGAAAGTATCATGTGTCCGAACTC-
AGACTAACATCGCAATACAGACGCTCCCGGTCACCGTGATTTCATCAA????????????GAGGATA
GTC--ATCTACCTCGACAC-GCCTC-ATACGACA-CCCTCCGCAACTCGACGAC--
GGCATTCTCGGC---
TACTTGGTTGGAACCAAACGAAAGACTTGATACTGACCGGTCTCTGATAGGCAAACCATCTCTG
GCGAGCACGGTCTCGACAGCAATGGAGTGTACGTACCGTTTCCTTGGCTACTTGCTTTCCCACG
AACATGTTAGCTAACACTCG-

TGCTTGCTCAGCTACAACGGCACCTCGGAGCTCCAGCTCGAGCGCATGAGCGTCTACTTCAACG
AGGCTTCCGGCAACAAGTACGTTCCTCGTGCTGTCCTCGTCGATCTCGAGCCTGGTACCATGGA
CGCCGTCCGCGCCGGTCCTTTCGGCCAGCTCTTCCGCCCTGACAACTTCGTCTTCGGTCAGTCC
GGTGCC
>P_linearis_MFLUCC_12_0271
??????CATTATAGAGTTTTTTAAACTCCCAACCCATGTGAACTTACC-
ATTGTTGCCTCGGCAGAAGCTACCTGGT--
TACCTTACCTTGGAACGGCCTACCCTGTAGCGCCTTACCCTGGAACGGCCTACCCTGTAACGGC
TGCCGGTGGACTACCAAACTCTTGTTATTTTATTGTAATCTGAGCGTCTTATTTTAATAAGTCAAA
ACTTTCAACAACGGATCTCTTGGTTCTGGCATCGATGAAGAACGCAGCGAAATGCGATAAGTAA
TGTGAATTGCAGAATTCAGTGAATCATCGAATCTTTGAACGCACATTGCGCCCATTAGTATTCTA
GTGGGCATGCCTGTTCGAGCGTCATTTCAACCCTTAAGCCTAGCTTAGTGTTGGGAGCCTACTG-
-CTTTTGCTAGCGGTAGCTCCTGAAATACAACGGCGGATCTGCGATATCCTCTGAGCGTAGTAA-
TTTTTATCTCGCTTTTGACTGGAGTTGCAGCGTCTTTAGCCGCTAAACCCCCC-AA-
TTTTTAATGGTTGACCTCAGGTTAGTCATCCTCAAA-TCCC-ATCATTC------CCATC--------
CTCATCATCATCGCCT--CGCAAA----CA-TTTTCCAACC-GGTGCCGAGAATCT-G--------
TTTTCGCACCTGCC-CATTTTCCCAG--ACACTTACCCC--------
GCCGCACGACCCCGCGGTGCAAACGAAAAATTTCTTA--
TCACAGCCCCACATCGCACAAACATTTTGGCAGCCATGCACTTTCCAAGACCCACAATGAACAT
TTGCTGACCCCGCCAAATAGGAAGCCGCCGAGCTCGGAAAGGGTTCCTTCAAGTACGCATGGG
TTCTTGACAAGCTCAAGGCCGAGCGTGAGCGTGGTATCACCATCGATATCGCTCTCTGGAAGTT
CGAGACCAACGAGTACAATGTCACCGTCATTGGTTAGTATCCCTGTCCACACGATGTACCATGC
ATCTGAATGT-
ATACTAACATGGCAACACAGATGCTCCCGGTCACCGTGATTTCATCAATGCTGCTTTCTGGTATG
TAACC-TGTCTGTCTCGACACCGCCTC-ATACGACAAC-----GACGTTCTA---C--
GACGTTCTCAACAAGTGCTTGGTTGGAAACAAGGGAAAGACTTGATACTGACCGGTCCCTGATA
GGCAAACCATCTCTGGCGAACACGGTCTCGACAGCAATGGAGTGTACGTACCCTTTCCTTAGCT
ACTTG-TTTCCCACGAACATCTTAGCTAACACTCG-
TGGTTGTTCAGCTACAACGGTACCTCCGAGCTCCAGCTCGAGCGCATGAGCGTCTACTTCAACG
AGGCTTCCGGCAACAAGTACGTTCCTCGTGCCGTCCTCGTCGATCTCGAGCCCGGTACCATGGA
TGCCGTCCGCGCCGGTCCTTTCGGTCAGCTCTTCCGCCCTGACAACTTCGTCTTCGGTCAGTCCG
GTGCC
>P_longiappendiculata_LC3013
???????????????????????????????ACCCATGTGAACTTACC-
ATTGTTGCCTCGGCAGAAGCTACCCGGT---
ACCTTACCTTGGAACGGCCTACCCTGTAGCGCCTTACCCTGGAACGGCTTACCCTGCAGCGGCT
GCCGGTGGACTACCAAACTCTTGTTATTTTTATGTTATCTGAGCGTCTTATTTTAATAAGTCAAAA
CTTTCAACAACGGATCTCTTGGTTCTGGCATCGATGAAGAACGCAGCGAAATGCGATAAGTAAT
GTGAATTGCAGAATTCAGTGAATCATCGAATCTTTGAACGCACATTGCGCCCATTAGTATTCTAG
TGGGCATGCCTGTTCGAGCGTCATTTCAACCCTTAAGCCTAGCTTAGTGTTGGGAGCCTACTG--
CTTTTGTTAGCTGTAGCTCCTGAAATACAACGGCGGATCTGCGATATCCTCTGAGCGTAGTAATT
TTTTTCCTCGCTTTTGACTGGAGTTGCAGCGTCTTTAGCCGCTAAACCCCCC-AA-
TTTTTAATGGTTGACCTCAGGTTAGTTATCCTCACAATTCC-ATCATCC-------------------
TCACTATCATTACCT--CGCAAA----TA-CTTCCAAACC-GGTGTCGAAAATAT-

GTGCTTCTTTTTTCGCGCCTGCC-CACATCCCCAG--ACACTTACCCC--------
GCCGCACGACCCCGCGGTGCAAACGAAAAATTTCTTA--
TCACAGCCCCACACCGCACAAACATTTTGGCAGCCATGCACTTTTCATAACCCAC-
ACGAGCATTTGCTGACCCCGCCAAATAGGAAGCCGCCGAGCTCGGAAAGGGTTCCTTCAAGTA
CGCATGGGTTCTTGACAAGCTCAAGGCCGAGCGTGAGCGTGGTATCACCATCGATATCGCTCTC
TGGAAGTTCGAGACCAACGAGTACAATGTCACCGTCATTGGTTAGTATCCCTGTCCACA---
TCCATCATGCATCCGAAATC-
AGACTAACACGGCACCACAGATGCTCCCGGTCACCGTGATTTCATCAATGCTGCCTTCTGGTAT
GTATCC-TATCTACCTCGGCATTGCCTCGATATGACGCCTTTTGACGAGTCTACGAC--
GACCTTG--AAC----
GCTTGATTAGGTCACGGATAAAAACACGATGCTAATGGGTCATTGATAGGCAAACCATCTCTGG
CGAGCACGGTCTCGACAGCAATGGAGTGTACGTACCATCTCC-T-CCGA-
TTGCTTCTTGTTGAGCACACGAACTAACTTGTG--
GCCTTGTTAGCTACAACGGTACCTCCGAGCTCCAGCTCGAGCGCATGAGTGTCTACTTCAATGA
GGCTTCCGGCAACAAGTACGTCCCTCGTGCCGTTCTCGTCGATCTCGAGCCTGGTACCATGGAT
GCCGTCCGCGCCGGTCCCTTCGGTCAGCTTTTCCGCCCTGACAACTTCGTCTT????????????????
>P_lushanensis_LC4344
???????????????????????????????ACCCATGTGAACTTACC-
ATTGTTGCCTCGGCAGAAGCTGCTCGGT-
GCACCTTACCCTGGAACGGCCTACCCTGTAGCGCCTTACCCTGGAACGGCTTACCCTGTAGCGG
CTGCCGGTGGACTACCAAACTCTTGTTATTTTATTGTAATCTGAGCGTCTTATTTTAATAAGTCAA
AACTTTCAACAACGGATCTCTTGGTTCTGGCATCGATGAAGAACGCAGCGAAATGCGATAAGTA
ATGTGAATTGCAGAATTCAGTGAATCATCGAATCTTTGAACGCACATTGCGCCCATTAGTATTCT
AGTGGGCATGCCTGTTCGAGCGTCATTTCAACCCTTAAGCCTAGCTTAGTGTTGGGAGCCTACT
G--
CTTTTGCTAGCGGTAGCTCCTGAAATACAACGGCGGATCTGCGATATCCTCTGAGCGTAGTAA-
TTTTTATCTCGCTTTTGACTGGAGTTGCAGCGTCTTTAGCCGCTAAATCCCCC-AA-
TTTTTAATGGTTGACCTCAGGTTAGTCATCCTCACAATCCC-ATCATCC--------------------
CCATCATCATCGTCT--CGCAAA----CA-TTTTCCAACT-GGTGCCGAAAATCT-G--------
TTTTCGCACCTGCC-CATTTTCCCAG--ACACTTACCCC--------
GCCGCACGACCCCGCGGTGCAAACGAAAAATTTCTTA--
TCACAGCCCCACATCGCACAAACATTTTGGCAGCCATGCACTTTCCATGACCCACAATGAACAA
TTGCTGACCCCGCCAAATAGGAAGCCGCCGAGCTCGGAAAGGGTTCCTTCAAGTACGCATGGG
TTCTTGACAAGCTCAAGGCCGAGCGTGAGCGTGGTATCACCATTGATATCGCTCTCTGGAAGTT
CGAGACCAACGAGTACAATGTCACCGTCATTGGTTAGTATCCCGGTCCACACCATGCACCATGC
ATCCGACCTT-
GTACTAACATCACAATACAGATGCTCCCGGTCACCGTGATTTCATCAATGCTGCCTTCTGGTACG
TAGTC-CATCTACCTCGACAC-GCCTCAATACGACAACCCTCCGCAACTCGACAAC--
GACCTTCTCAACAACTGCTTGGTTGGAACCAAAGAAAAGACCTGATACTGACCGGTCTCTGATA
GGCAAACCATCTCTGGCGAGCACGGTCTCGACAGCAATGGAGTGTACGTACCCTTTCCTTGGCT
ACTTGCTTTCCCACGAACATCTCAGCTAATACCCG-
TGATTGTGCAGCTACAACGGTACCTCTGAGCTCCAGCTCGAGCGCATGAGCGTCTACTTCAACG
AGGCTTCCGGCAACAAGTACGTTCCTCGTGCCGTCCTCGTCGATCTCGAGCCCGGTACCATGGA
TGCCGTCCGCGCCGGTCCTTTCGGTCAGCTCTTCCGCCCTGACAACTTTGTCTTCGGTCAGTCCG

GTGCC
>P_macadamiae_BRIP_63738B
??????????????????????AAACTCCCAACCCATGTGAACTTACC-
ATTGTTGCCTCGGCAGAAGCTGCTCGGT-
GCACCCTACCTTGGAACGGCCTACCCTGTAGCGCCTTACCCTGGAACGGCTTACCCTGGAACG
GCTGCCGGTGGACTACCAAACTCTTGTTATTTTATTGTAATCTGAGCGTCTTATTTTAATAAGTCA
AAACTTTCAACAACGGATCTCTTGGTTCTGGCATCGATGAAGAACGCAGCGAAATGCGATAAGT
AATGTGAATTGCAGAATTCAGTGAATCATCGAATCTTTGAACGCACATTGCGCCCATTAGTATTC
TAGTGGGCATGCCTGTTCGAGCGTCATTTCAACCCTTAAGCCTAGCTTAGTGTTGGGAGCCTACT
G--
CTTTTGCTAGCTGTAGCTCCTGAAATACAACGGCGGATCTGCGATATCCTCTGAGCGTAGTAAAT
TTTTATCTCGCTTTTGACTGGAGTTGCAGCGTCTTTGGCCGCTAAATCCCCC-AA-
TTTTTAATGGTTGACCTC??
?????????????????????????????????AAAATCTGG--------TTTTCGCACCTGCC-
CATTTTCTCAG--ACACTTACCCC--------
GCCGCACGACCCCGCGGTGCAAACGAAAAATTTCTTA--
TCACAGCCCCACATCACACAAACATTTTGGCAGCCACGCACCTTGCATGACCCACAATGAACAA
TTGCTGACCCCGCCAAATAGGAAGCCGCCGAGCTCGGTAAGGGTTCCTTCAAGTACCTGTG????
??
??
?????????????????????????????????A---------------AAC----------------------------
GACATTCTTAAC---
TGCTTGTTTGGAACCATACAAAAGACTTGATACTGACCGGTCTATGATAGGCAAACCATCTCTG
GCGAGCACGGTCTCGACAGCAATGGAGTGTATGTACCCTTTCCCTGGCTACTCGCTTTCTCGTG
AACATGTCAGCTAACAGTCG-
TGCTTGTTCAGCTACAACGGTACCTCCGAGCTCCAGCTCGAGCGCATGAGCGTCTACTTCAACG
AGGCTTCCGGCAACAAGTACGTTCCTCGTGCCGTCCTCGTCGATCTCGAGCCCGGTACCATGGA
TGCCGTCCGCGCCGGTCCTTTCGGTCAGCTCTTCCGCCCTGACAACTTCGTCTTCGGTCAGTCCG
GTGCC
>P_malayana_CBS_102220
AGGGATCATTATAGAGTTTTCTAAACTCCCAACCCATGTGAACTTACC-
ATTGTTGCCTCGGCAGAAGCTGCTCGGT-
GCACCTTACCTTGGAACGGCCTACCCTGTAGCGCCTTACCCTGGAACGGCTTACCCTGCAACGG
CTGCCGGTGGACTACTAAACTCTTGTTATTTTATTGTAATCTGAGCGTCTTATTTTAATAAGTCAA
AACTTTCAACAACGGATCTCTTGGTTCTGGCATCGATGAAGAACGCAGCGAAATGCGATAAGTA
ATGTGAATTGCAGAATTCAGTGAATCATCGAATCTTTGAACGCACATTGCGCCCATTAGTATTCT
AGTGGGCATGCCTGTTCGAGCGTCATTTCAACCCTTAAGCCTAGCTTAGTGTTGGGAGCCTACT
G--
CTTTTGCTAGCGGTAGCTCCTGAAATACAACGGCGGATCTGCGATATCCTCTGAGCGTAGTAA-
TTTTTATCTCGCTTTTGACTGGAGTTGCAGCGTCTTTAGCCGCTAAACCCCCC-AA-
TTTTTAATGGTTGACCTCAGGTTAGTCATGCTCACAGTCCC-ATCATCC------TCATC---------
--ACCATCATCGCCT--CGCAAA----CA-TTTTCCAACC-GTTGCCGAAATTCT-G--------
TTTTCGCACCTGCC-CATTTTCCCAG--ACAATTACCCC--------
GCCGCACGACCCCGCGGTGCAAACGAAAAATTTCTTA--TCACAGCCCCACTTCACAC-

AACATTTTGGCAGCCACGCACTTTGCATGACCCACAATGAACAATTGCTGACCCCGCCAAATAG
GAAGCCGCCGAGCTCGGAAAGGGTTCCTTCAAGTACGCATGGGTTCTTGACAAGCTCAAGGCC
GAGCGTGAGCGTGGTATCACCATTGATATCGCTCTCTGGAAGTTCGAGACCAACGAGTACAATG
TCACCGTCATTGGTTAGTATCCGTGTCCACAGAAAGTATCATGTGTCCGAACTC-
AGACTAACATTGCAATACAGACGCTCCCGGTCACCGTGATTTCATCAATGCTGCCTTCTGGTATG
TAGCC-CAACTACCTCGACAC-GCCTCAATACGACA-CCCTCCACAACTCGACGAC--
GGCATTCTCGGC---
TACTTGGTTGGAACCAAACGAAAGACTTGATACTGACCGGTCTCTGATAGGCAAACCATCTCTG
GCGAGCACGGTCTCGACAGCAATGGAGTGTACGTACCGCTTCCTTGGCTACTTGCTTTCCCACG
GACTTGTTAGCTAACACTCG-
TGCTTGCTCAGCTACAACGGCACCTCCGAGCTCCAGCTCGAGCGCATGAGCGTCTACTTCAACG
AGGCTTCCGGCAACAAGTACGTTCCTCGTGCTGTCCTCGTCGATCTCGAGCCCGGTACCATGGA
CGCCGTCCGCGCCGGTCCTTTCGGCCAGCTCTTCCGCCCTGACAACTTCGTCTTCGGTCAGTCC
GGTGCC
>P_monochaeta_CBS_144_97
AGGGATCATTATAGAGTTTTCTAAACTCCCAACCCATGTGAACTTACC-
ATTGTTGCCTCGGCAGAAGCTACCTGGT--
TACCCTACCTTGGAACGGCCTACCCTGTAGCGCCTTACCCTGGAACGGCCTACCCTGTAACGGC
TGCCGGTGGACTACCAAACTCTTGTTATTTTATTGTAATCTGAGCGTCTTATTTTAATAAGTCAAA
ACTTTCAACAACGGATCTCTTGGTTCTGGCATCGATGAAGAACGCAGCGAAATGCGATAAGTAA
TGTGAATTGCAGAATTCAGTGAATCATCGAATCTTTGAACGCACATTGCGCCCATTAGTATTCTA
GTGGGCATGCCTGTTCGAGCGTCATTTCAACCCTTAAGCCTAGCTTAGTGTTGGGAGCCTACCG-
-CTTTTGCTAGCGGTAGCTCCTGAAATACAACGGCGGATCTGCGATATCCTCTGAGCGTAGTAA-
TTTTTATCTCGCTTTTGACTGGAGTTGCAGCGTCTTTAGCCGCTAAACCCCCCAAATTTTTTAATG
GTTGACCTCAGGTTAGTCATCCACACAATCCC-
ATCATCATCATTCCCATCATTCCCATCTTATCATCATCGCCT----CAAA----CA-TCTTCCAACC-
GGTGCCGAAAATCT-G--------TTTTCGCACCTGCC-CATTTTCCCAG--ACAGTTACCCC-----
---GCCGCACGACCCCGCGGTGCAAACGAAAAATTTCTTA--
TCACAGCCCCACATCGCACAAACATTTTGGCAGCCATGCACTTTCCAAGACCCACAATGAACAA
TTGCTGACCCCGCCAAATAGGAAGCCGCCGAGCTCGGAAAGGGTTCCTTCAAGTACGCATGGG
TTCTTGACAAGCTCAAGGCCGAGCGTGAGCGTGGTATCACCATTGATATCGCTCTCTGGAAGTT
CGAGACCAACGAGTACAATGTCACCGTCATTGGTTAGTATCCCTGCCCACACCATGTATCATGC
ATCCGACCTT-
GTGCTAACATGGCAACACAGATGCTCCCGGTCACCGTGACTTCATCAATGCTGCCTTTTGGTATG
TAGCC-CATCTACCTCGACAC-GCCTCAATACGACAACCCCCCGCAACTCGACAAC--
GACGTTCTCAACAACTGCTTGGTTGGAAACAAAGGAAAGACTTGATACTGACCGGTCCTTAATA
GGCAAACCATCTCTGGCGAGCACGGTCTCGACAGCAATGGAGTGTACGTACCCTTTCCTTGGCT
ACTTGCTTTCCCACGAACATCTCAGCTAACACTCG-
TGGTTGTTCAGCTACAACGGTACCTCCGAGCTCCAGCTCGAGCGCATGAGCGTCTACTTCAACG
AGGCTTCCGGCAACAAGTACGTTCCTCGTGCCGTCCTCGTCGATCTCGAGCCCGGTACCATGGA
TGCCGTCCGCGCCGGTCCTTTCGGTCAGCTCTTCCGCCCTGACAACTTCGTCTTCGGTCAGTCCG
GTGCC
>P_monochaeta_CBS_440_83
AGGGATCATTATAGAGTTTTCTAAACTCCCAACCCATGTGAACTTACC-

ATTGTTGCCTCGGCAGAAGCTACCTGGT--
TACCCTACCTTGGAACGGCCTACCCTGTAGCGCCTTACCCTGGAACGGCCTACCCTGTAACGGC
TGCCGGTGGACTACCAAACTCTTGTTATTTTATTGTAATCTGAGCGTCTTATTTTAATAAGTCAAA
ACTTTCAACAACGGATCTCTTGGTTCTGGCATCGATGAAGAACGCAGCGAAATGCGATAAGTAA
TGTGAATTGCAGAATTCAGTGAATCATCGAATCTTTGAACGCACATTGCGCCCATTAGTATTCTA
GTGGGCATGCCTGTTCGAGCGTCATTTCAACCCTTAAGCCTAGCTTAGTGTTGGGAGCCTACCG-
-CTTTTGCTAGCGGTAGCTCCTGAAATACAACGGCGGATCTGCGATATCCTCTGAGCGTAGTAA-
TTTTTATCTCGCTTTTGACTGGAGTTGCAGCGTCTTTAGCCGCTAAACCCCCCAAATTTTTTAATG
GTTGACCTCAGGTTAGTCATCCACACAATCCC-
ATCATCATCATTCCCATCATTCCCATCTTATCATCATCGCCT----CAAA----CA-TCTTCCAACC-
GGTGCCGAAAATCT-G--------TTTTCGCACCTGCC-CATTTTCCCAG--ACAGTTACCCC-----
---GCCGCACGACCCCGCGGTGCAAACGAAAAATTTCTTA--
TCACAGCCCCACATCGCACAAACATTTTGGCAGCCATGCACTTTCCAAGACCCACAATGAACAA
TTGCTGACCCCGCCAAATAGGAAGCCGCCGAGCTCGGAAAGGGTTCCTTCAAGTACGCATGGG
TTCTTGACAAGCTCAAGGCCGAGCGTGAGCGTGGTATCACCATTGATATCGCTCTCTGGAAGTT
CGAGACCAACGAGTACAATGTCACCGTCATTGGTTAGTATCCCTGCCCACACCATGTATCATGC
ATCCGACCTT-
GTGCTAACATGGCAACACAGATGCTCCCGGTCACCGTGACTTCATCAATGCTGCCTTTTGGTATG
TAGCC-CATCTACCTCGACAC-GCCTCAATACGACAACCCCCCGCAACTCGACAAC--
GACGTTCTCAACAACTGCTTGGTTGGAAACAAAGGAAAGACTTGATACTGACCGGTCCTTAATA
GGCAAACCATCTCTGGCGAGCACGGTCTCGACAGCAATGGAGTGTACGTACCCTTTCCTTGGCT
ACTTGCTTTCCCACGAACATCTCAGCTAACACTCG-
TGGTTGTTCAGCTACAACGGTACCTCCGAGCTCCAGCTCGAGCGCATGAGCGTCTACTTCAACG
AGGCTTCCGGCAACAAGTACGTTCCTCGTGCCGTCCTCGTCGATCTCGAGCCCGGTACCATGGA
TGCCGTCCGCGCCGGTCCTTTCGGTCAGCTCTTCCGCCCTGACAACTTCGTCTTCGGTCAGTCCG
GTGCC
>P_montellica_MFLUCC_12_0279
??????CATTATAGAGTTTTTCAAACTCCCAACCCATGTGAACTTACC-
ACTGTTGCCTCGGCAGAGGCTACCCGGT---ACCT--
CCCTGGAACGGCCTACCCTGTAGCGCCCGACC------
CGGGTTACCCTGTAGCGGCTGCCGGTGGACCACTAAACTCTTGTTATTTTATTGTAATCTGAGCG
TCTTATATTAATAAGTCAAAACTTTCAACAACGGATCTCTTGGTTCTGGCATCGATGAAGAACGC
AGCGAAATGCGATAAGTAATGTGAATTGCAGAATTCAGTGAATCATCGAATCTTTGAACGCACA
TTGCGCCCATTAGTATTCTAGTGGGCATGCCTGTTCGAGCGTCATTTCAACCCTTAAGCCTAGCT
TAGTGTTGGGAGCCTACTAGCCCTCCGGGGCCTGTAGCTCCTGAAATACAACGGCGGATCTGTG
ATATCCTCTGAGCGTAGTAA-TTTTTATCTCGCTTTTGTTAGGATTCTCAGCATCT--
AGCCGCTAAACCCCCA-AT-TTTTTAATGGTTGACCTCAGGTTAGTCATCCTCACGACCTC-
ATCATAC-------------------CATGCCCACGTCACTCTGCCCAAATCACCATTTTGC-------
GTGTTGAAAATTT---TTTTTTTTTTCGCATCTGCC-CACATTCTCTG--ACACTTACCCC-------
-GCCGCACGACCCCGCGGTGCCAACGAAAAATTTCTTA--
TCACAGCCCCACCTCGCACGCAAA-----AAAATCATGCATTCTTCTGGACCCACATTGAGCA-
CTGCTAACCCCGCCAATTAGGAAGCCGCCGAGCTCGGTAAGGGTTCCTTCAAGTACGCATGGG
TTCTTGACAAGCTCAAGGCCGAGCGTGAGCGTGGTATCACCATCGATATTGCTCTCTGGAAGTT
CGAGACCAACGAGTACAATGTCACCGTCATTGGTTAGTATCCCTATCCAGATGGAGCGTTAAGC

ATCCGACCGT-
CGACTAACATTGCAACACAGATGCTCCCGGTCACCGTGACTTCATCAATGCTGCTTTCTGGTATG
TAGCCACGACTACCCCGACAC-GCCTCATCGCGCCG-CTTCTCGCAACTCTA-----
CGACAATCTCGGACTAT-
TCTGGCGGCAGCAACGTGAGAGGCATGATACTGACTGGTCTCCGACAGGCAAACTATCTCTGG
CGAGCACGGTCTCGACAGCAATGGAGTGTACGTACCATATCCCTGACTACTCGCTTTCTGACGA
ACATGCAAGCTAAACTTCG-
TGGTCCAATAGTTACAACGGTACCTCCGAGCTCCAGCTCGAGCGTATGAGCGTCTACTTCAACG
AGGCTTCCGGCAACAAGTACGTTCCCCGTGCCGTCCTCGTCGATCTCGAGCCCGGTACCATGGA
TGCCGTCCGCGCCGGTCCTTTCGGTCAGCTCTTCCGCCCTGACAACTTCGTCTTCGGTCAGTCCG
GTGCT
>P_nanjingensis_CSUFTCC16
????????????????????????????????????TGTGAACTTACC-
ATTGTTGCCTCGGCAGAAGCTGCTCGGC-
GCGCCTTACCTTGGAACGGCCTACCCTGTAGCGCCTTACCCTGGAACGGCTTACCCTGCAACGG
CTGCCGGTGGACTACCAAACTCTTGTTATTTTATGGTTATCTGAGCGTCTTATTTTAATAAGTCAA
AACTTTCAACAACGGATCTCTTGGTTCTGGCATCGATGAAGAACGCAGCGAAATGCGATAAGTA
ATGTGAATTGCAGAATTCAGTGAATCATCGAATCTTTGAACGCACATTGCGCCCATTAGTATTCT
AGTGGGCATGCCTGTTCGAGCGTCATTTCAACCCTTAAGCCTAGCTTAGTGTTGGGAGCCTACT
G--
CTTTTGCTAGCTGTAGCTCCTGAAATACAACGGCGGATCTGCGATATCCTCTGAGCGTAGTAA-
TTTTTATCTCGCTTTTGACTGGAGTTGCAGCGTCTTTAGCCGCTAAACCCCCC-AA-
TTTTTAATGGTTGACCTCAGGTTAGTCATCCTCGCAATCCC-ATCATCC------CCATT--------
CTCATCGTCATCACCT--CGCAAA----CA-TTTTCCAACC-GGTCCCGAAATTCT-G--------
TTTTCGCACCTGCC-CACTTTCCCAG--ACACTTACCCC--------
GCCGCACGACCCCGCGGTGCAAACGAAAAATTTCTTA--TCATAGCCCCACTTCACAC-
AACATTTTGGCAGCCACGCACTTTGCATGACCCATAATGAACAATTGCTGACCCCGCCAAATAG
GAAGCCGCCGAGCTCGGAAAGGGTTCCTTCAAGTACGCATGGGTTCTTGACAAGCTCAAGGCC
GAGCGTGAGCGTGGTATCACCATTGATATCGCTCTCTGGAAGTTCGAGACCAACGAGTACAATG
TCACCGTCATTGGTTAGTATCCCTGTCCACAGAAAGTATCATGTGTCCGAACAC-
AGACTAACATCGCGATACAGACGCTCCCGGTCACCGTGATTTCATCAATGCTGCCTTCTGGTAT
GTAGCC-CATCTACCTCGACAC-GCCTCAATACGACA-CCCTCCGCAACTCGACGAC--
GACATTCTCGGC---
TTCTTGGTTGGAACCGAACCAAAGACTTGATACTGACCGGTCTCTGATAGGCAAACCATCTCTG
GCGAGCACGGTCTCGACAGCAATGGAGTGTACGTACCGTTTCCTTGCCTACTTGCTTTCCCACG
AACATGTTAGCTAACACTCG-
TGCTTGCTCAGCTACAACGGCACCTCCGAGCTCCAGCTCGAGCGCATGAGCGTCTACTTCAACG
AGGCTTCCGGCAACAAGTACGTTCCTCGTGCCGTCCTCGTCGATCTCGAGCCCGGTACCATGGA
CGCCGTCCGCGCCGGTCCTTTCGGCCAGCTCTTCCGCCCTGACAACTTCGTCTTCGGTCAGTCC
GGTGCC
>P_nanjingensis_CSUFTCC20
????????????????????????????????????TGTGAACTTACC-
ATTGTTGCCTCGGCAGAAGCTGCTCGGC-
GCGCCTTACCTTGGAACGGCCTACCCTGTAGCGCCTTACCCTGGAACGGCTTACCCTGCAACGG

CTGCCGGTGGACTACCAAACTCTTGTTATTTTATGGTTATCTGAGCGTCTTATTTTAATAAGTCAA
AACTTTCAACAACGGATCTCTTGGTTCTGGCATCGATGAAGAACGCAGCGAAATGCGATAAGTA
ATGTGAATTGCAGAATTCAGTGAATCATCGAATCTTTGAACGCACATTGCGCCCATTAGTATTCT
AGTGGGCATGCCTGTTCGAGCGTCATTTCAACCCTTAAGCCTAGCTTAGTGTTGGGAGCCTACT
G--
CTTTTGCTAGCTGTAGCTCCTGAAATACAACGGCGGATCTGCGATATCCTCTGAGCGTAGTAA-
TTTTTATCTCGCTTTTGACTGGAGTTGCAGCGTCTTTAGCCGCTAAACCCCCC-AA-
TTTTTAATGGTTGACCTCAGGTTAGTCATCCTCGCAATCCC-ATCATCC------CCATT--------
CTCATCGTCATCACCT--CGCAAA----CA-TTTTCCAACC-GGTCCCGAAATTCT-G--------
TTTTCGCACCTGCC-CACTTTCCCAG--ACACTTACCCC--------
GCCGCACGACCCCGCGGTGCAAACGAAAAATTTCTTA--TCATAGCCCCACTTCACAC-
AACATTTTGGCAGCCACGCACTTTGCATGACCCATAATGAACAATTGCTGACCCCGCCAAATAG
GAAGCCGCCGAGCTCGGAAAGGGTTCCTTCAAGTACGCATGGGTTCTTGACAAGCTCAAGGCC
GAGCGTGAGCGTGGTATCACCATTGATATCGCTCTCTGGAAGTTCGAGACCAACGAGTACAATG
TCACCGTCATTGGTTAGTATCCCTGTCCACAGAAAGTATCATGTGTCCGAACAC-
AGACTAACATCGCGATACAGACGCTCCCGGTCACCGTGATTTCATCAATGCTGCCTTCTGGTAT
GTAGCC-CATCTACCTCGACAC-GCCTCAATACGACA-CCCTCCGCAACTCGACGAC--
GACATTCTCGGC---
TTCTTGGTTGGAACCGAACCAAAGACTTGATACTGACCGGTCTCTGATAGGCAAACCATCTCTG
GCGAGCACGGTCTCGACAGCAATGGAGTGTACGTACCGTTTCCTTGCCTACTTGCTTTCCCACG
AACATGTTAGCTAACACTCG-
TGCTTGCTCAGCTACAACGGCACCTCCGAGCTCCAGCTCGAGCGCATGAGCGTCTACTTCAACG
AGGCTTCCGGCAACAAGTACGTTCCTCGTGCCGTCCTCGTCGATCTCGAGCCCGGTACCATGGA
CGCCGTCCGCGCCGGTCCTTTCGGCCAGCTCTTCCGCCCTGACAACTTCGTCTTCGGTCAGTCC
GGTGCC
>P_nanningensis_CSUFTCC10
???????????????????????????????????TGTGAACTTACC-
ATTGTTGCCTCGGCAGAAGCTGCTCGGT-
GCACCTTACCTTGGAACGGCCTACCCTGTAGCGCCTTACCCTGGAACGGCTTACCCTGTAGCGG
CTGCCGGCGGACTACCAAACTCTTGTTATTTTATTGTAATCTGAGCGTCTTATTTTAATAAGTCAA
AACTTTCAACAACGGATCTCTTGGTTCTGGCATCGATGAAGAACGCAGCGAAATGCGATAAGTA
ATGTGAATTGCAGAATTCAGTGAATCATCGAATCTTTGAACGCACATTGCGCCCATTAGTATTCT
AGTGGGCATGCCTGTTCGAGCGTCATTTCAACCCTTAAGCCTAGCTTAGTGTTGGGAGCCTACT
G--
CTTTTACTAGCTGTAGCTCCTGAAATACAACGGCGGATCTGCGATATCCTCTGAGCGTAGTAA-
TTTTTATCTCGCTTTTGACTGGAGTTGCAGCGTCTTTAGCCGCTAAACCCCCC-AA-
TTTTTAATGGTTGACCTCAGGTTAGTCATCCTCGCAATCCC-ATCATCC------TCATC--------
TTCATCATCACCACCT--CGCAAA----GA-TTTCCACACC-GGTGCCGAAAATCT-G--------
TTTTCGCACCTGCC-CATTTTCCCAG--ACACTTACCCC--------
GCCGCACGACCCCGCGGTGCAAACGAAAAATTTCTTA--TCATAGCCCCACATCAC--
AAACATTTTGGCAGCCACGCACTCTGCATGACCCACAATGAACAATTGCTGACCCCGCCAAATA
GGAAGCCGCTGAGCTCGGAAAGGGTTCCTTCAAGTACGCATGGGTTCTTGACAAGCTCAAGGC
CGAGCGTGAGCGTGGTATCACCATTGATATCGCTCTCTGGAAGTTCGAGACCAACGAGTACAAT
GTCACCGTCATTGGTTAGTATCCCTGCCCACAACATGTGTCATGTCTCCGAACTCAAGACTAACC

TTACAATACAGACGCTCCCGGTCACCGTGATTTCATCAATGCTGCCTTCTGGTATGTAGCC-
CATCTACCCCGACGC-GTCTCAATACGACA-CCCCCGGCAACTCGACAAC--GACGTTCTCAAC-
--
TGCTTGGTTGAAACCAAATGAAAGACTTGATACTGACCGGTCTCTGATAGGCAAACCATCTCTG
GCGAGCACGGTCTCGACAGCAATGGAGTGTACGTACCCTTTCCCTGGCTACTCGCTTTCTCGTG
AACATGTCAGCTAACACTCG-
TGCTTGTTCAGCTACAACGGTACCTCCGAGCTCCAGCTCGAGCGCATGAGCGTCTACTTCAACG
AGGCTTCCGGCAACAAGTACGTTCCTCGTGCCGTCCTCGTCGATCTCGAGCCCGGTACCATGGA
TGCCGTCCGCGCCGGTCCTTTCGGTCAGCTCTTCCGCCCTGACAACTTCGTCTTCGGTCAGTCCG
GTGCC
>P_nanningensis_CSUFTCC11
????????????????????????????????????TGTGAACTTACC-
ATTGTTGCCTCGGCAGAAGCTGCTCGGT-
GCACCTTACCTTGGAACGGCCTACCCTGTAGCGCCTTACCCTGGAACGGCTTACCCTGTAGCGG
CTGCCGGCGGACTACCAAACTCTTGTTATTTTATTGTAATCTGAGCGTCTTATTTTAATAAGTCAA
AACTTTCAACAACGGATCTCTTGGTTCTGGCATCGATGAAGAACGCAGCGAAATGCGATAAGTA
ATGTGAATTGCAGAATTCAGTGAATCATCGAATCTTTGAACGCACATTGCGCCCATTAGTATTCT
AGTGGGCATGCCTGTTCGAGCGTCATTTCAACCCTTAAGCCTAGCTTAGTGTTGGGAGCCTACT
G--
CTTTTACTAGCTGTAGCTCCTGAAATACAACGGCGGATCTGCGATATCCTCTGAGCGTAGTAA-
TTTTTATCTCGCTTTTGACTGGAGTTGCAGCGTCTTTAGCCGCTAAACCCCCC-AA-
TTTTTAATGGTTGACCTCAGGTTAGTCATCCTCGCAATCCC-ATCATCC------TCATC--------
TTCATCATCACCACCT--CGCAAA----GA-TTTCCACACC-GGTGCCGAAAATCT-G--------
TTTTCGCACCTGCC-CATTTTCCCAG--ACACTTACCCC--------
GCCGCACGACCCCGCGGTGCAAACGAAAAATTTCTTA--TCATAGCCCCACATCAC--
AAACATTTTGGCAGCCACGCACTCTGCATGACCCACAATGAACAATTGCTGACCCCGCCAAATA
GGAAGCCGCTGAGCTCGGAAAGGGTTCCTTCAAGTACGCATGGGTTCTTGACAAGCTCAAGGC
CGAGCGTGAGCGTGGTATCACCATTGATATCGCTCTCTGGAAGTTCGAGACCAACGAGTACAAT
GTCACCGTCATTGGTTAGTATCCCTGCCCACAACATGTGTCATGTCTCCGAACTCAAGACTAACC
TTACAATACAGACGCTCCCGGTCACCGTGATTTCATCAATGCTGCCTTCTGGTATGTAGCC-
CATCTACCCCGACGC-GTCTCAATACGACA-CCCCCGGCAACTCGACAAC--GACGTTCTCAAC-
--
TGCTTGGTTGAAACCAAATGAAAGACTTGATACTGACCGGTCTCTGATAGGCAAACCATCTCTG
GCGAGCACGGTCTCGACAGCAATGGAGTGTACGTACCCTTTCCCTGGCTACTCGCTTTCTCGTG
AACATGTCAGCTAACACTCG-
TGCTTGTTCAGCTACAACGGTACCTCCGAGCTCCAGCTCGAGCGCATGAGCGTCTACTTCAACG
AGGCTTCCGGCAACAAGTACGTTCCTCGTGCCGTCCTCGTCGATCTCGAGCCCGGTACCATGGA
TGCCGTCCGCGCCGGTCCTTTCGGTCAGCTCTTCCGCCCTGACAACTTCGTCTTCGGTCAGTCCG
GTGCC
>P_neolitseae_NTUCC_17_011
??????CATTATAGAGTTTTCTAAACTCCCAACCCATGTGAACTTACC-
ATTGTTGCCTCGGCAGAAGCTGCTCGGC-
GCGCCTTACCTTGGAACGGCCTACCCTGTAGCGCCTTACCCTGGAACGGCTTACCCTGCAACGG
CTGCCGGTGGACTACCAAACTCTTGTTATTTTATGGTTATCTGAGCGTCTTATTTTAATAAGTCAA

AACTTTCAACAACGGATCTCTTGGTTCTGGCATCGATGAAGAACGCAGCGAAATGCGATAAGTA
ATGTGAATTGCAGAATTCAGTGAATCATCGAATCTTTGAACGCACATTGCGCCCATTAGTATTCT
AGTGGGCATGCCTGTTCGAGCGTCATTTCAACCCTTAAGCCTAGCTTAGTGTTGGGAGCCTACT
G--
CTTTTGCTAGCTGTAGCTCCTGAAATACAACGGCGGATCTGCGATATCCTCTGAGCGTAGTAA-
TTTTTATCTCGCTTTTGACTGGAGTTGCAGCGTCTTTAGCCGCTAAACCCCCC-AA-
TTTTTAATGGTGC???????????????ACCCCGCAT--CC-ATCATCC---------------------
CATCCTCAT-ACCT--CGCAA-----CG-TTTTCCAACC-GGTGTCGAAAATCG-G--------
TTTTCGCACCTGCC-CATTTTCCCAG--ACACTTACCCC--------
GCCGCACGACCCCGCGGTGCAAACGAAAAATTTCTTA--TCACAGCCCCACTTCACAC-
AACATTTTGGCAGCCACGCACTTTGCATGACCCACAGTGAACAATTGCTGACCCCGCCAAATAG
GAAGCCGCCGAGCTCGGAAAGGGTTCCTTCAAGTACGCATGGGTTCTTGACAAGCTCAAGGCC
GAGCGTGAGCGTGGTATCACCATTGATATCGCTCTCTGGAAGTTCGAGACCAACGAGTACAATG
TCACCGTCATTGGTTAGTATTCCTGTCCACAGAATGTATCATGTCTCCGAACTC-
AGACTAACATCACAATACAGACGCTCCCGGTCACCGTGATTTCATCAA???????????????????????
?????????????????????CATACGACA-CCCTCCGCAACTCGACGAC--GACATTCTCGGC---
TTCTTGGTTGGAACCGAACCAAAGACTTGATACTGACCGGTCTCTGATAGGCAAACCATCTCTG
GCGAGCACGGTCTCGACAGCAATGGAGTGTACGTACCGTTCCCTTGCCTACTTGCTTTCCCACG
AACATGTTAGCTAACACTCG-
TGCTTGCTCAGCTACAACGGCACCTCCGAGCTCCAGCTCGAGCGCATGAGCGTCTACTTCAACG
AGGCTTCCGGCAACAAGTACGTTCCTCGTGCCGTCCTCGTCGATCTCGAGCCCGGTACCATGGA
CGCCGTCCGCGCCGGTCCTTTCGGCCAGCTCTTCCGCCCTGACAACTTCGTCTTCGGTCAGTCC
GGTGCC
>P_novae_hollandiae_CBS_130973
AGGGATCATTATAGAGTTTTCTAAACTCCCAACCCATGTGAACTTACC-
ATTGTTGCCTCGGCAGAAGCTACCCGGT---
ACCTTACCTTGGAACGGCCTACCCTGTAGCGCCTTACCCTGGAACGGCTTACCCTGTAACGGCT
GCCGGTGGACTACCAAACTCTTGTTATTTTATTGTAATCTGAGCGTCTTATTTTAATAAGTCAAAA
CTTTCAACAACGGATCTCTTGGTTCTGGCATCGATGAAGAACGCAGCGAAATGCGATAAGTAAT
GTGAATTGCAGAATTCAGTGAATCATCGAATCTTTGAACGCACATTGCGCCCATTAGTATTCTAG
TGGGCATGCCTGTTCGAGCGTCATTTCAACCCTTAAGCCTAGCTTAGTGTTGGGAGCCTACTG--
CTTTTGCTAGCTGTAGCTCCTGAAATACAACGGCGGATCTGCGATATCCTCTGAGCGTAGTAA-
TTTTTTTCTCGCTTTTGACTGGAGTTGCAGCGTCCTTAGCCGCTAAACCCCCC-AA-
TTTTTAATGGTTGACCTCAGGTTAGTCATCCTCACAATCCC-ATCACCA-----------------
TCCTCCTTATCACCACCT--CGCAAA----CA--TTTTAAATC-GGTGCCGAGAATCT-G--------
TTTTCGCACCTGCC-CACATTCCCAG--ACACTTACCCC--------
GCCGCACGACCCCGCGGTGCAAAAGAAAAATTTCTTA--
TCACAGCCCCACATCGCACAAACATTTTGACAGCCATGCACTTTTCATGGCCCACAATGAGGAT
TTGCTGACCCCGCCAAATAGGAAGCCGCCGAGCTCGGAAAGGGTTCCTTCAAGTACGCATGGG
TTCTTGACAAGCTCAAGGCCGAGCGTGAGCGTGGTATCACCATTGATATCGCTCTCTGGAAGTT
CGAGACCAACGAGTACAATGTCACCGTCATTGGTTAGTATACCTGTCCACACCATGCATCACGC
ATCCGAATTC-
AGACTAACATGGCAATACAGATGCTCCCGGTCACCGTGATTTCATCAATGCTGCCTTCTGGTATG
TAGCC-CATGTACCTCGACAC-GCCCCAACACGACG-CCACCCGCAACTCGACAAC--

GACGTTCTCAAC----
ACTGGGTTGGAACTAAACGAAAGACTTGATACTGACCGGTCTCTGATAGGCAAACCATTTCTGG
CGAGCACGGTCTCGACAGCAATGGAGTGTACGTACCCTTTTCTTGGCTACTTGCT-
TCCCACGAACATGTCAGCTAACACTCG-
TGTTTGTTCAGCTACAACGGTACCTCCGAGCTCCAGCTCGAGCGCATGAGCGTCTACTTCAACG
AGGCTTCCGGCAACAAGTACGTTCCTCGTGCCGTCCTCGTCGATCTCGAGCCCGGTACCATGGA
TGCCGTCCGCGCCGGTCCTTTCGGTCAGCTCTTCCGCCCTGACAACTTCGTCTTCGGTCAGTCCG
GTGCC
>P_oryzae_CBS_353_69
AGGGATCATTATAGAGTTTTCTAAACTCCCAACCCATGTGAACTTACC-
ATTGTTGCCTCGGCAGAAGCTGCTCGGT-
GCACCCTACCTTGGAACGGCCTACCCTGTAGCGCCTTACCCTGGAACGGCTTACCCTGTAACGG
CTGCCGGTGGACTACCAAACTCTTGTTATTTTATTGTAATCTGAGCGTCTTATTTTAATAAGTCAA
AACTTTCAACAACGGATCTCTTGGTTCTGGCATCGATGAAGAACGCAGCGAAATGCGATAAGTA
ATGTGAATTGCAGAATTCAGTGAATCATCGAATCTTTGAACGCACATTGCGCCCATTAGTATTCT
AGTGGGCATGCCTGTTCGAGCGTCATTTCAACCCTTAAGCCTAGCTTAGTGTTGGGAGCCTACT
G--
CTTTTGCTAGCTGTAGCTCCTGAAATACAACGGCGGATCTGCGATATCCTCTGAGCGTAGTAAAT
TTTTATCTCGCTTTTGACTGGAGTTGCAGCGTCTTTGGCCGCTAAATCCCCC-AA-
TTTTTAATGGTTGACCTCAGGTTAGTCATCCTCGCAATCCC-ATCATCC------TCATC--------
CTCATCATCATCACCT--CGCAAA----CA-TTTCCACACC-GGTGCCGAAAATCTGG--------
TTTTCGCACCTGCC-CATTTTCCCAC--ACACTTACCCC--------
GCCGCACGACCCCGCGGTGCAAACGAAAAATTTCTTA--
TCATAGCCCCACATCACACAAACATTTTGGCAGCCACGCACTTTGCATGACCCACAATGAACAA
TTGCTGACCCCACCAAATAGGAAGCCGCCGAGCTCGGAAAGGGTTCCTTCAAGTACGCATGGG
TTCTTGACAAGCTCAAGGCCGAGCGTGAGCGTGGTATCACCATTGATATCGCTCTCTGGAAGTT
CGAGACCAACGAGTACAATGTCACCGTCATTGGTTAGTATCCCTGCCCACAACATGCATCATGT
CTCCGAACTCAAGACTAACCTTTCAATACAGACGCTCCCGGTCACCGTGATTTCATCAATGCTGC
CTTCTGGTATGTAGCC-CATCTACCTCGACGC-GCCTCAATACGACA-
CCCCCGGCAACTCGACCAC--GATAATCTCAAC---
TGCTTGGTTGGAACCATACGAAAGACTCGATACTGACCGGTCTATGATAGGCAAACCATCTCTG
GCGAGCACGGTCTCGACAGCAATGGAGTGTACGTACCCTTTCCCTGGCTGTTCGCTTTCTCGTG
AACATGTCAGCTAACAGTCG-
TGCTTGTTCAGCTACAACGGTACCTCCGAGCTCCAGCTCGAGCGCATGAGCGTCTACTTCAACG
AGGCTTCCGGCAACAAGTACGTTCCTCGTGCCGTCCTCGTCGATCTCGAGCCCGGTACCATGGA
TGCCGTCCGCGCCGGTCCTTTCGGTCAGCTCTTCCGCCCTGACAACTTCGTCTTCGGTCAGTCCG
GTGCC
>P_oryzae_CL107
AGGGATCATTATAGAGTTTTCTAAACTCCCAACCCATGTGAACTTACC-
ATTGTTGCCTCGGCAGAAGCTGCTCGGT-
GCACCCTACCTTGGAACGGCCTACCCTGTAGCGCCTTACCCTGGAACGGCTTACCCTGTAACGG
CTGCCGGTGGACTACCAAACTCTTGTTATTTTATTGTAATCTGAGCGTCTTATTTTAATAAGTCAA
AACTTTCAACAACGGATCTCTTGGTTCTGGCATCGATGAAGAACGCAGCGAAATGCGATAAGTA
ATGTGAATTGCAGAATTCAGTGAATCATCGAATCTTTGAACGCACATTGCGCCCATTAGTATTCT

AGTGGGCATGCCTGTTCGAGCGTCATTTCAACCCTTAAGCCTAGCTTAGTGTTGGGAGCCTACT
G--
CTTTTGCTAGCTGTAGCTCCTGAAATACAACGGCGGATCTGCGATATCCTCTGAGCGTAGTAAAT
TTTTATCTCGCTTTTGACTGGAGTTGCAGCGTCTTTGGCCGCTAAATCCCCC-AA-
TTTTTAATGGTTGACCTCAGGTTAGTCATCCTCGCAATCCC-ATCATCC------TCATC--------
CTCATCATCATCACTT--CGCAAA----CATTTCCCACACC-GGTGTCGAAAATCTGG--------
TTTTCGCACCTGCC-CATTTTCTCAG--ACACTTACCCC--------
GCCGCACGACCCCGCGGTGCAAACGAAAAATTTCTTA--
TCACAGCCCCACATCACACAAACATTTTGGCAGCCACGCACCTTGCATGACCCACAATGAACAA
TTGCTGACCCCGCCAAATAGGAAGCCGCCGAGCTCGGTAA?????????????????????????????????
??
???TG
CTGCCTTCTGGTATGTAGCC-CATCTACCTCGACGC-GCCTCAATACGACA-
CCCCCGGCAACTCGACCAC--GATAATCTCAAC---
TGCTTGGTTGGAACCATACGAAAGACTCGATACTGACCGGTCTATGATAGGCAAACCATCTCTG
GCGAGCACGGTCTCGACAGCAATGGAGTGTACGTACCCTTTCCCTGGCTGTTCGCTTTCTCGTG
AACATGTCAGCTAACAGTCG-
TGCTTGTTCAGCTACAACGGTACCTCCGAGCTCCAGCTCGAGCGCATGAGCGTCTACTTCAACG
AGGCTTCCGGCAACAAGTACGTTCCTCGTGCCGTCCTCGTCGATCTCGAGCCCGGTACCATGGA
TGCCGTCCGCGCCGGTCCTTTCGGTCAGCTCTTCCGCCCTGACAACTTCGTCTTCGGTCAGTCCG
GTGCC
>P_pallidotheae_MAFF_240993
????????TTACAGAGTTTTCTAAACTCCCAACCCATGTGAACTTACC-
ATTGTTGCCTCGGCAGAAGCTACCCTGTAGCGCCTTACCCGGGAACGGCCTACCCTGTAGCGCC
TTACCCTGGAACGGCCTACCCTGTAGCGGCTGCCGGTGGACTACTCAACTCTTGTTATTTTATGG
TTATCTGAGCGTCTTATTTTAATAAGTCAAAACTTTCAACAACGGATCTCTTGGTTCTGGCATCGA
TGAAGAACGCAGCGAAATGCGATAAGTAATGTGAATTGCAGAATTCAGTGAATCATCGAATCTT
TGAACGCACATTGCGCCCATTAGTATTCTAGTGGGCATGCCTGTTCGAGCGTCATTTCAACCCTT
AAGCCTAGCTTAGTGTTGGGAGCCTACTG--
CTTTTGCTAGCTGTAGCTCCTGAAATACAACGGCGGATCTGCGATATCCTCTGAGCGTAGTAATT
TTTTTTCTCGCTTTTGACTGGAGTTGCAGCGTCCTTAGCCGCTAAACCCCCC-AA-
TTTTTAATGGTT??????AGGTTAGTCATCCTCACGATTCC-ATCATCT-------------------
ATCTTATCACCGCTC----CGAG----CA-TTCTCATCTC-GGTGTCGAAAATCA---------
TTTTTCGCACCTGCC-CACGTTCCCTG--
GCACTTACCCCGCCGCGTGGCCGCACGACCCCGCGGTGCAAACGAAAAATTTCTTA--
TCACAGCCCCACCTCGCACAAACATTTTGGCAGCCACGCACTTT--
ATGACCCACAATGAGCCATTGCTGACCCCGCGAAATAGGAAGCCGCCGAGCTTGGTAAGGGTT
CCTTCAAGTACGCCTGGGTTCTTGACAAGCTCAAGGCCGAGCGTGAGCGTGGTATCACCATCGA
TATCGCTCTCTGGAAGTTCGAGACCAATGAGTACAATGTCACCGTCATTGGTCAGTATCCCTGTC
CTCAATATGGACCATGCATTCCAACTC-
AGACTAACACGGCAACACAGACGCTCCCGGTCACCGTGACTTCATCAATGCTGCCTTTTGGTAT
GTGCCC-CATCTACCTCGACGC-GCCTCAACACGACG-
CCTCCCGCAACGCGACAACGCGACATTCTCCAC---
TACCTAGTTGGAATCACACGAAAGCCTTGATACTGACCGGTCTTTGATAGGCAAACCATCTCTG

GCGAGCACGGTCTCGACAGCAATGGAGTGTACGTACCCTTTCCTTGGCTACTTGCTTTCCCATG
ACCATGTTAGCTGACACTCG-
TGGTTGTTCAGCTACAACGGTACCTCTGAGCTCCAGCTGGAGCGCATGAGCGTCTACTTCAACG
AGGCTTCCGGCAACAAGTACGTTCCTCGTGCCGTCCTCGTCGATCTCGAGCCCGGTACCATGGA
TGCCGTCCGTGCCGGTCCTTTCGGTCAGCTCTTCCGCCCTGACAACTTCGTCTTCGGTCAGTCCG
GTGCC
>P_papuana_CBS_331_96
AGGGATCATTATAGAGTTTTCTAAACTCCCAACCCATGTGAACTTACC-
ATTGTTGCCTCGGCAGAAGCTGCTCGGT-
GCACCTTACCTTGGAACGGCCTACCCTGTAGCGCCTTACCCTGGAACGGCTTACCCTGCAACGG
CTGCCGGTGGACTACCAAACTCTTGTTATTTTATGGTTATCTGAGCGTCTTATTTTAATAAGTCAA
AACTTTCAACAACGGATCTCTTGGTTCTGGCATCGATGAAGAACGCAGCGAAATGCGATAAGTA
ATGTGAATTGCAGAATTCAGTGAATCATCGAATCTTTGAACGCACATTGCGCCCATTAGTATTCT
AGTGGGCATGCCTGTTCGAGCGTCATTTCAACCCTTAAGCCTAGCTTAGTGTTGGGAGCCTACT
G--
CTTTTGCTAGCTGTAGCTCCTGAAATACAACGGCGGATCTGCGATATCCTCTGAGCGTAGTAA-
TTTTTATCTCGCTTTTGACTGGAGTTGCAGCGTCTTTAGCCGCTAAACCCCCC-AA-
TTTTTAATGGTTGACCTCAGGTTAGTCATGCTCACA-TCCC-ATCATCC------TCATC---------
--ATCATCATCGCCT--CGCAAA----CA-TTTTCCAACC-GGTGCCGAAATTCT-G--------
TTTTCGCACCTGCC-CATTTTTCCAG--ACACTTACCCC--------
GCCGCACGACCCCGCGGTGCAAACGAAAAATTTCTTATCTCACAGCCCCACTTCACAC-
AACATTTTGGCAGCCACGCACTTTGCATGACCCACAATGAACAATTGCTGACCCCGCCAAATAG
GAAGCCGCCGAGCTCGGAAAGGGTTCCTTCAAGTACGCATGGGTTCTTGACAAGCTCAAGGCC
GAGCGTGAGCGTGGTATCACCATTGATATCGCTCTCTGGAAGTTCGAGACCAACGAGTACAATG
TCACCGTCATTGGTTAGTATCCCTGTCCATAGAAAGTATCATGTGTCCGAACTC-
AGACTAACATCGCAATACAGACGCTCCCGGTCACCGTGATTTCATCAATGCTGCCTTCTGGTAT
GTAGCC-CAACTACCTCGACAC-GCCTCAATACGACA-CCCTCCACAACTCGACGAC--
GGCATTCTCGGC---
TACTTGGTTGGAACCAAACGAAAGACTTGATACTGACCGGTCTCTGATAGGCAAACCATCTCTG
GCGAGCACGGTCTCGACAGCAATGGAGTGTACGTACCGCTTCCTTGGCTGCTTGCTTTCCCACG
GACTTGTTAGCTAACACTCG-
TGCTTGCTCAGCTACAACGGCACCTCCGAGCTCCAGCTCGAGCGCATGAGCGTCTACTTCAACG
AGGCTTCCGGCAACAAGTACGTTCCTCGTGCTGTCCTCGTCGATCTCGAGCCCGGTACCATGGA
CGCCGTCCGCGCCGGTCCTTTCGGCCAGCTCTTCCGCCCTGACAACTTCGTCTTCGGTCAGTCC
GGTGCC
>P_papuana_MFLU_19_2764
??????CATTATAGAGTTTTCTAAACTCCCAACCCATGTGAACTTACC-
ATTGTTGCCTCGGCAGAAGCTGCTCGGT-
GCACCTTACCTTGGAACGGCCTACCCTGTAGCGCCTTACCCTGGAACGGCTTACCCTGCAACGG
CTGCCGGTGGACTACCAAACTCTTGTTATTTTATGGTTATCTGAGCGTCTTATTTTAATAAGTCAA
AACTTTCAACAACGGATCTCTTGGTTCTGGCATCGATGAAGAACGCAGCGAAATGCGATAAGTA
ATGTGAATTGCAGAATTCAGTGAATCATCGAATCTTTGAACGCACATTGCGCCCATTAGTATTCT
AGTGGGCATGCCTGTTCGAGCGTCATTTCAACCCTTAAGCCTAGCTTAGTGTTGGGAGCCTACT
G--

CTTTTGCTAGCTGTAGCTCCTGAAATACAACGGCGGATCTGCGATATCCTCTGAGCGTAGTAA-
TTTTTATCTCGCTTTTGACTGGAGTTGCAGCGTCTTTAGCCGCTAAACCCCCC-AA-
TTTTTAATGGTTGACCTCAGGTTAGTCATGCTCACA-TCCC-ATCATCC------TCATC---------
--ATCATCATCGCCT--CGCAAA----CA-TTTTCCAACC-GGTGCCGAAATTCT-G--------
TTTTCGCACCTGCC-CATTTTTCCAG--ACACTTACCCC--------
GCCGCACGACCCCGCGGTGCAAACGAAAAATTTCTTATCTCACAGCCCCACTTCACAC-
AACATTTTGGCAGCCACGCACTTTGCATGACCCACAATGAACAATTGCTGACCCCGCCAAATAG
GAAGCCGCCGAGCTCGGAAAGGGTTCCTTCAAGTACGCATGGGTTCTTGACAAGCTCAAGGCC
GAGCGTGAGCGTGGTATCACCATTGATATCGCTCTCTGGAAGTTCGAGACCAACGAGTACAATG
TCACCGTCATTGGTTAGTATCCCTGTCCATAGAAAGTATCATGTGTCCGAACTC-
AGACTAACATCGCAATACAGACGCTCCCGGTCACCGTGATTTCATCAATGCTGCCTTCTGGTAT
GTAGCC-CAACTACCTCGACAC-GCCTCAATACGACA-CCCTCCACAACTCGACGAC--
GGCATTCTCGGC---
TACTTGGTTGGAACCAAACGAAAGACTTGATACTGACCGGTCTCTGATAGGCAAACCATCTCTG
GCGAGCACGGTCTCGACAGCAATGGAGTGTACGTACCGCTTCCTTGGCTGCTTGCTTTCCCACG
GACTTGTTAGCTAACACTCG-
TGCTTGCTCAGCTACAACGGCACCTCCGAGCTCCAGCTCGAGCGCATGAGCGTCTACTTCAACG
AGGCTTCCGGCAACAAGTACGTTCCTCGTGCTGTCCTCGTCGATCTCGAGCCCGGTACCATGGA
CGCCGTCCGCGCCGGTCCTTTCGGCCAGCTCTTCCGCCCTGACAACTTCGTCTTCGGTCAGTCC
GGTGCC
>P_parva_CBS_265_37
AGGGATCATTATAGAGTTTTCTAAACTCCCAACCCATGTGAACTTACC-
ATTGTTGCCTCGGCAGAAGCTGCTCGGT-
GCACCTTACCTTGGAACGGCCTACCCTGTAGCGCCTTACCCTGGAACGGCTTACCCTGTAGCGG
CTGCCGGTGGACTACCAAACTCTTGTTATTTTATTGTAATCTGAGCGTCTTATTTTAATAAGTCAA
AACTTTCAACAACGGATCTCTTGGTTCTGGCATCGATGAAGAACGCAGCGAAATGCGATAAGTA
ATGTGAATTGCAGAATTCAGTGAATCATCGAATCTTTGAACGCACATTGCGCCCATTAGTATTCT
AGTGGGCATGCCTGTTCGAGCGTCATTTCAACCCTTAAGCCTAGCTTAGTGTTGGGAGCCTACT
G--
CTTTTACTAGCTGTAGCTCCTGAAATACAACGGCGGATCTGCGATATCCTCTGAGCGTAGTAA-
TTTTTATCTCGCTTTTGACTGGAGTTGCAGCGTCTTTAGCCGCTAAACCCCCC-AA--
CTTTAATGGTTGACCTCAGGTTAGTCATCCTCGCAATCCC-ATCATCC------TCATC---------
--TTCATCATCACCT--CGCAAA----GA-TTTCCACATC-GGTGCCGAAAATCT-G--------
TTTTCGCACCTGCC-CATTTTCCCAG--GCAATTACCCC--------
GCCGCACGACCCCGCGGTGCAAACGAAAAATTTCTTA--TCATAGCCCCACATCAC--
AAACATTTTGGCAGCCACGCACTCTGCATGACCCACAACGAACAATTGCTGACCCCGCCAAATA
GGAAGCCGCCGAGCTCGGAAAGGGTTCCTTCAAGTACGCATGGGTTCTTGACAAGCTCAAGGC
CGAGCGTGAGCGTGGTATCACCATTGATATCGCTCTCTGGAAGTTCGAGACCAACGAGTACAAT
GTCACCGTCATTGGTTAGTATCCCTGCCCACAACATGTGTCATGTCTCCGAACTCAAGACTAACC
TTACAATACAGACGCTCCCGGTCACCGTGATTTCATCAATGCTGCCTTCTGGTATGTAGCC-
CATCTACCTCGACGC-GCCTCAATACGACA-CCCCCGGCAACTCGACAAC--GACATTCTCAAC-
--
TGCTTGGTTGAAACCATACGAAAGACTTGATACTGACCGGTCTCTGATAGGCAAACCATCTCTG
GCGAGCACGGTCTCGACAGCAATGGAGTGTACGTACCCTTTCCCTGGCTACTCGCTTTCTCGTG

AACATGTCAGCTAACACTCG-
TGCTTGTTCAGCTACAACGGTACCTCCGAGCTCCAGCTCGAGCGCATGAGCGTCTACTTCAACG
AGGCTTCCGGCAACAAGTACGTTCCTCGTGCCGTCCTCGTCGATCTCGAGCCCGGTACCATGGA
TGCCGTCCGCGCCGGTCCTTTCGGTCAGCTCTTCCGC?????????????????????????????????
>P_photinicola_GZCC_16_0028
????GACATTATAGAGTTTTCTACACTCCCAACCCATGTGAACTTACC-
ATTGTTGCCTCGGCAGAAGCTGCTCGGT-
GCACCCTACCTTGTTACGACCTTTACTTCCGCGCCTTACCCTGGAACGGCTTACCCTGTAGCGGC
TGCCGGTGGACTACCAAACTCTTGTTATTTTATTGTAATCTGAGCGTCTTATTTTAATAAGTCAAA
ACTTTCAACAACGGATCTCTTGGTTCTGGCATCGATGAAGAACGCAGCGAAATGCGATAAGTAA
TGTGAATTGCAGAATTCAGTGAATCATCGAATCTTTGAACGCACATTGCGCCCATTAGTATTCTA
GTGGGCATGCCTGTTCGAGCGTCATTTCAACCCTTAAGCCTAGCTTAGTGTTGGGAGCCTACTG-
-CTTTTACTAGCTGTAGCTCCTGAAATACAACGGCGGATCTGCGATATCCTCTGAGCGTAGTAA-
TTTTTATCTCGCTTTTGACTGGAGTTGCAGCGTCTTTAGCCGCTAAATCCCCC-AA-
TTTTTAATGGTTGACCTCAGGTTAGTCATCCTCGCAATCCC-ATCATCC------TCATC---------
--CTCATCATCACCT--CGCAAA----CA-TTTCCACACC-GGTGCCGAAAATCTGG--------
TTTTCGCACCTGCC-CATTTTCCCAG--ACACTTACCCC--------
GCCGCACGACCCCGCGGTGCAAACGAAAAATTTCTTA--
TCACAGCCCCACATCACACAAACATTTTGGCAGCCACGCACTTTGCATGACCCACAATGAACAA
TTGCTGACCCCGCCAAATAGGAAGCCGCCGAGCTCGGAAAGGGTTCCTTCAAGTACGCATGGG
TTCTTGACAAGCTCAAGGCCGAGCGTGAGCGTGGTATCACCATTGATATCGCTCTCTGGAAGTT
CGAGACCAACGAGTACAATGTCACCGTCATTGGTTAGTATCCCTGCCCACAACATGTGTCATGT
CTCTGAACTCAAGACTAACCTTGCAATACAGACGCTCCCGGTCACCGTGATTTCATCAATGCTG
CCTTCTGGTATGTAGCC-CATCTACCTCGACGC-
GCCTCAATACGACACCCCCCGGCAACTCGACAAC--GACATTCTCAAC---
TGCTTGTTTGGAACCATACGAAAGACTTGATACTGACCGGTCTATGATAGGCAAACCATCTCTG
GCGAGCACGGTCTCGACAGCAATGGAGTGTACGTACCCTTTCCCTGGCTACTCGCTTTCTCGTG
AACATGTCAGCTAACAGTTG-
TGCTTGTTCAGCTACAACGGTACCTCCGAGCTCCAGCTCGAGCGCATGAGCGTCTACTTCAACG
AGGCTTCCGGCAACAAGTACGTTCCTCGTGCCGTCCTCGTCGATCTCGAGCCCGGTACCATGGA
TGCCGTCCGCGCCGGTCCTTTCGGTCAGCTCTTCCGCCCTGACAACTTCGTCT-CGGTCA-
TCCGGCG??
>P_pinisp_CBS_146841
AGGGATCATTATAGAGTTTTCTAAACTCCCAACCCATGTGAACTTACC-
ATTGTTGCCTCGGCAGAAGCTGCTCGGT-
GCACCTTACCTTGGAACGGCCTACCCTGTAGCGCCTTACCCTGGAACGGCTTACCCTGTAGCGG
CTGCCGGTGGACTACCAAACTCTTGTTATTTTATTGTAATCTGAGCGTCTTATTTTAATAAGTCAA
AACTTTCAACAACGGATCTCTTGGTTCTGGCATCGATGAAGAACGCAGCGAAATGCGATAAGTA
ATGTGAATTGCAGAATTCAGTGAATCATCGAATCTTTGAACGCACATTGCGCCCATTAGTATTCT
AGTGGGCATGCCTGTTCGAGCGTCATTTCAACCCTTAAGCCTAGCTTAGTGTTGGGAGCCTACT
G--
CTTTTGCTAGCTGTAGCTCCTGAAATACAACGGCGGATCTGCGATATCCTCTGAGCGTAGTAA-
TTTTTATCTCGCTTTTGACTGGAGTTGCAGCGTCTTTAGCCGCTAAATCCCCC-AA-
TTTTTAATGGTTGACCTC??????????TCCTCACAATCCC-GTCATCT------CCATT--------

CTCATCATCATCACCT--CGCAAA----CA-TTTTCCAACC-GGTGCCGAAAATCT-G--------
CTTTCGCACCTGCC-CATTTTCCCAG--ACACTTACCCC--------
GCCGCACGACCCCGCGGTGCAAACGAAAAATTTCTTA--
TCACAGCCCCACATCGCACAAACATTTTGGCAGCGATGCACTTTCCATGACCCACAATGAACAA
TTGCTGACCCCGCCAAATAGGAAGCCGCCGAGCTCGGAAAGGGTTCCTTCAAGTACGCATGGG
TTCTTGACAAGCTCAAGGCCGAGCGTGAGCGTGGTATCACCATTGATATCGCTCTCTGGAAGTT
CGAGACCAACGAGTACAATGTCACCGTCATTGGTTAGTATCCCTGTCCACACCATGTAACATGC
AACCGATCTT-
GTACTAACATGGCAATACAGATGCTCCCGGTCACCGTGATTTCATCAATGCTGCCTTCTGGTACG
TAGTC-CATCTACCTCGACAC-GCCTCAATACGACAACCCTCCGCAACTCGACAAC--
GACCTTCTCAACAACTGCTTGGTTGGAACCAAAGAAAAGACCTGATACTGACCGGTCTCTGATA
GGCAAACCATCTCTGGCGAGCACGGTCTCGACAGCAATGGAGTGTACGTACCCTTTCCTTGGCT
ACTTGCTTTCCCACGAACATCTCAGCTAATACCCG-
TGATTGTTCAGCTACAACGGTACCTCTGAGCTCCAGCTCGAGCGCATGAGCGTCTACTTCAACG
AGGCTTCCGGCAACAAGTACGTTCCTCGTGCCGTCCTCGTCGATCTCGAGCCCGGTACCATGGA
TGCCGTCCGCGCCGGTCCTTTCGGTCAGCTCTTCCGCCCTGACAACTTTGTCTTCGGTCAGTCCG
GTGCC
>P_portugalica_CBS_393_48
AGGGATCATTATAGAGTTTTCTAAACTCCCAACCCATGTGAACTTACC-
ATTGTTGCCTCGGCAGAAGCTACCCGGT---
ACCTTACCTTGGAACGGCCTACCCTGTAGCGCCTTACCCTGGAACGGCTTACCCTGTAACGGCT
GCCGGTGGACTACCAAACTCTTGTTATTTTATTGTTATCTGAGCGTCTTATTTTAATAAGTCAAAA
CTTTCAACAACGGATCTCTTGGTTCTGGCATCGATGAAGAACGCAGCGAAATGCGATAAGTAAT
GTGAATTGCAGAATTCAGTGAATCATCGAATCTTTGAACGCACATTGCGCCCATTAGTATTCTAG
TGGGCATGCCTGTTCGAGCGTCATTTCAACCCTTAAGCCTAGCTTAGTGTTGGGAGCCTACTG--
CTTTTGCTAGCTGTAGCTCCTGAAATACAACGGCGGATCTGCGATATCCTCTGAGCGTAGTAATT
TTTTTTCTCGCTTTTGACTGGAGTTGCAGCGTCTTTAGCCGCTAAATCCCCC-AA-
TTTTTAATGGTTGACCTCAGGTTAGTCATCCTCGCAATCTC-ATCATGCCCACCCCCATC-------
-ATCATCATCACCACCT--CGCAAA----CA-TCTTCAACTT-GGTGCCGAAAATCT-G--------
TTTTCGCACCTGCC-CATGTTCCCAG--ACACTTACCCC--------
GCCGCACGACCCCGCGGTGCAAACGAAAAAATTCTTA--
TCACAGCCCCACATCGCACAAACATTTTGGCAGCCATGCACTTTTCATGACCCACATTGAGCATT
TGCTGACCCCGCCAAATAGGAAGCCGCCGAGCTCGGAAAGGGTTCCTTCAAGTACGCATGGGT
TCTTGACAAGCTCAAGGCCGAGCGTGAGCGTGGTATCACCATTGATATCGCTCTCTGGAAGTTC
GAGACCAACGAGTACAATGTCACCGTCATTGGTTAGTATCCCTGTCCACAACATTGATCATGCCT
CGGAACTC-
AGACTAACATGGTAATACAGATGCTCCCGGTCACCGTGACTTCATCAATGCTGCCTTTTGGTATG
TAGCC-CATCTACCTCGACAC-GCCTCAATACGACG-CCTCCCGCAGCTCGACCAC--
GACGGCCTCAAC---
TATTTGGTTGGAACCAAACAAAAGACTTGATACTGACCGGTCTCTGATAGGCAAACCATCTCTG
GCGAGCACGGTCTCGACAGCAATGGAGTGTACGTACCCTTGCCTTCTCCATTTGCCTTCCCACG
AACATGTTAGCTAACACCCG-
TGCTTGCTCAGCTACAACGGTACCTCCGAGCTCCAGCTCGAGCGCATGAGCGTCTACTTCAACG
AGGCTTCCGGCAACAAGTACGTTCCTCGTGCCGTCCTCGTCGATCTCGAGCCCGGTACCATGGA

TGCCGTCCGCGCCGGTCCTTTCGGTCAGCTCTTCCGCCCTGACAACTTCGTCTTCGGTCAGTCCG
GTGCC
>P_rhizophorae_MFLUCC_17_0416
AGGGATCATTATAGAGTTTTCTAAACTCCCAACCCATGTGAACTTACC-
ATTGTTGCCTCGGCAGAAGCTGCTCGGT-
GCACCTTACCTTGGAACGGCCTACCCTGTAGCGCCTTACCCTGGAACGGCTTACCCTGCAGCGG
CTGCCGGTGGACTACCAAACTCTTGTTATTTTATTGTAATCTGAGCGTCTTATTTTAATAAGTCAA
AACTTTCAACAACGGATCTCTTGGTTCTGGCATCGATGAAGAACGCAGCGAAATGCGATAAGTA
ATGTGAATTGCAGAATTCAGTGAATCATCGAATCTTTGAACGCACATTGCGCCCATTAGTATTCT
AGTGGGCATGCCTGTTCGAGCGTCATTTCAACCCTTAAGCCTAGCTTAGTGTTGGGAGCCTACT
G--
CTTTTGCTAGCTGTAGCTCCTGAAATACAACGGCGGATCTGCGATATCCTCTGAGCGTAGTAA-
TTTTTATCTCGCTTTTGACTGGAGTTGCAGCGTCTTTAGCCGCTAAACCCCCC-AA-
TTTTTAATGGTTGACCTC???TTAGTCATCCTCGCAATCCC-ATCATCC------TCATC--------
TTCATCACCATCACCT--CGCAAA----CA-TTTCCACACC-GGTGCCGAAAATCT-G--------
TTTTCGCACCTGCC-CATTTTCCCAG--ACACTTACCCC--------
GCCGCACGACCCCGCGGTGCAAACGAAAAATTTCTTA--TCATAGCCCCACATCAC--
AAACATTTTGGCAGCCACGCACTCTGCATGACCCACAATGAACAATTGCTGACCCCGCCAAATA
GGAAGCCGCCGAGCTCGGAAAGGGTTCCTTCAAGTACGCATGGGTTCTTGACAAGCTCAAGGC
CGAGCGTGAGCGTGGTATCACCATTGATATCGCTCTCTGGAAGTTCGAGACCAACGAGTACAAT
GTCACCGTCATTGGTTAGTATCCCTGCCCACAACATGTGTCATGTCTCCGAACTCGAGACTAACC
TTACAATACAGACGCTCCCGGTCACCGTGATTTCATCAATGCTGCCTTCTGGTATGTAGCC-
CATCTACCCCGACGC-GTCTCAATACGACA-CCCCCGGCAACTCGACAAC--GACGTTCTCAAC-
--
TGCTTGGTTGAAACCAAATGAAAGACTTGATACTGACCGGTCTCTGATAGGCAAACCATCTCTG
GCGAGCACGGTCTCGACAGCAATGGAGTGTACGTACCCTTTCCCTGGCTACTCGCTTTCTCGTG
AACATGTCAGCTAACACTCG-
TGCTTGTTCAGCTACAACGGTACCTCCGAGCTCCAGCTCGAGCGCATGAGCGTCTACTTCAACG
AGGCTTCCGGCAACAAGTACGTTCCTCGTGCCGTCCTCGTCGATCTCGAGCCCGGTACCATGGA
TGCCGTCCGCGCCGGTCCTTTCGGTCAGCTCTTCCGCCCTGACAACTTCGTCTTCGGTCAGTCCG
GTGCC
>P_rhizophorae_MFLUCC_17_0417
AGGGATCATTATAGAGTTTTCTAAACTCCCAACCCATGTGAACTTACC-
ATTGTTGCCTCGGCAGAAGCTGCTCGGT-
GCACCTTACCTTGGAACGGCCTACCCTGTAGCGCCTTACCCTGGAACGGCTTACCCTGCAGCGG
CTGCCGGTGGACTACCAAACTCTTGTTATTTTATTGTAATCTGAGCGTCTTATTTTAATAAGTCAA
AACTTTCAACAACGGATCTCTTGGTTCTGGCATCGATGAAGAACGCAGCGAAATGCGATAAGTA
ATGTGAATTGCAGAATTCAGTGAATCATCGAATCTTTGAACGCACATTGCGCCCATTAGTATTCT
AGTGGGCATGCCTGTTCGAGCGTCATTTCAACCCTTAAGCCTAGCTTAGTGTTGGGAGCCTACT
G--
CTTTTGCTAGCTGTAGCTCCTGAAATACAACGGCGGATCTGCGATATCCTCTGAGCGTAGTAA-
TTTTTATCTCGCTTTTGACTGGAGTTGCAGCGTCTTTAGCCGCTAAACCCCCC-AA-
TTTTTAATGGTTGACCTC???TTAGTCATCCTCGCAATCCC-ATCATCC------TCATC--------
TTCATCACCATCACCT--CGCAAA----CA-TTTCCACACC-GGTGCCGAAAATCT-G--------

TTTTCGCACCTGCC-CATTTTCCCAG--ACACTTACCCC--------
GCCGCACGACCCCGCGGTGCAAACGAAAAATTTCTTA--TCATAGCCCCACATCAC--
AAACATTTTGGCAGCCACGCACTCTGCATGACCCACAATGAACAATTGCTGACCCCGCCAAATA
GGAAGCCGCCGAGCTCGGAAAGGGTTCCTTCAAGTACGCATGGGTTCTTGACAAGCTCAAGGC
CGAGCGTGAGCGTGGTATCACCATTGATATCGCTCTCTGGAAGTTCGAGACCAACGAGTACAAT
GTCACCGTCATTGGTTAGTATCCCTGCCCACAACATGTGTCATGTCTCCGAACTCGAGACTAACC
TTACAATACAGACGCTCCCGGTCACCGTGATTTCATCAATGCTGCCTTCTGGTATGTAGCC-
CATCTACCCCGACGC-GTCTCAATACGACA-CCCCCGGCAACTCGACAAC--GACGTTCTCAAC-
--
TGCTTGGTTGAAACCAAATGAAAGACTTGATACTGACCGGTCTCTGATAGGCAAACCATCTCTG
GCGAGCACGGTCTCGACAGCAATGGAGTGTACGTACCCTTTCCCTGGCTACTCGCTTTCTCGTG
AACATGTCAGCTAACACTCG-
TGCTTGTTCAGCTACAACGGTACCTCCGAGCTCCAGCTCGAGCGCATGAGCGTCTACTTCAACG
AGGCTTCCGGCAACAAGTACGTTCCTCGTGCCGTCCTCGTCGATCTCGAGCCCGGTACCATGGA
TGCCGTCCGCGCCGGTCCTTTCGGTCAGCTCTTCCGCCCTGACAACTTCGTCTTCGGTCAGTCCG
GTGCC
>P_rhododendri_IFRDCC_2399
??????CATTATAGAGTTTTCTAAACTCCCAACCCATGTGAACTTACC-
ATTGTTGCCTCGGCAGAAGCTGCTCGGT-
GCACCTTACCCTGGAACGGCCTACCCTGTAGCGCCTTACCCTGGAACGGCTTACCCTGTAGCGG
CTGCCGGTGGACTACCAAACTCTTGTTATTTTATTGTAATCTGAGCGTCTTATTTTAATAAGTCAA
AACTTTCAACAACGGATCTCTTGGTTCTGGCATCGATGAAGAACGCAGCGAAATGCGATAAGTA
ATGTGAATTGCAGAATTCAGTGAATCATCGAATCTTTGAACGCACATTGCGCCCATTAGTATTCT
AGTGGGCATGCCTGTTCGAGCGTCATTTCAACCCTTAAGCCTAGCTTAGTGTTGGGAGCCTACT
G--
CTTTTGCTAGCTGTAGCTCCTGAAATACAACGGCGGATCTGCGATATCCTCTGAGCGTAGTAA-
TTTTTATCTCGCTTTTGACTGGAGTTGCAGCGTCTTTAGCCGCTAAATCCCCT-AA-
TTTTTAATGGTTGACCTCACGTTAGTCATCCTCACAATCCC-ATCATCC--------------------
CCATCATCATCGTCT--CGCAAA----CA-TTTTCCAACT-GGTGCCGAAAATCT-G--------
TTTTCGCACCTGCC-CATTTTCCCAG--ACACTTACCCC--------
GCCGCACGACCCCGCGGTGCAAACGAAAAATTTCTTA--
TCACAGCCCCACATCGCACAAACATTTTGGCAGCCATGCACTTTCCATGACCCACAATGAACAA
TTGCTGACCCCGCCAAATAGGAAGCCGCCGAGCTCGGAAAGGGTTCCTTCAAGTACGCATGGG
TTCTTGACAAGCTCAAGGCCGAGCGTGAGCGTGGTATCACCATTGATATCGCTCTCTGGAAGTT
CGAGACCAACGAGTACAATGTCACCGTCATTGGTTAGTATCCCGGCCCACACCATGTACCATGC
ATCCGACCTT-
GTACTAACATCACAATACAGATGCTCCCGGTCACCGTGATTTCATCAATGCTGCTTTCTGGTACG
TAGCCATATCTACCTCGACAC-GCCTCGATACGACAACCCTCCGCAACTCGACAAC--
GACCTTCTCAACAACTGCTTGGTTGGAACCAAAGAAAAGACCTGATACTGACCGGTCTCTGATA
GGCAAACCATCTCTGGCGAGCACGGTCTCGACAGCAATGGAGTGTACGTACCCTTTCCTTGGCT
ACTTGCTTTCCCACGAACATCTCAGCTAATACCCG-
TGATTGTTCAGCTACAACGGTACCTCTGAGCTCCAGCTCGAGCGCATGAGCGTCTACTTCAACG
AGGCTTCCGGCAACAAGTACGTTCCTCGTGCCGTCCTCGTCGATCTCGAGCCCGGTACCATGGA
TGCCGTCCGCGCCGGTCCTTTCGGTCAGCTCTTCCGCCCTGACAACTTTGTCTTCGGTCAGTCCG

GTGCC
>P_rhodomyrtus_HGUP4230
AGGGATCATTATAGAGTTTTCTAAACTCCCAACCCATGTGAACTTACC-
ATTGTTGCCTCGGCAGAAGCTGCTCGGT-
GCACCCTACCTTGGAACGGCCTACCCTGTAGCGCCTTACCCTGGAACGGCTTACCCTGTAACGG
CTGCCGGTGGACTACCAAACTCTTGTTATTTTATTGTAATCTGAGCGTCTTATTTTAATAAGTCAA
AACTTTCAACAACGGATCTCTTGGTTCTGGCATCGATGAAGAACGCAGCGAAATGCGATAAGTA
ATGTGAATTGCAGAATTCAGTGAATCATCGAATCTTTGAACGCACATTGCGCCCATTAGTATTCT
AGTGGGCATGCCTGTTCGAGCGTCATTTCAACCCTTAAGCCTAGCTTAGTGTTGGGAGCCTACT
G--
CTTTTGCTAGCTGTAGCTCCTGAAATACAACGGCGGATCTGCGATATCCTCTGAGCGTAGTAAAT
TTTTATCTCGCTTTTGACTGGAGTTGCAGCGTCTTTGGCCGCTAAATCCCCC-AA-
TTTTTAATGGTTGACCTCAGGTTAGTCATCCTCGCAATCCC-ATCATCC------TCATC--------
CTCATCATCATCACCT--CGCAAA----CA-TTTCCACACC-GGTGCCGAAAATCTGG--------
TTTTCGCACCTGCC-CATTTTCCCAG--ACACTTACCCC--------
GCCGCACGACCCCGCGGTGCAAACGAAAAATTTCTTA--
TCACAGCCCCACATCACACAAACATTTTGGCAGCCACGCACTTTGCATGACCCACAATGAACAA
TTGCTGACCCCGCCAAATAGGAAGCCGCCGAGCTCGGAAAGGGTTCCTTCAAGTACGCATGGG
TTCTTGACAAGCTCAAGGCCGAGCGTGAGCGTGGTATCACCATTGATATCGCTCTCTGGAAGTT
CGAGACCAACGAGTACAATGTCACCGTCATTGGTTAGTATCCCTGCCCACAACATGTGTCATGT
CTCTGAACTCAAGACTAACCTTGCAATACAGACGCTCCCGGTCACCGTGATTTCATCAA????????
???GGGGGGTAGGC--ATCTACCTCGACGC-GCCTC-ATACGACA-
CCCCCGGCAACTCGACCAC--GATAATCTCAAC---
TGCTTGGTTGGAACCATACGAAAGACTCGATACTGACCGGTCTATGATAGGCAAACCATCTCTG
GCGAGCACGGTCTCGACAGCAATGGAGTGTACGTACCCTTTCCCTGGCTGTTCGCTTTCTCGTG
AACATGTCAGCTAACAGTCG-
TGCTTGTTCAGCTACAACGGTACCTCCGAGCTCCAGCTCGAGCGCATGAGCGTCTACTTCAACG
AGGCTTCCGGCAACAAGTACGTTCCTCGTGCCGTCCTCGTCGATCTCGAGCCCGGTACCATGGA
TGCCGTCCGCGCCGGTCCTTTCGGTCAGCTCTTCCGCCCTGACAACTTCGTCTTCGGTCAGTCCG
GTGCC
>P_rhodomyrtus_MG7
AGGGATCATTATAGAGTTTTCTAAACTCCCAACCCATGTGAACTTACC-
ATTGTTGCCTCGGCAGAAGCTGCTCGGT-
GCACCCTACCTTGGAACGGCCTACCCTGTAGCGCCTTACCCTGGAACGGCTTACCCTGTAACGG
CTGCCGGTGGACTACCAAACTCTTGTTATTTTATTGTAATCTGAGCGTCTTATTTTAATAAGTCAA
AACTTTCAACAACGGATCTCTTGGTTCTGGCATCGATGAAGAACGCAGCGAAATGCGATAAGTA
ATGTGAATTGCAGAATTCAGTGAATCATCGAATCTTTGAACGCACATTGCGCCCATTAGTATTCT
AGTGGGCATGCCTGTTCGAGCGTCATTTCAACCCTTAAGCCTAGCTTAGTGTTGGGAGCCTACT
G--
CTTTTGCTAGCTGTAGCTCCTGAAATACAACGGCGGATCTGCGATATCCTCTGAGCGTAGTAAAT
TTTTATCTCGCTTTTGACTGGAGTTGCAGCGTCTTTGGCCGCTAAATCCCCC-AA-
TTTTTAATGGTTGACCTC???TCATCATCATC
ACCT--CGCAAA----CA-TTTCCACACC-GGTGCCGAAAATCTGG--------
TTTTCGCACCTGCC-CATTTTCCCAG--ACACTTACCCC--------

GCCGCACGACCCCGCGGTGCAAACGAAAAATTTCTTA--
TCACAGCCCCACATCACACAAACATTTTGGCAGCCACGCACTTTGCATGACCCACAATGAACAA
TTGCTGACCCCGCCAAATAGGAAGCCGCCGAGCTCGGAAAGGGTTCCTTCAAGTACGCATGGG
TTCTTGACAAGCTCAAGGCCGAGCGTGAGCGTGGTATCACCATTGATATCGCTCTCTGGAAGTT
CGAGACCAACGAGTACAATGTCACCGTCATTGGTTAGTATCCCTGCCCACAACATGTGTCATGT
CTCTGAACTCAAGACTAACCTTGCAATACAGACGCTCCCGGTCACCGTGATTTCATCAA??CTGC
TTTCTGGTATGTAGCC-CATCTACCTCGACGC-GCCTCAATACGACA-
CCCCCGGCAACTCGACCAC--GATAATCTCAAC---
TGCTTGGTTGGAACCATACGAAAGACTCGATACTGACCGGTCTATGATAGGCAAACCATCTCTG
GCGAGCACGGTCTCGACAGCAATGGAGTGTACGTACCCTTTCCCTGGCTGTTCGCTTTCTCGTG
AACATGTCAGCTAACAGTCG-
TGCTTGTTCAGCTACAACGGTACCTCCGAGCTCCAGCTCGAGCGCATGAGCGTCTACTTCAACG
AGGCTTCCGGCAACAAGTACGTTCCTCGTGCCGTCCTCGTCGATCTCGAGCCCGGTACCATGGA
TGCCGTCCGCGCCGGTCCTTTCGGTCAGCTCTTCCGCCCTGACAACTTCGTCTTCGGTCAGTCCG
GTGCC
>P_rosea_MFLUCC_12_0258
??????CATTATAGAGTTTTCTAAACTCCCAACCCATGTGAACTTACC-
ATTGTTGCCTCGGCAGAAGCTGCTCGGT-
ACACCTTACCTTGGAACGGCCTACCCTGTAGCGCCTTACCCTGGAACGGCTTACCCTGTAACGG
CTGCCGGTGGACTACCAAACTCTTGTTATTTTATTGTAATCTGAGCGTCTTATTTTAATAAGTCAA
AACTTTCAACAACGGATCTCTTGGTTCTGGCATCGATGAAGAACGCAGCGAAATGCGATAAGTA
ATGTGAATTGCAGAATTCAGTGAATCATCGAATCTTTGAACGCACATTGCGCCCATTAGTATTCT
AGTGGGCATGCCTGTTCGAGCGTCATTTCAACCCTTAAGCCTAGCTTAGTGTTGGGAGCCTACT
G--
CTTTTACTAGCTGTAGCTCCTGAAATACAACGGCGGATCTGCGATATCCTCTGAGCGTAGTAA-
TTTTTATCTCGCTTTTGACTGGAGTTGCAGCGTCTTTAGCCGCTAAATCCCCC-AA-
TTTTTAATGGTTGACCTCAGGTTAGTCATCCTCGCATTCCC-ATCATCC-------------------
TCATCCTCATCACCT--CGCAAA----CA-TTTCCACACT-GGTGCCGAAAATCTGG--------
TTTTCGCACCTGCC-CATTTTCCCAG--ACACTTACCCC--------
GCCGCACGACCCCGCGGTGCAAACGAAAAATTTCTTA--
TCACAGCCCCACATCACACAAACATTTTGGCAGCCACGCACTTTGCATGACCCACAATGAACAA
TTGCTGACCCCGCTAAATAGGAAGCCGCCGAGCTCGGAAAGGGTTCCTTCAAGTACGCATGGG
TTCTTGACAAGCTCAAGGCCGAGCGTGAGCGTGGTATCACCATTGATATCGCTCTCTGGAAGTT
CGAGACCAACGAGTACAATGTCACCGTCATTGGTTAGTATCCCTGCCCACACCATGCATCATGT
CTTCGAACTCAAGACTAACCTTGCAATACAGACGCTCCCGGTCACCGTGATTTCATCAATGCTG
CTTTCTGGTATGTAGCC-CATCTACCTCGACGC-GCCTCAATACGACA-
CCACCGGCAACTCGACAAC--GACATTCTCAAC---
TGCTTGGTTGAAACCAAAAGAAAGACCTGATACTGACCGGTCTTTGATAGGCAAACCATCTCTG
GCGAGCACGGTCTCGACAGCAATGGAGTGTACGTACCCTTTCCCTGGCTACTCGTTTTTCCACG
AACATGTCAGCTAACAGTCG-
TGCTTGTTCAGCTACAACGGTACCTCCGAGCTCCAGCTCGAGCGCATGAGCGTCTACTTCAACG
AGGCTTCCGGTAACAAGTACGTTCCTCGTGCCGTCCTCGTCGATCTCGAGCCCGGTACCATGGA
TGCCGTCCGCGCCGGTCCTTTCGGTCAGCTCTTCCGCCCTGACAACTTCGTCTTCGGCCAGTCCG
GTGCC

>P_scoparia_CBS_176_25
AGGGATCATTATAGAGTTTTCTAAACTCCCAACCCATGTGAACTTACC-
ATTGTTGCCTCGGCAGAAGCTACCTGGT-
TTACCTTACCTTGGAACGGCCTACCCTGTAGCGCCTTACCCTGGAACGGCCTACCCTGTAACGG
CTGCCGGTGGACTACCAAACTCTTGTTATTTTATTGTAATCTGAGCGTCTTATTTTAATAAGTCAA
AACTTTCAACAACGGATCTCTTGGTTCTGGCATCGATGAAGAACGCAGCGAAATGCGATAAGTA
ATGTGAATTGCAGAATTCAGTGAATCATCGAATCTTTGAACGCACATTGCGCCCATTAGTATTCT
AGTGGGCATGCCTGTTCGAGCGTCATTTCAACCCTTAAGCCTAGCTTAGTGTTGGGAGCCTACT
G--
CTTTTGCTAGCGGTAGCTCCTGAAATACAACGGCGGATCTGCGATATCCTCTGAGCGTAGTAA-
TTTTTATCTCGCTTTTGACTGGAGTTGCAGCGTCTTTAGCCGCTAAACCCCCC-AA-
TTTTTAATGGTTGACCTCAGGTTAGTCATCCTCACAATGCC-ATCATTC------CCATC---------
--CTCCTCATCGCCT--CGCAAA----CA---TTCCAACC-GGTGCCGAAAATCT-G--------
TTTTCGCACCTGCC-CATTTTCCCAG--ACACTTACCCC--------
GCCGCACGACCCCGCGGTGCAAACGAAAAATTTCTTA--
TCACAGCCCCACATCACACAAACATTTTCGCAGCCATGCACTTTCCAGGACCCACAATGAACAA
TTGCTGACCCCGCCAAATAGGAAGCCGCCGAGCTCGGAAAGGGTTCCTTCAAGTACGCATGGG
TTCTTGACAAGCTCAAGGCCGAGCGTGAGCGTGGTATCACCATTGATATCGCTCTCTGGAAGTT
CGAGACCAACGAGTACAATGTCACCGTCATTGGTTAGTCACCCTGTCCATGCGATGTACCATGC
ATCTGAATGT-
ATGCTAACATGGCAACACAGATGCTCCCGGTCACCGTGATTTCATCAATGCTGCCTTTTGGTATG
TAGCC-CATCTACCTCGACAC-GCCTCAATACGACAACCCCCCGCAACTCGACAAC--
GACGTTCTCAACAAGTGCTTGGTTGGAAACAAGGGAAAGACTTGACACTGACCGGTCCCTGATA
GGCAAACCATCTCTGGCGAGCACGGTCTCGACAGCAATGGAGTGTACGTACCCTTTCCTTGGCT
ACTTGCTTTCCCACGAACATCTCAGCTAACACTCG-
TGGTTTTTCAGCTACAACGGTACCTCCGAGCTCCAGCTCGAGCGCATGAGCGTCTACTTCAACG
AGGCTTCCGGCAACAAGTACGTTCCTCGTGCCGTCCTCGTCGATCTCGAGCCCGGTACCATGGA
TGCCGTCCGCGCCGGTCCTTTCGGTCAGCTCTTCCGCCCTGACAACTTCGTCTTCGGTCAGTCCG
GTGCC
>P_sequoiae_MFLUCC_13_0399
AGGGATCATTATAGAGTTTTCTAAACTCCCAACCCATGTGAACTTACC-
ATTGTTGCCTCGGCAGAAGCTACCTGGT--
TACCCTACCTTGGAACGGCCTACCCTGTAGCGCCTTACCCTGGAACGGCCTACCCTGTAACGGC
TGCCGGTGGACTACCAAACTCTTGTTATTTTATTGTAATCTGAGCGTCTTATTTTAATAAGTCAAA
ACTTTCAACAACGGATCTCTTGGTTCTGGCATCGATGAAGAACGCAGCGAAATGCGATAAGTAA
TGTGAATTGCAGAATTCAGTGAATCATCGAATCTTTGAACGCACATTGCGCCCATTAGTATTCTA
GTGGGCATGCCTGTTCGAGCGTCATTTCAACCCTTAAGCCTAGCTTAGTGTTGGGAGCCTACCG-
-CTTTTGCTAGCGGTAGCTCCTGAAATACAACGGCGGATCTGCGATATCCTCTGAGCGTAGTAA-
TTTTTATCTCGCTTTTGACTGGAGTTGCAGCGTCTTTAGCCGCTAAACCCCCCAAA????????????
??
??
??
??
??

??
??
??
??
??
???
>P_shandogensis_JZB340038
AGGGATCATTATAGAGTTTTCTAAACTCCCAACCCATGTGAACTTACC-
ATTGTTGCCTCGGCAGAAGCTGCTCGGT-
GCACCCTACCTTGGAACGGCCTACCCTGTAGCGCCTTACCCTGGAACGGCTTACCCTGTAGCGG
CTGCCGGTGGACTACCAAACTCTTGTTATTTTATTGTAATCTGAGCGTCTTATTTTAATAAGTCAA
AACTTTCAACAACGGATCTCTTGGTTCTGGCATCGATGAAGAACGCAGCGAAATGCGATAAGTA
ATGTGAATTGCAGAATTCAGTGAATCATCGAATCTTTGAACGCACATTGCGCCCATTAGTATTCT
AGTGGGCATGCCTGTTCGAGCGTCATTTCAACCCTTAAGCCTAGCTTAGTGTTGGGAGCCTACT
G--
CTTTTACTAGCTGTAGCTCCTGAAATACAACGGCGGATCTGCGATATCCTCTGAGCGTAGTAA-
TTTTTATCTCGCTTTTGACTGGAGTTGCAGCGTCTTTAGCCGCTAAATCCCCC-AA-
TTTTTAATGGT-GACCTC?????????????????????????ATCATCC------TCATC--------
CTCATCATCATCACCT--CGCAAA----CA-TTTCCACACC-GGTGCCGAAAATCTGG--------
TTTTCGCACCTGCC-CATTTTCCCAG--ACACTTACCCC--------
GCCGCACGACCCCGCGGTGCAAACGAAAAATTTCTTA--
TCACAGCCCCACATCACACAAACATTTTGGCAGCCACGCACTTTGCATGACCCACAATGAACAA
TTGCTGACCCCGCCAAATAGGAAGCCGCCGAGCTCGGAAAGGGTTCCTTCAAGTACGCATGGG
TTCTTGACAAGCTCAAGGCCGAGCGTGAGCGTGGTATCACCATTGATATCGCTCTCTGGAAGTT
CGAGACCAACGAGTACAATGTCACCGTCATTGGTTAGTATCCCTGCCCACAACATGTGTCATGT
CTCTGAACTCAAGACTAACCTTGCAATACAGACGCTCCCGGTCACCGTGATTTCATCAATGCTG
CCTTCTGGTATGTAGCC-CATCTACCTCGACGC-GCCTCAATACGACA-
CCCCCGGCAACTCGACAAC--GACATTCTCAAC---
TGCTTGTTTGGAACCATACGAAAGACTTGATACTGACCGGTCTATGATAGGCAAACCATCTCTG
GCGAGCACGGTCTCGACAGCAATGGAGTGTACGTACCCTTTCCCTGGCTACTCGCTTTCTCGTG
AACATATCAGCTAACAGTCG-
TGCTTGTTCAGCTACAACGGTACCTCCGAGCTCCAGCTCGAGCGCATGAGCGTCTACTTCAACG
AGGCTTCCGGCAACAAGTACGTTCCTCGTGCCGTCCTCGTCGATCTCGAGCCCGGTACCATGGA
TGCCGTCCGCGCCGGTCCTTTCGGTCAGCTCTTCCGCCCTGACAACTTCGTCTTCGGCCAATCCG
GAA??
>P_shorea_MFLUCC_12_0314
AGGGATCATTATAGAGTTTTCTAAACTCCCAACCCATGTGAACTTACC-
ATTGTTGCCTCGGCAGAAGCTGCTCGGT-
GCACCTTACCTTGGAACGGCCTACCCTGTAGCGCCTTACCCTGGAACGGCTTACCCTGCAACGG
CTGCCGGTGGACTACCAAACTCTTGTTATTTTATGGTTATCTGAGCGTCTTATTTTAATAAGTCAA
AACTTTCAACAACGGATCTCTTGGTTCTGGCATCGATGAAGAACGCAGCGAAATGCGATAAGTA
ATGTGAATTGCAGAATTCAGTGAATCATCGAATCTTTGAACGCACATTGCGCCCATTAGTATTCT
AGTGGGCATGCCTGTTCGAGCGTCATTTCAACCCTTAAGCCTAGCTTAGTGTTGGGAGCCTACT
G--

CTTTTACTAGCTGTAGCTCCTGAAATACAACGGCGGATCTGCGATATCCTCTGAGCGTAGTAA-
TTTTTATCTCGCTTTTGACTGGAGTTGCAGCGTCTTTAGCCGCTAAACCCCC--AA-
TTTTTACTGGT-GACCTCAGGTTAGTCATCCTCGCGATCTC-ATCACCA------------------
CTCTCCCCTCATCACCT--CGCAAA----CA-ATTTCCAACA-GGTGCCGAAAATCT-G--------
TTTTCGCACCTGCC-CATTGTCCCAG--ACACTTACCCC--------
GCCGCACGACCCCGCGGTGCAAACGAAAAATTTCTTA--TCACAGCCCCACTTCACAC-
AACATTTTGGCAGCCACGCACTTTGCATGACCCACAA-
AAACAATTGCTGACCCCGCCAAACAGGAAGCCGCCGAGCTCGGAAAGGGTTCCTTCAAGTACG
CATGGGTTCTTGACAAGCTCAAGGCCGAGCGTGAGCGTGGTATCACCATTGATATCGCTCTCTG
GAAGTTCGAGACCAACGAGTACAATGTCACCGTCATTGGTTAGTATCACTGTCCACAACATGTA
TCATG--TCCGAACTC-
AGACTAACGTTATCATACAGACGCTCCCGGTCACCGTGATTTCATCAA???????????????????????
CATC-ACCTCGACAC-GCCTC-ATACGACA-CCCTCCGCAACTCGACGAC--GACATTCTCGGC-
--
TACTTGGTTGAAACCAAACGAAAAACTTGATACTGACCGGTCTCTGTTAGGCAAACCATCTCTG
GCGAGCACGGTCTCGACAGCAATGGAGTGTACGTACCATTTACTTGGCTCCTTGCTTTCCCACG
AACACGTTAGCTAACACTCG-
TGCTTGCTCAGCTACAACGGCACCTCCGAGCTCCAGCTCGAGCGCATGAGCGTCTACTTCAACG
AGGCTTCCGGCAACAAGTACGTTCCTCGTGCCGTCCTCGTCGATCTCGAGCCCGGTACCATGGA
CGCCGTCCGCGCCGGTCCTTTCGGCCAGCTCTTCCGCCCTGACAACTTCGTCTTCGGTCAGTCC
GGTGCC
>P_spathulata_CBS_356_86
AGGGATCATTATAGAGTTTTCTAAACTCCCAACCCATGTGAACTTACC-
ACTGTTGCCTCGGCAGAAGCTACCCGGT-
ATACCTTACCTTGGAACGGCCTACCCTGTAGCGCCTTACCCTGGAACGGCTTACCCTGTAGCGG
CTGCCGGTGGACTACTAAACTCTTGTTATTTTATTGTAATCTGAGCGTCTTATTTTAATAAGTCAA
AACTTTCAACAACGGATCTCTTGGTTCTGGCATCGATGAAGAACGCAGCGAAATGCGATAAGTA
ATGTGAATTGCAGAATTCAGTGAATCATCGAATCTTTGAACGCACATTGCGCCCATTAGTATTCT
AGTGGGCATGCCTGTTCGAGCGTCATTTCAACCCTTAAGCCTAGCTTAGTGTTGGGAGCCTACG
G--
CTTTTACTAGCTGTAGCTCCTGAAATACAACGGCGGATCTGCGATATCCTCTGAGCGTAGTAATT
TTTTTTCTCGCTTTTGACTGGAGTTGCAGCGTCTTTAGCCGCTAAACCCCCC-AA-
TTTTTAATGGTTGACCTCAGGTTAGTCATCCTCACGATCCC-AACATCA------TCACC--------
---ATCACTATCACCT--TGCACA----CA-TTTTCAACTC-GGTGCCGAAAATCA-G--------
TTTTCGCGCTTGCC-CACATTCCCAG--
ACACTTACCCCGCCGCGTGGCCGCACGACCCCGCGGTGCAAACGAAAAATTTCTTA--T--
CAGCCCCACATCGCACAAATATTTTGACAGCCATGCACTTTTCACAATCCACAATGAGCAATTG
CTGACCCCGCCAAATAGGAAGCCGCCGAGCTCGGTAAGGGTTCCTTCAAGTACGCATGGGTTC
TTGACAAGCTCAAGGCCGAGCGCGAGCGTGGTATCACCATCGATATCGCTCTCTGGAAGTTCGA
GACCAACGAGTACAATGTCACCGTCATTGGTCAGTATCCCTGTCCACCAGCTGTTTCATGCATCC
GAACTC-
GCACTAACATGGCAATATAGATGCTCCCGGTCACCGTGATTTCATCAATGCTGCCTTCTGGTATG
TAGCC-TATCTACCTCGACAC-GCCTCAATACGACG-CCCCCCGCAACTCGACGCC--
GACGTTCTCAAC---TACTTGGTTGGATCCAAACGGAAGACTTGATACTGACCG---

TCTCATAGGCAAACCATCTCTGGCGAGCACGGTCTCGACAGCAATGGAGTGTACGTGCCCTTTC
CTTGGCTACTTGCTTTCCCACGAACATACTAGCTAACCCTCG-
TGGTTGTTCAGCTACAACGGTACCTCCGAGCTCCAGCTCGAGCGCATGAGCGTCTACTTCAACG
AGGCTTCTGGCAACAAGTACGTTCCTCGTGCCGTCCTTGTCGATCTCGAGCCCGGAACCATGGA
TGCCGTCCGCGCCGGTCCTTTCGGTCAGCTCTTCCGCCCTGACAACTTCGTCTTCGGCCAGTCCG
GTGCC
>P_spathuliappendiculata_CBS_144035
???GATCATTATAGAGTTTTCTAAACTCCCAACCCATGTGAACTTACC-
ATTGTTGCCTCGGCAGAGGCTACCCGGT----
ACCTACCCTGGAACGGCCTACCCTGTAGCGCCTTACCCGGGAACGGACTACCCTGTAGCGGCT
GCCGGTGGACTACTCAACTCTTGTTATTTTATTGTTATCTGAGCGTCTTATTTTAATAAGTCAAAA
CTTTCAACAACGGATCTCTTGGTTCTGGCATCGATGAAGAACGCAGCGAAATGCGATAAGTAAT
GTGAATTGCAGAATTCAGTGAATCATCGAATCTTTGAACGCACATTGCGCCCATTAGTATTCTAG
TGGGCATGCCTGTTCGAGCGTCATTTCAACCCTTAAGCCTAGCTTAGTGTTGGGAGCCTACTG--
CTTTTACTAGCTGTAGCTCCTGAAATACAACGGCGGATCTGCGATATCCTCTGAGCGTAGTAA-
TTTTTTTCTCGCTTTTGACTGGAGTTGCAGCGTCTTTAGCCGCTAAACCCCCC-AA-
TTTTTAATGGT???????AGGTTAGTCATCCTCACAATGCC-ATCATCA--------------------
TCCCCATCATCACTC-CCCCAAA----CA-TCTTCACCTC-GGTGTCGAAAATCGAG---------
TTTCGCACCTGCC-CACATCCTCCG--
ACACTTACCCCGCCGCGTGGCCGCACGACCCCGCGGTGCAAACGAAAAATTTCTTA--
TCACAGCCCCACCTCGCACAAACATTTTGG-------------
TTCATGACCCACAATGATCAAATGCTGACCCCGCCAAACAGGAAGCCGCCGAGCTCGGTAAGG
GTTCCTTCAAGTACGCCTGGGTTCTTGACAAGCTCAAGGCCGAGCGTGAGCGTGGTATCACCAT
CGATATCGCTCTCTGGAAGTTCGAGACCAACGAGTACAATGTCACCGTCATTGGTTAGTATCCCT
GTCCACACCAAGCGTCATGCGTCCGATCTC-
AGACTAACTTGGCAA?????????????????????????????????????TGCTGCCTTCTGGTATGTAGCC-
CATCTACCTCGACAC-GCCTCGATACGAC-----------------------GACGGTCCCAAC---
TACTTGGTCGGAACCAAACCAAAGACTTGACACTGACCAGTCTTCGATAGGCAAACCATCTCTG
GCGAGCACGGTCTCGACAGCAATGGAGTGTACGTACCCTTTCCTTGGCTCCCTGCTTTCCCGTG
AACGTCTAGGCTAACACTCG-
CGGCTGTTCAGCTACAACGGTACCTCCGAGCTCCAGCTCGAGCGCATGAGCGTCTACTTCAACG
AGGCTTCCGGCAACAAGTACGTTCCTCGTGCCGTCCTCGTCGATCTCGAGCCCGGTACCATGGA
TGCCGTCCGCGCCGGTCCTTTCGGTCAGCTCTTCCGCCCTGACAACTTCGTCTTCGGTCAATCCG
GTGC?
>P_telopeae_CBS_114137
AGGGATCATTATAGAGTTTTCTAAACTCCCAACCCATGTGAACTTACC-
ATTGTTGCCTCGGCAGAAGCTGCTCGGT-
GCACCCTACCTTGGAACGGCCTACCCTGTAGCGCCTTACCCTGGAACGGCTTACCCTGTAACGG
CTGCCGGTGGACTACCAAACTCTTGTTATTTTATTGTAATCTGAGCGTCTTATTTTAATAAGTCAA
AACTTTCAACAACGGATCTCTTGGTTCTGGCATCGATGAAGAACGCAGCGAAATGCGATAAGTA
ATGTGAATTGCAGAATTCAGTGAATCATCGAATCTTTGAACGCACATTGCGCCCATTAGTATTCT
AGTGGGCATGCCTGTTCGAGCGTCATTTCAACCCTTAAGCCTAGCTTAGTGTTGGGAGCCTACT
G--
CTTTTGCTAGCTGTAGCTCCTGAAATACAACGGCGGATCTGCGATATCCTCTGAGCGTAGTAAAT

TTTTATCTCGCTTTTGACTGGAGTTGCAGCGTCTTTGGCCGCTAAATCCCCC-AA-
TTTTTAATGGTTGACCTC????TAGTCATCCTCGCAATCCCCATCATCC------TCATC--------
CTCATTATCACCACCT--CGCAAA----CA-TTTCCACACC-GGTGCCGAAAATCTGG--------
TTTTCGCACCTGCC-CATTTTCCCAG--ACACTTACCCC--------
GCCGCACGACCCCGCGGTGCAAACGAAAAATTTCTTA--
TCACAGCCCCACATCACACAAACATTTTGGCAGCCACGCACTTTGCATGACCCACAATGAACAA
TTGCTGACCCCGCCAAATAGGAAGCCGCCGAGCTCGGAAAGGGTTCCTTCAAGTACGCATGGG
TTCTTGACAAGCTCAAGGCCGAGCGTGAGCGTGGTATCACCATTGATATCGCTCTCTGGAAGTT
CGAGACCAACGAGTACAATGTCACCGTCATTGGTTAGTATCCCTGTCCACAACATGTGTCATGT
CTCTGAACTCAAGACTAACCTTGCAATACAGACGCTCCCGGTCACCGTGATTTCATCAATGCTG
CTTTCTGGTATGTAGCC-CATCTACCTCGACGC-
GCCTCAATACGACACCCCCCGGCAACTCGACAAC--GACATTCTTAAC---
TGCTTGTTTGGAACCATACAAAAGACTTGATACTGACCGGTCTATGATAGGCAAACCATCTCTG
GCGAGCACGGTCTCGACAGCAATGGAGTGTATGTACCCTTTCCCTGGCTATTCGCTTTCTCGTGA
ACATGTCAGCTAACAGTCG-
TGCTTGTTCAGCTACAACGGTACCTCCGAGCTCCAGCTCGAGCGCATGAGCGTCTACTTCAACG
AGGCTTCCGGCAACAAGTACGTTCCTCGTGCCGTCCTCGTCGATCTCGAGCCCGGTACCATGGA
TGCCGTCCGCGCCGGTCCTTTCGGTCAGCTCTTCCGCCCTGACAACTTCGTCTTCGGTCAGTCCG
GTGCC
>P_telopeae_CBS_114161
AGGGATCATTATAGAGTTTTCTAAACTCCCAACCCATGTGAACTTACC-
ATTGTTGCCTCGGCAGAAGCTGCTCGGT-
GCACCCTACCTTGGAACGGCCTACCCTGTAGCGCCTTACCCTGGAACGGCTTACCCTGTAACGG
CTGCCGGTGGACTACCAAACTCTTGTTATTTTATTGTAATCTGAGCGTCTTATTTTAATAAGTCAA
AACTTTCAACAACGGATCTCTTGGTTCTGGCATCGATGAAGAACGCAGCGAAATGCGATAAGTA
ATGTGAATTGCAGAATTCAGTGAATCATCGAATCTTTGAACGCACATTGCGCCCATTAGTATTCT
AGTGGGCATGCCTGTTCGAGCGTCATTTCAACCCTTAAGCCTAGCTTAGTGTTGGGAGCCTACT
G--
CTTTTGCTAGCTGTAGCTCCTGAAATACAACGGCGGATCTGCGATATCCTCTGAGCGTAGTAAAT
TTTTATCTCGCTTTTGACTGGAGTTGCAGCGTCTTTGGCCGCTAAATCCCCC-AA-
TTTTTAATGGTTGACCTCAGGTTAGTCATCCTCGCAATCCCCATCATCC------TCATC--------
CTCATTATCACCACCT--CGCAAA----CA-TTTCCACACC-GGTGCCGAAAATCTGG--------
TTTTCGCACCTGCC-CATTTTCCCAG--ACACTTACCCC--------
GCCGCACGACCCCGCGGTGCAAACGAAAAATTTCTTA--
TCACAGCCCCACATCACACAAACATTTTGGCAGCCACGCACTTTGCATGACCCACAATGAACAA
TTGCTGACCCCGCCAAATAGGAAGCCGCCGAGCTCGGAAAGGGTTCCTTCAAGTACGCATGGG
TTCTTGACAAGCTCAAGGCCGAGCGTGAGCGTGGTATCACCATTGATATCGCTCTCTGGAAGTT
CGAGACCAACGAGTACAATGTCACCGTCATTGGTTAGTATCCCTGTCCACAACATGTGTCATGT
CTCTGAACTCAAGACTAACCTTGCAATACAGACGCTCCCGGTCACCGTGATTTCATCAATGCTG
CCTTCTGGTATGTAGCC-CATCTACCTCGACGC-
GCCTCAATACGACACCCCCCGGCAACTCGACAAC--GACATTCTTAAC---
TGCTTGTTTGGAACCATACAAAAGACTTGATACTGACCGGTCTATGATAGGCAAACCATCTCTG
GCGAGCACGGTCTCGACAGCAATGGAGTGTATGTACCCTTTCCCTGGCTATTCGCTTTCTCGTGA
ACATGTCAGCTAACAGTCG-

TGCTTGTTCAGCTACAACGGTACCTCCGAGCTCCAGCTCGAGCGCATGAGCGTCTACTTCAACG
AGGCTTCCGGCAACAAGTACGTTCCTCGTGCCGTCCTCGTCGATCTCGAGCCCGGTACCATGGA
TGCCGTCCGCGCCGGTCCTTTCGGTCAGCTCTTCCGCCCTGACAACTTCGTCTTCGG??????????
??
>P_thailandica_MFLUCC_17_1616
AGGGATCATTATAGAGTTTTCTAAACTCCCAACCCATGTGAACTTACC-
ATTGTTGCCTCGGCAGAAGCTGCTCGGT-
GCACCTTACCTTGGAACGGCCTACCCTGTAGCGCCTTACCCTGGAACGGCTTACCCTGCAACGG
CTGCCGGTGGACTACCAAACTCTTGTTATTTTATTGTAATCTGAGCGTCTTATTTTAATAAGTCAA
AACTTTCAACAACGGATCTCTTGGTTCTGGCATCGATGAAGAACGCAGCGAAATGCGATAAGTA
ATGTGAATTGCAGAATTCAGTGAATCATCGAATCTTTGAACGCACATTGCGCCCATTAGTATTCT
AGTGGGCATGCCTGTTCGAGCGTCATTTCAACCCTTAAGCCTAGCTTAGTGTTGGGAGCCTACT
G--
CTTTTACTAGCTGTAGCTCCTGAAATACAACGGCGGATCTGCGATATCCTCTGAGCGTAGTAAAT
TTTTATCTCGCTTTTGACTGGAGTTGCAGCGTCTTTAGCCGCTAAATCCCCC-AA-
TTTTTAATGGTTGACCTC???TTAGTCATCCTCGCAATCCC-ATCATCC------TCATC--------
TTCATCACCATCACCT--CGCAAA----CA-TTTCCACACC-GGTGCCGAAAATCT-G--------
TTTTCGCACCTGCC-CATTTTCCCAG--ACACTTACCCC--------
GCCGCACGACCCCGCGGTGCAAACGAAAAATTTCTTA--TCATAGCCCCACATCAC--
AAACATTTTGGCAGCCACGCACTCTGCATGACCCACAATGAACAATTGCTGACCCCGCCAAATA
GGAAGCCGCCGAGCTCGGAAAGGGTTCCTTCAAGTACGCATGGGTTCTTGACAAGCTCAAGGC
CGAGCGTGAGCGTGGTATCACCATTGATATCGCTCTCTGGAAGTTCGAGACCAACGAGTACAAT
GTCACCGTCATTGGTTAGTATCCCTGCCCACAACATGTGTCATGTCTCCGAACTCGAGACTAACC
TTACAATACAGACGCTCCCGGTCACCGTGATTTCATCAATGCTGCCTTCTGGTATGTAGCC-
CATCTACCCCGACGC-GTCTCAATACGACA-CCCCCGGCAACTCGACAAC--GACGTTCTCAAC-
--
TGCTTGGTTGAAACCAAATGAAAGACTTGATACTGACCGGTCTCTGATAGGCAAACCATCTCTG
GCGAGCACGGTCTCGACAGCAATGGAGTGTACGTACCCTTTCCCTGGCTACTCGCTTTCTCGTG
AACATGTCAGCTAACACTCG-
TGCTTGTTCAGCTACAACGGTACCTCCGAGCTCCAGCTCGAGCGCATGAGCGTCTACTTCAACG
AGGCTTCCGGCAACAAGTACGTTCCTCGTGCCGTCCTCGTCGATCTCGAGCCCGGTACCATGGA
TGCCGTCCGCGCCGGTCCTTTCGGTCAGCTCTTCCGCCCTGACAACTTCGTCTTCGGTCAGTCCG
GTGCC
>P_thailandica_MFLUCC_17_1617
AGGGATCATTATAGAGTTTTCTAAACTCCCAACCCATGTGAACTTACC-
ATTGTTGCCTCGGCAGAAGCTGCTCGGT-
GCACCTTACCTTGGAACGGCCTACCCTGTAGCGCCTTACCCTGGAACGGCTTACCCTGCAACGG
CTGCCGGTGGACTACCAAACTCTTGTTATTTTATTGTAATCTGAGCGTCTTATTTTAATAAGTCAA
AACTTTCAACAACGGATCTCTTGGTTCTGGCATCGATGAAGAACGCAGCGAAATGCGATAAGTA
ATGTGAATTGCAGAATTCAGTGAATCATCGAATCTTTGAACGCACATTGCGCCCATTAGTATTCT
AGTGGGCATGCCTGTTCGAGCGTCATTTCAACCCTTAAGCCTAGCTTAGTGTTGGGAGCCTACT
G--
CTTTTACTAGCTGTAGCTCCTGAAATACAACGGCGGATCTGCGATATCCTCTGAGCGTAGTAAAT
TTTTATCTCGCTTTTGACTGGAGTTGCAGCGTCTTTAGCCGCTAAATCCCCC-AA-

TTTTTAATGGTTGACCTC???TTAGTCATCCTCGCAATCCC-ATCATCC------TCATC--------
TTCATCACCATCACCT--CGCAAA----CA-TTTCCACACC-GGTGCCGAAAATCT-G--------
TTTTCGCACCTGCC-CATTTTCCCAG--ACACTTACCCC--------
GCCGCACGACCCCGCGGTGCAAACGAAAAATTTCTTA--TCATAGCCCCACATCAC--
AAACATTTTGGCAGCCACGCACTCTGCATGACCCACAATGAACAATTGCTGACCCCGCCAAATA
GGAAGCCGCCGAGCTCGGAAAGGGTTCCTTCAAGTACGCATGGGTTCTTGACAAGCTCAAGGC
CGAGCGTGAGCGTGGTATCACCATTGATATCGCTCTCTGGAAGTTCGAGACCAACGAGTACAAT
GTCACCGTCATTGGTTAGTATCCCTGCCCACAACATGTGTCATGTCTCCGAACTCGAGACTAACC
TTACAATACAGACGCTCCCGGTCACCGTGATTTCATCAATGCTGCCTTCTGGTATGTAGCC-
CATCTACCCCGACGC-GTCTCAATACGACA-CCCCCGGCAACTCGACAAC--GACGTTCTCAAC-
--
TGCTTGGTTGAAACCAAATGAAAGACTTGATACTGACCGGTCTCTGATAGGCAAACCATCTCTG
GCGAGCACGGTCTCGACAGCAATGGAGTGTACGTACCCTTTCCCTGGCTACTCGCTTTCTCGTG
AACATGTCAGCTAACACTCG-
TGCTTGTTCAGCTACAACGGTACCTCCGAGCTCCAGCTCGAGCGCATGAGCGTCTACTTCAACG
AGGCTTCCGGCAACAAGTACGTTCCTCGTGCCGTCCTCGTCGATCTCGAGCCCGGTACCATGGA
TGCCGTCCGCGCCGGTCCTTTCGGTCAGCTCTTCCGCCCTGACAACTTCGTCTTCGGTCAGTCCG
GTGCC
>P_trachycarpicola_IFRDCC_2240
??????CATTATGGAGTTTTCTAAACTCCCAACCCATGTGAACTTACC-
ATTGTTGCCTCGGCAGAAGCTGCTCGGT-
GCACCCTACCTTGGAACGGCCTACCCTGTAGCGCCTTACCCTGGAACGGCTTACCCTGTAACGG
CTGCCGGTGGACTACCAAACTCTTGTTATTTTATTGTAATCTGAGCGTCTTATTTTAATAAGTCAA
AACTTTCAACAACGGATCTCTTGGTTCTGGCATCGATGAAGAACGCAGCGAAATGCGATAAGTA
ATGTGAATTGCAGAATTCAGTGAATCATCGAATCTTTGAACGCACATTGCGCCCATTAGTATTCT
AGTGGGCATGCCTGTTCGAGCGTCATTTCAACCCTTAAGCCTAGCTTAGTGTTGGGAGCCTACT
G--
CTTTTGCTAGCTGTAGCTCCTGAAATACAACGGCGGATCTGCGATATCCTCTGAGCGTAGTAAAT
TTTTATCTCGCTTTTGACTGGAGTTGCAGCGTCTTTGGCCGCTAAATCCCCC-AA-
TTTTTAATGGTTGACCTCAGGTTAGTCATCCTCGCAATCCC-ATCATCC------TCATC--------
CTCATCATCATCACTT--CGCAAA----CATTTCCCACACC-GGTGTCGAAAATCTGG--------
TTTTCGCACCTGCC-CATTTTCTCAG--ACACTTACCCC--------
GCCGCACGACCCCGCGGTGCAAACGAAAAATTTCTTA--
TCACAGCCCCACATCACACAAACATTTTGGCAGCCACGCACCTTGCATGACCCACAATGAACAA
TTGCTGACCCCGCCAAATAGGAAGCCGCCGAGCTCGGAAAGGGTTCCTTCAAGTACGCATGGG
TTCTTGACAAGCTCAAGGCCGAGCGTGAGCGTGGTATCACCATTGATATCGCTCTCTGGAAGTT
CGAGACCAACGAGTACAATGTCACCGTCATTGGTTAGTATCCCTGCCCACAATATGTGTCATGT
CTCTGAACTCAAGACTAACCTTGCAATACAGACGCTCCCGGTCACCGTGATTTCATCAATGCTG
CTTTCTGGTATGTAGCC-CATCTACCTCGACGC-GCCTCAATACGACA-
CCCCCGGCAACTCGACCAC--GATAATCTCAAC---
TGCTTGGTTGGAACCATACGAAAGACTCGATACTGACCGGTCTATGATAGGCAAACCATCTCTG
GCGAGCACGGTCTCGACAGCAATGGAGTGTACGTACCCTTTCCCTGGCTGTTCGCTTTCTCGTG
AACATGTCAGCTAACAGTCG-
TGCTTGTTCAGCTACAACGGTACCTCCGAGCTCCAGCTCGAGCGCATGAGCGTCTACTTCAACG

AGGCTTCCGGCAACAAGTACGTTCCTCGTGCCGTCCTCGTCGATCTCGAGCCCGGTACCATGGA
TGCCGTCCGCGCCGGTCCTTTCGGTCAGCTCTTCCGCCCTGACAACTTCGTCTTCGGTCAGTCCG
GTGCC
>P_unicolor_MFLUCC_12_0276
??????CATTATAGAGTTTTCTAAACTCCCAACCCATGTGAACTTACC-
ATTGTTGCCTCGGCAGAAGCTACCTGGT--
TACCTTACCTTGGAACGGCCTACCCTGTAGCGCCTTACCCTGGAACGGCCTACCCTGTAACGGC
TGCCGGTGGACTACCAAACTCTTGTTATTTTATTGTAATCTGAGCGTCTTATTTTAATAAGTCAAA
ACTTTCAACAACGGATCTCTTGGTTCTGGCATCGATGAAGAACGCAGCGAAATGCGATAAGTAA
TGTGAATTGCAGAATTCAGTGAATCATCGAATCTTTGAACGCACATTGCGCCCATTAGTATTCTA
GTGGGCATGCCTGTTCGAGCGTCATTTCAACCCTTAAGCCTAGCTTAGTGTTGGGAGCCTACTG-
-CTTTTGCTAGCGGTAGCTCCTGAAATACAACGGCGGATCTGCGATATCCTCTGAGCGTAGTAA-
TTTTTATCTCGCTTTTGACTGGAGTTGCAGCGTCTTTAGCCGCTAAACCCCCCAAA-
TTTTTAATGGTTGACCTC??
??
??
??
??
??
TGCTGCTTTCTGGTATGTAGCC-CATCTACCTCGACAC-
GCCTCAATACGACAACCCCCCGCAACTCGACAAC--
GACGTTCTCAACAAGTGCTTGGTTGGAAACAAGGGAAAGACTTGATACTGACCGGTCCCTGATA
GGCAAACCATCTCTGGCGAGCACGGTCTCGACAGCAATGGAGTGTACGTACCCTTTCCTTGGCT
ACTTGCTTTCCCACGAACATCTCAGCTAACACTCG-
TGGTTTTTCAGCTACAACGGTACCTCCGAGCTCCAGCTCGAGCGCATGAGCGTCTACTTCAACG
AGGCTTCCGGCAACAAGTACGTTCCTCGTGCCGTCCTCGTCGATCTCGAGCCCGGTACCATGGA
TGCCGTCCGCGCCGGTCCTTTCGGTCAGCTCTTCCGCCCTGACAACTTCGTCTTCGGTCAGTCCG
GTGCC
>P_verruculosa_MFLUCC_12_0274
??????CATTATAGAGTTTTCTAAACTCCCAACCCATGTGAACTTACC-
ATTGTTGCCTCGGCAGAAGCTACCTGGT--
TACCCTACCTTGGAACGGCCTACCCTGTAGCGCCTTACCCTGGAACGGCCTACCCTGTAACGGC
TGCCGGTGGACTACCAAACTCTTGTTATTTTATTGTAATCTGAGCGTCTTATTTTAATAAGTCAAA
ACTTTCAACAACGGATCTCTTGGTTCTGGCATCGATGAAGAACGCAGCGAAATGCGATAAGTAA
TGTGAATTGCAGAATTCAGTGAATCATCGAATCTTTGAACGCACATTGCGCCCATTAGTATTCTA
GT-GGCATGCCTGTTCGAGCGTCATTTCAACCCTTAAGCCTAGCTTAGTGTTGGGAGCCTACCG-
-CTTTTGCTAGCGGTAGCTCCTGAAATACAACGGCGGATCTGCGATATCCTCTGAGCGTAGTAA-
TTTTTATCTCGCTTTTGACTGGAGTTGCAGCGTCTTTAGCCGCTAAACCCCCCAAATTTTTTAATG
GTTGACCTCAGGTTAGTCATCCACACAATCCC-ATCATTC------
CCATCATTCCCACCTTATCATCATCGCCT----CAAA----CA-TCTTCCAACC-
GGTGCCGAAAATCT-G--------TTTTCGCGCCTGCC-CATTTTCCCAG--GCACTTACCCC-----
---GCCGCACGACCCCGCGGTGCAAACGAAAAATTTCTTA--
TCACAGCCCCACATCGCACAAACATTTTGGCAGCCATGCACTTTCCAAGACCCACAATGAACAA
TTGCTGACCCCGCCAAATAGGAAGCCGCCGAGCTCGGAAAGGGTTCCTTCAAGTACGCATGGG

TTCTTGACAAGCTCAAGGCCGAGCGTGAGCGTGGTATCACCATTGATATCGCTCTCTGGAAGTT
CGAGACCAACGAGTACAATGTCACCGTCATTGGTTAGTATCCCTGCCCACACGATGTATCATGT
ATCCGACCTT-GTGCTAACATGGCAACACAGATGCTCCCGGTCACCGTGA-
TTCATCAA??
??
??
??
??
>P_yanglingensis_LC4553
???????????????????????????????ACCCATGTGAACTTACC-
ATTGTTGCCTCGGCAGAAGCTACCCGGT---
ACCTTACCTTGGAACGGCCTACCCTGTAGCGCCTTACCCTGGAACGGCTTACCCTGCAGCGGCT
GCCGGTGGACTACCAAACTCTTGTTATTTTTATGTTATCTGAGCGTCTTATTTTAATAAGTCAAAA
CTTTCAACAACGGATCTCTTGGTTCTGGCATCGATGAAGAACGCAGCGAAATGCGATAAGTAAT
GTGAATTGCAGAATTCAGTGAATCATCGAATCTTTGAACGCACATTGCGCCCATTAGTATTCTAG
TGGGCATGCCTGTTCGAGCGTCATTTCAACCCTTAAGCCTAGCTTAGTGTTGGGAGCCTACTG--
CTTTTGTTAGCTGTAGCTCCTGAAATACAACGGCGGATCTGCGATATCCTCTGAGCGTAGTAATT
TTTTTCCTCGCTTTTGACTGGAGTTGCAGCGTCTTTAGCCGCTAAACCCCCC-AA-
TTTTTAATGGTTGACCTC????????????????CAACCCC-ATCATCC--------------------
TCACTATCATTACCT--CGCAAA----CA-TTTCCAAACC-
GGTGTCGAAAATCTGGTGTTTCTTTTTTCGCGCCTGCC-CACATTCCCAG--ACACTTACCCC----
----GCCGCACGACCCCGCGGTGCAAACGAAAAATTTCTTA--
TCACAGCCCCACATCGCACAAACATTTTGGCAGCCATGCACTTTTCATAACCCAC-
ACGAGCATTTGCTGACCCCGCCAAACAGGAAGCCGCCGAGCTCGGAAAGGGTTCCTTCAAGTA
CGCATGGGTTCTTGACAAGCTCAAGGCCGAGCGTGAGCGTGGTATCACCATCGATATCGCTCTC
TGGAAGTTCGAGACCAACGAGTACAATGTCACCGTCATTGGTTAGTATCCCTGTCCACAACATC
CATCATGCATCCGAAATC-
AGACTAACACGGCACCACAGATGCTCCCGGTCACCGTGATTTTATCAATGCTGCTTTCTGGTATG
TAGCT-CATCTACCTCGACGC-GCCTCAGCGCGACG-CCTCTCGCAACTCGACAAC--
GACGTTATCAAC---
TACTTGGTTGAAACCAAGCGAAAGACTTGATACTGACAGGCCTCTGATAGGCAAACCATCTCTG
GCGAGCACGGTCTCGACAGCAATGGAGTGTACGTACCGTACCCTTGGCTACCTACT-
TCCCACGAACATGTCAGCTAACACTTG----
TGGTTCAGCTACAACGGTACCTCCGAGCTCCAGCTCGAGCGCATGAGCGTCTACTTCAACGAGG
CTTCCGGCAACAAGTACGTTCCTCGTGCCGTCCTCGTCGATCTCGAGCCCGGTACCATGGATGC
CGTCCGCGCCGGTCCTTTCGGTCAGCTCTTCCGCCCTGACAACTTCGTCTTCGGTCAGTCCGGTG
CC
>Pestalotiopsis_appendiculata_CGMCC_3_23550
?????????????????????????CTCCCAACCCATGTGAACTTACC-
ATTGTTGCCTCGGCAGAAGCTACCTGGT--
TACCTTACCTTGGAACGGCCTACCCTGTAGCGCCTTACCCTGGAACGGCCTACCCTGTAACGGC
TGCCGGTGGACTACCAAACTCTTGTTATTTTATTGTAATCTGAGCGTCTTATTTTAATAAGTCAAA
ACTTTCAACAACGGATCTCTTGGTTCTGGCATCGATGAAGAACGCAGCGAAATGCGATAAGTAA
TGTGAATTGCAGAATTCAGTGAATCATCGAATCTTTGAACGCACATTGCGCCCATTAGTATTCTA

GTGGGCATGCCTGTTCGAGCGTCATTTCAACCCTTAAGCCTAGCTTAGTGTTGGGAGCCTACTG-
-CTTTTGCTAGCGGTAGCTCCTGAAATACAACGGCGGATCTGCGATATCCTCTGAGCGTAGTAA-
TTTTTATCTCGCTTTTGACTGGAGTTGCAGCGTCTTTAGCCGCTAAACCC??????????????????????
?????????????????????????C---CAACC--
----------------GGTGCCGAGAATCT-G--------TTTTCGCATCTGCC-CATTTTCCCAG--
ACACTTACCCC--------GCCGCACGACCCCGCGGTGCAAACGAAAAATTTCTTA--
TCACAGCCCCACATCGCACAAACATTTCGGCAGCCATGCACTTTCCAAGACCCACAATGAACAT
TTGCTGACCCCGCCAAATAGGAAGCCGCCGAGCTCGGAAAGGGTTCCTTCAAGTACGCATGGG
TTCTTGACAAGCTCAAGGCCGAGCGTGAGCGTGGTATCACCATCGATATCGCTCTCTGGAAGTT
CGAGACCAACGAGTACAATGTCACCGTCATTGGTTAGTATTCCTGTCCACACGATGTACCATGC
ATCTGAATGT-
ATACTAACATGGCAACACAGATGCTCCCGGTCACCGTGA??????????????????????TATGTAGGA
-CCTCTACCTCGACAC-GCCTCAATACGACAACCCCCCGCAACTCGACAAC--
GACGTTCTCAACAAGTGCTTGGTTGGAAACAAGGGAAAGACTTGATACTGACCGGTCCCTGATA
GGCAAACCATCTCTGGCGAGCACGGTCTCGACAGCAATGGAGTGTACGTACCCTTTCCTTAGCT
ACTTGCTTTCCCACGAACATCTTAGCTAACACTCG-
TGGTTGTTCAGCTACAACGGTACCTCCGAGCTCCAGCTCGAGCGCATGAGCGTCTACTTCAACG
AGGCTTCCGGCAACAAGTACGTTCCTCGTGCCGTCCTCGTCGATCTCGAGCCCGGTACCATGGA
TGCCGTCCGCGCCGGTCCTTTCGGTCAGCTCTTCCGCCCTGACAACTTCGTCTTCGGTCAGTCCG
GTGCC
>Pestalotiopsis_cangshanensis_CGMCC_3_23544
???CTTACC-
ATTGTTGCCTCGGCAGAAGCTGCTCGGT-
GCACCTTACCCTGGAACGGCCTACCCTGTAGCGCCTTACCCTGGAACGGCTTACCCTGTAGCGG
CTGCCGGTGGACTACCAAACTCTTGTTATTTTATTGTAATCTGAGCGTCTTATTTTAATAAGTCAA
AACTTTCAACAACGGATCTCTTGGTTCTGGCATCGATGAAGAACGCAGCGAAATGCGATAAGTA
ATGTGAATTGCAGAATTCAGTGAATCATCGAATCTTTGAACGCACATTGCGCCCATTAGTATTCT
AGTGGGCATGCCTGTTCGAGCGTCATTTCAACCCTTAAGCCTAGCTTAGTGTTGGGAGCCTACT
G--
CTTTTGCTAGCGGTAGCTCCTGAAATACAACGGCGGATCTGCGATATCCTCTGAGCGTAGTAA-
TTTTTATCTCGCTTTTGACTGGAGTTGCAGCGTCTTTAGCCGCTAAATCCCCC-AA-
TTTTTAATGGTTGACCTC????????????????CAATCCC-ATCATTC------CCATC--------
CTCATCATCATCACCT--CGCAAA----CA-TTTTCCAACT-GGTGCCGAAAATCT-G--------
TTTTCGCACCTGCC-CATTTTCCCAG--ACACTTACCCC--------
GCCGCACGACCCCGCGGTGCAAACGAAAAATTTCTTA--
TCACAGCCCCACATCGCACAAACATTTTGGCAGTCATGCACTTTCCAAGACCCACAATGAACAA
TTGCTGACCCCGCCAAATAGGAAGCCGCCGAGCTCGGAAAGGGTTCCTTCAAGTACGCATGGG
TTCTTGACAAGCTCAAGGCCGAGCGTGAGCGTGGTATCACCATTGATATCGCTCTCTGGAAGTT
CGAGACCAACGAGTACAATGTCACCGTCATTGGTTAGTATCCCTGTCCACACGATGTACCATGC
ATCCGACCTT-
GTACTAACATGGCAATCCAGATGCTCCCGGTCACCGTGATTTCATCAA???????????????????????
ACTCTA-CTCGACGC-GCCCTCATACGACA-CCCCCGGCAACTCGACCAC--GATAATCTCAAC-
--
TGCTTGGTTGGAACCATACGAAAGACTCGATACTGACCGGTCTATGATAGGCAAACCATCTCTG

GCGAGCACGGTCTCGACAGCAATGGAGTGTACGTACCCTTTCCCTGGCTGTTCGCTTTCTCGTG
AACATGTCAGCTAACAGTCG-
TGCTTGTTCAGCTACAACGGTACCTCCGAGCTCCAGCTCGAGCGCATGAGCGTCTACTTCAACG
AGGCTTCCGGCAACAAGTACGTTCCTCGTGCCGTCCTCGTCGATCTCGAGCCCGGTACCATGGA
TGCCGTCCGCGCCGGTCCTTTCGGTCAGCTCTTCCGCCCTGACAACTTCGTCTTCGGTCAGTCCG
GTGCC
>Pestalotiopsis_chiaroscuro_BRIP_72970
??????CATTATAGAGTTTTCTAAACTCCCAACCCATGTGAACTTACC-
ATTGTTGCCTCGGCAGAAGCTGCTCGGT-
GCACCTTACCTTGGAACGGCCTACCCTGTAGCGCCTTACCCTGGAACGGCTTACCCTGCAACGG
CTGCCGGTGGACTACCAAACTCTTGTTATTTTATGGTTATCTGAGCGTCTTATTTTAATAAGTCAA
AACTTTCAACAACGGATCTCTTGGTTCTGGCATCGATGAAGAACGCAGCGAAATGCGATAAGTA
ATGTGAATTGCAGAATTCAGTGAATCATCGAATCTTTGAACGCACATTGCGCCCATTAGTATTCT
AGTGGGCATGCCTGTTCGAGCGTCATTTCAACCCTTAAGCCTAGCTTAGTGTTGGGAGCCTACT
G--
CTTTTGCTAGCGGTAGCTCCTGAAATACAACGGCGGATCTGCGATATCCTCTGAGCGTAGTAA-
TTTTTATCTCGCTTTTGACTGGAGTTGCAGCGTCTTTAGCCGCTAAACCCCCC-AA-
ATTTTAATGGTTGACCTCAGGTTAGTCATGCTCAAAGTCCC-ATCATCC------TCATC--------
---ATCATCATCGCCT--CGCAAA----CA-TTTTCCAACC-GGTGCCGAAATTCT-G--------
TTTTCGCACCTGCC-CATTTTCCCAG--ACACTTACCCC--------
GCCGCACGACCCCGCGGTGCAAACGAAAAATTTCTTA--TCACAGCCCCACTTCACAC-
AACATTTTGGCAGCCACGCACTTTGCATGACCCACAATGAACAATTGCTGACCCCGCCAAATAG
GAAGCCGCCGAGCTCGGAAAGGGTTCCTTCAAGTACGCATGGGTTCTTGACAAGCTCAAGGCC
GAGCGTGAGCGTGGTATCACCATTGATATCGCTCTCTGGAAGTTCGAGACCAACGAGTACAATG
TCACCGTCATTGGTTAGTATCCCTGTCCACAGAAAGTATCATGTGTCCGAACTC-
AGACTAACATCGCAATACAGACGCTCCCGGTCACCGTGATTTCATCAATGCTGCCTTCTGGTAT
GTAGCC-CATCTACCTCGACAC-GCCTCAATACGACA-CCCTCCGCAACTCGACGAC--
GGCATTCTCGGC---
TACTTGGTTGGAACCAAACGAAAGACTTGATACTGACCGGTCTCTGATAGGCAAACCATCTCTG
GCGAGCACGGTCTCGACAGCAATGGAGTGTACGTACCGCTTCCTTGGCTACTTGCTTTCCCACG
AACATGTTAGCTAACACTCG-
TGCTTGCTCAGCTACAACGGCACCTCCGAGCTCCAGCTCGAGCGCATGAGCGTCTACTTCAACG
AGGCTTCCGGCAACAAGTACGTTCCTCGTGCTGTCCTCGTCGATCTCGAGCCCGGTACCATGGA
CGCCGTCCGCGCCGGTCCTTTCGGCCAGCTCTTCCGCCCTGACAACTTCGTCTTCGGTCAGTCC
GGTGCC
>Pestalotiopsis_daliensis_CGMCC_3_23548
?????????????????????????CTCCCAACCCATGTGAACTTACC-
ATTGTTGCCTCGGCAGAAGCTACCTGGT--
TACCTTACCTTGGAACGGCCTACCCTGTAGCGCCTTACCCTGGAACGGCCTACCCTGTAACGGC
TGCCGGTGGACTACCAAACTCTTGTTATTTTATTGTAATCTGAGCGTCTTATTTTAATAAGTCAAA
ACTTTCAACAACGGATCTCTTGGTTCTGGCATCGATGAAGAACGCAGCGAAATGCGATAAGTAA
TGTGAATTGCAGAATTCAGTGAATCATCGAATCTTTGAACGCACATTGCGCCCATTAGTATTCTA
GTGGGCATGCCTGTTCGAGCGTCATTTCAACCCTTAAGCCTAGCTTAGTGTTGGGAGCCTACTG-
-CTTTTGCTAGCGGTAGCTCCTGAAATACAACGGCGGATCTGCGATATCCTCTGAGCGTAGTAA-

TTTTTATCTCGCTTTTGACTGGAGTTGCAGCGTCTTTAGCCGCTA??????????????????????????????
????GTAATCATC-------TTCC-ATCCTCA-----------------------TCATCATCGCCT--
CGCAAA----CA-TTTTCCAACC-GGTGCCGAGAATCT-G--------TTTTCGCATCTGCC-
CATTTTCCCAG--ACACTTACCCC--------
GCCGCACGACCCCGCGGTGCAAACGAAAAATTTCTTA--
TCACAGCCCCACATCGCACAAACATTTTGGCAGCCATGCACTTTCCAAGACCCACAATGAACAT
TTGCTGACCCCGCCAAATAGGAAGCCGCCGAGCTCGGAAAGGGTTCCTTCAAGTACGCATGGG
TTCTTGACAAGCTCAAGGCCGAGCGTGAGCGTGGTATCACCATCGATATCGCTCTCTGGAAGTT
CGAGACCAACGAGTACAATGTCACCGTCATTGGTTAGTATCCCTGTCCACACGATGTACCATGC
ATCTGAATGT-
ATACTAACATGGCAACACAGATGCTCCCGGTCACCGTGA????????????????????GGTATGTAGC
C-CATCTACCTCGACAC-GCCTCATTACGACAACCCCCCGCAACTCGACAAC--
GACGTTCTCAACAAGTGCTTGCTTGGAAATAAGGGAAAGACTTGATACTGACCGGTCCCTGATA
GGCAAACCATCTCTGGCGAGCACGGTCTCGACAGCAATGGAGTGTATGTATCCTTTCCTTGGCT
ACTTGCTTTCCCACGAACATCTCAGCTAACACTCG-
TGGTTGTTCAGCTACAACGGTACCTCCGAGCTCCAGCTCGAGCGCATGAGCGTCTACTTCAACG
AGGCTTCCGGCAACAAGTACGTTCCTCGTGCCGTCCTCGTCGATCTCGAGCCCGGTACCATGGA
TGCCGTCCGCGCCGGTCCTTTCGGTCAGCTCTTCCGCCCTGACAACTTCGTCTTCGGTCAGTCCG
GTGCC
>Pestalotiopsis_fusoidea_CGMCC_3_23545
???CGGCAGAAGCTGCTCGGT-
GCACCTTACCCTGGAACGGCCTACCCTGTAGCGCCTTACCCTGGAACGGCTTACCCTGTAGCGG
CTGCCGGTGGACTACCAAACTCTTGTTATTTTATTGTAATCTGAGCGTCTTATTTTAATAAGTCAA
AACTTTCAACAACGGATCTCTTGGTTCTGGCATCGATGAAGAACGCAGCGAAATGCGATAAGTA
ATGTGAATTGCAGAATTCAGTGAATCATCGAATCTTTGAACGCACATTGCGCCCATTAGTATTCT
AGTGGGCATGCCTGTTCGAGCGTCATTTCAACCCTTAAGCCTAGCTTAGTGTTGGGAGCCTACT
G--
CTTTTGCTAGCTGTAGCTCCTGAAATACAACGGCGGATCTGCGATATCCTCTGAGCGTAGTAA-
TTTTTATCTCGCTTTTGACTGGAGTTGCAACGTCTTTAGCCGCTAAATCCCCC-AA-
TTTTTAATGGTTGACCTC??????ACCATCAT-----TCCC-ATCATCA-----------------------
TCATCGTCGCCT--TGCGAA----CA-TTTCCCAACC-GGTGCCGAAATTCT-G--------
TTTTCGCACCTGCC-CACTTTCCCAG--ACACTTACCCC--------
GCCGCACGACCCCGCGGTGCAAACGAAAAATTTCTTA--
TCACAGCCCCACATCGCACAAACATTTTGGCAGCCATGCACTTTCCATGACCCACAATGAACCA
TTGCTGACCCCGCCAAATAGGAAGCCGCCGAGCTCGGAAAGGGTTCCTTCAAGTACGCATGGG
TTCTTGACAAGCTCAAGGCCGAGCGTGAGCGTGGTATCACCATTGATATCGCTCTCTGGAAGTT
CGAGACCAACGAGTACAATGTCACCGTCATTGGTTAGTATCCCTGTCTACACAATGTACCATGC
ATCCGACCTT-
GTACTAACATGGCAATCCAGATGCTCCCGGTCACCGTGATTTCATCAA??????????????????AGT
C-CATCTACCTCGACAC-GCCTCATTACGACAACCCTCCGCAACTCGACAAC--
GACGTTCTCAACAACTGCTTGGTTGGAACCAAAGAAAAGACCTGATACTGACCGGTCTCTGATA
GGCAAACCATCTCTGGTGAGCACGGTCTCGACAGCAATGGAGTGTACGTACCCTTTCCTTGGCT
ACTTGCTTTCCCACGAACATGTCAGCTAACACTCG-
TGGTTGTTCAGTTACAACGGTACCTCCGAGCTCCAGCTCGAGCGCATGAGCGTCTACTTCAACG

AGGCTTCCGGTAACAAGTACGTTCCTCGTGCCGTCCTTGTCGATCTCGAGCCCGGTACCATGGA
TGCCGTCCGCGCCGGTCCTTTCGGTCAGCTCTTCCGCCCTGACAACTTTGTCTTCGGTCAGTCCG
GTGCC
>Pestalotiopsis_hispanicaCBS115391
??????CATTATAGAGTTTTCTAAACTCCCAACCCATGTGAACTTACC-
ATTGTTGCCTCGGCAGAAGCTGCTCGGT-
ACACCCTACCTTGGAACGGCCTACCCTGTAGCGCCTTACCCTGGAACGGCTTACCCTGTAACGG
CTGCCGGTGGACTACCAAACTCTTGTTATTTTATTGTAATCTGAGCGTCTTATTTTAATAAGTCAA
AACTTTCAACAACGGATCTCTTGGTTCTGGCATCGATGAAGAACGCAGCGAAATGCGATAAGTA
ATGTGAATTGCAGAATTCAGTGAATCATCGAATCTTTGAACGCACATTGCGCCCATTAGTATTCT
AGTGGGCATGCCTGTTCGAGCGTCATTTCAACCCTTAAGCCTAGCTTAGTGTTGGGAGCCTACT
G--
CTTTTACTAGCTGTAGCTCCTGAAATACAACGGCGGATCTGCGATATCCTCTGAGCGTAGTAAAT
TTTTATCTCGCTTTTGACTGGAGTTGCAGCGTCTTTAGCCGCTAAATCCCCC-AA-
TTTTTAATGGTTGACCTC?????????ATCCTCGCAATCCC-ATCATCCTCATCATCATC--------
ATCATCATCACCACCT--CGCAAA----CG-TTGCCACACC-GGTGCCGAAAATCTGG--------
TTTTCGCACCTGCC-CATTTTCCCAG--ACACTTACCCC--------
GCCGCACGACCCCGCGGTGCAAACGAAAAATTTCTTA--
TCATAGCCCCACATCACACAAACATTTTGGCAGCCACGCACTTTGCATGACCCACAATGAACAA
TTGCTGACCCCGCCAAATAGGAAGCCGCCGAGCTCGGAAAGGGTTCCTTCAAGTACGCATGGG
TTCTTGACAAGCTCAAGGCCGAGCGTGAGCGTGGTATCACCATTGATATCGCTCTCTGGAAGTT
CGAGACCAACGAGTACAATGTCACCGTCATTGGTTAGTATCCCTGTCCACAACATGTGTCATGT
CTCCAAACTCAAGACTAACCTTGCAATACAGACGCTCCCGGTCACCGTGATTTCATCAATGCTG
CCTTCTGGTATGTAGCC-CATCTACCTCGACGC-
GCCTCAATACGACACCCCCGGGCATCACGACAAC--GACATTCTCAAC---
TGCTTGTTTGGAACCATACGAAAGACTTGATACTGACCGGTCTATGATAGGCAAACCATCTCTG
GCGAGCACGGTCTCGACAGCAATGGAGTGTACGTACCCTTTCTCTGGCTACCCGCGTTCTCGTG
AAGATGTCAGCTAACAGTCG-
TGCTTGTTTAGCTACAACGGTACCTCCGAGCTCCAGCTCGAGCGCATGAGCGTCTACTTCAACG
AGGCTTCCGGCAACAAGTACGTTCCTCGTGCCGTCCTCGTCGATCTCGAGCCCGGTACCATGGA
TGCCGTCCGCGCCGGTCCTTTCGGCCAGCTCTTCCGCCCTGACAACTTCGTCTTCGGTCAGTCCG
GTGC?
>Pestalotiopsis_kenyana_CBS_911_96
AGGGATCATTATAGAGTTTTCTAAACTCCCAACCCATGTGAACTTACC-
ATTGTTGCCTCGGCAGAAGCTGCTCGGT-
GCACCCTACCTTGGAACGGCCTACCCTGTAGCGCCTTACCCTGGAACGGCTTACCCTGTAACGG
CTGCCGGTGGACTACCAAACTCTTGTTATTTTATTGTAATCTGAGCGTCTTATTTTAATAAGTCAA
AACTTTCAACAACGGATCTCTTGGTTCTGGCATCGATGAAGAACGCAGCGAAATGCGATAAGTA
ATGTGAATTGCAGAATTCAGTGAATCATCGAATCTTTGAACGCACATTGCGCCCATTAGTATTCT
AGTGGGCATGCCTGTTCGAGCGTCATTTCAACCCTTAAGCCTAGCTTAGTGTTGGGAGCCTACT
G--
CTTTTGCTAGCTGTAGCTCCTGAAATACAACGGCGGATCTGCGATATCCTCTGAGCGTAGTAAAT
TTTTATCTCGCTTTTGACTGGAGTTGCAGCGTCTTTGGCCGCTAAATCCCCC-AA-
TTTTTAATGGTTGACCTCAGGTTAGTCATCCTCGCAATCCC-ATCATCC------TCATC--------

CTCATCATCATCACTT--CGCAAA----CATTTCCCACACC-GGTGTCGAAAATCTGG--------
TTTTCGCACCTGCC-CATTTTCTCAG--ACACTTACCCC--------
GCCGCACGACCCCGCGGTGCAAACGAAAAATTTCTTA--
TCACAGCCCCACATCACACAAACATTTTGGCAGCCACGCACCTTGCATGACCCACAATGAACAA
TTGCTGACCCCGCCAAATAGGAAGCCGCCGAGCTCGGAAAGGGTTCCTTCAAGTACGCATGGG
TTCTTGACAAGCTCAAGGCCGAGCGTGAGCGTGGTATCACCATTGATATCGCTCTCTGGAAGTT
CGAGACCAACGAGTACAATGTCACCGTCATTGGTTAGTATCCCTGCCCACAATATGTGTCATGT
CTCTGAACTCAAGACTAACCTTGCAATACAGACGCTCCCGGTCACCGTGATTTCATCAATGCTG
CCTTCTGGTATGTAGCC-CATCTACCTCGACGC-GCCTCAATACGACA-
CCCCCGGCAACTCGACCAC--GATAATCTCAAC---
TGCTTGGTTGGAACCATACGAAAGACTCGATACTGACCGGTCTATGATAGGCAAACCATCTCTG
GCGAGCACGGTCTCGACAGCAATGGAGTGTACGTACCCTTTCCCTGGCTGTTCGCTTTCTCGTG
AACATGTCAGCTAACAGTCG-
TGCTTGTTCAGCTACAACGGTACCTCCGAGCTCCAGCTCGAGCGCATGAGCGTCTACTTCAACG
AGGCTTCCGGCAACAAGTACGTTCCTCGTGCCGTCCTCGTCGATCTCGAGCCCGGTACCATGGA
TGCCGTCCGCGCCGGTCCTTTCGGTCAGCTCTTCCGCCCTGACAACTTCGTCTTCGGTCAGTCCG
GTGCC
>Pestalotiopsis_lushanensis_LC8182
AGGGATCATTATAGAGTTTTCTAAACTCCCAACCCATGTGAACTTACC-
ATTGTTGCCTCGGCAGAAGCTGCTCGGT-
GCACCTTACCCTGGAACGGCCTACCCTGTAGCGCCTTACCCTGGAACGGCTTACCCTGTAGCGG
CTGCCGGTGGACTACCAAACTCTTGTTATTTTATTGTAATCTGAGCGTCTTATTTTAATAAGTCAA
AACTTTCAACAACGGATCTCTTGGTTCTGGCATCGATGAAGAACGCAGCGAAATGCGATAAGTA
ATGTGAATTGCAGAATTCAGTGAATCATCGAATCTTTGAACGCACATTGCGCCCATTAGTATTCT
AGTGGGCATGCCTGTTCGAGCGTCATTTCAACCCTTAAGCCTAGCTTAGTGTTGGGAGCCTACT
G--
CTTTTGCTAGCGGTAGCTCCTGAAATACAACGGCGGATCTGCGATATCCTCTGAGCGTAGTAA-
TTTTTATCTCGCTTTTGACTGGAGTTGCAGCGTCTTTAGCCGCTAAATCCCCC-AA-
TTTTTAATGGTTGACCTCAGGTTAGTCATCCTCACAATCCC-ATCATCC-------------------
CCATCATCATCGTCT--CGCAAA----CA-TTTTCCAACT-GGTGCCGAAAATCT-G--------
TTTTCGCACCTGCC-CATTTTCCCAG--ACACTTACCCC--------
GCCGCACGACCCCGCGGTGCAAACGAAAAATTTCTTA--
TCACAGCCCCACATCGCACAAACATTTTGGCAGCCATGCACTTTCCATGACCCACAATGAACAA
TTGCTGACCCCGCCAAATAGGAAGCCGCCGAGCTCGGAAAGGGTTCCTTCAAGTACGCATGGG
TTCTTGACAAGCTCAAGGCCGAGCGTGAGCGTGGTATCACCATTGATATCGCTCTCTGGAAGTT
CGAGACCAACGAGTACAATGTCACCGTCATTGGTTAGTATCCCGGTCCACACCATGCACCATGC
ATCCGACCTT-
GTACTAACATCACAATACAGATGCTCCCGGTCACCGTGATTTCATCAATGCTGCCTTCTGGTACG
TAGTC-CATCTACCTCGACAC-GCCTCAATACGACAACCCTCCGCAACTCGACAAC--
GACCTTCTCAACAACTGCTTGGTTGGAACCAAAGAAAAGACCTGATACTGACCGGTCTCTGATA
GGCAAACCATCTCTGGCGAGCACGGTCTCGACAGCAATGGAGTGTACGTACCCTTTCCTTGGCT
ACTTGCTTTCCCACGAACATCTCAGCTAATACCCG-
TGATTGTGCAGCTACAACGGTACCTCTGAGCTCCAGCTCGAGCGCATGAGCGTCTACTTCAACG
AGGCTTCCGGCAACAAGTACGTTCCTCGTGCCGTCCTCGTCGATCTCGAGCCCGGTACCATGGA

TGCCGTCCGCGCCGGTCCTTTCGGTCAGCTCTTCCGCCCTGACAACTTTGTCTTCGGTCAGTCCG
GTGCC
>Pestalotiopsis_rosarioides_CGMCC_3_23549
??????????????????????AAACTCCCAACCCATGTGAACTTACC-
ATTGTTGCCTCGGCAGAAGCTACCTGGT--
TACCTTACCTTGGAACGGCCTACCCTGTAGCGCCTTACCCTGGAACGGCCTACCCTGTAACGGC
TGCCGGTGGACTACCAAACTCTTGTTATTTTATTGTAATCTGAGCGTCTTATTTTAATAAGTCAAA
ACTTTCAACAACGGATCTCTTGGTTCTGGCATCGATGAAGAACGCAGCGAAATGCGATAAGTAA
TGTGAATTGCAGAATTCAGTGAATCATCGAATCTTTGAACGCACATTGCGCCCATTAGTATTCTA
GTGGGCATGCCTGTTCGAGCGTCATTTCAACCCTTAAGCCTAGCTTAGTGTTGGGAGCCTACTG-
-CTTTTGCTAGCGGTAGCTCCTGAAATACAACGGCGGATCTGCGATATCCTCTGAGCGTAGTAA-
TTTTTATCTCGCTTTTGACTGGAGTTGCAGCGTCTTTAGCCGCTAAACCCCCC-AA-
TTTTTAATGGTTGACCTC???
???????????????????CCAACC-GGTGCCGAGAATCT-G--------TTTTCGCATCTGCC-
CATTTTCCCAG--ACACTTACCCC--------
GCCGTACGACCCCGCGGTGCAAACGAAAAATTTCTTA--
TCACAGCCCCACATCGCACAAACATTTTGGCAGCCATGCACTTTCCAAGACCCACAATGAACAT
TTGCTGACCCCGCCAAATAGGAAGCCGCCGAGCTCGGAAAGGGTTCCTTCAAGTACGCATGGG
TTCTTGACAAGCTCAAGGCCGAGCGTGAGCGTGGTATCACCATCGATATCGCTCTCTGGAAGTT
CGAGACCAACGAGTACAATGTCACCGTCATTGGTTAGTATTCCTGTCCACACGATGTACTATGC
ATCTGAATGT-
ATACTAACATGGCAACACAGATGCTCCCGGTCACCGTGATTTCATCAA???????????????????????
????????TCGACAC-GCCTCAATACGACAACCCCCCGCAACTCGACAAC--
GACGTTCTCAACAAGTGCTTGGTTGGAAACAAGGGAAAGACTTGATACTGACCGGTCCCTGATA
GGCAAACCATCTCTGGCGAACACGGTCTCGACAGCAATGGAGTGTACGTACCCTTTCCTTAGCT
ACTTGCTTTCCCACGAACATCTTAGCTAACACTCG-
TGGTTGTTCAGCTACAACGGTACCTCCGAGCTCCAGCTCGAGCGCATGAGCGTCTACTTCAACG
AGGCTTCCGGCAACAAGTACGTTCCTCGTGCCGTCCTCGTCGATCTCGAGCCCGGTACCATGGA
TGCCGTCCGCGCCGGTCCTTTCGGTCAGCTCTTCCGCCCTGACAACTTCGTCTTCGGTCAGTCCG
GTGCC
>Pestalotiopsis_sp_LC3637
???ATTGTTGCCTCGGCAGAAGCTGCTCGGT-
GCACCCTACCTTGGAACGGCCTACCCTGTAGCGCCTTACCCTGGAACGGCTTACCCTGTAGCGG
CTGCCGGTGGACTACCAAACTCTTGTTATTTTATTGTAATCTGAGCGTCTTATTTTAATAAGTCAA
AACTTTCAACAACGGATCTCTTGGTTCTGGCATCGATGAAGAACGCAGCGAAATGCGATAAGTA
ATGTGAATTGCAGAATTCAGTGAATCATCGAATCTTTGAACGCACATTGCGCCCATTAGTATTCT
AGTGGGCATGCCTGTTCGAGCGTCATTTCAACCCTTAAGCCTAGCTTAGTGTTGGGAGCCTACT
G--
CTTTTACTAGCTGTAGCTCCTGAAATACAACGGCGGATCTGCGATATCCTCTGAGCGTAGTAA-
TTTTTATCTCGCTTTTGACTGGAGTTGCAGCGTCTTTAGCCGCTAAATCCCCC-AA-
TTTTTAATGGTTGACCTCAGGTTAGTCATCCTCGCAATCCC-ATCATCC------TCATC--------
CTCATCATCATCACCT--CGCAAA----CA-TTTCCACACC-GGTGCCGAAAATCTGG--------
TTTTCGCACCTGCC-CATTTTCCCAG--ACACTTACCCC--------
GCCGCACGACCCCGCGGTGCAAACGAAAAATTTCTTA--

TCACAGCCCCACATCACACAAACATTTTGGCAGCCACGCACTTTGCATGACCCACAATGAACAA
TTGCTGACCCCGCCAAATAGGAAGCCGCCGAGCTCGGAAAGGGTTCCTTCAAGTACGCATGGG
TTCTTGACAAGCTCAAGGCCGAGCGTGAGCGTGGTATCACCATTGATATCGCTCTCTGGAAGTT
CGAGACCAACGAGTACAATGTCACCGTCATTGGTTAGTATCCCTGCCCACAACATGTGTCATGT
CTCTGAACTCAAGACTAACCTTGCAATACAGACGCTCCCGGTCACCGTGATTTCATCAATGCTG
CCTTCTGGTATGTAGCC-CATCTACCTCGACGC-
GCCTCAATACGACACCCCCCGGCAACTCGACAAC--GACATTCTCAAC---
TGCTTGTTTGGAACCATACGAAAGACTTGATACTGACCGGTCTATGATAGGCAAACCATCTCTG
GCGAGCACGGTCTCGACAGCAATGGAGTGTACGTACCCTTTCCCTGGCTACTCGCTTTCTCGTG
AACATGTCAGCTAACAGTTG-
TGCTTGTTCAGCTACAACGGTACCTCCGAGCTCCAGCTCGAGCGCATGAGCGTCTACTTCAACG
AGGCTTCCGGCAACAAGTAC??
???
>Pestalotiopsis_suae_CGMCC_3_23546
??????????????????????????????????CTCCCAACCCATGTGAACTTACC-
ATTGTTGCCTCGGCAGAAGCTGCTCGGT-
GCACCCTACCTTGGAACGGCCTACCCTGTAGCGCCTTACCCTGGAACGGCTTACCCTGTAGCGG
CTGCCGGTGGACTACCAAACTCTTGTTATTTTATTGTAATCTGAGCGTCTTATTTTAATAAGTCAA
AACTTTCAACAACGGATCTCTTGGTTCTGGCATCGATGAAGAACGCAGCGAAATGCGATAAGTA
ATGTGAATTGCAGAATTCAGTGAATCATCGAATCTTTGAACGCACATTGCGCCCATTAGTATTCT
AGTGGGCATGCCTGTTCGAGCGTCATTTCAACCCTTAAGCCTAGCTTAGTGTTGGGAGCCTACT
G--
CTTTTACTAGCTGTAGCTCCTGAAATACAACGGCGGATCTGCGATATCCTCTGAGCGTAGTAA-
TTTTTATCTCGCTTTTGACTGGAGTTGCAGCGTCTTTAGCCGCTAAATCCCCC-AA-
TTTTTAATGGTTGACCTCAGGTTAGTCATCCTCGCATTCCC-ATCATCC--------------------
TCATCCTCATCACCT--CGCAAA----CA-TTTCCACACT-GGTGCCGAAAATCTGG--------
TTTTCGCACCTGCC-CATTTTCCCAG--ACACTTACCCC--------
GCCGCACGACCCCGCGGTGCAAACGAAAAATTTCTTA--
TCACAGCCCCACATCACACAAACATTTTGGCAGCCACGCACTTTGCATGACCCACAATGAACAA
TTGCTGACCCCGCTAAATAGGAAGCCGCCGAGCTCGG--
AGGGTTCCTTCAAGTACGCATGGGTTCTTGACAAGCTCAAGGCCGAGCGTGAGCGTGGTATCAC
CATTGATATCGCTCTCTGGAAGTTCGAGACCAACGAGTACAATGTCACCGTCATTGGTTAGTATC
CCTGCCCACA---
CCAT??ACTCTA-CTCGACGC-GCCCTCATACGACA-
CCACCGGCAACTCGACAAC--GACATTGTCAAC---
TGCTTGGTTGAAACCAAAAGAAAGACCTGATACTGACCGGTCTTTGATAGGCAAACCATCTCTG
GCGAGCACGGTCTCGACAGCAATGGAGTGTACGTACCCTTTCCCTGGCTACTCGTTTTTCCACG
AACATGTCAGCTAACAGTCG-
TGCTTGTTCAGCTACAACGGTACCTCCGAGCTCCAGCTCGAGCGCATGAGCGTCTACTTCAACG
AGGCTTCCGGTAACAAGTACGTTCCTCGTGCCGTCCTCGTCGATCTCGAGCCCGGTACCATGGA
TGCCGTCCGCGCCGGTCCTTTCGGTCAGCTCTTCCGCCCTGACAACTTCGTCTTCGGCCAGTCCG
GTGCC
>S18
???

???
???
???
???
???
????????AGTCATCCTCGCAATCCC-ATCATCA--------------------------TCATCACCT--
CGCAAA----CA-TTTCCACGCC-GGTGCCGAAAATCTGG--------TTTTCGCACCTGCC-
CATTTTCCCGG--ACACTTACCCC--------
GCCGCACGACCCCGCGGTGCAAACGAAAAATTTCTTA--
TCATAGCCCCACATCACACAAACATTTTGGCAGCCACGCACTTTGCAAGACCCACAACGAACAA
TTGCTGACCCCGCCAAATAGGAAGCCGCCGAGCTCGGAAAGGGTTCCTTCAAGTACGCATGGG
TTCTTGACAAGCTCAAGGCCGAGCGTGAGCGTGGTATCACCATTGATATCGCTCTCTGGAAGTT
CGAGACCAACGAGTACAATGTCACCGTCATTGGTTAGTATCCCCGTCCACAACATGCATCATGT
CTCCGAACTCAAGACTAACCTTGCCATACAGATGCTCCCGGTCACCGTGATTTCATCAATGCTGC
CTTCTGGTATGTAGCC-CATCTACCCCGACGC-GTCTCAATACGACA-
CCCCCGGCAACTCGACAAC--GACGTTCTCAAC---
TGCTTGGTTGAAACCAAATGAAAGACTTGATACTGACCGGTCTCTGATAGGCAAACCATCTCTG
GCGAGCACGGTCTCGACAGCAATGGAGTGTACGTACCCTTTCCCTGGCTACTCGCTTTCTCGTG
AACATGTCAGCTAACACTCG-
TGCTTGTTCAGCTACAACGGTACCTCCGAGCTCCAGCTCGAGCGCATGAGCGTCTACTTCAACG
AGGCTTCCGGCAACAAGTACGTTCCTCGTGCCGTCCTCGTCGATCTCGAGCCCGGTACCATGGA
TGCCGTCCGCGCCGGTCCTTTCGGTCAGCTCTTCCGCCCTGACAACTTCGTCT?????????????????
